# Global burden, quality of care, and cross-country inequalities in Alzheimer’s disease and other dementias from 1990 to 2021

**DOI:** 10.7189/jogh.15.04269

**Published:** 2025-10-03

**Authors:** Yajie Zhu, Siqing Cheng, Zeyu Luo, Jie Shen, Jin Cao, Lingzi Yao, Jiali Zhou, Shiyi Shan, Peige Song, Changzheng Yuan

**Affiliations:** 1 School of Information Science and Technology, Hangzhou Normal University, Hangzhou, China; 2 School of Public Health and The Second Affiliated Hospital, Zhejiang University School of Medicine, Hangzhou, China; 3 Centre for Global Health, Usher Institute of Population Health Sciences and Informatics, University of Edinburgh, Edinburgh, UK; 4 Department of Nutrition, Harvard T.H. Chan School of Public Health, Boston, Massachusetts, USA

## Abstract

**Background:**

As global populations age, the burden of dementia increases, raising challenges for healthcare systems. Care quality is key to supporting individuals with dementia, but few studies have assessed this at a global level. We aimed to assess the burden and care quality of Alzheimer’s disease and related dementias (ADRDs) and cross-country inequalities between 1990 and 2021.

**Methods:**

We conducted a secondary analysis of the Global Burden of Diseases Study (GBD) 2021, focussing on disability-adjusted life-years (DALYs) and care quality (measured by a variational autoencoder-based Quality of Care Index (QCI-v), range = 1–100) of ADRDs. We assessed trends using average annual percentage changes (AAPCs) and measured cross-country inequalities using the slope index of inequality and concentration index.

**Results:**

The global age-standardised DALY rates of ADRDs per 100 000 population slightly increased from 1331.59 (95% confidence interval (CI) = 881.57, 1781.61) in 1990 to 1347.24 (95% CI = 906.36, 1788.12) in 2021, with an AAPC of 0.04% (95% CI = 0.02, 0.05). The global age-standardised QCI-v of ADRDs increased from 51.56 (95% CI = 45.87, 57.26) to 54.27 (95% CI = 48.68, 59.86), with an AAPC of 0.16% (95% CI = 0.12, 0.21). Regions with a low-middle or low sociodemographic index (SDI), as well as the African Region and the South-East Asia Region, experienced an increasing burden, but a decreasing care quality. In 2021, high-middle SDI regions and the Western Pacific Region had the highest burden, while care quality was notably low in low SDI region. The European Region showed the greatest inequality in terms of burden, while the Eastern Mediterranean showed marked disparities in care quality.

**Conclusions:**

The disease burden and care quality of ADRDs both increased from 1990 to 2021, accompanied by significant disparities between countries. Regions with low-middle or low SDI, particularly the African and South-East Asia Regions, faced rising burdens and declining care quality.

Dementia, characterised by the progressive decline in cognitive functions such as memory, language, problem-solving, and other thinking abilities, significantly impairs daily life [1–3]. Alzheimer’s disease and related dementias (ADRDs) are the most prevalent forms of this neurodegenerative condition [4,5]. The World Health Organization (WHO) estimated that approximately 55 million individuals globally were affected by dementia in 2019, with projections reaching 139 million by 2050 [6]. The socioeconomic impact of ADRDs is significant, extending beyond affected individuals to their families, healthcare systems, and broader societal structures, with global costs estimated at USD 1.3 trillion in 2019 [7]. Notably, the burden of ADRDs is unevenly distributed across different ages, sexes, and particularly among countries [5,8], underscoring the need to explore how socioeconomic factors and variations in healthcare systems contribute to cross-country inequalities in ADRD burden.

Care quality is essential for supporting the well-being of individuals living with ADRDs [3]. It encompasses a broad spectrum of services, including medical care, formal and informal long-term care, hospice care, and end-of-life support [3,9]. However, substantial variations in care quality exist across countries [10]. Healthcare professionals in low- and middle-income countries, for example, often receive limited training in the diagnosis and treatment of ADRDs, which adversely affects the quality and accessibility of care [11,12]. Inadequate healthcare infrastructure, limited access to trained professionals, and insufficient resources for effective management likewise pose significant barriers to addressing the challenges associated with ageing and ADRDs [12,13]. Understanding these inequalities in care quality is essential for optimising health resource allocation and improving global health governance.

The Global Burden of Diseases (GBD) 2021 study provides a comprehensive framework for quantifying the epidemiology and burden of diseases, including ADRDs [14,15]. Its data can therefore be utilised to monitor health inequalities by describing and analysing various health indicators across different demographic levels and geographic regions. Addressing these inequalities is crucial for informing equity-oriented health policies, programmes, and practices and improving care for ADRDs globally.

In this study, we used data from the GBD 2021 to describe the disease burden and care quality of ADRDs from 1990 to 2021, as well as examined, through WHO-recommended standard health equity analytic methods, whether sociodemographic development level-related inequalities exist in the burden and care quality of ADRDs across countries.

## METHODS

Our study adheres to the Journal of Global Health’s (JoGH) Guidelines for Reporting Analyses of Big Data Repositories Open to Public (GRABDROP) guidelines (Table S1 in the **Online Supplementary Document**).

### Data sources and case definition

We conducted a secondary analysis using data from the GBD 2021, which provides the most recent comprehensive, standardised assessment of all-cause and cause-specific incidence, prevalence, mortality, and disability for 371 diseases and injuries and 88 risk factors for 204 countries and territories during 1990–2021 [14–16]. The GBD utilises various data sources, including literature, civil registration and vital statistics, household surveys, and hospital records. All data sources used in this analysis are available on the Global Health Data Exchange (see data availability statement below). Within the GBD study, they are reported by super-region, region, and country, with super-regions based on epidemiological similarity and geographical closeness. Where data for individual locations were not available, prevalence estimates were modeled with available data from super-region priors. DisMod-MR 2.1, a Bayesian meta-regression tool, was run as a geographical cascade. First, a model was run with all global data. Using random effects and predictive covariates, the results of this initial model were passed down to the next level models by super-region as Bayesian priors [15]. This was repeated for regional, country, and, where applicable, subnational models (eMethods in the **Online Supplementary Document**).

We also determined each country’s sociodemographic index (SDI), which quantifies the sociodemographic development level of a country or territory based on income, education, and fertility conditions. The SDI ranges from 0 to 1, with 0 representing the theoretical minimum level of development relevant to health and 1 the theoretical maximum level. Based on the country-level SDI estimates for the year 2021, 204 countries and territories were classified into five quintiles (low, low-middle, middle, high-middle, and high) within the GBD study [15].

Within the GBD 2021 framework, ADRDs are defined as a progressive, degenerative, and chronic neurological disorder characterised by memory impairment and other neurological dysfunctions, with the Diagnostic and Statistical Manual of Mental Disorders III, IV, or V, or ICD case definitions serving as the reference. The eligibility criteria, the literature search strategy, data input, as well as a flowchart for estimating ADRDs fatal and non-fatal burden within the GBD study are provided in the eMethods in the **Online Supplementary Document**.

We extracted age-, sex- and location-specific estimates and 95% uncertainty intervals of incidence, prevalence, mortality, years of life lost (YLLs), years lived with disability (YLDs), and disability-adjusted life-years (DALYs) for ADRDs from 1990 to 2021 for 204 countries and territories. Age categories were formed in five-year intervals, starting from the age of 40 years up to ages 95 and above.

### Burden and care quality of ADRDs

We used age-standardised DALY rates from 1990 to 2021 to represent the burden of ADRDs at the global, regional, and national levels. We determined the care quality of ADRDs using a variational autoencoder-based Quality of Care Index (QCI-v). This metric and its 95% confidence interval (CI) were derived based on a novel neural network model that incorporated variational autoencoder into the principal component analysis framework by utilising the similarity between a one-layer linear autoencoder and the principal component analysis [17]. Our model included two linear encoders and one linear decoder. The first encoder mapped four meaningful secondary indices into the mean of the latent variable, while the second encoder mapped the uncertainty of the four indices into the variance of the latent variable. The four secondary indices were:

1. Mortality-to-incidence ratio: this reflects survival rates post-diagnosis and the effectiveness of care interventions.
2. DALY-to-prevalence ratio: this measures the burden of the disease relative to its prevalence, indicating the efficiency and effectiveness of healthcare interventions.
3. Incidence-to-prevalence ratio: this shows how well new cases are being managed and controlled within the population.
4. YLLs-to-YLDs ratio: this represents the balance between mortality and morbidity, indicating the focus of healthcare on prolonging life vs improving quality of life.

These metrics collectively provide a robust framework for evaluating disease-specific care quality, reflecting the effectiveness of healthcare interventions from both mortality and morbidity perspectives. We used the bootstrap method to compute the 95% CI of the four secondary indices. The model sampled from the latent space and used the decoder to reconstruct the input variables. It learned a latent distribution of the original input features and was trained by minimising the reconstruction error and the Kullback-Leibler divergence between the learned latent distribution and a prior Gaussian distribution using the Adam optimiser with a learning rate of 0.001 for 10 000 epochs. The QCI-v was constructed by rescaling the latent variable into the range of 1 to 100, with higher values indicating better care quality.

### Statistical analysis

#### Description and trend analysis

We used the population weights from the GBD 2021 as the standard population weights to calculate the age-standardised rates for all measures. In order to estimate the 95% CI of age-standardised rates, we first calculated the standard deviations of the age-specific rates by treating them as log-normal distribution, and then combined these standard deviations using the Delta method:



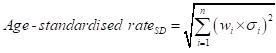



where *w_i_* and *σ_i_* and are the standard population weight and the standard deviation of the age-specific rate for age group *i*, respectively.

We described the distribution of age-standardised DALY rates per 100 000 population and age-standardised QCI-v for ADRDs across sexes, different global regions, and 204 countries/territories from 1990 to 2021. We used Welch’s *t*-test to compare the age-standardised DALY rates and age-standardised QCI-v among males and females. We used the Kruskal-Wallis test to assess differences across SDI regions and WHO regions, after which we used *post-hoc* analysis to determine the region with the highest value and compare it to others *via* Dunn’s test. We adjusted our analysis for multiple comparisons through the Bonferroni method.

We estimated average annual percentage changes (AAPCs) by joinpoint regression to measure the average temporal trend in burden and care quality over between 1990 and 2021, with the AAPC representing the annual change percentage transformed from the weighted average of the slope coefficients of the underlying joinpoint regression model in the study period [18]. When the AAPC and its lower limit of 95% CI were both positive, the age-standardised DALY rates and age-standardised QCI-v were considered to be increasing, and *vice versa*; if the estimated CIs overlapped zero, the age-standardised DALY rates and age-standardised QCI-v were deemed to be stable. This followed the formula:



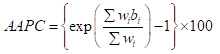



where *b_i_* is the slope coefficient for the *i^th^* segment with *i* indexing the segments in the desired range of years, and *w_i_* is the length of each segment in the range of years.

### Cross-country inequality analysis

We adopted the slope index of inequality (SII) and the concentration index, which are standard metrics of absolute and relative gradient inequality, to quantify the distributive inequality in the burden and care quality of ADRDs across countries [19]. We calculated the SII by regressing national age-standardised DALY rates and age-standardised QCI-v on an SDI-associated relative position scale, which was defined by the midpoint of the cumulative range of the population ranked by the SDI in this country. Heteroskedasticity was addressed using a robust regression model. We calculated the concentration index by numerically integrating the area under the Lorenz concentration curve, fitted using the cumulative fraction of DALYs and QCI-v against the cumulative relative distribution of the population ranked by SDI. This index ranges from −1 (perfect inequality favouring the lowest SDI) to 1 (perfect inequality favouring the highest SDI). For DALYs of ADRDs, a negative SII/concentration index indicated that lower SDI countries bear a higher burden, and *vice versa*. For QCI-v of ADRDs, a positive SII/concentration index represents lower care quality in lower SDI countries. A larger absolute value of the SII/concentration index indicated greater inequality.

We performed all descriptive, trend, and inequality analyses for the overall population, and for males and females separately. We also stratified all analyses by five SDI regions (high, high-middle, middle, low-middle, and low) and six WHO regions (African region, Eastern Mediterranean region, South-East Asia region, Region of the Americas, Western Pacific Region, and European region) to better understand variations in the burden, care quality, and inequalities at the regional level.

We conducted all statistical analyses using Python, version 3.9.11 (Python Software Foundation, Oregon, USA) and *R*, version 4.3.3 (R Foundation for Statistical Computing, Vienna, Austria). Age-standardised DALY rates, QCI-v, SII, concentration index, AAPC were all presented with their respective 95% CIs. All statistical tests were two-sided, and *P*-values <0.05 were considered statistically significant.

## RESULTS

### Disease burden of ADRDs from 1990 to 2021

**Figure 1:**
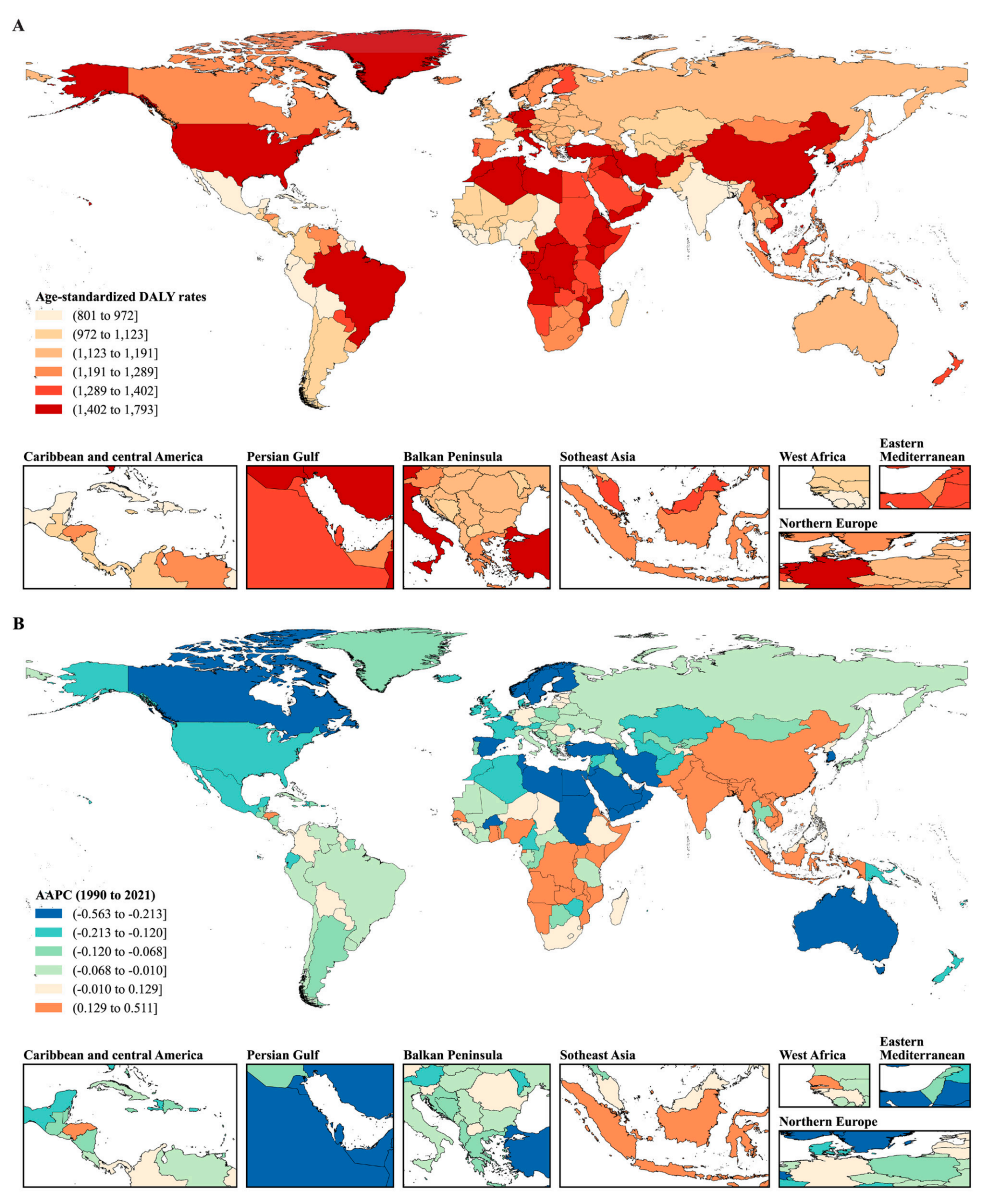
Age-standardised DALY rates. **Panel A.** ADRDs across all countries in 2021. **Panel B.** AAPC from 1990 to 2021. AAPC – average annual percentage change, ADRDs – Alzheimer's disease and related dementias, DALY – disability-adjusted life year.

The age-standardised DALY rates of ADRDs per 100 000 population increased slightly from 1331.59 (95% CI = 881.57, 1781.61) from 1990 to 1347.24 (95% CI = 906.36, 1788.12) in 2021, with an AAPC of 0.04% (95% CI = 0.02, 0.05) (Figures S1 and S2, and Table S2 in the **Online Supplementary Document**). The increasing trend was more pronounced among males (AAPC = 0.08%; 95% CI = 0.06, 0.09) compared to females (AAPC = 0.06%; 95% CI = 0.03, 0.09). In 2021, the age-standardised DALY rates per 100 000 population were higher among females (1508.22; 95% CI = 1031.91, 1984.54) compared to males (1112.87; 95% CI = 724.07, 1501.66). Significant increases in the burden of ADRDs were observed in nearly all SDI regions, except for the high SDI region (AAPC = −0.10%; 95% CI = −0.11, −0.09). Among WHO regions, significant increases were noted in South-East Asia and Africa, with AAPCs of 0.34% (95% CI = 0.30, 0.38) and 0.20% (95% CI = 0.19, 0.22), respectively. In 2021, the age-standardised DALY rates of ADRDs per 100 000 population were the highest in the high-middle SDI region (1439.00; 95% CI = 965.91, 1912.09) and the Western Pacific region (1574.93; 95% CI = 1065.35, 2084.50). By 2021, the five countries with the highest burden of ADRDs were Democratic Republic of the Congo, Gabon, Afghanistan, Congo and Angola (**Figure 1**; Table S4 in the **Online Supplementary Document**).

### Care quality of ADRDs from 1990 to 2021

**Figure 2:**
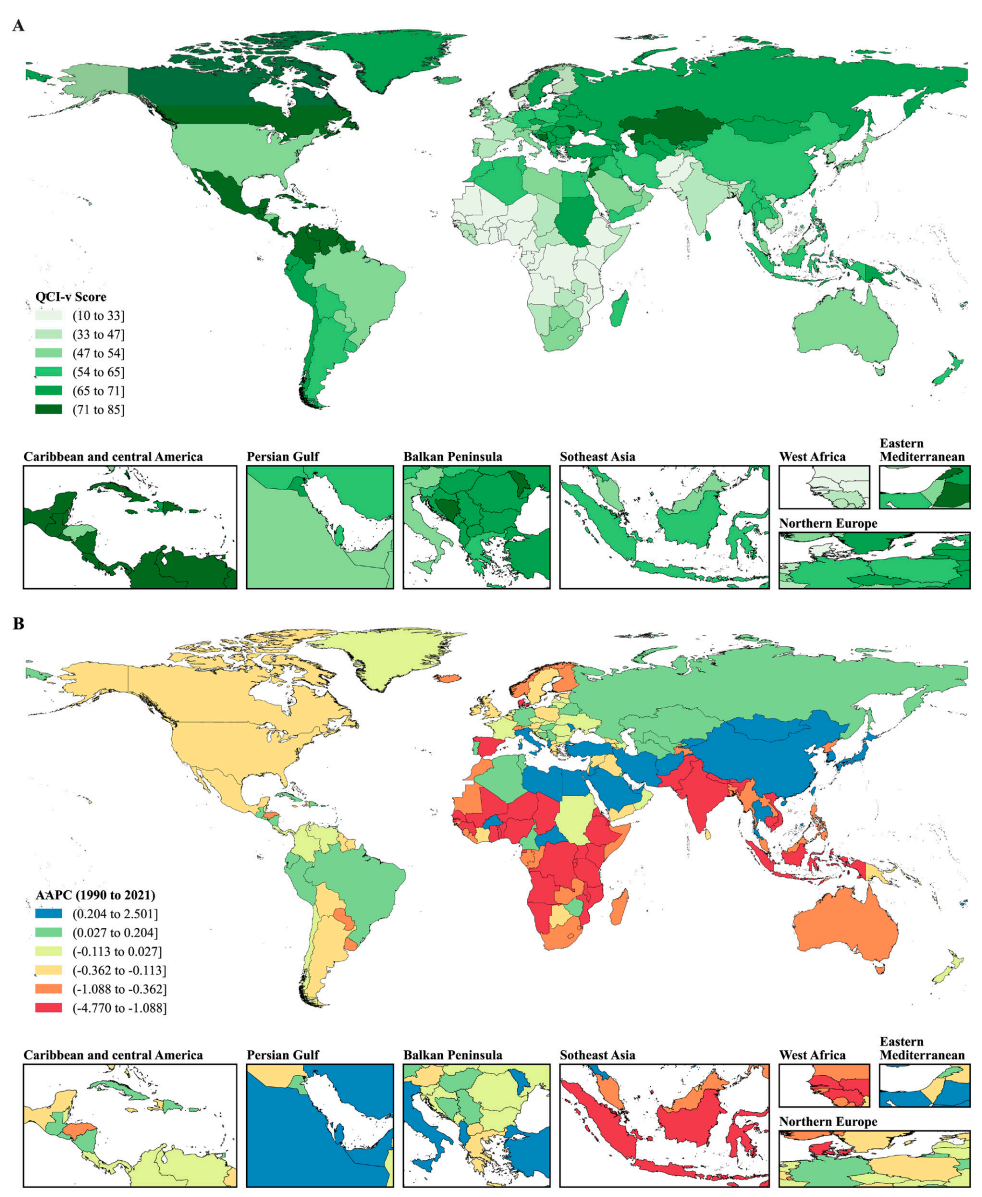
**Panel A.** Age-standardised QCI-v of ADRDs across all countries in 2021 **Panel B.** AAPC from 1990 to 2021. AAPC – average annual percentage change, ADRDs – Alzheimer's disease and related dementias, QCI-v – quality of care index based on variational autoencoders.

The age-standardised QCI-v of ADRDs slightly increased from 51.56 (95% CI = 45.87, 57.26) in 1990 to 54.27 (95% CI = 48.68, 59.86) in 2021, with an AAPC of 0.16% (95% CI = 0.12, 0.21) (Figures S3 and S4, and Table S3 in the **Online Supplementary Document**). Improvements were more pronounced among females (AAPC = 0.19%; 95% CI = 0.14, 0.23) than males (AAPC = 0.04%; 95% CI = 0.02, 0.07). The age-standardised QCI-v in 2021 was higher among females (57.67; 95% CI = 52.23, 63.11) compared to males (56.25; 95% CI = 51.31, 61.20). Regarding the SDI, the age-standardised QCI-v increased in high-middle and middle SDI regions, but decreased in low-middle and low SDI regions, with AAPCs of −1.01% (95% CI = −1.15, −0.87) and −1.55% (95% CI = −1.72, −1.38) respectively. In terms of WHO regions, the age-standardised QCI-v increased in the Western Pacific, but decreased in Africa (AAPC = −1.44%; 95% CI = −1.49, −1.39), South-East Asia (AAPC = −1.16%; 95% CI = −1.33, −0.99), the Eastern Mediterranean (AAPC = −0.10%; 95% CI = −0.14%, −0.06) and the Americas (AAPC = −0.05%; 95% CI = −0.08, −0.02). In 2021, the lowest age-standardised QCI-v of ADRDs was observed in the low-SDI regions (31.16; 95% CI = 24.48, 37.84) and the African region (29.41; 95% CI = 22.72, 36.11), with Eritrea, Mali, Ethiopia, Nigeria, Mozambique having the lowest care quality for ADRDs (**Figure 2**; Table S5 in the **Online Supplementary Document**).

### Cross-country inequalities in the burden of ADRDs

**Figure 3:**
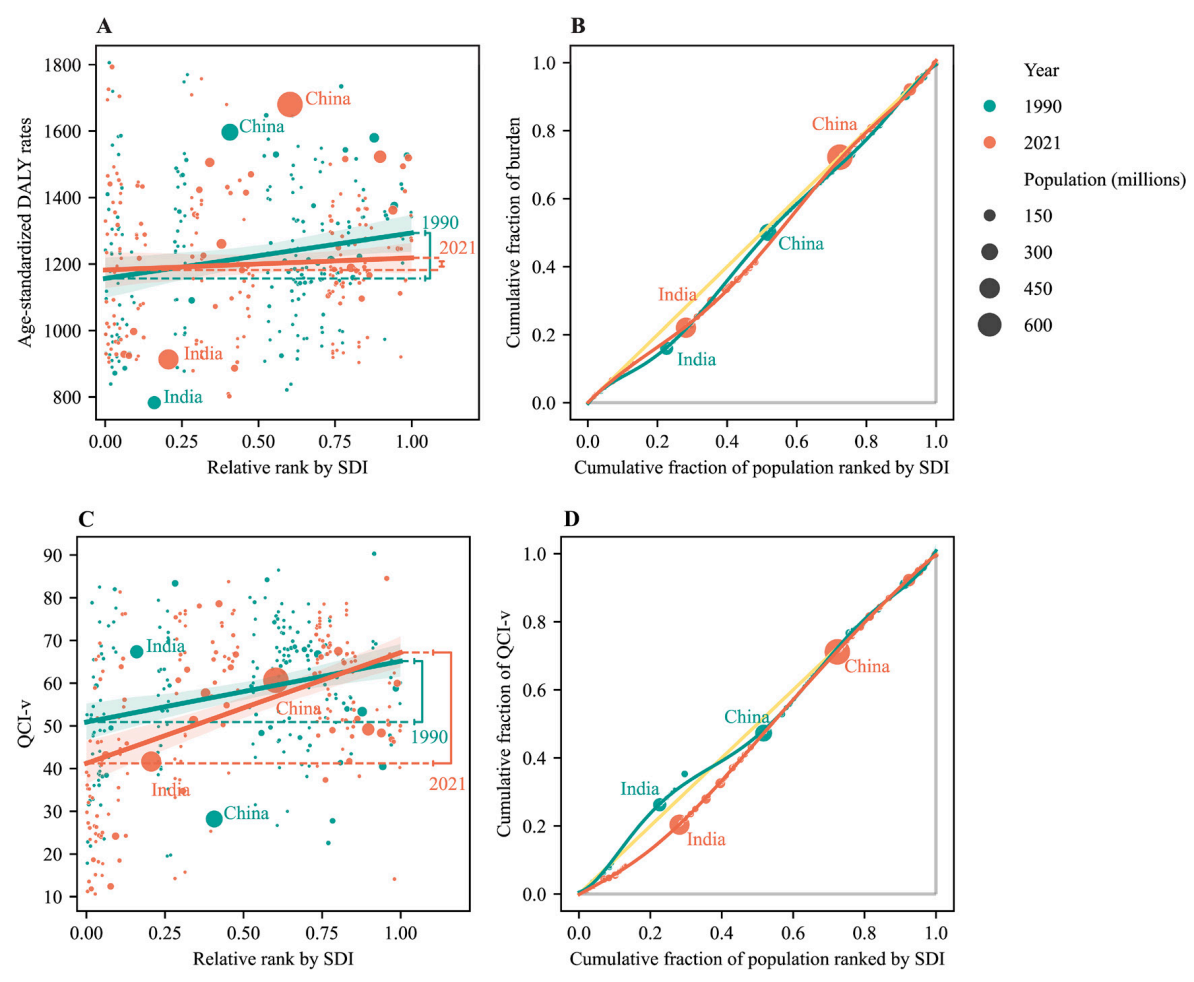
Absolute and relative inequalities in the age-standardised DALY rates and age-standardised QCI-v of ADRDs worldwide, 1990 and 2021. **Panel A.** The slope index of inequality, shown as the slope of the regression line, represents the absolute difference in ADRDs burden between countries or territories with the highest and lowest SDI. **Panel B.** Concentration curves (Lorenz curves) graphed by plotting the cumulative fraction of population ranked by SDI (x-axis) against the cumulative fraction of ADRDs care quality ranked by age-standardised DALY (y-axis). **Panel C.** The slope index of inequality, shown as the slope of the regression line, represents the absolute difference in ADRDs care quality between countries or territories with the highest and lowest SDI. **Panel D.** Concentration curves (Lorenz curves) graphed by plotting the cumulative fraction of population ranked by SDI (x-axis) against the cumulative fraction of ADRDs burden ranked by age-standardised QCI-v (y-axis). The concentration index, calculated as twice the area between the 45° diagonal line and the Lorenz curve, representing the relative extent to which the ADRDs burden or care quality is concentrated among the poor (negative value) or the rich (positive value). Dots representing countries or territories, with different sizes representing the population sizes. ADRDs – Alzheimer's disease and related dementias, DALY – disability-adjusted life-year, SDI – sociodemographic index.

The SII of age-standardised DALY rates for ADRDs narrowed from 136.93 (95% CI = 36.83, 237.02) in 1990 to 36.48 (95% CI = −47.49, 120.45) in 2021. Disparities were significant among males (SII = 122.50; 95% CI = 56.69, 188.31) in 2021. Across WHO regions, the SII was significantly positive among European countries (110.61; 95% CI = 48.51, 172.70) in 2021. Moreover, the concentration index, which measures relative inequality, increased slightly from 0.0604 (95% CI = 0.0427, 0.0781) in 1990 to 0.0639 (95% CI = 0.0470, 0.0809) in 2021. Looking at differences in sex, the concentration index was lower among females (0.0557; 95% CI = 0.0383, 0.0731) compared to males (0.0696; 95% CI = 0.0530, 0.0861) in 2021. Looking at SDI and WHO regions, the concentration index was the highest in the high-middle SDI regions, with a negative value of −0.0971 (95% CI = −0.1191, −0.0750), and was significantly positive in the Eastern Mediterranean region, with a value of 0.0443 (95% CI = 0.0074, 0.0811) (**Figure 3****,** **Table 1**; Figures S5–S8, Tables S6 and S7 in the **Online Supplementary Document**).

**Table 1:** The SII and concentration index for age-standardised DALY rates of ADRDs by sex and region, 1990–2021, presented as point estimate (95% confidence interval)

	SII						Concentration index					
	**Both**		**Female**		**Male**		**Both**		**Female**		**Male**	
	**1990**	**2021**	**1990**	**2021**	**1990**	**2021**	**1990**	**2021**	**1990**	**2021**	**1990**	**2021**
**Global**	136.93 (36.83, 237.02)	36.48 (−47.49, 120.45)	47.12 (−69.33, 163.56)	−25.04 (−123.56, 73.49)	188.74 (114.99, 262.48)	122.50 (56.69, 188.31)	0.0604 (0.0427, 0.0781)	0.0639 (0.0470, 0.0809)	0.0450 (0.0273, 0.0627)	0.0557 (0.0383, 0.0731)	0.0628 (0.0456, 0.0800)	0.0696 (0.0530, 0.0861)
**SDI regions**												
High SDI	69.63 (−33.58, 172.85)	101.20 (−37.02, 239.42)	134.66 (17.10, 252.22)	135.43 (−32.31, 303.18)	−88.01 (−242.31, 66.30)	69.25 (−59.77, 198.28)	−0.0450 (−0.0565, −0.0335)	0.0329 (0.0112, 0.0546)	−0.0346 (−0.0455, −0.0236)	0.0369 (0.0153, 0.0585)	−0.0484 (−0.0558, −0.0409)	0.0176 (−0.0072, 0.0425)
High-middle SDI	43.25 (−128.93, 215.42)	−103.67 (−320.63, 113.29)	36.35 (−126.09, 198.79)	−153.39 (−437.70, 130.92)	82.14 (−77.94, 242.23)	−25.31 (−199.11, 148.48)	0.0198 (−0.0142, 0.0538)	−0.0971 (−0.1191, −0.0750)	0.0257 (−0.0063, 0.0576)	−0.1052 (−0.1289, −0.0815)	0.0116 (−0.0331, 0.0563)	−0.0954 (−0.1160, −0.0748)
Middle SDI	56.54 (−129.79, 242.87)	−121.72 (−311.97, 68.53)	87.99 (−188.72, 364.70)	−157.69 (−413.87, 98.49)	43.88 (−92.66, 180.43)	−85.29 (−248.02, 77.43)	0.0491 (0.0256, 0.0726)	−0.0433 (−0.0728, −0.0137)	0.0490 (0.0220, 0.0760)	−0.0417 (−0.0707, −0.0126)	0.0387 (0.0209, 0.0565)	−0.0387 (−0.0716, −0.0058)
Low-middle SDI	−55.92 (−228.18, 116.34)	79.26 (−97.87, 256.39)	−52.38 (−267.45, 162.69)	27.42 (−170.22, 225.06)	−67.56 (−192.83, 57.72)	117.86 (−26.32, 262.04)	−0.0352 (−0.0623, −0.0082)	−0.0126 (−0.0517, 0.0265)	−0.0322 (−0.0584, −0.0060)	−0.0267 (−0.0641, 0.0107)	−0.0449 (−0.0733, −0.0165)	0.0103 (−0.0326, 0.0533)
Low SDI	327.29 (129.07, 525.50)	132.93 (−173.78, 439.63)	261.08 (30.73, 491.44)	80.53 (−294.78, 455.85)	372.16 (207.64, 536.69)	147.22 (−34.91, 329.34)	0.1565 (0.1278, 0.1852)	0.0031 (−0.0428, 0.0491)	0.1416 (0.1117, 0.1716)	−0.0053 (−0.0534, 0.0428)	0.1516 (0.1251, 0.1781)	0.0092 (−0.0294, 0.0478)
**GBD regions**												
AFR	158.55 (−76.17, 393.27)	166.06 (−81.35, 413.47)	115.53 (−172.17, 403.23)	126.87 (−174.00, 427.75)	96.48 (−60.51, 253.47)	82.10 (−70.10, 234.31)	−0.0142 (−0.0507, 0.0223)	−0.0204 (−0.0597, 0.0189)	−0.0199 (−0.0588, 0.0189)	−0.0312 (−0.0698, 0.0074)	−0.0076 (−0.0445, 0.0294)	−0.0079 (−0.0455, 0.0296)
EMR	109.65 (7.51, 211.79)	−26.68 (−105.58, 52.22)	120.61 (44.91, 196.30)	−8.33 (−66.37, 49.71)	80.66 (−20.45, 181.77)	53.16 (−42.72, 149.05)	0.0610 (0.0073, 0.1147)	0.0443 (0.0074, 0.0811)	0.0437 (−0.0089, 0.0963)	0.0333 (−0.0024, 0.0689)	0.0747 (0.0209, 0.1285)	0.0644 (0.0260, 0.1028)
EUR	198.03 (118.59, 277.47)	110.61 (48.51, 172.70)	240.97 (147.07, 334.88)	146.56 (58.20, 234.92)	135.34 (74.33, 196.35)	77.55 (27.83, 127.27)	0.0234 (0.0041, 0.0427)	0.0105 (−0.0093, 0.0304)	0.0263 (0.0050, 0.0476)	0.0127 (−0.0093, 0.0347)	0.0087 (−0.0092, 0.0267)	0.0073 (−0.0108, 0.0254)
AMR	101.20 (−106.83, 309.22)	−1.13 (−171.23, 168.96)	161.32 (−75.02, 397.67)	6.58 (−214.76, 227.92)	−20.93 (−138.20, 96.33)	−59.40 (−165.87, 47.07)	0.0671 (0.0260, 0.1083)	0.0423 (−0.0036, 0.0882)	0.0617 (0.0225, 0.1009)	0.0471 (0.0023, 0.0918)	0.0616 (0.0194, 0.1038)	0.0354 (−0.0118, 0.0827)
SEAR	208.50 (−7.18, 424.17)	95.46 (−112.04, 302.96)	202.60 (−85.80, 491.00)	96.51 (−145.35, 338.36)	139.95 (−46.68, 326.59)	98.86 (−30.86, 228.58)	0.0925 (0.0094, 0.1756)	0.0663 (−0.0025, 0.1351)	0.0893 (0.0056, 0.1730)	0.0650 (−0.0047, 0.1348)	0.0769 (0.0059, 0.1478)	0.0616 (0.0045, 0.1188)
WPR	51.29 (−63.62, 166.21)	−48.99 (−150.73, 52.75)	93.23 (−40.85, 227.32)	−12.36 (−132.64, 107.92)	−12.85 (−105.64, 79.94)	−81.87 (−151.74, −11.99)	−0.0378 (−0.0655, −0.0102)	−0.0172 (−0.0551, 0.0208)	−0.0322 (−0.0589, −0.0056)	−0.0129 (−0.0499, 0.0241)	−0.0542 (−0.0785, −0.0298)	−0.0268 (−0.0685, 0.0150)

ADRDs – Alzheimer's disease and related dementias, AFR – African Region, AMR – Region of the Americas, DALY – disability-adjusted life year, EMR – Eastern Mediterranean Region, EUR – European Region, SDI – sociodemographic index, SEAR – South-East Asia Region, SII – Slope Index of Inequality, WPR – Western Pacific Region

### Cross-country inequalities in the care quality of ADRDs

The SII of age-standardised QCI-v for ADRDs increased from 1990 to 2021, with the gap between the highest and lowest SDI countries widening from 14.26 (95% CI = 7.49, 21.03) to 25.96 (95% CI = 18.78, 33.14). The SII of age-standardised QCI-v was higher among females (26.22; 95% CI = 19.29, 33.15) than males (19.74; 95% CI = 13.70, 25.78) in 2021. Meanwhile, the SII of age-standardised QCI-v was the highest in the low-middle SDI region, with a positive value of 16.89 (95% CI = 1.49, 32.30). Among WHO regions, the SII was the highest in the Eastern Mediterranean region, with a positive value of 26.65 (95% CI = 9.97, 43.33). During the same period, the concentration index of the age-standardised QCI-v for ADRDs rose significantly, increasing from −0.0027 (95% CI = −0.0319, 0.0265) to 0.0760 (95% CI = 0.0557, 0.0963). In 2021, the concentration index was higher among females (0.0910; 95% CI = 0.0708, 0.1112) than males (0.0358; 95% CI = 0.0199, 0.0518). It was significantly positive in middle, low-middle, and low SDI regions, ranging from 0.0562 (95% CI = 0.0224, 0.0901) to 0.1179(95% CI = 0.0085, 0.2274), and was the highest in the Eastern Mediterranean region, with a positive value of 0.1470 (95% CI = 0.0721, 0.2219) (**Figure 3****,** **Table 2**; Figures S9–S12, Tables S8 and S9 in the **Online Supplementary Document**).

**Table 2:** SII and concentration index for age-standardised QCI-v of ADRDs by sex and region, 1990–2021, presented as point estimate (95% confidence interval)

	SII						Concentration index					
	**Both**		**Female**		**Male**		**Both**		**Female**		**Male**	
	**1990**	**2021**	**1990**	**2021**	**1990**	**2021**	**1990**	**2021**	**1990**	**2021**	**1990**	**2021**
**Global**	14.26 (7.49, 21.03)	25.96 (18.78, 33.14)	13.64 (6.96, 20.32)	26.22 (19.29, 33.15)	11.80 (6.18, 17.42)	19.74 (13.70, 25.78)	−0.0027 (−0.0319, 0.0265)	0.0760 (0.0557, 0.0963)	0.0037 (−0.0206, 0.0279)	0.0910 (0.0708, 0.1112)	−0.0003 (−0.0271, 0.0266)	0.0358 (0.0199, 0.0518)
**SDI regions**												
High SDI	−7.51 (−15.24, 0.23)	−12.12 (−21.96, −2.28)	−7.17 (−16.13, 1.79)	−12.05 (−21.23, −2.88)	−6.14 (−13.70, 1.42)	−8.20 (−16.49, 0.09)	0.0079 (0.0009, 0.0149)	0.0112 (−0.0252, 0.0476)	0.0160 (0.0079, 0.0241)	0.0019 (−0.0284, 0.0321)	−0.0231 (−0.0278, −0.0183)	0.0386 (0.0015, 0.0756)
High-middle SDI	−2.87 (−17.23, 11.50)	−3.14 (−17.18, 10.90)	−1.68 (−15.33, 11.96)	−1.27 (−15.66, 13.13)	−6.86 (−19.63, 5.91)	−5.56 (−17.51, 6.40)	−0.0200 (−0.0888, 0.0489)	−0.0076 (−0.0335, 0.0183)	−0.0167 (−0.0793, 0.0459)	−0.0191 (−0.0410, 0.0028)	−0.0163 (−0.0688, 0.0362)	0.0187 (−0.0080, 0.0455)
Middle SDI	−8.69 (−24.52, 7.13)	9.06 (−2.06, 20.17)	−8.27 (−23.85, 7.32)	10.02 (−2.67, 22.70)	−8.07 (−17.36, 1.23)	6.66 (−2.54, 15.86)	−0.1083 (−0.1582, −0.0584)	0.0562 (0.0224, 0.0901)	−0.0963 (−0.1410, −0.0516)	0.0608 (0.0284, 0.0932)	−0.0724 (−0.1077, −0.0371)	0.0296 (0.0018, 0.0575)
Low-middle SDI	1.67 (−9.60, 12.94)	16.89 (1.49, 32.30)	1.50 (−9.55, 12.55)	17.59 (1.09, 34.10)	2.53 (−7.92, 12.97)	12.56 (0.46, 24.66)	0.0271 (−0.0050, 0.0592)	0.1049 (0.0347, 0.1751)	0.0274 (−0.0018, 0.0567)	0.0987 (0.0170, 0.1804)	0.0224 (−0.0052, 0.0500)	0.0809 (0.0398, 0.1221)
Low SDI	13.29 (−1.21, 27.78)	14.71 (−4.27, 33.68)	12.84 (−1.12, 26.81)	16.36 (−1.24, 33.97)	9.71 (−1.52, 20.93)	10.56 (−5.67, 26.78)	−0.1699 (−0.2232, −0.1166)	0.1179 (0.0085, 0.2274)	−0.1230 (−0.1677, −0.0783)	0.1198 (0.0214, 0.2182)	−0.1699 (−0.2187, −0.1212)	0.0712 (−0.0049, 0.1473)
**WHO regions**												
AFR	13.72 (3.27, 24.17)	14.37 (1.90, 26.85)	11.67 (0.76, 22.58)	13.44 (0.42, 26.45)	14.30 (5.17, 23.42)	16.81 (4.30, 29.31)	0.0829 (0.0406, 0.1252)	0.1341 (0.0370, 0.2313)	0.0759 (0.0391, 0.1128)	0.1101 (0.0244, 0.1958)	0.0528 (0.0116, 0.0940)	0.1030 (0.0398, 0.1662)
EMR	−0.19 (−14.35, 13.98)	26.65 (9.97, 43.33)	0.94 (−13.17, 15.05)	26.47 (6.77, 46.17)	0.10 (−11.12, 11.33)	14.24 (2.04, 26.44)	0.0354 (−0.0113, 0.0820)	0.1470 (0.0721, 0.2219)	0.0808 (0.0135, 0.1482)	0.2066 (0.1065, 0.3067)	−0.0061 (−0.0351, 0.0228)	0.0598 (0.0213, 0.0984)
EUR	−10.04 (−16.19, −3.90)	−16.62 (−24.12, −9.12)	−8.91 (−14.08, −3.73)	−14.59 (−21.17, −8.01)	−9.70 (−15.35, −4.06)	−15.50 (−22.42, −8.59)	−0.0477 (−0.0810, −0.0144)	−0.0357 (−0.0659, −0.0056)	−0.0434 (−0.0739, −0.0128)	−0.0301 (−0.0550, −0.0052)	−0.0302 (−0.0550, −0.0055)	−0.0256 (−0.0560, 0.0047)
AMR	5.06 (−6.70, 16.82)	2.25 (−7.77, 12.26)	4.81 (−6.92, 16.53)	1.49 (−6.28, 9.26)	4.31 (−6.11, 14.74)	6.25 (−3.85, 16.36)	−0.0140 (−0.0680, 0.0400)	−0.0285 (−0.0795, 0.0225)	−0.0113 (−0.0627, 0.0400)	−0.0073 (−0.0555, 0.0410)	−0.0087 (−0.0513, 0.0340)	−0.0554 (−0.0998, −0.0110)
SEAR	−9.74 (−27.56, 8.08)	17.21 (−5.72, 40.14)	−8.78 (−24.84, 7.28)	17.31 (−7.41, 42.03)	−9.60 (−31.31, 12.11)	15.15 (−7.32, 37.62)	0.0126 (−0.0586, 0.0839)	0.0739 (−0.0053, 0.1531)	0.0133 (−0.0474, 0.0740)	0.0827 (−0.0151, 0.1805)	0.0135 (−0.0638, 0.0908)	0.0522 (0.0064, 0.0979)
WPR	−9.83 (−24.37, 4.72)	2.64 (−7.35, 12.64)	−10.74 (−23.91, 2.43)	1.28 (−7.75, 10.32)	−3.80 (−18.24, 10.63)	8.19 (−2.43, 18.81)	0.1109 (−0.0151, 0.2369)	−0.0241 (−0.0713, 0.0230)	0.0607 (−0.0316, 0.1531)	−0.0254 (−0.0720, 0.0212)	0.1586 (0.0491, 0.2682)	0.0082 (−0.0271, 0.0434)

ADRDs – Alzheimer's disease and related dementias, AFR – African Region, AMR – Region of the Americas, EMR – Eastern Mediterranean Region, EUR – European Region, QCI-v – quality of care index based on variational autoencoders, SDI – sociodemographic index, SEAR – South-East Asia Region, SII – Slope Index of Inequality, WPR – Western Pacific Region

## DISCUSSION

We observed an increase in the burden of ADRDs, with age-standardised DALY rates per 100 000 population rising from 1331.59 in 1990 to 1347.24 in 2021. This increase was more pronounced among males than females, particularly significant across almost all SDI regions (except for high SDI regions), and notable within South-East Asia and Africa. We likewise noted an improvement in the care quality of ADRDs, with the age-standardised QCI-v increasing from 51.56 in 1990 to 54.27 in 2021, and more so among females compared to males. There was a decline in care quality during 1990–2021 in low-middle and low SDI regions, as well as in Africa, Southeast Asia, the Eastern Mediterranean, and the Americas. When considering both disease burden and care quality, low-middle SDI and low SDI regions, as well as the African and South-East Asia regions, experienced increases in the ASRDs burden, but worsening care quality during 1990–2021. Furthermore, our findings reveal growing inequalities in both disease burden and care quality between sexes and across different regions. These findings underscore the necessity for targeted healthcare strategies and resource allocation to effectively address these disparities.

The observed increase in the burden of ADRDs highlights a significant public health challenge at a global level that possibly reflects improved diagnostics and increased awareness leading to more cases being identified. Lifestyle factors like obesity and sedentary behaviour, along with environmental influences, also contribute to this trend [10].

Previous studies have reported that the burden of ADRDs is unevenly distributed across age, sex, and geographic regions [4,5,20]. Specifically, ADRDs exhibit a notable age-related pattern, with prevalence increasing exponentially with advancing age [4,5]. This increase is primarily driven by neurodegenerative processes, such as the accumulation of amyloid plaques and tau tangles, which become more pronounced as individuals age [6]. Moreover, women experience a higher burden of ADRDs than men; while this disparity is partly attributable to women's longer life expectancies, it is also influenced by distinct biological mechanisms [21,22]. Key factors include hormonal changes (*e.g.* post-menopausal oestrogen decline), differences in brain structure and function, and genetic susceptibility (*e.g.* the impact of the APOE ε4 allele), all of which contribute to the increased risk and progression of ADRDs in women [23,24]. Our analysis showed that age-standardised DALY rates of ADRDs have increased more markedly among males than females over the last three decades. This could be attributed to the rising prevalence of vascular risk factors among men, such as diabetes, which is known to exacerbate cognitive decline [25]. Additionally, the slower improvement in care quality for men over time, as highlighted in our analysis, may also lead to worse health outcomes as these individuals age. Understanding these sex-specific trends and underlying factors is crucial for developing targeted interventions and improving care strategies tailored to the unique needs of both men and women affected by these conditions [26].

Previous studies have indicated a higher burden of ADRDs in low- and middle-income countries compared to high-income countries [3–5]. Social determinants of inequalities in care quality, such as socioeconomic disparities, educational attainment, healthcare accessibility, and racial and ethnic discrimination, may contribute significantly to the quality of ADRDs care [27,28]. Our analysis revealed significant disparities in the burden of ADRDs across different geographic regions from 1990 to 2021. For instance, the increase in ADRDs burden was particularly pronounced in regions with lower SDIs, as well as in South-East Asia and Africa, suggesting that socioeconomic factors and variations in regional health policies play critical roles in shaping disease burden [13]. The potential heterogeneity across high and low SDI countries is evident in disparities related to access to medical resources, healthcare infrastructure, and the availability of trained healthcare professionals and caregivers [10,13]. Lack of awareness, deficiency of financial resources, resources for diagnosing ADRDs, and treatment and related costs may pose challenges to survival and quality of life of ADRDs patients, particularly in low-income countries [13]. Competing health priorities, such as infectious diseases and malnutrition, coupled with under-resourced health systems, may further draw away focus from ADRDs in these regions [29,30].

We noted an increase in the care quality of ADRDs globally over the past three decades. This improvement is likely related to increased public awareness, enhanced diagnostic tools that facilitate earlier interventions, and better access to healthcare resources. Previous studies evaluating the care quality of ADRDs among individuals in care facilities within specific geographical areas underscore the need for standardised and equitable care [31–36]. For instance, a prior study involving individuals with dementia has demonstrated significant variations in both quality of life and care quality across eight European countries [35]. However, progress in care quality improvement was more pronounced among females than males, suggesting the need for sex-sensitive healthcare policies to ensure equitable care for all. Alarmingly, the decline noted here in care quality in low and low-middle SDI regions, particularly in Africa, Southeast Asia, the Eastern Mediterranean, and the Americas, underscores the vulnerability of healthcare systems in these areas to effectively manage complex conditions like ADRDs. This highlights the necessity for targeted interventions that strengthen healthcare systems and improve care quality, particularly in low-resource settings.

Taking the changes in the burden of ADRDS and the relate quality of care together, our analysis highlights a paradox in ADRDs epidemiology: high-income countries, while providing superior care, also experience a higher burden of these conditions. This may stem from enhanced diagnostic capabilities and increased awareness leading to more cases being identified and reported. In contrast, low-income countries face a lower burden, but deliver poorer care, primarily due to inadequate healthcare infrastructure, limited access to trained professionals, and insufficient resources for effective management. This disparity emphasises the need for comprehensive global health policies that both increase resources and improve the transfer of dementia care knowledge across countries [37]. Our findings further indicate that, in the study period, the burden of ADRDs was increasing in regions with lower SDI, particularly in Africa and Southeast Asia, yet the care quality was declining. This mismatch suggests that these regions are not well-prepared for the growing challenges posed by ADRD, highlighting the need for enhanced healthcare infrastructure and better training for healthcare providers [38]. Simultaneously, it will be necessary to advocate for international collaborations to facilitate the transfer of knowledge and technological innovations from high-income nations. Cost-effective models of care, adaptable for diverse settings, could also help with improving outcomes in low- and middle-income countries, but could also provide insights for high-income regions facing their own challenges in dementia care.

We noted that, while the absolute inequality in the burden of ADRDs has narrowed over time, the absolute inequality in care quality has widened. This indicates that the gap in disease burden between the highest and lowest SDI countries is decreasing, but disparities in care quality provided are increasing. As of 2021, significant positive absolute inequalities in ADRD burden persisted in Europe, suggesting that some higher SDI countries in this region still face substantial disease burdens despite healthcare advancements. Low-middle SDI regions, Africa, and the Eastern Mediterranean exhibited positive absolute inequalities in care quality, reflecting better services in higher SDI areas within those regions. Relative inequality has increased for both ADRD burden and care quality, suggesting that improvements are not equitably distributed. Regions with middle to high SDIs, along with the Eastern Mediterranean, show notable relative inequalities in ADRDs burden, indicating that even affluent regions can have marginalised nations with poorer outcomes. Additionally, significant relative inequality in care quality persists in middle, low-middle, and low SDI regions, as well as the Eastern Mediterranean. These disparities highlight the influence of socioeconomic status on access to care and underscore the need for comprehensive policies and targeted global health interventions. Enhancing healthcare access and quality in under-resourced areas, along with fostering international cooperation, is essential to address these disparities and ensure that high-quality care is accessible to all individuals, regardless of their geographic or economic conditions [39].

This study has several strengths. It is the first to assess the care quality of ADRDs globally. By simultaneously estimating both the burden and care quality, it identified regions with a high disease burden yet low care quality, as well as areas where an increasing burden coincides with declining care quality. This dual approach offers a novel perspective for policymakers to target resources effectively, aiming to reduce global inequalities in dementia care and improve outcomes for affected populations. Moreover, it is the first to explore cross-country inequalities in the burden and care quality of ADRDs, providing critical insights for policymakers to understand the disparities and challenges in managing ADRDs globally and regionally. By highlighting regions and trends where intervention is most needed, our findings can guide international efforts to optimise care and allocate resources where they are most likely to improve patient outcomes and reduce inequalities.

Our study also has some limitations. First, while the GBD estimates are the best available data for concurrent large-scale comparisons, caution is warranted when interpreting our findings, as the GBD data is inherently affected by factors such data availability, inaccuracies in the classification of non-fatal conditions, and a lack of primary data (particularly for morbidity). This is especially relevant in underdeveloped countries, where poor medical performance may lead to potential missed diagnosis, and inadequate documentation and record-keeping might result in documentation loss being more common than in developed countries, leading to an underestimation of the burden of ADRDs [40,41]. To mitigate these biases, it is crucial to improve the detection of ADRDs and increase health check-ups for the elderly, necessitating a heightened societal focus on the burden of ADRDs. Second, our study does not explore ethnic and racial disparities, as we lacked data to do so. Additionally, some subtypes of ADRDs were not evaluated separately in the GBD, which limited our capacity for more extensive exploration.

## CONCLUSIONS

Our analysis showed an increase in both the burden and care quality of ADRDs from 1990 to 2021, accompanied by significant disparities between countries. Higher SDI regions, despite better care quality, face a greater burden of ADRDs, likely due to enhanced diagnostic capabilities. In contrast, lower SDI regions, particularly those in Africa and Southeast Asia, experience both increases in the burden of ADRDs and a decline in the related quality of care. Further studies should focus on the determinants of the burden and care quality of ADRDs or attempt to establish effective prevention, management, and treatment programmes, particularly in countries with lower levels of sociodemographic development.

## Additional material


Online Supplementary Document
