# Supplementary material for: Global burden, quality of care, and cross-country inequalities in Alzheimer’s disease and other dementias from 1990 to 2021
Source: J Glob Health. 2025 Oct 3;15:04269. doi: 10.7189/jogh.15.04269 (PMC12491904; doi:10.7189/jogh.15.04269)
Supplement: Online Supplementary Document [file jogh-15-04269-s001.pdf]

Supplement to: Zhu Y, Cheng S, Luo Z, Shen J, Cao J, Yao L, Zhou J, Shan S, Song P, Yuan C. Global burden, quality of care, and cross-country inequalities in Alzheimer's disease and other dementias from 1990 to 2021. J Glob Health. 2025;15:04269.

Supplementary Appendix

Global burden, quality of care, and cross-country inequalities in Alzheimer's disease and other dementias from 1990 to 2021

This supplementary material has been provided by the authors to give readers additional information about their work.

Table of Contents

Appendix 1. Guideline *4*

Table S1. Adherence to JoGH's GRABDROP guidelines items. 4

Appendix 2. eMethods *6*

1 Overview of the GBD 2021 6

2 Estimation of the non-fatal burden of Alzheimer's disease and other dementias in GBD 2021 7

2.1 Flowchart 7

2.2 Case definition 7

2.3 Input data 8

2.4 Modelling strategy 12

3 Estimation of the fatal burden of Alzheimer's disease and other dementias in GBD 2021 15

3.1 Flowchart 15

3.2 Input data 15

3.3 Modelling strategy 16

References 19

Appendix 3. Supplementary results *21*

Supplementary tables 21

Table S2. The age-standardized DALY rates of ADRDs and its temporal change by sex and nation, 1990-2021 21

Table S3. The age-standardized QCI-v of ADRDs and its temporal change by sex and nation, 1990-2021 32

Table S4. The year trends of slope index of inequality for burden of ADRDs by sex, SDI quantiles and WHO regions, from 1990 to 2021 43

Table S5. The year trends of concentration index for burden of ADRDs by sex, SDI quantiles and WHO regions, from 1990 to 2021 49

Table S6. The year trends of slope index of inequality for care quality of ADRDs by sex, SDI quantiles and WHO regions, from 1990 to 2021 55

Table S7. The year trends of concentration index for care quality of ADRDs by sex, SDI quantiles and WHO regions, from 1990 to 2021 61

Supplementary figures 67

Figure S1. Age-standardized DALY rates of ADRDs in females across all countries in 2021 (A) and AAPC from 1990 to 2021 (B) 67

Figure S2. Age-standardized DALY rates of ADRDs in males across all countries in 2021 (A) and AAPC from 1990 to 2021 (B) 68

Figure S3. Age-standardized QCI-v of ADRDs in females across all countries in 2021 (A) and AAPC from 1990 to 2021 (B) 69

Figure S4. Age-standardized QCI-v of ADRDs in males across all countries in 2021 (A) and AAPC from 1990 to 2021 (B) 70

Figure S5. Absolute and relative inequalities in the age-standardized DALY rates of ADRDs

in females worldwide, 1990 and 2021 71
Figure S6. Absolute and relative inequalities in the age-standardized DALY rates of ADRDs in males worldwide, 1990 and 2021 72
Figure S7. The year trends of slope index of inequality for burden of ADRDs by sex, SDI quantiles and WHO regions, from 1990 to 2021 73
Figure S8. The year trends of concentration index for burden of ADRDs by sex, SDI quantiles and WHO regions, from 1990 to 2021 74
Figure S9. Absolute and relative inequalities in the age-standardized QCI-v of ADRDs in females worldwide, 1990 and 2021 75
Figure S10. Absolute and relative inequalities in the age-standardized QCI-v of ADRDs in males worldwide, 1990 and 2021 76
Figure S11. The year trends of slope index of inequality for care quality of ADRDs by sex, SDI quantiles and WHO regions, from 1990 to 2021 77
Figure S12. The year trends of concentration index for care quality of ADRDs by sex, SDI quantiles and WHO regions, from 1990 to 2021 78

Appendix 1. Guideline

Table S1. Adherence to JoGH's GRABDROP guidelines items.

JoGH guideline items
1. Please list all papers published by each co-author in previous 3 years that were based on secondary analysis of a big data repository
1) Shiyi Shan, Jiayao Ying, Jiali Zhou, Zeyu Luo, Jing Wu, Guangdian Shen, Denan Jiang, Lingzi Yao, Liying Zhou, Peige Song* (2025). Cross-country inequalities in the epidemiology of chronic kidney disease due to type 2 diabetes mellitus, 1990-2021: findings from the Global Burden of Disease Study 2021. Diabetology & Metabolic Syndrome, 17 (1), 1-11.<br>2) Yajie Zhu, Weidi Sun, Yuan Song, Dexter Canoy, Peige Song*, Kazem Rahimi (2025). Associations of pain phenotypes and pain relief medications with stroke and its subtypes: A prospective cohort study. Scientific Reports, 15:21640.<br>3) Ke Tang, Longzhu Zhu, Shiyi Shan, Zeyu Luo, Jiali Zhou, Jiayao Ying, Jing Wu, Guangdian Shen, Peige Song* (2025). Global, regional and national trends in the epidemiology of rheumatoid arthritis from 1990 to 2021: an age-period-cohort effect analysis of the Global Burden of Disease Study 2021. RMD Open, 11: e005383.<br>4) Shanshan Tang, Jin Cao, Denan Jiang, Longzhu Zhu, Zeyu Luo, Shiyi Shan, Jiali Zhou, Jiayao Ying, Jing Wu, Peige Song*, Wei Li* (2025). Global, regional and national temporal trends in incidence and mortality of non-Hodgkin lymphoma from 1992 to 2021: an age-period-cohort analysis. Annals of Hematology.<br>5) Zeyu Luo, Denan Jiang, Shiyi Shan, Jiali Zhou, Weidi Sun, Jing Wu, Jiayao Ying, Liying Zhou, Yajie Zhu, Peige Song*, Kazem Rahimi (2025). Cross-country inequalities in disease burden and quality of care of stroke, 1990-2021: a systematic analysis of the Global Burden of Disease Study 2021. European Journal of Neurology, 32 (2): e70050.<br>6) Shiyi Shan, Zeyu Luo, Lingzi Yao, Jiali Zhou, Jing Wu, Denan Jiang, Jiayao Ying, Jin Cao, Liying Zhou, Sheyu Li, Peige Song* (2024). Cross-country inequalities in disease burden and care quality of chronic kidney disease due to type 2 diabetes mellitus, 1990–2021: Findings from the global burden of disease study 2021. Diabetes, Obesity and Metabolism, 26:5950–5959.<br>7) Lingzi Yao, Jin Cao, Siqing Cheng, Shiyi Shan, Denan Jiang, Zeyu Luo, Shuting Li, Leying Hou, Xue Li, Peige Song* (2024). Inequalities in disease burden and care quality of chronic obstructive pulmonary disease, 1990-2021: Findings from the Global Burden of Disease Study 2021. Journal of Global Health,14:04213.<br>8) Zeyu Luo, Shiyi Shan, Jin Cao, Jiali Zhou, Liying Zhou, Denan Jiang, Lingzi Yao, Jing Wu, Jiayao Ying, Yajie Zhu, Peige Song*, Kazem Rahimi (2024). Temporal trends in cross-country inequalities of stroke and subtypes burden from 1990 to 2021: a secondary analysis of the global burden of disease study 2021. eClinicalMedicine, 76:102829.<br>9) Siqing Cheng, Jin Cao, Leying Hou, Shuting Li, Weidi Sun, Shiyi Shan, Jianhui Zhao, Lingzi Yao, Xue Li, Bin He, Peige Song* (2024). Temporal trends and projections in the global burden of neck pain: findings from the Global Burden of Disease Study 2019. Pain, 165(6):10-1097.
2. Please explain the key elements of your study design and the use of the available datasets that make your study an original scientific contribution
This study presents a comprehensive secondary analysis of GBD 2021 data, examining trends in Alzheimer's disease and related dementias (ADRDs) across 204 countries from 1990–2021. It is the first global study to jointly assess disease burden and care quality of ADRDs. We developed a novel Quality of Care Index using a variational autoencoder (QCI-v), a machine learning model that integrates four secondary indicators (e.g., mortality-to-

incidence ratio) and, for the first time, provides 95% confidence intervals for care quality estimates using bootstrap sampling. The study also applies standard equity metrics (SII and concentration index) to assess cross-country inequalities, identifying regions with rising burden and declining care quality—an urgent global health concern.
3. Please list all publications that addressed similar research questions in the same dataset and indicate where you cited them in your paper
Nichols, Emma, and Theo Vos. "The estimation of the global prevalence of dementia from 1990-2019 and forecasted prevalence through 2050: an analysis for the Global Burden of Disease (GBD) study 2019." *Alzheimer's & Dementia* 17 (2021): e051496.<br>Nichols, Emma, et al. "Estimation of the global prevalence of dementia in 2019 and forecasted prevalence in 2050: an analysis for the Global Burden of Disease Study 2019." *The Lancet Public Health* 7.2 (2022): e105-e125.<br>Xu, Changqing, et al. "Epidemiological and sociodemographic transitions in the global burden and risk factors for Alzheimer's disease and other dementias: a secondary analysis of GBD 2021." *International journal for equity in health* 24.1 (2025): 1-15.
4. Please explain how you addressed multiple testing through an appropriately rigorous statistical threshold and indicate this in the methods section
Multiple hypothesis testing was addressed by applying Bonferroni correction for post-hoc comparisons (see Methods, lines 172–176). All statistical tests were two-sided, and a significance threshold of $P < 0.05$ was applied after adjustment.
5. Please declare to what extent have AI chatbots been used in developing your paper and to which parts of the paper did they contribute
AI chatbots were not used in the writing or analysis of this manuscript. All content was developed by the authors using manual literature review, statistical programming (Python 3.9.11, R 4.3.3), and expert interpretation of results.

Appendix 2. eMethods

1 Overview of the GBD 2021

The Global Burden of Diseases, Injuries, and Risk Factors Study (GBD) 2021 is a comprehensive approach to global descriptive epidemiology [1-3]. It is a systematic, scientific effort to quantify the comparative magnitude of health loss due to diseases, injuries, and risk factors by age, sex, and geography for specific points in time. Institute for Health Metrics and Evaluation (IHME) serves as the coordinating center for the GBD and affiliated projects. Commonly used epidemiological quantity of interest in GBD includes incidence, prevalence, mortality, years lived with disability (YLDs), years of life lost (YLLs), and disability-adjusted life-years (DALYs). GBD 2021 estimates numbers and rates of incidence, prevalence, YLDs, and DALYs for the years 1990–2021, deaths and YLLs for 1980–2021. This was done for 288 causes of death by age-sex-location-year for 25 age groups from birth to 95 years and older; for males, females, and both sexes combined; in 204 countries and territories grouped into 21 regions and seven super-regions.

For GBD 2021, the causes of death cause list has increased to 288 causes, from the 286 causes in GBD 2019. The non-fatal cause list has expanded from 364 causes in GBD 2019 to 365 causes in GBD 2021. The total number of fatal and non-fatal causes combined for GBD 2021 is 371. As in GBD 2019, we made no estimates for YLDs for just five causes, either because no disability is possible (as is the case with sudden infant death syndrome); because disability may occur rarely but at levels too low for accurate estimation given the data (as for aortic aneurysm); or because the disability is captured by the complicating causes that led to that cause of death (as for indirect maternal deaths, late maternal deaths, and maternal deaths aggravated by HIV/AIDS). GBD 2021 includes 19 189 new data sources for DALYs, 12 new causes, and other important methodological updates. Notably, GBD 2021 disaggregated the under-5 age group, providing more detailed age-based estimates of burden for young children. GBD 2021 reports ten non-COVID-19-related causes for the first time: pulmonary arterial hypertension, hepatoblastoma, Burkitt lymphoma, other non-Hodgkin lymphoma, eye cancer (including retinoblastoma and other eye cancers as distinct causes), soft tissue and other extraosseous sarcomas, malignant neoplasm of bone and articular cartilage, neuroblastoma, and other peripheral nervous cell tumours. Another important advancement of GBD 2021 is the addition of the impact of the COVID-19 pandemic on disease burden. GBD 2021 includes the estimation of burden for COVID-19, post-COVID-19 condition (also known as long COVID), other pandemic-related outcomes, and the effect of COVID-19 on other selected diseases and injuries.

In this manuscript, we estimated the burden of ADRDs for age groups between 40 and 95+ years, for males and females, between 1990 and 2021, for 204 countries and territories. All estimates can be accessed via GBD Compare (https://vizhub.healthdata.org/gbd-compare/), GBD Results Tool (https://vizhub.healthdata.org/gbd-results/), and model-specific visualisations (Epi Visualization https://vizhub.healthdata.org/epi/ and CoD Visualization

https://vizhub.healthdata.org/cod/).

2 Estimation of the non-fatal burden of Alzheimer's disease and other dementias in GBD 2021

2.1 Flowchart

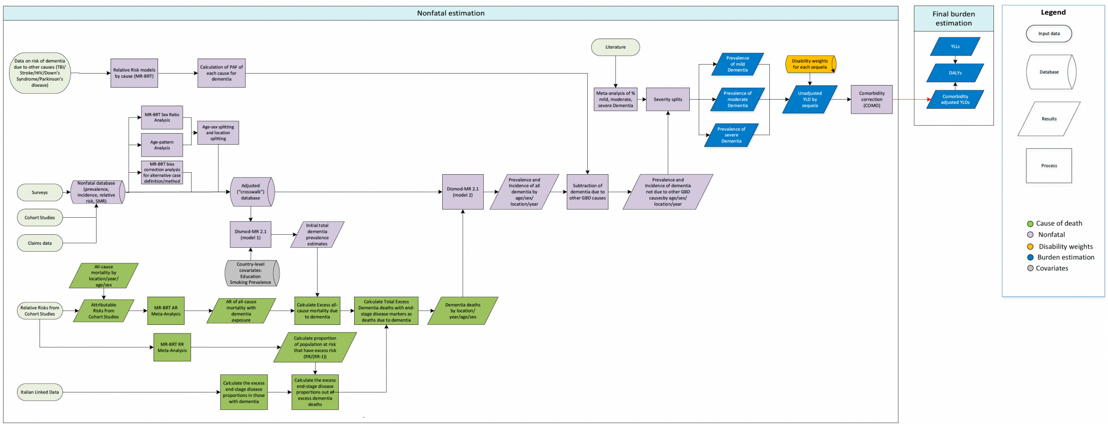

2.2 Case definition

Dementia is a progressive, degenerative, and chronic neurological disorder typified by memory impairment and other neurological dysfunctions. For the purposes of GBD 2021, we used the Diagnostic and Statistical Manual of Mental Disorders III, IV or V, or ICD case definitions as the reference. The DSMIV definition is:

- Multiple cognitive deficits manifested by both memory impairment and one of the following: aphasia, apraxia, agnosia, disturbance in executive functioning
- Must cause significant impairment in occupational functioning and represent a significant decline
- Course is characterised by gradual onset and continuing cognitive decline
- Cognitive deficits are not due to other psychiatric conditions
- Deficits do not occur exclusively during the course of a delirium

A wide array of diagnostic and screening instruments exists, including Clinical Dementia Rating scale (CDR), Mini-Mental State Examination (MMSE), and the Geriatric Mental State (GMS). For severity rating purposes we use the CDR as the reference. The relevant ICD-10 codes for dementia are F00, F01, F02, F03, G30, and G31. The ICD-9 codes are 290, 291.2, 291.8, 294 and 331.

Alternative case definitions accepted for inclusion included (1) diagnosis based on the result of an algorithm, (2) diagnosis based on general practitioner data, (3) diagnosis based on clinical records, (4) diagnosis using 10/66 algorithm, and (5) diagnosis with NIA-AA (National Institute of Ageing – Alzheimer's Association) criteria instead of ICD or DSM.

Unlike most causes in the Global Burden of Disease project, dementia mortality and morbidity estimates are modelled jointly. This is because of marked discrepancies between prevalence

data and cause of death data. Specifically, prevalence data suggest little to no variation over time (e.g., 1990–2020), whereas age-standardised mortality rates in vital registrations in high-income countries have increased multiple times over this same period. Additionally, prevalence variation between countries is much smaller than the variation in death rates assigned to dementia in vital registration. We attribute these discrepancies to changing coding practices rather than epidemiological change.

Because of this joint procedure, descriptions of the mortality estimation process are included where relevant.

2.3 Input data

Model inputs

To inform our estimates of burden due to dementia, we use mortality data from relative risk studies and linked hospital to mortality data, as well as prevalence and incidence data from surveys and administrative data such as claims sources. We included incidence data in dementia modelling for the first time in GBD 2021. We excluded all U.S. Marketscan claims data in GBD 2021 because of the paucity of data available to inform a reliable adjustment, and because the large number of these data meant claims had an unduly large influence on the global fit of the dementia model.

Item Response Theory for prevalence prediction

The prevalence models for dementia are data sparse, and there are not many surveys done in low-income settings. However, there are a larger body of surveys that collect data on cognitive tests and functional limitations, which are the two main components of a DSM or ICD diagnosis. Predictions of dementia prevalence using information from these questions would allow for expanded data coverage and additional information in locations where there are currently no data guiding estimates.

Generating these predictions requires calibrating a model to samples that have information about both functional limitations, cognition, and adjudicated dementia diagnoses. However, making comparisons across surveys can be difficult, as each survey asks a different set of questions about cognition and limitations, although there is some overlap. This overlap allows for the use of item response theory methods for the harmonisation of these scales. Once the scales are harmonised, the subsamples can be utilised to create a model for the prediction of prevalence.

In GBD 2019, data from the ADAMS and HRS surveys were extracted and used for item response theory modelling to estimate prevalence. HRS is a nationally representative survey in the USA, which has data on cognition and functional limitations. ADAMS is a subsample of HRS that includes much more detailed neuropsychological testing and adjudicated dementia diagnoses. ADAMS includes almost all questions in HRS plus additional questions as well.

Severity splits

Methods to determine severity splits for dementia were redesigned for GBD 2019 onwards. A new systematic review was conducted to collect information on the proportion of individuals in each dementia severity class out of the population of all individuals with dementia. There are a variety of commonly-used methods for severity rating; we took the Clinical Dementia Rating (CDR) scale as our reference definition for severity classification, along with a doctor-given diagnosis according to DSM III, IV, V or ICD case definitions as our reference definition for dementia.

However, as a neurodegenerative disorder with a wide range of categories in which symptoms manifest, there are an abundance of classification tools which discern between severity levels along different criteria. We accepted severities classified by:

- Clinical dementia rating sum-of-boxes (CSR-SB)
- Blessed test of information, memory, and concentration (BIMC)
- Global deterioration scale (GDS)
- Geriatric Mental State Examination (GMS)
- CAMDEX
- DSM-III-R
- Karasawa's

We excluded any studies which classified dementia severity according to scales that only evaluated cognitive function and memory, excluding activities of daily living (ADLs). The most prominent such scale is MMSE (Mini-Mental State Exam).

The most recent systematic review covered literature published through August 2017 and the following search string was used:

((dementia[MeSH Terms] OR dementia[Title] OR Alzheimer disease[Title]) AND (severity[Title/Abstract] OR CDR[Title/Abstract] OR Clinical Dementia Rating Scale[Title/Abstract]) AND (Severity of illness index[MeSH] OR diagnosis[sh] OR Cross-Sectional Studies[MeSH])) AND ("1950/01/01"[Date - Publication] : "2100/02/25"[Date - Publication]) NOT (animals[MeSH] NOT humans[MeSH]))

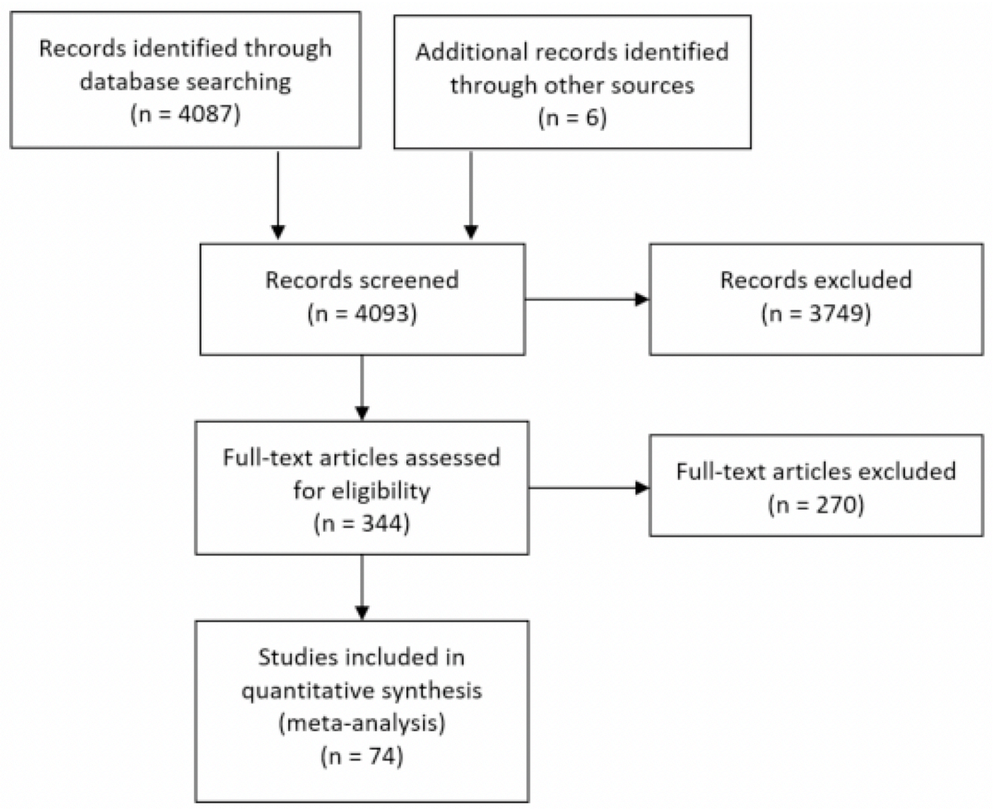

eMethods Figure 2.1. Prisma diagram of dementia severity split systematic review

This yielded 4087 total hits, of which 338 passed initial title/abstract screening. After full-text screening, 68 sources met screening criteria and were extracted, along with one source identified through the bibliographies of other sources, and five additional sources used in GBD 2017 for other purposes. A total of 74 sources were extracted and informed the severity split, as compared to the 11 sources used in previous GBD rounds.

The severity split analysis was conducted using the MR-BRT tool (meta-regression—Bayesian, regularised, trimmed; additional information can be found in appendix 1, section 2.5 of the cited paper)[2].

We multiplied estimations of prevalence (country-year-sex-age-specific) by the fractions of mild, moderate, and severe dementia and estimated 95% uncertainty intervals at the 1,000-draw level. The severity distributions over age for each sex are visualised below, followed by a table describing each severity.

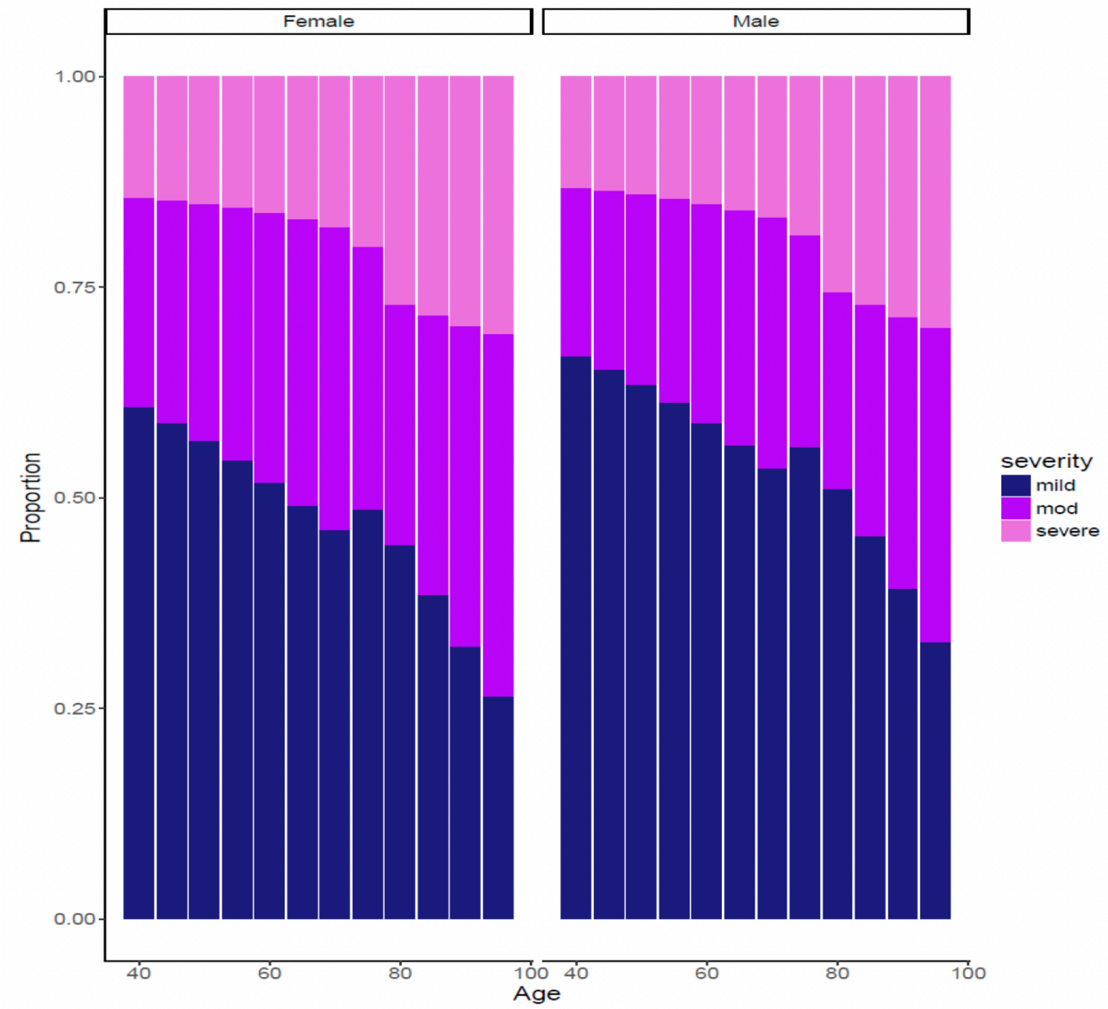

eMethods Figure 2.2. Severity ratios for each 5-year age bin, by sex

eMethods Table 2.1. Dementia severity levels

Severity level	Lay description
Mild	The person has some trouble remembering recent events and finds it hard to concentrate and make decisions and plans. They may have slight to moderate difficulty engaging in community affairs, complicated hobbies, and intellectual interests.
Moderate	The person retains highly learned material, but has severe memory problems, is disoriented with respect to time and sometimes place. They are severely impaired in their ability to handle problems and make social judgements. They require assistance with daily activities, and only retain simple chores and hobbies.
Severe	The person has complete memory loss, no longer recognises close family members, and requires help with all daily activities, including personal care.

Relative risk due to other causes

While the DSM definition excludes dementia cases where the syndrome is caused by other psychiatric disorders, it does not exclude dementia cases caused by other diseases not included

in DSM. This includes stroke, Parkinson's disease, Down's syndrome, and traumatic brain injury (TBI), which are found elsewhere in the GBD cause list. To prevent double counting of prevalent cases, both under dementia and each of these other causes, we adjusted our dementia prevalence to exclude cases caused by these other conditions. To do so, in GBD 2019 we used data from the Aging, Demographics and Memory study (ADAMS), to estimate the relative risk of getting dementia for each condition included in the ADAMS dataset (stroke, Parkinson's disease, TBI). We then conducted more extensive systematic reviews on all five of these conditions to model each separately. Relative risk models were run using MR-BRT, and population attributable fractions (PAF) for each condition were calculated with the following equation, where exposure is defined as the prevalence of condition:

$$PAF = \frac{exposure * (RR - 1)}{[exposure * (RR - 1)] + 1}$$

Finally, attributable burden was calculated as the PAF multiplied by total burden (ie, dementia incidence/prevalence)

eMethods Table 2.2. Summary of each systematic review for relative risk due to other causes

	Stroke	Parkinson's disease	Down's syndrome	TBI
**Data Type**	Relative Risks	Proportions and Relative Risks	Proportions	Relative Risks
**Review Hits**	504	1475	355	
**Accepted During Title/Abstract Screening**	79	135	102	
**Accepted During Full Text**	35 (33 from systematic review and two from PubMed search)	56	26	45

eMethods Table 2.3. Total source count used in GBD 2021 modelling

Measure	Total sources	Countries with data
All measures	513	55
Prevalence	245	48
Incidence	81	24
Other	212	37

2.4 Modelling strategy

First, prevalence data were sex split, crosswalked, and age split. Studies with age and sex detail separately were split into age- and sex-specific datapoints. Data specified as "both" sex data were split into male- and female-specific datapoints using MR-BRT to get a model ratio of

female/male prevalence and then using the following equations:

Male prevalence:

$$prev_{male} = prev_{male} * \frac{pop_{both}}{(pop_{male} + ratio * pop_{female})}$$

Female prevalence:

$$prev_{female} = ratio * prev_{male}$$

We also split datapoints where the age range was greater than 25 years using the global age pattern.

Dementia studies are heterogeneous. Even with a smaller number of definitions (DSM/ICD), there are a large number of different ways to diagnose dementia. For example, out of 272 sources used in a previous round, GBD 2017, there were 263 different methods of diagnosing dementia (overlap was among those who used 10/66 protocol or AGECAT algorithm). Most use a two-step procedure, where you screen using a cognitive test and then only fully evaluate those that fall below a certain pre-defined threshold. We controlled for methods differences by crosswalking alternative case definitions to reference. Study covariates are based on broad categories determined after going through the diagnostic heterogeneity and there are some added for specific criteria that we know are biased. The same study-level covariates were used in GBD 2021 as in GBD 2019. Crosswalking was carried out using a logit difference network meta-regression analysis.

eMethods Table 2.4. MR-BRT Crosswalk Adjustment Factors for Dementia (Network Analysis)

Data input	Reference or alternative case definition	Gamma	Beta Coefficient, Logit (95% UI)*	Adjustment factor**
DSM or ICD case definition	Ref	0.34	---	---
Clinical records diagnosis criteria	Alt		-0.05 (-0.72 – _0.61)	0.95
Algorithm diagnosis criteria (AGECAT)	Alt		0.08 (-0.59 – _0.74)	1.08
NIA-AA diagnosis criteria	Alt		0.51 (-0.16 – _1.17)	1.67
10/66 algorithm diagnosis criteria	Alt		0.97 (0.30 – _1.64)	2.64
GP records used for diagnosis	Alt		-1.21 (-1.88 – _-0.54)	0.30

**MR-BRT crosswalk adjustments can be interpreted as the factor the alternative case definition is adjusted by to reflect what it would have been had it been measured using the reference case definition. If the log/logit beta coefficient is negative, then the alternative is adjusted up to the reference. If the log/logit beta coefficient is positive, then the alternative is adjusted down to the reference.*

***The adjustment factor column is the exponentiated beta coefficient. For log beta coefficients, this is the relative rate between the two case definitions. For logit beta coefficients, this is the relative odds between the two case definitions.*

Two country-level covariates were included in the initial DisMod-MR 2.1 model 1 (disease model—Bayesian meta-regression, details on this method can be found in appendix 1, section 4.5 of the citation). Age-standardised education was used as a proxy for general brain health/use that may be protective of dementia – specifically Alzheimer's disease. Smoking prevalence (age-standardised, both sexes) was also used as a covariate to guide estimates, as the literature has shown a positive relationship between smoking and dementia.

Note that two DisMod-MR models were run with prevalence and incidence inputs – the first used prevalence, within-condition mortality rate, and relative risk data before mortality adjustments (DisMod-MR Model 1 in flowchart). The second used dementia prevalence and incidence data and cause-specific mortality rate inputs from final mortality modelling (DisMod-MR Model 2 in flowchart) which accounts for all dementia regardless of cause (this is the dementia impairment envelope). The tables below summarise country-level covariates used in each of these DisMod-MR model.

eMethods Table 2.5. Summary of covariates used in the Parkinson's Disease DisMod-MR meta-regression model (adjusted prevalence, Model 1)

Covariate	Type	Parameter	Exponentiated beta (95% Uncertainty Interval)
Smoking prevalence (age-standardised)	Prevalence	1.00 (0.015 to 1.94)	2.72 (1.01 to 6.96)
Education age-standardised	Prevalence	-0.083 (-0.083 to -0.083)	0.92 (0.92 to 0.92)

eMethods Table 2.6. Summary of covariates used in the Parkinson's Disease DisMod-MR meta-regression model (unadjusted prevalence, Model 2)

Covariate	Type	Parameter	Exponentiated beta (95% Uncertainty Interval)
Smoking (age-standardised)	Incidence	1.35 (0.76 to 1.90)	3.86 (2.13 to 6.71)
Education age-standardised	Incidence	-0.012 (-0.037 to -0.0004)	0.99 (0.96 to 1.00)
Education age-standardised	Prevalence	-0.083 (-0.083 to -0.083)	0.92 (0.92 to 0.92)

As mentioned previously, the estimation of morbidity due to dementia occurs in conjunction with the mortality estimation. Additional details on this process can be found in the COD capstone appendix.

We pulled the cause-specific mortality results from final fatal estimates into a final DisMod-

MR model (Model 2). To prevent double counting of prevalent cases, both under dementia and under other causes that can lead to dementia, we adjusted our dementia prevalence to exclude cases caused by these other conditions, which include stroke, Parkinson's disease, traumatic brain injury, and Down's syndrome. To do so, we used data from the Aging, Demographics and Memory study (ADAMS) and new systematic reviews, to estimate the relative risk of getting dementia for each condition included in the ADAMS dataset (stroke, Parkinson's disease, TBI). We first fit logistic regression models predicting the outcome of dementia given each exposure, with an additional covariate on age.

We then used these models to predict the probability of dementia given each exposure at various ages and divided the probability of having dementia by the probability of not having dementia at each age to calculate relative risks. After calculating age specific relative risks, we used these data and estimates of dementia prevalence from our DisMod-MR 2.1 model to calculate the population attributable fractions (PAFs) for each cause and age using the formula:

$$PAF = \frac{exposure * (RR - 1)}{[exposure * (RR - 1)] + 1}$$

Finally, we multiplied the PAF by the total prevalence to get the amount of dementia prevalence that can be attributed to each cause and subtracted this from the total prevalence to get the prevalence of dementia that is not due to other GBD causes.

3 Estimation of the fatal burden of Alzheimer's disease and other dementias in GBD 2021

3.1 Flowchart

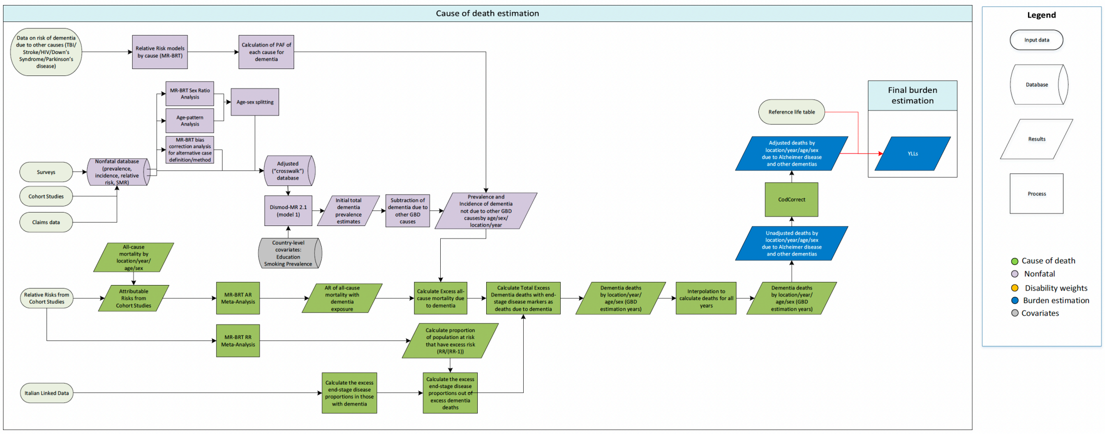

3.2 Input data

In GBD 2019 onward, fatal modelling was redesigned to remove reliance on vital registration data (described in more detail in the "Modelling strategy" section). Instead, two new source types were extracted:

(1) Literature on the relative risk of all-cause mortality given the exposure of dementia. Relative risk sources were identified through a systematic review using search terms2 in PubMed. This

yielded 4470 total hits, of which 34 studies were marked for extraction. Overall, the data were heterogeneous and varied in the exposure category measured (all dementia, Alzheimer's disease, cognitive impairment) and in the different factors controlled for in analyses.

(2) Linked vital registration and hospitalisation data. We used mortality records linked to inpatient records, covering all deaths from 2003 to 2017 in the Emilia-Romagna region of Italy.

eMethods Table 3.1. Results of systematic review on all-cause excess mortality with dementia

**N**		**60**
**Region name (%)**	East Asia	4 (6.7)
	Eastern sub-Saharan Africa	2 (3.3)
	High-income Asia Pacific	4 (6.7)
	High-income North America	22 (36.7)
	North Africa and Middle East	1 (1.7)
	Tropical Latin America	1 (1.7)
	Western Europe	26 (43.3)
**Exposure (%)**	Alzheimer's disease	11 (18.3)
	Cognitive impairment	10 (16.7)
	Other dementia	35 (58.3)
	Vascular dementia	4 (6.7)
**Conducted in clinical setting (%)**	Clinical setting	10 (16.7)
	Population representative	50 (83.3)
**Controlled for education (%)**	Controlled	32 (53.3)
	No control	28 (46.7)
**Controlled for basic CVD info (%)**	Controlled	33 (55.0)
	No control	27 (45.0)
**Extensive CVD control (%)**	Controlled	15 (25.0)
	No control	45 (75.0)
**Controlled for smoking and alcohol (%)**	Controlled	11 (18.3)
	No control	49 (81.7)
**Controlled for factors in causal pathway (%)**	Controlled	13 (21.7)
	No control	47 (78.3)

3.3 Modelling strategy

Overview

Dementia mortality rates have increased more than five-fold since 1980 in high-quality vital registration systems such as in the USA and Scandinavia. We have not seen an equivalent increase in prevalence and incidence data sources. If at all, there has been a modest decline in incidence and prevalence of dementia in studies in the UK and the USA[4]. Also, the greater than 20-fold variation in mortality rates of dementia between countries is much greater than the four-fold difference in prevalence and incidence between countries. As it is unlikely that case fatality from dementia has dramatically increased over the time period and that it would differ by a

very large margin between countries, the hypothesis is that certifying and coding practices have changed over time and at a different pace between countries. To avoid spurious large trends over time in the fatal component of the burden of dementia, we decided for GBD 2013 to make dementia mortality rates consistent with the most recent rates relative to prevalence of countries that are most likely to certify or code dementia as an underlying cause of death. This approach was applied again for GBD 2017 with some modifications. For GBD 2019 onward, the fatal modelling process was redesigned to avoid the need for using estimates only from the highest dementia mortality locations. This was accomplished with an attributable risk model based on a systematic review of cohort studies and relative risk data, and end-stage disease proportions from linked hospital and death records. The modelling process is described below.

<u>Modelling steps</u>

1) Relative risk data

First, using relative risk data extracted from studies identified by systematic review, we calculated attributable risk and the GBD estimate of all-cause mortality rate for a given study location and time, using the following formula:

$$Attributable\ Risk = (Relative\ Risk - 1) * All - cause\ Mortality$$

We then conducted a meta-analysis on the attributable risk data, using covariates for age, sex, exposure category (all dementia, Alzheimer's disease, cognitive impairment), whether the study was conducted in a clinical sample, and categories indicating different types of variables that were controlled for in the component studies (educational attainment, cardiovascular disease comorbidities, smoking and alcohol consumption, and daily activities or residence in a nursing home). Relative risks were estimated using a second Bayesian bias-reduction meta-regression model and the same studies identified through systematic review. Regression results for relative risk and attributable risk analyses are displayed below.

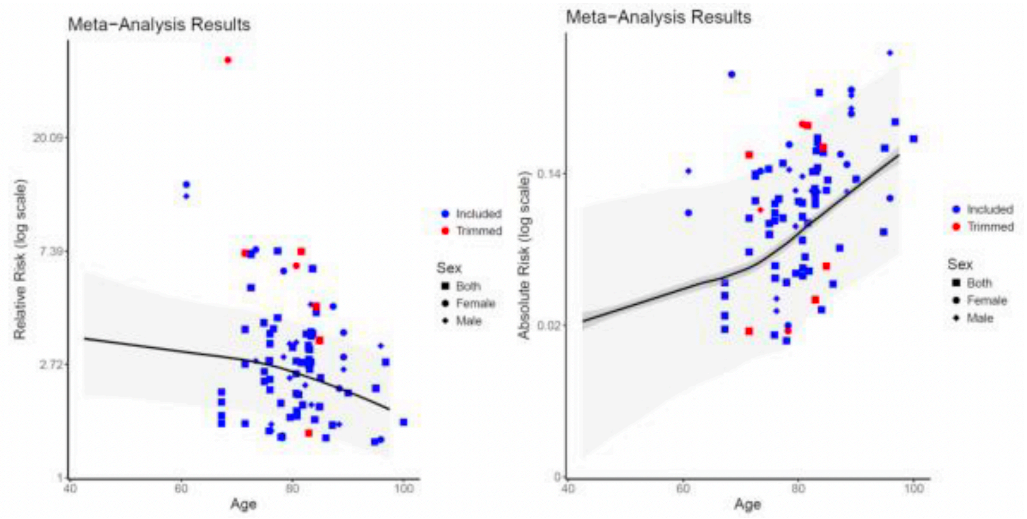

eMethods Figure 3.1. Regression results for relative risk and attributable risk analyses

Meta-regression results were used to calculate the total number of excess deaths due to

dementia as the product of our prevalence estimates (post-adjustment for dementia caused by other GBD diseases) and our estimates of attributable risk. See the non-fatal write-up on dementia for details on prevalence calculations.

2) Linked data

The excess deaths calculated through the multiplication of attributable risk and prevalence represent the total number of excess deaths due to having dementia, which likely includes deaths due to other conditions, such as cardiovascular diseases, that are more common in those with dementia as compared to the general population due to common underlying risk factors such as blood pressure, smoking, and lower educational attainment. In order to subset this total number of excess dementia deaths to calculate the number of deaths that were caused by dementia, we completed an analysis of linked clinical and mortality data. We used mortality records linked to inpatient records, covering all deaths from 2003 to 2017 in the Emilia-Romagna region of Italy. Using these data, we looked for markers of severe, end-stage disease in the clinical records up to one year before death.

To select these markers, for each ICD code that appeared in the data we calculated the difference in the proportion of individuals who died with dementia and had a record of each code in the year before death and the proportion of individuals who died without dementia and had a record of the same code in the year before death. We reviewed the 150 codes with the highest difference and selected codes that indicated end-stage disease, excluding codes for conditions such as cardiovascular disease. Codes for decubitus ulcer, malnutrition, sepsis, pneumonia, urinary tract infections, falling from bed, senility, dehydration, sodium imbalance, muscular wasting, bronchitis, dysphagia, hip fracture, and bedridden status were used as indicators of severe disease.

In order to determine the proportion of excess deaths that were caused by dementia, we calculated the proportion of dementia deaths that had clinical markers of end-stage disease in the year before death, above and beyond the occurrence of end-stage disease markers in those who died without dementia. The subtraction of the proportions with end-stage disease markers in those without dementia from the proportions in those with dementia represents the proportion of individuals who are assumed to have died with severe, end-stage dementia out of total deaths in those with dementia.

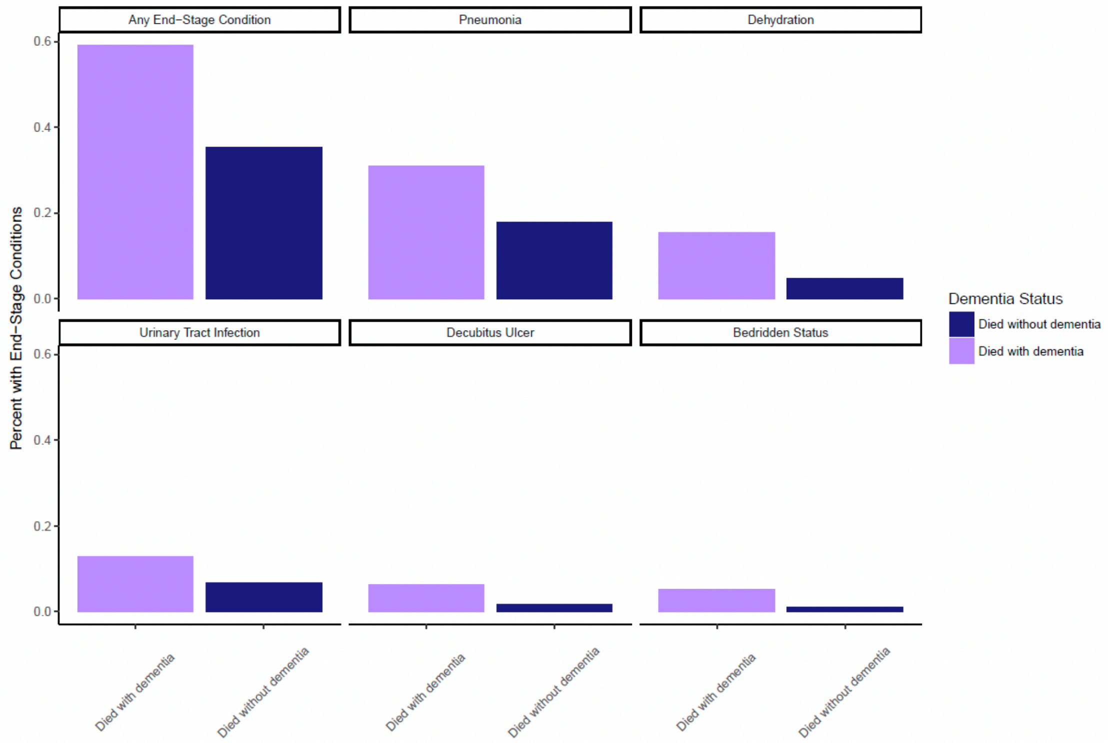

eMethods Figure 3.2. Percent of individuals who died with and without dementia with end stage conditions in the last year before death

3) Calculation of deaths due to dementia

In order to apply these estimates to the total excess deaths we then adjusted these proportions to calculate the proportion of individuals who died with severe, end-stage dementia out of excess dementia deaths using the formula:

$$\frac{Died\ with\ Severe\ Disease}{Excess\ Dementia\ Deaths} = \frac{Died\ with\ Severe\ Disease}{Total\ Dementia\ Deaths} * \frac{Relative\ Risk}{Relative\ Risk - 1}$$

We then calculated the number of deaths due to dementia as the product of total excess dementia deaths and the proportion of those who died with severe disease out of excess dementia deaths. These final estimates of deaths due to dementia were then used to adjust data on causes of death from all other causes in vital registration systems.

4) Interpolation for all years

Finally, we used log-linear interpolation to interpolate these results (limited to 1990, 1995, 2000, 2005, 2010, 2015, 2017, 2019, 2020, 2021, and 2022) to create estimates for the entire time series from 1980 to 2022. Socio-demographic Index was used as a covariate to extrapolate back to the year 1980.

References

1. Brauer, M., *et al.* Global burden and strength of evidence for 88 risk factors in 204 countries and 811 subnational locations, 1990–2021: a systematic analysis for the Global Burden of Disease Study 2021. *The Lancet* **403**, 2162-2203 (2024).
2. Ferrari, A.J., *et al.* Global incidence, prevalence, years lived with disability (YLDs), disability-adjusted life-years (DALYs), and healthy life expectancy (HALE) for 371 diseases and injuries

in 204 countries and territories and 811 subnational locations, 1990–2021: a systematic analysis for the Global Burden of Disease Study 2021. *The Lancet* **403**, 2133-2161 (2024).

3. Ong, K.L., *et al.* Global, regional, and national burden of diabetes from 1990 to 2021, with projections of prevalence to 2050: a systematic analysis for the Global Burden of Disease Study 2021. *The Lancet* **402**, 203-234 (2023).
4. Matthews, F.E., *et al.* A two-decade comparison of prevalence of dementia in individuals aged 65 years and older from three geographical areas of England: results of the Cognitive Function and Ageing Study I and II. *The Lancet* **382**, 1405-1412 (2013).

Appendix 3. Supplementary results

Supplementary tables

Table S2. The age-standardized DALY rates of ADRDs and its temporal change by sex and nation, 1990-2021

Location	Both			Female			Male		
	1990	2021	AAPC	1990	2021	AAPC	1990	2021	AAPC
Afghanistan	1805.78	1725.83	-0.15	2252.49	2134.20	-0.17	1435.50	1311.38	-0.29
	(1132.79, 2478.78)	(1103.89, 2347.76)	(-0.16, -0.13)	(1392.07, 3112.91)	(1341.69, 2926.71)	(-0.20, -0.15)	(916.85, 1954.14)	(854.01, 1768.76)	(-0.30, -0.27)
Albania	1203.85	1170.48	-0.10	1305.81	1298.01	-0.03	1042.92	1005.47	-0.12
	(805.95, 1601.74)	(782.79, 1558.17)	(-0.15, -0.06)	(881.77, 1729.85)	(868.90, 1727.11)	(-0.08, 0.01)	(676.18, 1409.66)	(670.31, 1340.63)	(-0.16, -0.09)
Algeria	1525.27	1431.09	-0.20	1722.98	1600.48	-0.23	1384.66	1301.07	-0.20
	(981.40, 2069.14)	(950.77, 1911.40)	(-0.21, -0.19)	(1109.47, 2336.48)	(1049.59, 2151.37)	(-0.25, -0.21)	(883.25, 1886.07)	(879.13, 1723.01)	(-0.22, -0.18)
American Samoa	1298.47	1210.83	-0.22	1502.56	1405.20	-0.21	1048.52	993.98	-0.15
	(846.62, 1750.32)	(807.56, 1614.11)	(-0.24, -0.19)	(985.83, 2019.29)	(932.40, 1878.00)	(-0.23, -0.19)	(676.92, 1420.11)	(658.88, 1329.09)	(-0.21, -0.10)
Andorra	1341.76	1249.60	-0.24	1490.54	1395.56	-0.22	1166.48	1075.93	-0.28
	(906.45, 1777.06)	(828.13, 1671.07)	(-0.28, -0.19)	(1026.17, 1954.91)	(952.28, 1838.84)	(-0.27, -0.18)	(758.94, 1574.02)	(687.69, 1464.18)	(-0.32, -0.24)
Angola	1529.21	1704.52	0.36	1849.38	2020.04	0.30	1141.35	1210.82	0.19
	(986.12, 2072.30)	(1069.62, 2339.43)	(0.33, 0.38)	(1217.78, 2480.99)	(1285.25, 2754.83)	(0.27, 0.32)	(699.85, 1582.85)	(748.16, 1673.47)	(0.16, 0.22)
Antigua and Barbuda	975.01	931.64	-0.13	1027.62	1002.20	-0.08	882.64	826.43	-0.19
	(656.58, 1293.45)	(628.15, 1235.13)	(-0.19, -0.07)	(701.02, 1354.21)	(680.55, 1323.85)	(-0.13, -0.03)	(583.47, 1181.82)	(553.01, 1099.86)	(-0.29, -0.08)
Argentina	1142.50	1104.07	-0.11	1289.66	1246.23	-0.11	895.06	866.13	-0.10
	(758.07, 1526.93)	(745.58, 1462.56)	(-0.12, -0.09)	(858.05, 1721.27)	(849.96, 1642.50)	(-0.14, -0.08)	(591.25, 1198.87)	(576.30, 1155.96)	(-0.12, -0.09)
Armenia	1145.52	1190.51	0.13	1232.29	1284.88	0.12	996.28	1041.84	0.14
	(772.49, 1518.55)	(804.30, 1576.71)	(0.08, 0.17)	(838.49, 1626.09)	(875.03, 1694.74)	(0.08, 0.16)	(661.42, 1331.15)	(698.75, 1384.94)	(0.09, 0.19)
Australia	1311.47	1190.94	-0.32	1503.41	1391.25	-0.25	961.50	929.91	-0.11
	(884.57, 1738.37)	(814.41, 1567.47)	(-0.38, -0.26)	(1027.35, 1979.47)	(964.92, 1817.57)	(-0.29, -0.22)	(635.50, 1287.50)	(615.95, 1243.87)	(-0.15, -0.06)
Austria	1340.60	1272.36	-0.17	1450.76	1405.19	-0.10	1087.22	1062.89	-0.08
	(874.68, 1806.53)	(856.88, 1687.84)	(-0.19, -0.15)	(957.63, 1943.88)	(961.05, 1849.32)	(-0.13, -0.08)	(698.28, 1476.15)	(690.56, 1435.21)	(-0.10, -0.05)
Azerbaijan	1186.52	1148.40	-0.11	1253.35	1232.24	-0.06	1018.93	1005.10	-0.04
	(798.96, 1574.08)	(755.42, 1541.39)	(-0.14, -0.07)	(854.00, 1652.71)	(815.13, 1649.35)	(-0.10, -0.01)	(662.82, 1375.05)	(652.54, 1357.66)	(-0.07, -0.02)
Bahamas	962.65	933.01	-0.12	1017.90	995.24	-0.09	864.50	842.37	-0.09
	(656.74, 1268.55)	(637.91, 1228.10)	(-0.18, -0.06)	(704.83, 1330.96)	(687.30, 1303.19)	(-0.20, 0.01)	(570.67, 1158.34)	(562.28, 1122.45)	(-0.14, -0.03)
Bahrain	1553.93	1388.54	-0.37	1687.29	1539.06	-0.29	1363.75	1208.78	-0.40
	(1000.53, 2107.32)	(888.59, 1888.50)	(-0.41, -0.34)	(1092.82, 2281.75)	(998.62, 2079.50)	(-0.33, -0.25)	(864.14, 1863.35)	(771.27, 1646.29)	(-0.45, -0.34)
Bangladesh	871.77	928.52	0.21	996.24	1056.64	0.21	781.86	813.16	0.16
	(574.40, 1169.15)	(587.73, 1269.31)	(0.15, 0.28)	(663.43, 1329.06)	(671.35, 1441.93)	(0.14, 0.28)	(504.57, 1059.14)	(513.74, 1112.57)	(0.09, 0.22)

Location	Both			Female			Male		
	1990	2021	AAPC	1990	2021	AAPC	1990	2021	AAPC
Barbados	953.15 (647.64, 1258.66)	937.40 (641.92, 1232.89)	-0.05 (-0.10, 0.01)	996.67 (684.56, 1308.77)	989.61 (685.62, 1293.60)	-0.02 (-0.07, 0.03)	877.50 (587.95, 1167.04)	864.92 (580.97, 1148.86)	-0.04 (-0.08, -0.01)
Belarus	1189.50 (796.13, 1582.86)	1176.64 (790.91, 1562.36)	-0.03 (-0.07, 0.00)	1269.55 (855.88, 1683.22)	1252.97 (850.40, 1655.55)	-0.04 (-0.07, -0.01)	996.29 (656.97, 1335.61)	987.54 (647.25, 1327.84)	-0.03 (-0.08, 0.02)
Belgium	1516.38 (1008.22, 2024.55)	1366.03 (929.23, 1802.83)	-0.35 (-0.38, -0.31)	1664.79 (1123.71, 2205.87)	1512.83 (1049.49, 1976.16)	-0.31 (-0.34, -0.28)	1204.46 (774.17, 1634.76)	1141.14 (742.90, 1539.37)	-0.16 (-0.21, -0.11)
Belize	987.61 (682.46, 1292.76)	959.48 (662.70, 1256.26)	-0.10 (-0.15, -0.05)	1040.72 (730.76, 1350.69)	1034.43 (720.07, 1348.79)	-0.03 (-0.11, 0.06)	924.74 (628.74, 1220.73)	882.24 (602.10, 1162.38)	-0.16 (-0.25, -0.07)
Benin	965.46 (630.00, 1300.92)	944.07 (606.86, 1281.28)	-0.07 (-0.12, -0.03)	1138.80 (747.37, 1530.23)	1077.82 (691.52, 1464.11)	-0.17 (-0.22, -0.13)	766.28 (495.06, 1037.50)	746.37 (477.45, 1015.29)	-0.08 (-0.12, -0.04)
Bermuda	996.98 (676.79, 1317.17)	964.61 (674.27, 1254.95)	-0.09 (-0.20, 0.02)	1062.24 (731.90, 1392.58)	1036.68 (737.45, 1335.90)	-0.06 (-0.19, 0.06)	883.67 (580.42, 1186.91)	854.94 (581.54, 1128.34)	-0.13 (-0.21, -0.04)
Bhutan	889.88 (596.08, 1183.68)	1006.79 (631.37, 1382.21)	0.40 (0.38, 0.42)	956.94 (647.01, 1266.87)	1142.56 (714.43, 1570.69)	0.58 (0.55, 0.60)	804.34 (514.86, 1093.82)	865.89 (536.58, 1195.20)	0.24 (0.22, 0.27)
Bolivia (Plurinational State of)	877.83 (579.09, 1176.58)	878.97 (592.57, 1165.36)	0.01 (-0.01, 0.02)	949.75 (626.83, 1272.68)	963.88 (653.93, 1273.83)	0.06 (0.03, 0.09)	780.23 (509.37, 1051.09)	770.35 (511.25, 1029.46)	-0.04 (-0.06, -0.03)
Bosnia and Herzegovina	1152.82 (772.97, 1532.68)	1118.61 (766.14, 1471.08)	-0.10 (-0.14, -0.06)	1245.68 (847.94, 1643.42)	1208.65 (835.62, 1581.67)	-0.10 (-0.15, -0.05)	1001.50 (655.36, 1347.64)	982.34 (661.98, 1302.71)	-0.05 (-0.10, -0.01)
Botswana	1253.28 (793.68, 1712.88)	1215.06 (795.21, 1634.91)	-0.11 (-0.17, -0.04)	1468.67 (934.99, 2002.35)	1383.76 (905.75, 1861.77)	-0.20 (-0.27, -0.12)	889.73 (571.19, 1208.26)	865.01 (557.82, 1172.20)	-0.07 (-0.12, -0.02)
Brazil	1529.38 (1017.69, 2041.07)	1505.59 (1026.27, 1984.92)	-0.05 (-0.08, -0.02)	1674.83 (1123.35, 2226.31)	1629.55 (1119.51, 2139.59)	-0.09 (-0.10, -0.08)	1329.71 (875.14, 1784.28)	1323.56 (888.41, 1758.71)	-0.02 (-0.04, 0.00)
Brunei Darussalam	1154.53 (746.46, 1562.61)	1185.71 (783.68, 1587.75)	0.08 (0.03, 0.13)	1298.40 (840.66, 1756.15)	1317.19 (872.05, 1762.33)	0.05 (0.01, 0.09)	924.26 (595.56, 1252.95)	939.73 (608.11, 1271.36)	0.04 (-0.05, 0.13)
Bulgaria	1147.16 (757.97, 1536.35)	1138.18 (765.56, 1510.80)	-0.02 (-0.05, 0.01)	1263.19 (839.98, 1686.40)	1230.89 (837.63, 1624.16)	-0.08 (-0.11, -0.05)	999.05 (652.74, 1345.36)	976.90 (645.86, 1307.93)	-0.07 (-0.10, -0.04)
Burkina Faso	1117.98 (704.50, 1531.45)	1021.19 (645.20, 1397.18)	-0.30 (-0.36, -0.24)	1364.24 (860.06, 1868.42)	1198.91 (757.69, 1640.14)	-0.43 (-0.52, -0.34)	815.02 (519.79, 1110.24)	777.12 (489.07, 1065.18)	-0.15 (-0.20, -0.11)
Burundi	1232.81 (809.09, 1656.52)	1289.90 (809.26, 1770.54)	0.15 (0.12, 0.18)	1457.43 (964.76, 1950.10)	1553.95 (983.42, 2124.47)	0.22 (0.19, 0.26)	925.28 (592.37, 1258.20)	983.33 (617.16, 1349.51)	0.19 (0.17, 0.22)
Cabo Verde	907.44 (606.59, 1208.30)	923.17 (612.20, 1234.13)	0.05 (0.01, 0.09)	1035.73 (701.09, 1370.37)	1025.49 (687.65, 1363.32)	-0.05 (-0.09, -0.01)	727.96 (478.98, 976.94)	731.54 (471.45, 991.63)	0.05 (-0.04, 0.15)
Cambodia	1232.27 (826.77, 1637.76)	1384.62 (898.24, 1871.00)	0.38 (0.36, 0.40)	1340.51 (903.95, 1777.08)	1507.14 (988.93, 2025.35)	0.38 (0.36, 0.40)	1060.79 (695.93, 1425.66)	1159.94 (737.71, 1582.17)	0.29 (0.28, 0.30)
Cameroon	1033.78 (661.10, 1406.47)	972.84 (596.73, 1348.96)	-0.19 (-0.24, -0.15)	1230.04 (782.03, 1678.05)	1128.38 (694.45, 1562.31)	-0.28 (-0.33, -0.22)	807.99 (516.62, 1099.36)	759.09 (469.06, 1049.12)	-0.21 (-0.24, -0.17)
Canada	1346.61 (956.89, 1736.33)	1246.27 (889.93, 1602.62)	-0.25 (-0.31, -0.18)	1461.01 (1048.58, 1873.45)	1361.16 (981.53, 1740.80)	-0.23 (-0.26, -0.20)	1155.51 (809.62, 1501.39)	1091.87 (766.30, 1417.44)	-0.19 (-0.23, -0.15)

Location	Both			Female			Male		
	1990	2021	AAPC	1990	2021	AAPC	1990	2021	AAPC
Central African Republic	1719.68 (1116.78, 2322.58)	1693.82 (1099.49, 2288.14)	-0.05 (-0.09, -0.02)	2105.11 (1383.38, 2826.85)	2046.71 (1335.13, 2758.30)	-0.09 (-0.13, -0.05)	1083.25 (681.72, 1484.78)	1031.11 (647.32, 1414.89)	-0.17 (-0.22, -0.12)
Chad	932.09 (607.95, 1256.24)	929.76 (600.24, 1259.28)	-0.00 (-0.02, 0.01)	1107.21 (719.79, 1494.63)	1121.88 (725.77, 1517.99)	0.04 (0.02, 0.07)	714.77 (461.60, 967.93)	750.21 (477.90, 1022.52)	0.16 (0.15, 0.18)
Chile	1094.39 (726.66, 1462.12)	1081.24 (740.54, 1421.94)	-0.05 (-0.09, -0.01)	1258.20 (843.69, 1672.72)	1231.98 (848.80, 1615.16)	-0.07 (-0.10, -0.04)	867.23 (573.46, 1161.00)	863.53 (581.39, 1145.68)	0.00 (-0.03, 0.04)
China	1596.63 (1027.29, 2165.97)	1680.03 (1127.36, 2232.70)	0.16 (0.09, 0.24)	1782.58 (1162.22, 2402.93)	1886.13 (1305.95, 2466.30)	0.18 (0.10, 0.26)	1284.11 (787.78, 1780.43)	1385.14 (877.92, 1892.35)	0.24 (0.18, 0.30)
Colombia	1072.79 (730.43, 1415.16)	1072.21 (748.34, 1396.08)	-0.01 (-0.03, 0.01)	1182.19 (808.69, 1555.69)	1169.45 (829.57, 1509.34)	-0.04 (-0.06, -0.02)	944.87 (641.06, 1248.69)	938.79 (640.18, 1237.41)	-0.04 (-0.07, 0.00)
Comoros	1304.27 (861.95, 1746.60)	1412.57 (889.86, 1935.29)	0.24 (0.21, 0.28)	1578.29 (1050.17, 2106.42)	1711.33 (1086.23, 2336.44)	0.25 (0.22, 0.27)	946.00 (621.46, 1270.54)	1025.58 (637.41, 1413.76)	0.26 (0.23, 0.30)
Congo	1748.03 (1117.00, 2379.07)	1709.00 (1097.49, 2320.50)	-0.08 (-0.11, -0.04)	2002.00 (1303.11, 2700.88)	1984.96 (1282.60, 2687.33)	-0.03 (-0.07, 0.01)	1354.68 (833.70, 1875.65)	1346.56 (853.76, 1839.37)	-0.01 (-0.04, 0.02)
Cook Islands	1332.35 (860.73, 1803.97)	1186.48 (798.85, 1574.11)	-0.38 (-0.40, -0.36)	1535.21 (1000.09, 2070.34)	1351.91 (919.83, 1783.99)	-0.42 (-0.44, -0.39)	1106.16 (701.10, 1511.21)	983.09 (641.97, 1324.20)	-0.38 (-0.40, -0.36)
Costa Rica	1089.53 (750.91, 1428.15)	1065.87 (747.79, 1383.95)	-0.07 (-0.08, -0.05)	1203.62 (836.65, 1570.60)	1171.00 (828.82, 1513.18)	-0.08 (-0.09, -0.06)	963.50 (660.26, 1266.74)	929.83 (641.68, 1217.98)	-0.10 (-0.15, -0.05)
Croatia	1185.79 (794.10, 1577.49)	1143.40 (770.45, 1516.35)	-0.11 (-0.18, -0.04)	1268.57 (854.63, 1682.52)	1234.79 (838.91, 1630.67)	-0.08 (-0.13, -0.02)	1019.87 (664.35, 1375.39)	991.45 (659.53, 1323.38)	-0.09 (-0.15, -0.03)
Cuba	914.53 (613.76, 1215.29)	904.90 (615.19, 1194.60)	-0.03 (-0.07, -0.00)	980.56 (665.42, 1295.69)	966.40 (661.20, 1271.60)	-0.05 (-0.08, -0.02)	845.16 (562.05, 1128.27)	827.14 (552.02, 1102.25)	-0.07 (-0.10, -0.04)
Cyprus	1477.46 (906.29, 2048.63)	1318.55 (854.86, 1782.25)	-0.37 (-0.41, -0.33)	1630.07 (1013.57, 2246.56)	1451.27 (957.19, 1945.35)	-0.37 (-0.40, -0.34)	1244.34 (759.02, 1729.67)	1112.07 (705.52, 1518.63)	-0.36 (-0.42, -0.31)
Czechia	1150.26 (761.09, 1539.43)	1143.20 (779.46, 1506.93)	-0.01 (-0.04, 0.01)	1227.88 (818.05, 1637.71)	1239.80 (850.36, 1629.24)	0.04 (0.01, 0.07)	993.80 (647.25, 1340.34)	986.65 (663.77, 1309.53)	-0.02 (-0.05, 0.01)
Côte d'Ivoire	978.43 (629.95, 1326.90)	966.51 (609.76, 1323.26)	-0.06 (-0.09, -0.03)	1175.59 (761.80, 1589.37)	1138.41 (720.76, 1556.05)	-0.10 (-0.19, -0.02)	760.80 (478.48, 1043.12)	756.01 (472.93, 1039.09)	-0.03 (-0.08, 0.03)
Democratic People's Republic of Korea	1263.20 (821.19, 1705.22)	1288.58 (839.77, 1737.39)	0.07 (0.05, 0.09)	1380.20 (904.17, 1856.24)	1410.91 (922.74, 1899.07)	0.07 (0.06, 0.09)	961.20 (603.58, 1318.82)	964.03 (616.12, 1311.94)	0.01 (-0.00, 0.03)
Democratic Republic of the Congo	1585.88 (1018.75, 2153.01)	1793.12 (1139.14, 2447.09)	0.40 (0.37, 0.42)	2000.46 (1304.30, 2696.62)	2118.19 (1351.88, 2884.50)	0.19 (0.16, 0.22)	1148.94 (714.67, 1583.21)	1215.53 (757.68, 1673.38)	0.18 (0.15, 0.21)
Denmark	1203.05 (775.74, 1630.36)	1151.42 (737.49, 1565.35)	-0.12 (-0.19, -0.06)	1246.20 (822.69, 1669.72)	1207.63 (786.03, 1629.23)	-0.09 (-0.14, -0.03)	1125.66 (701.53, 1549.78)	1065.63 (667.65, 1463.62)	-0.16 (-0.24, -0.09)
Djibouti	1365.61 (869.38, 1861.85)	1439.71 (919.49, 1959.93)	0.17 (0.14, 0.20)	1623.32 (1044.13, 2202.52)	1760.95 (1124.66, 2397.24)	0.26 (0.23, 0.29)	1005.06 (640.07, 1370.05)	1058.33 (676.65, 1440.00)	0.17 (0.14, 0.19)
Dominica	984.73 (666.65, 1302.80)	959.13 (649.76, 1268.50)	-0.08 (-0.11, -0.06)	1024.77 (697.08, 1352.47)	1001.21 (686.32, 1316.11)	-0.07 (-0.10, -0.05)	905.92 (594.05, 1217.79)	874.70 (583.74, 1165.65)	-0.12 (-0.15, -0.09)

Location	Both			Female			Male		
	1990	2021	AAPC	1990	2021	AAPC	1990	2021	AAPC
Dominican Republic	992.61<br>(678.67, 1306.56)	970.13<br>(675.39, 1264.87)	-0.07<br>(-0.10, -0.04)	1081.47<br>(742.95, 1419.99)	1052.68<br>(740.80, 1364.57)	-0.08<br>(-0.12, -0.05)	900.34<br>(607.11, 1193.57)	870.54<br>(589.36, 1151.73)	-0.10<br>(-0.14, -0.06)
Ecuador	838.57<br>(563.05, 1114.09)	809.21<br>(551.19, 1067.22)	-0.12<br>(-0.14, -0.10)	928.14<br>(627.52, 1228.77)	889.14<br>(615.24, 1163.04)	-0.14<br>(-0.19, -0.10)	740.12<br>(492.87, 987.36)	705.33<br>(467.92, 942.74)	-0.16<br>(-0.20, -0.13)
Egypt	1467.85<br>(940.23, 1995.46)	1349.24<br>(884.83, 1813.66)	-0.27<br>(-0.33, -0.21)	1617.08<br>(1037.82, 2196.33)	1508.88<br>(972.10, 2045.66)	-0.22<br>(-0.28, -0.16)	1351.93<br>(866.30, 1837.56)	1260.08<br>(825.73, 1694.42)	-0.22<br>(-0.30, -0.14)
El Salvador	1093.09<br>(745.80, 1440.39)	1081.00<br>(760.08, 1401.93)	-0.03<br>(-0.04, -0.02)	1204.71<br>(828.80, 1580.61)	1179.67<br>(838.25, 1521.08)	-0.05<br>(-0.09, -0.02)	953.77<br>(644.66, 1262.89)	938.07<br>(643.40, 1232.75)	-0.07<br>(-0.14, 0.01)
Equatorial Guinea	1583.34<br>(1013.90, 2152.78)	1679.95<br>(1070.80, 2289.10)	0.18<br>(0.15, 0.22)	1865.76<br>(1207.52, 2524.00)	1997.92<br>(1294.08, 2701.76)	0.22<br>(0.19, 0.25)	1161.38<br>(713.36, 1609.39)	1186.85<br>(737.59, 1636.12)	0.07<br>(0.04, 0.10)
Eritrea	1304.94<br>(846.00, 1763.88)	1491.99<br>(939.98, 2044.00)	0.44<br>(0.41, 0.46)	1485.74<br>(965.76, 2005.71)	1715.80<br>(1071.77, 2359.83)	0.47<br>(0.46, 0.49)	904.76<br>(576.74, 1232.77)	1004.66<br>(626.24, 1383.08)	0.34<br>(0.31, 0.36)
Estonia	1173.34<br>(779.14, 1567.54)	1203.77<br>(810.48, 1597.06)	0.08<br>(0.05, 0.11)	1244.58<br>(834.34, 1654.83)	1278.15<br>(872.22, 1684.08)	0.09<br>(0.05, 0.12)	977.36<br>(638.96, 1315.76)	1036.62<br>(674.87, 1398.37)	0.19<br>(0.16, 0.22)
Eswatini	1253.46<br>(810.48, 1696.44)	1257.48<br>(804.96, 1710.00)	0.01<br>(-0.00, 0.03)	1415.39<br>(921.89, 1908.89)	1411.53<br>(898.77, 1924.29)	-0.01<br>(-0.02, 0.00)	910.85<br>(580.20, 1241.50)	867.91<br>(540.81, 1195.01)	-0.16<br>(-0.18, -0.14)
Ethiopia	1410.04<br>(896.66, 1923.41)	1440.69<br>(928.26, 1953.13)	0.07<br>(0.04, 0.09)	1684.22<br>(1064.85, 2303.59)	1788.33<br>(1172.23, 2404.43)	0.19<br>(0.16, 0.22)	1080.89<br>(680.75, 1481.03)	1096.81<br>(701.64, 1491.97)	0.05<br>(0.03, 0.08)
Fiji	1270.35<br>(822.82, 1717.89)	1209.68<br>(809.20, 1610.16)	-0.16<br>(-0.19, -0.13)	1447.15<br>(942.71, 1951.59)	1330.74<br>(899.65, 1761.82)	-0.28<br>(-0.31, -0.24)	1017.59<br>(646.41, 1388.77)	973.46<br>(637.10, 1309.83)	-0.14<br>(-0.17, -0.12)
Finland	1423.52<br>(924.80, 1922.23)	1308.77<br>(874.92, 1742.62)	-0.27<br>(-0.30, -0.25)	1484.22<br>(977.36, 1991.09)	1390.04<br>(946.51, 1833.57)	-0.23<br>(-0.26, -0.20)	1274.79<br>(796.33, 1753.26)	1181.41<br>(765.89, 1596.94)	-0.24<br>(-0.28, -0.21)
France	1159.15<br>(759.89, 1558.41)	1095.93<br>(741.82, 1450.04)	-0.18<br>(-0.20, -0.16)	1251.93<br>(828.43, 1675.43)	1184.48<br>(817.60, 1551.37)	-0.18<br>(-0.20, -0.16)	968.98<br>(619.35, 1318.61)	958.15<br>(616.18, 1300.12)	-0.04<br>(-0.07, -0.01)
Gabon	1769.95<br>(1157.84, 2382.06)	1757.41<br>(1128.83, 2385.98)	-0.02<br>(-0.04, 0.00)	2070.24<br>(1374.41, 2766.07)	2052.07<br>(1331.20, 2772.95)	-0.03<br>(-0.06, 0.01)	1283.84<br>(808.46, 1759.22)	1261.89<br>(777.03, 1746.76)	-0.06<br>(-0.07, -0.04)
Gambia	1019.56<br>(658.16, 1380.96)	1019.81<br>(631.91, 1407.71)	-0.00<br>(-0.09, 0.08)	1187.56<br>(760.91, 1614.22)	1178.31<br>(730.29, 1626.33)	-0.02<br>(-0.11, 0.08)	783.98<br>(509.13, 1058.83)	792.67<br>(488.57, 1096.77)	0.02<br>(-0.11, 0.14)
Georgia	1170.37<br>(785.64, 1555.10)	1191.43<br>(797.28, 1585.59)	0.06<br>(-0.04, 0.16)	1244.47<br>(840.98, 1647.96)	1272.23<br>(859.20, 1685.26)	0.06<br>(-0.02, 0.14)	995.54<br>(653.85, 1337.23)	1038.02<br>(679.56, 1396.48)	0.14<br>(0.06, 0.23)
Germany	1526.28<br>(1028.87, 2023.70)	1519.47<br>(1054.97, 1983.97)	-0.00<br>(-0.06, 0.05)	1662.84<br>(1128.30, 2197.39)	1681.64<br>(1184.19, 2179.10)	0.05<br>(-0.00, 0.11)	1190.13<br>(787.28, 1592.98)	1256.72<br>(843.88, 1669.57)	0.19<br>(0.14, 0.24)
Ghana	888.74<br>(578.03, 1199.45)	979.14<br>(603.41, 1354.87)	0.30<br>(0.26, 0.35)	1054.15<br>(681.20, 1427.09)	1139.08<br>(693.97, 1584.19)	0.24<br>(0.20, 0.29)	684.61<br>(443.79, 925.44)	711.21<br>(444.53, 977.89)	0.12<br>(0.09, 0.15)
Greece	1322.22<br>(861.72, 1782.72)	1283.20<br>(859.44, 1706.96)	-0.10<br>(-0.12, -0.08)	1471.69<br>(967.72, 1975.66)	1441.54<br>(983.70, 1899.39)	-0.07<br>(-0.09, -0.05)	1116.07<br>(717.62, 1514.52)	1087.47<br>(706.85, 1468.09)	-0.09<br>(-0.10, -0.07)
Greenland	1449.87<br>(944.03, 1955.72)	1406.03<br>(960.55, 1851.52)	-0.09<br>(-0.15, -0.02)	1560.33<br>(1029.62, 2091.03)	1555.60<br>(1072.02, 2039.18)	0.00<br>(-0.10, 0.10)	1240.40<br>(806.08, 1674.71)	1218.76<br>(823.41, 1614.11)	-0.05<br>(-0.09, -0.01)

Location	Both			Female			Male		
	1990	2021	AAPC	1990	2021	AAPC	1990	2021	AAPC
Grenada	1008.17 (677.88, 1338.46)	975.57 (657.61, 1293.52)	-0.10 (-0.18, -0.02)	1090.13 (740.02, 1440.24)	1027.19 (698.75, 1355.63)	-0.13 (-0.20, -0.05)	855.33 (569.53, 1141.13)	816.74 (529.26, 1104.22)	-0.14 (-0.23, -0.05)
Guam	1259.33 (830.25, 1688.40)	1100.00 (792.94, 1407.06)	-0.46 (-0.53, -0.38)	1459.07 (964.05, 1954.09)	1216.67 (892.58, 1540.75)	-0.61 (-0.68, -0.54)	1050.22 (689.00, 1411.45)	947.60 (656.44, 1238.75)	-0.35 (-0.50, -0.19)
Guatemala	1102.40 (735.43, 1469.38)	1076.32 (744.15, 1408.48)	-0.09 (-0.11, -0.06)	1206.15 (812.25, 1600.05)	1182.06 (822.66, 1541.47)	-0.07 (-0.10, -0.03)	977.72 (643.06, 1312.38)	942.33 (644.75, 1239.91)	-0.13 (-0.16, -0.10)
Guinea	926.95 (609.39, 1244.51)	959.03 (618.47, 1299.58)	0.11 (0.09, 0.14)	1113.32 (725.91, 1500.73)	1141.50 (735.98, 1547.02)	0.08 (0.06, 0.11)	742.91 (484.19, 1001.63)	760.85 (481.36, 1040.34)	0.08 (0.06, 0.10)
Guinea-Bissau	983.01 (628.98, 1337.03)	1010.88 (626.01, 1395.76)	0.08 (0.06, 0.10)	1145.54 (734.23, 1556.86)	1160.62 (712.17, 1609.06)	0.04 (0.00, 0.07)	779.12 (491.43, 1066.81)	776.96 (482.48, 1071.44)	-0.01 (-0.04, 0.01)
Guyana	907.70 (617.03, 1198.37)	919.08 (628.72, 1209.45)	0.05 (-0.11, 0.21)	979.34 (675.02, 1283.66)	988.07 (686.67, 1289.46)	0.03 (-0.28, 0.34)	825.54 (551.30, 1099.78)	828.88 (558.90, 1098.86)	0.04 (-0.12, 0.19)
Haiti	1017.47 (680.66, 1354.28)	962.65 (639.60, 1285.69)	-0.17 (-0.19, -0.15)	1065.06 (725.28, 1404.84)	1013.93 (684.04, 1343.83)	-0.16 (-0.17, -0.15)	950.65 (613.98, 1287.31)	909.25 (584.32, 1234.18)	-0.15 (-0.16, -0.13)
Honduras	1184.40 (805.67, 1563.13)	1277.73 (842.44, 1713.01)	0.25 (0.20, 0.30)	1280.14 (877.44, 1682.83)	1404.70 (934.28, 1875.12)	0.31 (0.26, 0.37)	1060.08 (718.74, 1401.41)	1126.56 (736.82, 1516.30)	0.19 (0.18, 0.21)
Hungary	1152.50 (762.90, 1542.10)	1138.06 (775.00, 1501.13)	-0.03 (-0.06, 0.00)	1233.87 (823.34, 1644.41)	1223.13 (841.75, 1604.52)	-0.02 (-0.05, 0.01)	998.16 (648.12, 1348.20)	975.41 (650.60, 1300.22)	-0.06 (-0.10, -0.02)
Iceland	1357.87 (920.21, 1795.54)	1283.30 (880.13, 1686.48)	-0.19 (-0.25, -0.14)	1492.01 (1012.23, 1971.79)	1414.50 (975.34, 1853.66)	-0.19 (-0.25, -0.13)	1161.53 (785.46, 1537.60)	1110.95 (755.03, 1466.88)	-0.14 (-0.23, -0.06)
India	782.89 (513.32, 1052.46)	913.31 (593.42, 1233.20)	0.51 (0.44, 0.58)	886.54 (585.60, 1187.48)	1027.47 (670.24, 1384.70)	0.50 (0.42, 0.58)	676.52 (436.06, 916.98)	770.65 (498.87, 1042.42)	0.44 (0.34, 0.55)
Indonesia	1090.59 (745.72, 1435.45)	1260.65 (819.69, 1701.62)	0.47 (0.45, 0.49)	1236.87 (853.57, 1620.17)	1427.81 (924.23, 1931.39)	0.47 (0.44, 0.51)	909.90 (625.94, 1193.86)	1032.64 (671.68, 1393.59)	0.41 (0.39, 0.43)
Iran (Islamic Republic of)	1512.90 (1006.96, 2018.84)	1414.92 (955.89, 1873.95)	-0.22 (-0.23, -0.21)	1651.80 (1108.75, 2194.86)	1574.48 (1074.19, 2074.76)	-0.15 (-0.16, -0.14)	1359.28 (899.81, 1818.76)	1262.62 (844.47, 1680.78)	-0.24 (-0.25, -0.22)
Iraq	1465.85 (993.88, 1937.82)	1413.43 (939.70, 1887.15)	-0.11 (-0.14, -0.07)	1597.52 (1092.60, 2102.43)	1538.84 (1035.85, 2041.83)	-0.11 (-0.14, -0.08)	1316.08 (881.87, 1750.28)	1254.71 (817.04, 1692.39)	-0.14 (-0.17, -0.11)
Ireland	1326.89 (858.68, 1795.11)	1246.51 (843.00, 1650.02)	-0.20 (-0.24, -0.16)	1456.74 (955.43, 1958.06)	1380.51 (953.97, 1807.06)	-0.18 (-0.22, -0.13)	1100.94 (699.37, 1502.51)	1062.34 (695.04, 1429.65)	-0.11 (-0.13, -0.09)
Israel	1293.89 (840.87, 1746.91)	1259.16 (850.02, 1668.30)	-0.09 (-0.12, -0.06)	1448.66 (955.96, 1941.36)	1397.74 (961.22, 1834.25)	-0.12 (-0.15, -0.09)	1109.31 (708.22, 1510.41)	1068.68 (701.79, 1435.57)	-0.12 (-0.17, -0.08)
Italy	1543.32 (1000.05, 2086.59)	1515.77 (1037.08, 1994.46)	-0.06 (-0.10, -0.02)	1696.71 (1113.60, 2279.81)	1701.28 (1189.27, 2213.29)	0.01 (-0.03, 0.05)	1259.92 (793.20, 1726.64)	1220.91 (798.70, 1643.13)	-0.12 (-0.17, -0.07)
Jamaica	1018.01 (710.66, 1325.35)	981.42 (687.05, 1275.79)	-0.11 (-0.17, -0.05)	1084.71 (766.68, 1402.75)	1057.46 (750.28, 1364.64)	-0.08 (-0.14, -0.02)	926.65 (634.61, 1218.70)	880.77 (600.74, 1160.81)	-0.13 (-0.21, -0.05)
Japan	1375.88 (910.65, 1841.11)	1362.21 (957.11, 1767.31)	-0.03 (-0.06, -0.01)	1547.04 (1038.91, 2055.17)	1541.69 (1113.94, 1969.44)	-0.02 (-0.05, 0.02)	1065.60 (679.43, 1451.76)	1077.28 (708.67, 1445.90)	0.03 (0.01, 0.05)

Location	Both			Female			Male		
	1990	2021	AAPC	1990	2021	AAPC	1990	2021	AAPC
Jordan	1456.15 (979.98, 1932.32)	1344.36 (902.51, 1786.21)	-0.26 (-0.29, -0.22)	1617.08 (1085.63, 2148.54)	1513.03 (1026.12, 1999.94)	-0.21 (-0.27, -0.15)	1296.88 (872.01, 1721.75)	1203.32 (802.75, 1603.90)	-0.24 (-0.27, -0.20)
Kazakhstan	1167.72 (780.88, 1554.56)	1109.97 (736.22, 1483.73)	-0.17 (-0.21, -0.13)	1236.63 (833.57, 1639.68)	1179.05 (791.18, 1566.92)	-0.16 (-0.19, -0.12)	995.91 (654.37, 1337.45)	953.09 (622.87, 1283.30)	-0.14 (-0.18, -0.09)
Kenya	1266.88 (833.14, 1700.63)	1435.62 (937.76, 1933.48)	0.40 (0.36, 0.43)	1587.77 (1048.39, 2127.15)	1702.29 (1122.63, 2281.95)	0.21 (0.17, 0.26)	919.37 (601.76, 1236.97)	970.68 (636.84, 1304.52)	0.17 (0.12, 0.23)
Kiribati	1328.54 (866.05, 1791.04)	1446.48 (939.37, 1953.59)	0.28 (0.27, 0.28)	1471.41 (964.50, 1978.32)	1617.97 (1051.04, 2184.91)	0.31 (0.30, 0.32)	1092.58 (682.32, 1502.85)	1127.56 (713.65, 1541.48)	0.10 (0.09, 0.11)
Kuwait	1506.13 (1020.89, 1991.37)	1367.46 (938.47, 1796.45)	-0.31 (-0.40, -0.22)	1696.25 (1166.46, 2226.04)	1533.41 (1083.08, 1983.74)	-0.33 (-0.45, -0.21)	1320.32 (880.18, 1760.46)	1249.07 (823.77, 1674.37)	-0.21 (-0.36, -0.07)
Kyrgyzstan	1183.34 (795.38, 1571.29)	1156.07 (789.78, 1522.36)	-0.07 (-0.10, -0.04)	1253.22 (848.89, 1657.56)	1235.04 (853.87, 1616.21)	-0.04 (-0.07, -0.01)	1012.06 (671.13, 1352.99)	1002.23 (664.48, 1339.98)	-0.03 (-0.08, 0.01)
Lao People's Democratic Republic	1162.33 (783.77, 1540.89)	1217.25 (796.24, 1638.27)	0.15 (0.13, 0.17)	1294.49 (874.30, 1714.68)	1381.13 (909.95, 1852.31)	0.21 (0.19, 0.24)	962.56 (637.54, 1287.58)	1000.87 (646.74, 1354.99)	0.13 (0.11, 0.15)
Latvia	1173.69 (781.30, 1566.09)	1171.51 (786.75, 1556.26)	0.00 (-0.03, 0.03)	1246.62 (838.78, 1654.47)	1243.15 (842.48, 1643.82)	-0.01 (-0.05, 0.04)	992.56 (652.29, 1332.82)	998.47 (658.46, 1338.48)	0.01 (-0.03, 0.05)
Lebanon	1414.91 (955.18, 1874.64)	1374.57 (945.89, 1803.25)	-0.09 (-0.13, -0.06)	1608.89 (1089.40, 2128.39)	1555.01 (1076.75, 2033.27)	-0.10 (-0.14, -0.07)	1166.91 (779.96, 1553.87)	1159.97 (781.28, 1538.67)	-0.01 (-0.07, 0.05)
Lesotho	1235.48 (829.20, 1641.75)	1286.27 (811.74, 1760.79)	0.13 (0.10, 0.16)	1367.01 (921.42, 1812.60)	1427.80 (900.53, 1955.06)	0.14 (0.10, 0.17)	872.03 (567.23, 1176.84)	865.77 (556.61, 1174.94)	-0.02 (-0.05, 0.02)
Liberia	921.11 (578.12, 1264.11)	918.05 (576.49, 1259.61)	-0.02 (-0.08, 0.05)	1115.36 (707.50, 1523.22)	1103.63 (692.08, 1515.18)	-0.04 (-0.12, 0.04)	749.61 (468.68, 1030.53)	735.02 (462.25, 1007.79)	-0.06 (-0.11, -0.02)
Libya	1644.82 (1118.11, 2171.53)	1500.14 (995.01, 2005.26)	-0.30 (-0.36, -0.25)	1818.15 (1252.25, 2384.06)	1657.74 (1108.96, 2206.52)	-0.30 (-0.36, -0.24)	1473.97 (989.95, 1957.98)	1337.12 (857.03, 1817.22)	-0.32 (-0.37, -0.28)
Lithuania	1170.04 (781.02, 1559.06)	1175.32 (789.85, 1560.80)	0.02 (-0.01, 0.05)	1264.48 (855.20, 1673.76)	1253.64 (850.74, 1656.53)	-0.02 (-0.06, 0.01)	988.75 (649.39, 1328.12)	997.85 (657.66, 1338.05)	0.02 (-0.02, 0.06)
Luxembourg	973.61 (647.40, 1299.81)	920.75 (616.13, 1225.37)	-0.18 (-0.24, -0.13)	1047.30 (702.24, 1392.37)	1001.36 (679.20, 1323.53)	-0.14 (-0.20, -0.09)	820.39 (539.70, 1101.08)	796.87 (517.64, 1076.11)	-0.10 (-0.17, -0.03)
Madagascar	1076.72 (714.72, 1438.72)	1092.26 (726.65, 1457.88)	0.05 (0.03, 0.07)	1341.81 (896.67, 1786.95)	1325.61 (885.30, 1765.92)	-0.03 (-0.06, -0.01)	813.57 (537.90, 1089.24)	799.14 (530.73, 1067.56)	-0.05 (-0.08, -0.02)
Malawi	1261.35 (812.84, 1709.86)	1385.37 (876.32, 1894.43)	0.32 (0.27, 0.37)	1527.26 (984.78, 2069.74)	1605.62 (1021.74, 2189.50)	0.17 (0.14, 0.21)	933.00 (593.47, 1272.54)	971.88 (603.55, 1340.21)	0.16 (0.10, 0.21)
Malaysia	1329.65 (894.29, 1765.01)	1330.37 (868.86, 1791.87)	0.00 (-0.05, 0.06)	1558.70 (1053.20, 2064.19)	1597.81 (1042.88, 2152.75)	0.07 (0.05, 0.10)	1065.53 (705.82, 1425.24)	1070.90 (699.54, 1442.25)	0.01 (-0.04, 0.05)
Maldives	1083.75 (735.39, 1432.11)	1149.69 (787.28, 1512.10)	0.19 (0.17, 0.20)	1294.27 (889.07, 1699.46)	1345.46 (927.84, 1763.09)	0.12 (0.06, 0.19)	980.58 (661.58, 1299.58)	970.77 (659.49, 1282.04)	-0.04 (-0.05, -0.02)
Mali	1095.45 (692.91, 1497.99)	1086.21 (674.24, 1498.18)	-0.02 (-0.04, -0.00)	1316.02 (837.97, 1794.07)	1330.73 (823.69, 1837.76)	0.04 (0.01, 0.07)	856.79 (540.74, 1172.84)	860.26 (530.43, 1190.09)	0.02 (-0.03, 0.06)

Location	Both			Female			Male		
	1990	2021	AAPC	1990	2021	AAPC	1990	2021	AAPC
Malta	1320.81 (862.56, 1779.06)	1260.58 (853.78, 1667.37)	-0.15 (-0.24, -0.07)	1451.27 (960.18, 1942.37)	1412.05 (965.40, 1858.70)	-0.15 (-0.17, -0.13)	1105.83 (714.42, 1497.23)	1037.37 (690.95, 1383.78)	-0.20 (-0.33, -0.07)
Marshall Islands	1345.25 (855.06, 1835.44)	1257.61 (796.34, 1718.87)	-0.22 (-0.23, -0.20)	1487.27 (959.83, 2014.70)	1419.79 (898.87, 1940.71)	-0.15 (-0.16, -0.14)	1122.91 (691.94, 1553.88)	1103.88 (689.48, 1518.27)	-0.05 (-0.07, -0.03)
Mauritania	989.30 (643.48, 1335.11)	983.07 (616.10, 1350.04)	-0.03 (-0.10, 0.04)	1156.87 (749.90, 1563.84)	1181.43 (735.79, 1627.07)	0.05 (-0.01, 0.11)	763.93 (493.51, 1034.36)	792.10 (496.49, 1087.71)	0.11 (0.07, 0.16)
Mauritius	1225.49 (816.85, 1634.13)	1165.29 (785.88, 1544.71)	-0.16 (-0.26, -0.07)	1345.56 (905.21, 1785.91)	1317.39 (887.65, 1747.12)	-0.08 (-0.16, -0.00)	995.44 (655.33, 1335.56)	928.21 (625.79, 1230.63)	-0.23 (-0.30, -0.16)
Mexico	924.28 (631.00, 1217.56)	886.77 (602.94, 1170.59)	-0.14 (-0.16, -0.12)	1047.58 (715.74, 1379.42)	988.73 (671.53, 1305.93)	-0.19 (-0.22, -0.17)	812.53 (552.88, 1072.19)	763.29 (519.17, 1007.40)	-0.20 (-0.23, -0.18)
Micronesia (Federated States of)	1422.16 (915.30, 1929.03)	1399.74 (901.02, 1898.46)	-0.05 (-0.07, -0.03)	1590.44 (1032.63, 2148.25)	1551.57 (1011.17, 2091.98)	-0.08 (-0.10, -0.07)	1182.48 (738.59, 1626.36)	1160.37 (738.65, 1582.09)	-0.06 (-0.08, -0.04)
Monaco	1355.40 (888.50, 1822.31)	1346.25 (898.27, 1794.23)	-0.02 (-0.03, -0.01)	1486.51 (988.14, 1984.87)	1501.58 (1022.63, 1980.54)	0.03 (0.02, 0.04)	1117.96 (709.32, 1526.60)	1126.28 (730.74, 1521.81)	0.02 (0.01, 0.04)
Mongolia	1233.35 (815.95, 1650.74)	1201.72 (799.33, 1604.11)	-0.08 (-0.16, 0.01)	1323.84 (881.71, 1765.98)	1280.40 (859.06, 1701.75)	-0.12 (-0.22, -0.02)	1075.20 (701.51, 1448.88)	1065.51 (697.91, 1433.12)	-0.05 (-0.12, 0.03)
Montenegro	1171.73 (790.51, 1552.96)	1146.22 (764.64, 1527.79)	-0.06 (-0.11, -0.02)	1267.03 (865.49, 1668.57)	1247.39 (836.89, 1657.88)	-0.04 (-0.09, 0.01)	1025.47 (677.03, 1373.90)	991.32 (652.48, 1330.16)	-0.11 (-0.14, -0.07)
Morocco	1487.87 (994.20, 1981.55)	1432.94 (951.09, 1914.79)	-0.12 (-0.13, -0.11)	1652.93 (1117.15, 2188.72)	1567.02 (1038.99, 2095.04)	-0.17 (-0.18, -0.17)	1322.60 (869.52, 1775.67)	1285.17 (841.28, 1729.05)	-0.09 (-0.10, -0.08)
Mozambique	1364.46 (872.30, 1856.62)	1465.16 (891.04, 2039.28)	0.24 (0.20, 0.28)	1609.38 (1044.50, 2174.26)	1662.04 (1027.97, 2296.10)	0.11 (0.07, 0.15)	1039.37 (642.54, 1436.20)	1072.62 (645.06, 1500.19)	0.10 (0.07, 0.14)
Myanmar	1166.51 (798.83, 1534.18)	1217.23 (803.42, 1631.04)	0.15 (0.13, 0.16)	1358.12 (919.66, 1796.58)	1376.96 (911.45, 1842.48)	0.05 (0.04, 0.07)	893.46 (615.52, 1171.40)	951.80 (626.42, 1277.19)	0.21 (0.19, 0.22)
Namibia	1202.83 (792.66, 1613.00)	1293.23 (818.43, 1768.03)	0.23 (0.21, 0.26)	1393.67 (923.02, 1864.32)	1463.63 (928.72, 1998.54)	0.16 (0.14, 0.18)	863.89 (558.47, 1169.31)	934.35 (586.93, 1281.76)	0.25 (0.23, 0.28)
Nauru	1335.67 (849.22, 1822.11)	1339.52 (855.46, 1823.57)	0.01 (-0.00, 0.02)	1672.86 (1059.68, 2286.04)	1515.16 (959.26, 2071.07)	-0.32 (-0.33, -0.31)	1138.91 (724.76, 1553.05)	1098.55 (692.98, 1504.13)	-0.12 (-0.13, -0.11)
Nepal	839.16 (564.12, 1114.19)	928.61 (599.58, 1257.65)	0.33 (0.31, 0.36)	954.95 (644.23, 1265.68)	1050.03 (679.12, 1420.94)	0.31 (0.29, 0.34)	719.09 (480.12, 958.06)	785.71 (504.34, 1067.07)	0.29 (0.27, 0.31)
Netherlands	1440.21 (958.51, 1921.91)	1418.92 (950.32, 1887.51)	-0.05 (-0.06, -0.03)	1560.99 (1052.53, 2069.46)	1570.21 (1071.32, 2069.10)	0.02 (-0.01, 0.05)	1201.97 (772.01, 1631.93)	1201.00 (774.31, 1627.69)	-0.01 (-0.05, 0.03)
New Zealand	1391.17 (934.28, 1848.07)	1317.91 (897.71, 1738.12)	-0.18 (-0.21, -0.15)	1572.72 (1075.39, 2070.04)	1527.60 (1055.33, 1999.87)	-0.10 (-0.13, -0.06)	1061.03 (694.77, 1427.29)	1039.77 (687.10, 1392.45)	-0.07 (-0.11, -0.03)
Nicaragua	1091.79 (759.70, 1423.89)	1066.00 (756.40, 1375.60)	-0.09 (-0.12, -0.07)	1183.78 (831.39, 1536.17)	1162.43 (834.17, 1490.70)	-0.06 (-0.11, -0.01)	953.18 (643.84, 1262.51)	923.45 (636.38, 1210.52)	-0.11 (-0.17, -0.05)
Niger	988.53 (636.77, 1340.29)	988.86 (627.25, 1350.46)	0.00 (-0.04, 0.04)	1146.80 (744.12, 1549.48)	1164.36 (746.23, 1582.49)	0.05 (0.01, 0.09)	779.75 (506.15, 1053.36)	802.44 (502.38, 1102.50)	0.10 (0.07, 0.13)

Location	Both			Female			Male		
	1990	2021	AAPC	1990	2021	AAPC	1990	2021	AAPC
Nigeria	886.77 (566.66, 1206.87)	924.41 (578.70, 1270.13)	0.13 (0.09, 0.17)	1000.28 (638.27, 1362.28)	1120.02 (705.82, 1534.21)	0.37 (0.30, 0.43)	719.75 (456.99, 982.52)	706.37 (420.83, 991.90)	-0.08 (-0.12, -0.04)
Niue	1430.05 (911.31, 1948.79)	1296.89 (843.63, 1750.15)	-0.32 (-0.34, -0.30)	1574.20 (1013.05, 2135.34)	1430.66 (940.30, 1921.02)	-0.31 (-0.33, -0.29)	1147.50 (724.86, 1570.14)	1071.55 (682.89, 1460.21)	-0.22 (-0.25, -0.19)
North Macedonia	1108.95 (749.48, 1468.43)	1117.12 (741.61, 1492.63)	0.02 (0.00, 0.05)	1204.58 (818.23, 1590.92)	1215.28 (813.37, 1617.19)	0.03 (-0.00, 0.05)	989.93 (655.55, 1324.30)	976.51 (639.37, 1313.64)	-0.05 (-0.07, -0.02)
Northern Mariana Islands	1256.73 (823.01, 1690.46)	1227.66 (805.73, 1649.60)	-0.13 (-0.22, -0.05)	1459.81 (967.35, 1952.28)	1395.07 (926.93, 1863.20)	-0.21 (-0.31, -0.10)	1057.75 (684.54, 1430.97)	1032.24 (663.58, 1400.90)	-0.10 (-0.16, -0.04)
Norway	1426.32 (948.18, 1904.47)	1277.87 (852.23, 1703.51)	-0.35 (-0.37, -0.33)	1536.83 (1038.50, 2035.15)	1396.82 (946.91, 1846.73)	-0.31 (-0.33, -0.29)	1235.06 (796.15, 1673.98)	1108.80 (720.02, 1497.58)	-0.33 (-0.37, -0.30)
Oman	1532.04 (1018.19, 2045.88)	1410.86 (915.47, 1906.25)	-0.27 (-0.34, -0.19)	1657.82 (1123.49, 2192.14)	1538.58 (1020.98, 2056.19)	-0.25 (-0.32, -0.17)	1366.71 (891.68, 1841.73)	1259.90 (784.70, 1735.10)	-0.28 (-0.38, -0.17)
Pakistan	928.81 (602.65, 1254.98)	997.30 (634.39, 1360.20)	0.22 (0.19, 0.25)	1099.57 (708.41, 1490.73)	1188.20 (751.28, 1625.11)	0.25 (0.20, 0.29)	805.16 (521.70, 1088.62)	823.51 (519.59, 1127.44)	0.07 (0.04, 0.11)
Palau	1301.55 (836.58, 1766.51)	1164.10 (736.86, 1591.34)	-0.36 (-0.39, -0.33)	1494.28 (967.45, 2021.12)	1385.44 (866.48, 1904.40)	-0.24 (-0.26, -0.23)	1062.71 (684.35, 1441.07)	980.20 (630.44, 1329.96)	-0.26 (-0.30, -0.22)
Palestine	1478.09 (967.16, 1989.03)	1379.45 (912.25, 1846.64)	-0.24 (-0.26, -0.22)	1608.33 (1063.37, 2153.29)	1498.11 (1001.22, 1994.99)	-0.25 (-0.26, -0.23)	1293.04 (827.20, 1758.88)	1195.12 (777.94, 1612.30)	-0.25 (-0.28, -0.23)
Panama	1045.38 (723.66, 1367.11)	1042.17 (738.85, 1345.49)	-0.00 (-0.03, 0.03)	1154.53 (803.00, 1506.07)	1154.51 (825.67, 1483.35)	0.02 (-0.02, 0.07)	923.52 (630.62, 1216.41)	907.07 (632.99, 1181.15)	-0.05 (-0.08, -0.02)
Papua New Guinea	1235.91 (828.29, 1643.52)	1175.20 (767.30, 1583.10)	-0.16 (-0.19, -0.14)	1408.68 (952.21, 1865.15)	1337.83 (883.00, 1792.65)	-0.16 (-0.18, -0.14)	1067.90 (710.69, 1425.12)	1029.59 (665.04, 1394.14)	-0.12 (-0.13, -0.10)
Paraguay	1397.14 (952.94, 1841.33)	1391.47 (945.27, 1837.67)	-0.00 (-0.05, 0.04)	1528.28 (1046.16, 2010.40)	1516.61 (1030.00, 2003.21)	-0.01 (-0.06, 0.03)	1229.22 (820.13, 1638.31)	1221.85 (818.09, 1625.60)	0.00 (-0.05, 0.05)
Peru	821.37 (550.39, 1092.35)	802.10 (554.61, 1049.59)	-0.06 (-0.15, 0.04)	897.61 (603.04, 1192.18)	885.06 (617.86, 1152.26)	-0.03 (-0.13, 0.08)	735.73 (487.58, 983.88)	703.78 (476.41, 931.15)	-0.12 (-0.17, -0.08)
Philippines	1176.83 (789.80, 1563.86)	1225.67 (829.22, 1622.11)	0.13 (0.09, 0.16)	1362.55 (912.62, 1812.48)	1385.98 (946.87, 1825.08)	0.05 (-0.00, 0.11)	1006.55 (675.87, 1337.23)	1004.19 (673.57, 1334.82)	-0.01 (-0.05, 0.02)
Poland	1213.78 (809.43, 1618.12)	1179.31 (797.78, 1560.83)	-0.10 (-0.12, -0.08)	1299.05 (872.70, 1725.41)	1273.17 (873.46, 1672.89)	-0.07 (-0.08, -0.05)	1040.69 (684.34, 1397.03)	1008.27 (667.71, 1348.83)	-0.10 (-0.12, -0.07)
Portugal	1327.43 (857.43, 1797.43)	1292.88 (871.70, 1714.05)	-0.09 (-0.11, -0.07)	1449.68 (946.63, 1952.73)	1428.50 (979.16, 1877.84)	-0.05 (-0.07, -0.02)	1090.28 (681.70, 1498.87)	1076.14 (702.21, 1450.07)	-0.05 (-0.07, -0.02)
Puerto Rico	994.61 (675.48, 1313.73)	951.36 (667.81, 1234.91)	-0.16 (-0.20, -0.13)	1067.83 (735.40, 1400.26)	1018.42 (724.13, 1312.70)	-0.16 (-0.20, -0.12)	901.25 (597.70, 1204.80)	858.16 (587.53, 1128.79)	-0.16 (-0.20, -0.13)
Qatar	1548.25 (998.02, 2098.48)	1397.92 (906.48, 1889.37)	-0.33 (-0.43, -0.23)	1706.75 (1116.53, 2296.96)	1545.60 (1026.09, 2065.11)	-0.31 (-0.37, -0.25)	1406.62 (890.89, 1922.35)	1251.89 (797.57, 1706.20)	-0.40 (-0.53, -0.27)
Republic of Korea	1734.58 (1113.41, 2355.74)	1493.81 (1032.73, 1954.90)	-0.48 (-0.53, -0.43)	1905.85 (1231.15, 2580.54)	1675.85 (1171.57, 2180.14)	-0.42 (-0.46, -0.38)	1260.26 (788.02, 1732.50)	1157.82 (770.37, 1545.26)	-0.26 (-0.30, -0.23)

Location	Both			Female			Male		
	1990	2021	AAPC	1990	2021	AAPC	1990	2021	AAPC
Republic of Moldova	1158.00 (765.02, 1550.98)	1112.57 (761.12, 1464.02)	-0.13 (-0.16, -0.10)	1246.67 (830.23, 1663.11)	1195.14 (822.66, 1567.61)	-0.13 (-0.16, -0.10)	983.74 (639.99, 1327.49)	955.92 (641.92, 1269.92)	-0.10 (-0.13, -0.07)
Romania	1143.93 (760.09, 1527.77)	1143.40 (770.19, 1516.61)	-0.00 (-0.02, 0.01)	1248.17 (834.44, 1661.90)	1239.97 (839.94, 1640.00)	-0.02 (-0.05, 0.01)	993.07 (655.16, 1330.98)	982.37 (652.98, 1311.76)	-0.04 (-0.06, -0.02)
Russian Federation	1212.94 (805.32, 1620.56)	1188.57 (792.54, 1584.59)	-0.07 (-0.09, -0.04)	1270.63 (849.96, 1691.29)	1268.58 (852.11, 1685.05)	-0.00 (-0.04, 0.03)	1017.43 (663.04, 1371.81)	1001.31 (661.38, 1341.25)	-0.04 (-0.07, -0.02)
Rwanda	1310.85 (836.41, 1785.29)	1472.43 (918.98, 2025.87)	0.38 (0.36, 0.41)	1532.86 (972.63, 2093.09)	1684.22 (1063.36, 2305.07)	0.31 (0.28, 0.34)	975.11 (609.32, 1340.90)	1073.09 (651.59, 1494.59)	0.31 (0.29, 0.34)
Saint Kitts and Nevis	938.94 (625.92, 1251.96)	919.10 (625.84, 1212.35)	-0.07 (-0.18, 0.03)	990.51 (668.02, 1312.99)	971.13 (666.90, 1275.36)	-0.06 (-0.22, 0.09)	838.64 (548.81, 1128.47)	820.25 (547.63, 1092.88)	-0.06 (-0.18, 0.05)
Saint Lucia	971.71 (649.76, 1293.67)	950.61 (651.78, 1249.44)	-0.07 (-0.15, 0.01)	1024.76 (692.77, 1356.76)	1027.42 (711.95, 1342.88)	0.01 (-0.09, 0.11)	879.36 (571.43, 1187.30)	840.00 (564.70, 1115.30)	-0.15 (-0.21, -0.09)
Saint Vincent and the Grenadines	1026.81 (691.56, 1362.05)	936.85 (639.69, 1234.01)	-0.30 (-0.37, -0.24)	1080.22 (730.96, 1429.48)	1016.55 (702.33, 1330.77)	-0.20 (-0.29, -0.12)	922.34 (608.70, 1235.97)	838.48 (562.22, 1114.74)	-0.33 (-0.43, -0.23)
Samoa	1432.56 (925.83, 1939.29)	1365.90 (872.56, 1859.24)	-0.15 (-0.17, -0.14)	1586.24 (1041.73, 2130.74)	1555.69 (992.65, 2118.73)	-0.06 (-0.07, -0.05)	1200.00 (750.31, 1649.69)	1129.04 (727.39, 1530.70)	-0.20 (-0.22, -0.18)
San Marino	1275.10 (863.84, 1686.35)	1148.30 (797.31, 1499.29)	-0.36 (-0.42, -0.30)	1361.96 (941.89, 1782.03)	1248.93 (871.91, 1625.96)	-0.29 (-0.35, -0.23)	1142.57 (741.77, 1543.37)	1029.07 (706.46, 1351.67)	-0.38 (-0.45, -0.31)
Sao Tome and Principe	929.05 (596.39, 1261.71)	912.78 (588.21, 1237.34)	-0.06 (-0.09, -0.03)	1043.10 (672.44, 1413.76)	1066.22 (689.77, 1442.67)	0.07 (0.01, 0.13)	697.43 (443.21, 951.64)	699.83 (445.78, 953.88)	0.01 (-0.07, 0.08)
Saudi Arabia	1496.81 (979.28, 2014.33)	1364.34 (883.40, 1845.28)	-0.31 (-0.33, -0.29)	1660.37 (1105.81, 2214.94)	1548.29 (1014.99, 2081.60)	-0.23 (-0.27, -0.20)	1343.29 (863.31, 1823.27)	1230.03 (784.88, 1675.19)	-0.28 (-0.31, -0.26)
Senegal	987.13 (629.81, 1344.45)	1043.98 (651.34, 1436.61)	0.15 (0.10, 0.20)	1161.89 (751.83, 1571.95)	1209.93 (756.99, 1662.88)	0.10 (0.07, 0.12)	794.30 (499.72, 1088.89)	839.37 (520.30, 1158.44)	0.19 (0.11, 0.28)
Serbia	1145.04 (754.08, 1536.00)	1132.05 (759.70, 1504.40)	-0.04 (-0.06, -0.02)	1238.83 (822.63, 1655.03)	1224.18 (833.98, 1614.38)	-0.04 (-0.07, -0.01)	999.70 (647.40, 1351.99)	992.83 (647.28, 1338.37)	-0.02 (-0.04, 0.01)
Seychelles	1247.27 (837.55, 1656.98)	1167.43 (787.95, 1546.90)	-0.21 (-0.23, -0.19)	1378.55 (933.04, 1824.05)	1317.50 (894.20, 1740.80)	-0.15 (-0.17, -0.13)	1010.86 (664.54, 1357.19)	925.50 (621.07, 1229.94)	-0.29 (-0.34, -0.23)
Sierra Leone	909.63 (586.09, 1233.17)	905.30 (574.77, 1235.82)	-0.01 (-0.06, 0.03)	1096.40 (704.94, 1487.85)	1076.76 (690.81, 1462.71)	-0.06 (-0.11, -0.00)	711.79 (463.25, 960.34)	711.75 (452.70, 970.81)	-0.00 (-0.04, 0.04)
Singapore	1000.21 (661.15, 1339.27)	978.92 (680.39, 1277.45)	-0.07 (-0.15, 0.01)	1104.85 (734.38, 1475.31)	1082.43 (759.56, 1405.30)	-0.07 (-0.12, -0.02)	817.72 (538.03, 1097.42)	825.22 (557.13, 1093.30)	0.04 (-0.02, 0.09)
Slovakia	1172.52 (779.93, 1565.10)	1155.07 (782.34, 1527.80)	-0.05 (-0.08, -0.01)	1266.27 (849.53, 1683.01)	1247.44 (855.86, 1639.02)	-0.05 (-0.09, 0.00)	1011.17 (660.08, 1362.27)	985.30 (656.05, 1314.54)	-0.08 (-0.11, -0.05)
Slovenia	1141.16 (764.10, 1518.22)	1152.09 (791.40, 1512.78)	0.04 (-0.00, 0.09)	1235.69 (834.19, 1637.20)	1243.08 (863.77, 1622.38)	0.02 (-0.02, 0.07)	970.50 (643.95, 1297.04)	1007.02 (672.26, 1341.79)	0.12 (0.03, 0.21)
Solomon Islands	1288.58 (823.49, 1753.67)	1288.86 (828.86, 1748.86)	-0.01 (-0.04, 0.03)	1509.88 (964.74, 2055.02)	1491.44 (964.39, 2018.49)	-0.04 (-0.09, 0.01)	1116.76 (703.33, 1530.19)	1086.64 (694.42, 1478.86)	-0.10 (-0.13, -0.06)

Location	Both			Female			Male		
	1990	2021	AAPC	1990	2021	AAPC	1990	2021	AAPC
Somalia	1241.81 (803.26, 1680.37)	1329.24 (833.85, 1824.63)	0.22 (0.19, 0.24)	1463.98 (945.36, 1982.59)	1517.39 (951.19, 2083.58)	0.12 (0.10, 0.14)	961.46 (602.53, 1320.39)	963.33 (592.94, 1333.72)	0.01 (-0.02, 0.04)
South Africa	1185.47 (797.86, 1573.09)	1214.78 (795.33, 1634.24)	0.08 (0.04, 0.12)	1369.89 (928.81, 1810.97)	1379.03 (904.58, 1853.47)	0.03 (-0.01, 0.07)	868.83 (577.86, 1159.79)	885.57 (573.83, 1197.30)	0.07 (0.04, 0.09)
South Sudan	1229.70 (795.29, 1664.11)	1234.42 (794.22, 1674.62)	0.02 (-0.01, 0.05)	1498.56 (975.96, 2021.17)	1530.33 (984.54, 2076.12)	0.08 (0.05, 0.10)	964.59 (618.37, 1310.80)	968.50 (614.60, 1322.39)	0.01 (-0.00, 0.03)
Spain	1375.55 (907.25, 1843.85)	1249.26 (848.55, 1649.97)	-0.31 (-0.36, -0.26)	1551.55 (1035.97, 2067.13)	1413.27 (977.64, 1848.90)	-0.32 (-0.36, -0.28)	1066.47 (682.90, 1450.04)	994.60 (645.43, 1343.78)	-0.23 (-0.25, -0.20)
Sri Lanka	1118.47 (761.51, 1475.43)	1102.31 (759.37, 1445.25)	-0.05 (-0.09, -0.01)	1262.98 (859.18, 1666.79)	1202.71 (840.75, 1564.67)	-0.15 (-0.19, -0.11)	978.05 (668.04, 1288.07)	945.11 (633.39, 1256.83)	-0.10 (-0.15, -0.06)
Sudan	1428.95 (925.84, 1932.06)	1321.05 (892.96, 1749.14)	-0.25 (-0.27, -0.24)	1561.25 (1035.86, 2086.65)	1472.75 (1000.51, 1944.99)	-0.19 (-0.20, -0.17)	1298.21 (824.96, 1771.47)	1196.87 (800.26, 1593.48)	-0.26 (-0.27, -0.25)
Suriname	997.14 (682.03, 1312.26)	966.07 (670.29, 1261.84)	-0.09 (-0.14, -0.04)	1072.38 (738.14, 1406.63)	1031.69 (719.59, 1343.79)	-0.10 (-0.15, -0.05)	903.24 (611.66, 1194.83)	870.04 (587.21, 1152.88)	-0.11 (-0.17, -0.05)
Sweden	1336.06 (898.79, 1773.33)	1245.67 (858.07, 1633.27)	-0.22 (-0.26, -0.18)	1487.94 (1027.52, 1948.36)	1415.58 (1001.78, 1829.38)	-0.16 (-0.19, -0.12)	1065.55 (685.57, 1445.54)	1014.51 (662.03, 1367.00)	-0.16 (-0.20, -0.11)
Switzerland	1343.21 (896.85, 1789.56)	1271.17 (871.81, 1670.53)	-0.16 (-0.22, -0.10)	1509.18 (1026.60, 1991.77)	1457.48 (1020.92, 1894.04)	-0.09 (-0.16, -0.01)	1036.94 (666.57, 1407.31)	1003.89 (658.24, 1349.53)	-0.10 (-0.16, -0.05)
Syrian Arab Republic	1440.84 (943.60, 1938.07)	1349.29 (880.21, 1818.37)	-0.21 (-0.24, -0.18)	1615.95 (1073.79, 2158.11)	1535.48 (1019.07, 2051.90)	-0.16 (-0.20, -0.12)	1288.69 (841.85, 1735.53)	1219.21 (790.28, 1648.15)	-0.18 (-0.21, -0.14)
Taiwan (Province of China)	1047.79 (684.08, 1411.50)	1111.69 (755.23, 1468.15)	0.25 (0.13, 0.37)	1222.98 (805.47, 1640.49)	1314.37 (902.91, 1725.83)	0.30 (0.20, 0.41)	829.28 (536.64, 1121.92)	848.28 (555.32, 1141.24)	0.13 (0.03, 0.23)
Tajikistan	1173.64 (793.29, 1553.98)	1170.47 (770.72, 1570.22)	-0.01 (-0.03, 0.02)	1272.84 (861.94, 1683.74)	1282.82 (842.38, 1723.26)	0.03 (-0.01, 0.07)	1016.72 (680.07, 1353.38)	1040.09 (688.65, 1391.54)	0.08 (0.05, 0.11)
Thailand	1210.15 (809.97, 1610.33)	1181.32 (817.61, 1545.02)	-0.08 (-0.13, -0.03)	1355.56 (913.20, 1797.91)	1339.54 (938.52, 1740.57)	-0.04 (-0.10, 0.01)	999.54 (669.49, 1329.59)	965.67 (653.76, 1277.58)	-0.11 (-0.16, -0.06)
Timor-Leste	1141.66 (778.88, 1504.45)	1193.88 (782.46, 1605.31)	0.15 (0.12, 0.18)	1320.67 (891.30, 1750.04)	1412.32 (936.72, 1887.93)	0.22 (0.19, 0.25)	948.32 (645.75, 1250.90)	964.52 (628.19, 1300.84)	0.06 (0.03, 0.09)
Togo	969.88 (623.08, 1316.69)	1018.21 (637.24, 1399.18)	0.15 (0.11, 0.19)	1143.80 (732.23, 1555.38)	1147.09 (724.71, 1569.47)	-0.01 (-0.05, 0.03)	749.19 (478.48, 1019.89)	774.17 (477.05, 1071.28)	0.09 (0.05, 0.14)
Tokelau	1468.23 (920.62, 2015.84)	1333.83 (871.19, 1796.48)	-0.31 (-0.34, -0.27)	1669.82 (1060.41, 2279.22)	1519.92 (996.05, 2043.79)	-0.30 (-0.32, -0.29)	1228.05 (770.42, 1685.68)	1122.60 (727.80, 1517.41)	-0.29 (-0.31, -0.27)
Tonga	1394.03 (909.07, 1879.00)	1349.26 (903.65, 1794.87)	-0.10 (-0.13, -0.07)	1590.02 (1041.94, 2138.09)	1514.02 (1016.61, 2011.43)	-0.15 (-0.19, -0.12)	1135.65 (730.17, 1541.13)	1107.94 (722.53, 1493.35)	-0.08 (-0.12, -0.03)
Trinidad and Tobago	976.56 (654.46, 1298.66)	940.43 (649.51, 1231.35)	-0.11 (-0.17, -0.05)	1040.06 (706.63, 1373.49)	1009.91 (709.45, 1310.37)	-0.09 (-0.14, -0.04)	879.67 (582.85, 1176.49)	844.57 (568.76, 1120.38)	-0.12 (-0.16, -0.09)
Tunisia	1575.51 (1050.53, 2100.49)	1451.65 (983.17, 1920.14)	-0.27 (-0.30, -0.24)	1742.86 (1182.54, 2303.18)	1589.81 (1096.34, 2083.28)	-0.31 (-0.33, -0.28)	1397.64 (921.35, 1873.94)	1282.86 (847.04, 1718.67)	-0.28 (-0.30, -0.25)

Location	Both			Female			Male		
	1990	2021	AAPC	1990	2021	AAPC	1990	2021	AAPC
Turkmenistan	1178.62 (784.01, 1573.23)	1114.95 (748.13, 1481.76)	-0.18 (-0.22, -0.15)	1261.89 (846.28, 1677.49)	1196.34 (809.00, 1583.69)	-0.18 (-0.21, -0.15)	1018.63 (669.95, 1367.32)	970.24 (635.70, 1304.77)	-0.17 (-0.21, -0.12)
Tuvalu	1431.10 (892.82, 1969.38)	1364.18 (869.32, 1859.05)	-0.15 (-0.17, -0.14)	1581.68 (996.23, 2167.13)	1534.65 (979.82, 2089.48)	-0.10 (-0.11, -0.08)	1156.89 (719.29, 1594.49)	1123.79 (708.76, 1538.82)	-0.09 (-0.11, -0.08)
Türkiye	1647.44 (1103.39, 2191.48)	1469.72 (982.37, 1957.08)	-0.35 (-0.40, -0.30)	1804.93 (1223.55, 2386.31)	1620.09 (1106.62, 2133.56)	-0.32 (-0.40, -0.25)	1418.87 (936.90, 1900.84)	1264.40 (819.36, 1709.44)	-0.37 (-0.39, -0.34)
Uganda	1177.98 (778.04, 1577.92)	1355.53 (856.49, 1854.57)	0.46 (0.42, 0.50)	1377.82 (905.42, 1850.22)	1553.05 (988.08, 2118.02)	0.40 (0.36, 0.44)	917.51 (580.10, 1254.93)	979.41 (603.34, 1355.48)	0.20 (0.17, 0.24)
Ukraine	1202.62 (797.13, 1608.10)	1183.88 (791.98, 1575.78)	-0.05 (-0.07, -0.04)	1270.99 (847.65, 1694.32)	1260.18 (859.31, 1661.05)	-0.03 (-0.05, -0.01)	1019.04 (665.81, 1372.27)	1014.68 (646.54, 1382.82)	-0.02 (-0.04, 0.01)
United Arab Emirates	1515.05 (990.30, 2039.80)	1277.01 (839.05, 1714.96)	-0.56 (-0.67, -0.45)	1687.74 (1095.77, 2279.71)	1568.30 (985.60, 2151.00)	-0.25 (-0.48, -0.02)	1360.90 (880.75, 1841.05)	1226.14 (810.92, 1641.36)	-0.32 (-0.42, -0.21)
United Kingdom	1223.10 (798.53, 1647.67)	1166.24 (776.07, 1556.41)	-0.16 (-0.19, -0.13)	1324.01 (873.74, 1774.29)	1301.39 (879.67, 1723.11)	-0.06 (-0.08, -0.03)	1002.86 (638.31, 1367.40)	973.02 (627.50, 1318.53)	-0.10 (-0.15, -0.06)
United Republic of Tanzania	1340.91 (870.95, 1810.88)	1317.51 (842.40, 1792.63)	-0.05 (-0.08, -0.03)	1637.87 (1066.06, 2209.67)	1621.35 (1040.94, 2201.77)	-0.03 (-0.05, -0.00)	961.07 (615.16, 1306.98)	907.02 (570.58, 1243.45)	-0.19 (-0.21, -0.17)
United States Virgin Islands	1009.04 (678.14, 1339.95)	903.85 (631.09, 1176.60)	-0.35 (-0.36, -0.34)	1066.49 (725.55, 1407.43)	935.24 (668.32, 1202.16)	-0.42 (-0.44, -0.41)	916.28 (599.14, 1233.41)	853.83 (569.80, 1137.87)	-0.22 (-0.24, -0.20)
United States of America	1579.65 (1064.14, 2095.17)	1522.75 (1035.36, 2010.14)	-0.13 (-0.14, -0.11)	1702.87 (1159.40, 2246.35)	1683.25 (1162.96, 2203.54)	-0.04 (-0.07, -0.01)	1362.27 (898.34, 1826.20)	1306.41 (864.28, 1748.54)	-0.14 (-0.17, -0.12)
Uruguay	1138.52 (754.55, 1522.49)	1134.52 (761.90, 1507.15)	-0.03 (-0.03, -0.02)	1278.05 (851.24, 1704.85)	1272.12 (862.50, 1681.74)	-0.02 (-0.04, -0.01)	899.25 (591.14, 1207.37)	890.87 (586.54, 1195.19)	-0.04 (-0.08, -0.00)
Uzbekistan	1130.75 (758.49, 1503.01)	1096.55 (729.19, 1463.91)	-0.10 (-0.12, -0.08)	1213.48 (819.27, 1607.69)	1178.54 (792.38, 1564.70)	-0.10 (-0.12, -0.07)	982.34 (653.13, 1311.55)	961.80 (632.75, 1290.84)	-0.07 (-0.09, -0.05)
Vanuatu	1239.44 (799.46, 1679.42)	1239.20 (786.18, 1692.23)	-0.01 (-0.05, 0.03)	1428.23 (927.99, 1928.46)	1394.96 (897.04, 1892.89)	-0.09 (-0.14, -0.04)	1073.02 (672.69, 1473.34)	1059.12 (661.52, 1456.73)	-0.04 (-0.09, 0.00)
Venezuela (Bolivarian Republic of)	1242.36 (851.73, 1632.99)	1222.28 (852.66, 1591.89)	-0.05 (-0.10, 0.00)	1341.80 (923.34, 1760.26)	1308.09 (920.80, 1695.37)	-0.08 (-0.09, -0.06)	1117.04 (759.95, 1474.13)	1096.18 (748.04, 1444.33)	-0.06 (-0.11, -0.01)
Viet Nam	1357.92 (913.41, 1802.43)	1423.20 (923.50, 1922.89)	0.16 (0.14, 0.17)	1492.26 (1014.01, 1970.51)	1571.01 (1029.36, 2112.67)	0.17 (0.15, 0.19)	1096.86 (711.98, 1481.74)	1137.86 (716.69, 1559.03)	0.12 (0.10, 0.14)
Yemen	1557.49 (1022.05, 2092.92)	1452.45 (939.78, 1965.12)	-0.22 (-0.26, -0.18)	1646.98 (1102.69, 2191.27)	1579.03 (1019.48, 2138.58)	-0.14 (-0.15, -0.12)	1406.24 (894.09, 1918.39)	1313.34 (841.34, 1785.33)	-0.22 (-0.26, -0.19)
Zambia	1184.27 (772.74, 1595.79)	1288.87 (828.69, 1749.04)	0.26 (0.22, 0.30)	1467.14 (958.14, 1976.15)	1534.16 (992.13, 2076.18)	0.14 (0.09, 0.20)	923.83 (602.63, 1245.03)	946.66 (602.24, 1291.08)	0.08 (0.06, 0.10)
Zimbabwe	1299.24 (852.88, 1745.60)	1245.99 (793.58, 1698.40)	-0.13 (-0.17, -0.08)	1504.89 (993.67, 2016.11)	1440.81 (910.56, 1971.06)	-0.14 (-0.18, -0.10)	956.32 (618.45, 1294.18)	934.10 (604.29, 1263.90)	-0.07 (-0.10, -0.05)

Notes: DALY, Disability-adjusted life year. ADRDs, Alzheimer's disease and related dementia; AAPC, Average annual percentage change.

Table S3. The age-standardized QCI-v of ADRDs and its temporal change by sex and nation, 1990-2021

Location	Both			Female			Male		
	1990	2021	AAPC	1990	2021	AAPC	1990	2021	AAPC
Afghanistan	21.86 (14.69, 29.03)	26.10 (19.32, 32.88)	0.56 (0.50, 0.63)	8.61 (1.00, 16.23)	11.98 (4.75, 19.21)	1.05 (0.79, 1.31)	44.93 (39.62, 50.24)	51.71 (46.64, 56.78)	0.42 (0.37, 0.47)
Albania	62.60 (56.93, 68.28)	66.98 (61.45, 72.50)	0.25 (0.12, 0.38)	65.81 (60.26, 71.36)	68.15 (62.64, 73.67)	0.15 (0.03, 0.28)	61.40 (56.42, 66.37)	69.20 (64.49, 73.90)	0.39 (0.31, 0.46)
Algeria	54.35 (48.39, 60.31)	56.85 (51.13, 62.58)	0.13 (0.04, 0.22)	56.81 (50.92, 62.69)	60.54 (54.79, 66.28)	0.18 (0.12, 0.24)	53.11 (47.92, 58.30)	54.50 (49.61, 59.39)	0.09 (0.04, 0.14)
American Samoa	47.92 (41.71, 54.14)	54.01 (48.05, 59.98)	0.37 (0.30, 0.45)	49.41 (43.30, 55.52)	54.81 (48.86, 60.76)	0.34 (0.28, 0.41)	53.52 (48.39, 58.66)	59.14 (54.21, 64.08)	0.30 (0.19, 0.41)
Andorra	53.90 (47.94, 59.86)	53.76 (47.77, 59.74)	0.02 (-0.09, 0.13)	59.36 (53.68, 65.04)	57.97 (52.23, 63.70)	-0.04 (-0.16, 0.08)	51.63 (46.42, 56.84)	53.62 (48.44, 58.79)	0.14 (-0.01, 0.28)
Angola	48.94 (42.87, 55.02)	23.94 (16.95, 30.94)	-2.30 (-2.41, -2.20)	55.39 (49.62, 61.16)	30.79 (24.20, 37.38)	-1.89 (-1.99, -1.79)	45.06 (39.52, 50.60)	25.53 (19.61, 31.45)	-1.79 (-2.00, -1.59)
Antigua and Barbuda	73.06 (67.72, 78.41)	75.21 (69.89, 80.53)	0.08 (0.00, 0.16)	78.29 (73.02, 83.57)	78.92 (73.70, 84.14)	0.00 (-0.08, 0.09)	65.65 (60.84, 70.47)	72.26 (67.61, 76.90)	0.31 (0.19, 0.43)
Argentina	64.87 (59.17, 70.57)	62.17 (56.50, 67.84)	-0.14 (-0.19, -0.10)	65.43 (59.78, 71.08)	62.69 (57.09, 68.29)	-0.13 (-0.19, -0.07)	70.57 (65.82, 75.33)	68.27 (63.49, 73.05)	-0.13 (-0.17, -0.09)
Armenia	71.84 (66.52, 77.15)	65.11 (59.69, 70.53)	-0.30 (-0.40, -0.20)	73.37 (68.05, 78.68)	67.56 (62.14, 72.99)	-0.24 (-0.33, -0.15)	71.46 (66.78, 76.13)	64.26 (59.56, 68.96)	-0.32 (-0.41, -0.22)
Australia	61.35 (56.01, 66.69)	47.53 (42.36, 52.71)	-0.77 (-0.89, -0.66)	66.43 (61.12, 71.74)	51.75 (46.52, 56.99)	-0.76 (-0.87, -0.65)	58.56 (53.80, 63.32)	50.19 (45.50, 54.88)	-0.49 (-0.62, -0.37)
Austria	56.77 (50.68, 62.85)	53.73 (47.79, 59.67)	-0.18 (-0.24, -0.12)	60.36 (54.47, 66.25)	57.64 (51.88, 63.40)	-0.16 (-0.22, -0.09)	58.17 (53.07, 63.28)	55.07 (49.96, 60.19)	-0.19 (-0.24, -0.14)
Azerbaijan	66.44 (60.96, 71.93)	65.73 (60.02, 71.44)	-0.03 (-0.10, 0.05)	69.51 (64.12, 74.91)	68.47 (62.86, 74.08)	-0.05 (-0.11, 0.02)	64.85 (59.94, 69.77)	64.73 (59.80, 69.66)	0.01 (-0.07, 0.09)
Bahamas	75.20 (69.93, 80.48)	75.51 (70.22, 80.80)	0.02 (-0.10, 0.13)	79.53 (74.35, 84.72)	79.97 (74.76, 85.18)	0.03 (-0.16, 0.21)	68.76 (64.02, 73.49)	69.32 (64.61, 74.04)	0.06 (-0.04, 0.16)
Bahrain	51.09 (45.01, 57.16)	62.54 (56.73, 68.35)	0.66 (0.52, 0.81)	56.04 (50.14, 61.94)	65.39 (59.71, 71.07)	0.49 (0.40, 0.59)	52.87 (47.69, 58.05)	64.60 (59.62, 69.58)	0.65 (0.55, 0.74)
Bangladesh	59.36 (53.53, 65.19)	43.24 (36.57, 49.91)	-1.07 (-1.41, -0.74)	59.63 (53.86, 65.40)	43.67 (37.15, 50.19)	-1.06 (-1.33, -0.79)	60.66 (55.71, 65.62)	47.40 (42.01, 52.80)	-0.82 (-1.05, -0.60)
Barbados	78.55 (73.35, 83.75)	73.79 (68.54, 79.04)	-0.21 (-0.33, -0.08)	83.64 (78.50, 88.77)	79.57 (74.42, 84.73)	-0.16 (-0.23, -0.09)	69.45 (64.77, 74.13)	66.50 (61.75, 71.25)	-0.17 (-0.42, 0.07)
Belarus	69.83 (64.31, 75.34)	71.13 (65.72, 76.54)	0.06 (-0.02, 0.14)	71.33 (65.87, 76.80)	73.31 (67.99, 78.64)	0.08 (0.01, 0.15)	70.77 (66.08, 75.46)	70.46 (65.70, 75.22)	0.01 (-0.09, 0.10)
Belgium	55.29 (49.59, 60.99)	51.05 (45.17, 56.93)	-0.25 (-0.40, -0.11)	59.04 (53.49, 64.59)	55.50 (49.84, 61.16)	-0.19 (-0.33, -0.06)	57.56 (52.56, 62.56)	52.17 (47.04, 57.31)	-0.32 (-0.48, -0.15)

Location	Both			Female			Male		
	1990	2021	AAPC	1990	2021	AAPC	1990	2021	AAPC
Belize	76.87 (71.79, 81.94)	74.52 (69.42, 79.63)	-0.09 (-0.19, 0.01)	83.11 (78.07, 88.14)	80.00 (74.90, 85.10)	-0.12 (-0.32, 0.08)	68.73 (64.12, 73.34)	69.20 (64.63, 73.76)	-0.01 (-0.16, 0.14)
Benin	48.47 (42.28, 54.67)	33.15 (26.64, 39.65)	-1.18 (-1.42, -0.95)	50.92 (44.82, 57.02)	37.25 (30.85, 43.65)	-0.99 (-1.18, -0.79)	52.94 (47.79, 58.08)	39.21 (33.82, 44.59)	-0.96 (-1.12, -0.79)
Bermuda	74.58 (69.28, 79.89)	75.99 (70.92, 81.05)	0.01 (-0.20, 0.23)	78.43 (73.22, 83.65)	80.14 (75.15, 85.12)	0.06 (-0.10, 0.22)	68.37 (63.57, 73.18)	70.52 (65.89, 75.14)	0.12 (-0.06, 0.31)
Bhutan	61.43 (55.64, 67.22)	24.28 (17.00, 31.57)	-2.97 (-3.10, -2.83)	66.77 (61.18, 72.36)	26.18 (19.06, 33.31)	-3.00 (-3.12, -2.89)	55.64 (50.47, 60.82)	30.91 (25.10, 36.72)	-1.89 (-1.96, -1.82)
Bolivia (Plurinational State of)	60.73 (54.90, 66.56)	57.57 (51.76, 63.37)	-0.17 (-0.19, -0.15)	66.88 (61.13, 72.63)	63.47 (57.80, 69.14)	-0.18 (-0.21, -0.14)	54.50 (49.49, 59.52)	52.83 (47.82, 57.84)	-0.11 (-0.14, -0.07)
Bosnia and Herzegovina	67.38 (61.88, 72.88)	71.26 (65.98, 76.54)	0.17 (0.10, 0.25)	69.57 (64.11, 75.03)	73.50 (68.27, 78.73)	0.17 (0.08, 0.26)	67.65 (62.86, 72.43)	70.21 (65.57, 74.84)	0.09 (0.01, 0.18)
Botswana	50.32 (44.02, 56.61)	47.68 (41.62, 53.73)	-0.19 (-0.45, 0.08)	51.42 (45.19, 57.65)	50.36 (44.36, 56.35)	-0.05 (-0.31, 0.21)	59.59 (54.56, 64.62)	57.56 (52.59, 62.53)	-0.17 (-0.30, -0.04)
Brazil	48.33 (42.65, 54.01)	51.27 (45.82, 56.73)	0.20 (0.12, 0.29)	49.27 (43.62, 54.93)	49.51 (44.02, 54.99)	0.01 (-0.07, 0.08)	54.89 (50.05, 59.73)	62.56 (57.92, 67.21)	0.43 (0.35, 0.51)
Brunei Darussalam	49.56 (43.27, 55.86)	44.53 (38.32, 50.74)	-0.36 (-0.51, -0.20)	51.32 (45.10, 57.54)	47.11 (40.96, 53.26)	-0.27 (-0.41, -0.13)	62.95 (57.98, 67.91)	57.71 (52.63, 62.79)	-0.22 (-0.45, 0.02)
Bulgaria	71.92 (66.35, 77.50)	69.91 (64.46, 75.36)	-0.09 (-0.17, -0.01)	73.19 (67.70, 78.69)	71.72 (66.34, 77.10)	-0.05 (-0.11, 0.02)	71.61 (66.82, 76.40)	70.32 (65.57, 75.08)	-0.07 (-0.13, -0.01)
Burkina Faso	22.82 (15.70, 29.94)	27.26 (20.16, 34.36)	0.58 (0.17, 1.01)	21.16 (14.01, 28.31)	30.69 (23.78, 37.61)	1.24 (0.75, 1.74)	40.33 (34.90, 45.77)	35.99 (30.38, 41.60)	-0.40 (-0.60, -0.20)
Burundi	54.54 (48.68, 60.39)	32.06 (25.19, 38.93)	-1.71 (-1.89, -1.52)	57.92 (52.14, 63.70)	37.39 (30.83, 43.94)	-1.40 (-1.46, -1.35)	56.55 (51.49, 61.61)	36.79 (31.21, 42.38)	-1.42 (-1.59, -1.25)
Cabo Verde	55.97 (50.17, 61.76)	43.31 (37.11, 49.50)	-0.81 (-0.98, -0.63)	61.69 (56.07, 67.31)	50.71 (44.79, 56.64)	-0.60 (-0.73, -0.47)	50.60 (45.57, 55.64)	37.78 (32.31, 43.24)	-0.92 (-1.17, -0.67)
Cambodia	66.88 (61.48, 72.29)	41.96 (35.68, 48.25)	-1.49 (-1.54, -1.45)	70.52 (65.18, 75.86)	47.63 (41.58, 53.68)	-1.27 (-1.30, -1.23)	63.77 (58.95, 68.59)	40.43 (34.99, 45.87)	-1.46 (-1.50, -1.41)
Cameroon	23.52 (16.45, 30.59)	25.00 (17.45, 32.55)	0.20 (-0.08, 0.47)	27.62 (20.68, 34.57)	31.00 (23.84, 38.17)	0.42 (0.16, 0.67)	29.36 (23.71, 35.00)	29.17 (23.29, 35.05)	0.01 (-0.18, 0.19)
Canada	90.31 (86.16, 94.46)	84.53 (80.32, 88.73)	-0.22 (-0.27, -0.17)	90.56 (86.26, 94.85)	85.15 (80.79, 89.51)	-0.20 (-0.25, -0.14)	90.16 (86.13, 94.19)	85.52 (81.46, 89.59)	-0.18 (-0.23, -0.13)
Central African Republic	36.06 (29.82, 42.30)	38.36 (32.23, 44.49)	0.21 (0.08, 0.34)	39.16 (33.06, 45.25)	42.40 (36.38, 48.41)	0.25 (0.11, 0.39)	47.37 (42.04, 52.69)	44.87 (39.49, 50.26)	-0.10 (-0.24, 0.05)
Chad	52.62 (46.53, 58.70)	36.14 (29.50, 42.78)	-1.21 (-1.27, -1.14)	53.92 (47.84, 60.01)	39.48 (32.96, 46.00)	-0.99 (-1.05, -0.93)	59.06 (54.05, 64.08)	40.61 (35.20, 46.02)	-1.21 (-1.25, -1.18)
Chile	68.05 (62.67, 73.43)	66.84 (61.43, 72.26)	-0.06 (-0.13, 0.01)	66.67 (61.26, 72.07)	66.42 (61.01, 71.84)	0.02 (-0.07, 0.10)	72.93 (68.30, 77.56)	71.36 (66.73, 75.98)	-0.11 (-0.21, -0.00)

Location	Both			Female			Male		
	1990	2021	AAPC	1990	2021	AAPC	1990	2021	AAPC
China	28.20 (21.40, 35.00)	60.69 (55.15, 66.23)	2.50 (2.29, 2.72)	35.13 (28.74, 41.53)	66.41 (61.12, 71.71)	1.98 (1.73, 2.23)	30.23 (24.40, 36.06)	56.61 (51.48, 61.74)	1.99 (1.81, 2.17)
Colombia	78.96 (73.84, 84.08)	78.08 (73.07, 83.09)	-0.04 (-0.08, 0.01)	80.25 (75.09, 85.40)	79.98 (74.94, 85.02)	-0.00 (-0.05, 0.05)	77.98 (73.52, 82.44)	76.12 (71.65, 80.58)	-0.07 (-0.12, -0.02)
Comoros	38.46 (32.26, 44.66)	14.98 (7.74, 22.21)	-2.96 (-3.23, -2.69)	40.17 (34.02, 46.31)	16.75 (9.67, 23.83)	-2.78 (-3.04, -2.51)	49.50 (44.42, 54.58)	27.39 (21.55, 33.22)	-1.86 (-2.00, -1.71)
Congo	19.51 (12.55, 26.48)	14.26 (8.07, 20.45)	-1.03 (-1.22, -0.84)	29.60 (23.09, 36.11)	20.89 (14.89, 26.90)	-1.15 (-1.31, -0.99)	15.16 (8.98, 21.35)	17.88 (12.23, 23.53)	0.56 (0.23, 0.89)
Cook Islands	43.89 (37.48, 50.29)	58.60 (52.89, 64.31)	0.94 (0.90, 0.98)	47.39 (41.20, 53.58)	62.25 (56.66, 67.85)	0.88 (0.84, 0.92)	47.01 (41.61, 52.41)	58.67 (53.70, 63.64)	0.72 (0.69, 0.75)
Costa Rica	76.71 (71.58, 81.84)	76.25 (71.17, 81.34)	-0.00 (-0.12, 0.11)	78.52 (73.40, 83.64)	77.99 (72.88, 83.09)	-0.01 (-0.10, 0.09)	75.12 (70.61, 79.63)	75.10 (70.60, 79.60)	-0.03 (-0.08, 0.02)
Croatia	69.72 (64.25, 75.19)	68.89 (63.44, 74.34)	-0.02 (-0.15, 0.12)	72.20 (66.76, 77.63)	71.53 (66.16, 76.90)	-0.01 (-0.14, 0.12)	68.19 (63.40, 72.98)	68.16 (63.43, 72.90)	-0.00 (-0.12, 0.12)
Cuba	71.65 (66.39, 76.90)	74.08 (68.96, 79.21)	0.09 (0.03, 0.14)	74.64 (69.42, 79.86)	78.04 (72.92, 83.17)	0.15 (0.09, 0.21)	69.19 (64.57, 73.81)	70.03 (65.41, 74.65)	0.04 (-0.03, 0.11)
Cyprus	38.15 (31.07, 45.23)	49.45 (43.03, 55.88)	0.88 (0.75, 1.02)	42.50 (35.73, 49.27)	54.31 (48.21, 60.41)	0.80 (0.68, 0.92)	40.91 (35.22, 46.59)	51.74 (46.41, 57.08)	0.79 (0.65, 0.93)
Czechia	66.76 (61.16, 72.37)	66.86 (61.43, 72.28)	-0.02 (-0.05, 0.01)	69.32 (63.79, 74.84)	69.34 (63.96, 74.71)	-0.01 (-0.05, 0.02)	67.04 (62.18, 71.90)	67.03 (62.32, 71.74)	-0.00 (-0.05, 0.05)
Côte d'Ivoire	31.47 (24.70, 38.23)	28.90 (21.75, 36.05)	-0.27 (-0.44, -0.10)	35.11 (28.49, 41.73)	33.87 (26.97, 40.76)	-0.15 (-0.34, 0.04)	39.41 (33.85, 44.97)	34.78 (29.12, 40.45)	-0.36 (-0.55, -0.17)
Democratic People's Republic of Korea	52.67 (46.44, 58.89)	45.89 (39.51, 52.26)	-0.45 (-0.52, -0.38)	56.82 (50.78, 62.85)	50.43 (44.22, 56.64)	-0.38 (-0.44, -0.32)	51.66 (46.30, 57.02)	48.76 (43.44, 54.09)	-0.18 (-0.21, -0.14)
Democratic Republic of the Congo	38.15 (31.80, 44.50)	18.66 (11.67, 25.65)	-2.30 (-2.43, -2.17)	40.78 (34.70, 46.85)	23.97 (17.25, 30.70)	-1.74 (-1.88, -1.60)	43.28 (37.85, 48.71)	27.37 (21.58, 33.15)	-1.44 (-1.56, -1.31)
Denmark	45.55 (39.46, 51.64)	14.13 (6.89, 21.36)	-3.70 (-4.33, -3.07)	52.43 (46.56, 58.30)	23.53 (16.66, 30.41)	-2.60 (-2.90, -2.29)	41.48 (36.10, 46.86)	14.21 (8.19, 20.23)	-3.34 (-3.90, -2.79)
Djibouti	38.55 (32.08, 45.01)	17.74 (10.61, 24.87)	-2.48 (-2.60, -2.37)	42.34 (36.07, 48.62)	21.37 (14.42, 28.33)	-2.19 (-2.30, -2.09)	45.66 (40.37, 50.96)	30.31 (24.72, 35.90)	-1.33 (-1.44, -1.22)
Dominica	74.69 (69.36, 80.02)	73.10 (67.75, 78.46)	-0.08 (-0.12, -0.03)	80.16 (74.91, 85.40)	78.82 (73.57, 84.07)	-0.05 (-0.09, -0.01)	64.68 (59.86, 69.51)	64.64 (59.83, 69.45)	0.01 (-0.07, 0.09)
Dominican Republic	71.88 (66.69, 77.06)	76.30 (71.33, 81.27)	0.11 (-0.01, 0.23)	76.52 (71.30, 81.74)	81.57 (76.61, 86.52)	0.18 (0.06, 0.30)	67.68 (63.07, 72.30)	69.75 (65.19, 74.31)	0.00 (-0.14, 0.14)
Ecuador	68.35 (62.82, 73.88)	69.61 (64.11, 75.11)	0.07 (-0.01, 0.14)	72.44 (66.95, 77.93)	74.49 (69.15, 79.84)	0.10 (0.03, 0.16)	64.62 (59.85, 69.39)	64.48 (59.65, 69.32)	0.02 (-0.04, 0.09)
Egypt	54.24 (48.40, 60.07)	60.69 (55.01, 66.38)	0.40 (0.20, 0.59)	58.31 (52.55, 64.07)	62.11 (56.36, 67.87)	0.18 (0.06, 0.31)	52.98 (47.95, 58.01)	59.02 (54.11, 63.92)	0.37 (0.19, 0.55)

Location	Both			Female			Male		
	1990	2021	AAPC	1990	2021	AAPC	1990	2021	AAPC
El Salvador	73.09 (67.79, 78.39)	76.26 (71.21, 81.31)	0.13 (-0.00, 0.27)	74.90 (69.61, 80.20)	78.11 (73.06, 83.16)	0.10 (-0.01, 0.21)	72.24 (67.69, 76.79)	74.71 (70.22, 79.19)	0.13 (0.02, 0.24)
Equatorial Guinea	44.05 (37.82, 50.28)	25.33 (18.50, 32.15)	-1.76 (-1.94, -1.58)	50.90 (44.94, 56.86)	31.03 (24.52, 37.54)	-1.61 (-1.78, -1.45)	39.04 (33.43, 44.65)	28.64 (22.82, 34.45)	-0.92 (-1.06, -0.77)
Eritrea	48.13 (41.92, 54.33)	10.62 (3.02, 18.21)	-4.77 (-5.00, -4.53)	50.89 (44.80, 56.99)	15.06 (7.67, 22.45)	-3.85 (-4.03, -3.68)	55.36 (50.25, 60.48)	27.92 (22.09, 33.74)	-2.17 (-2.25, -2.10)
Estonia	69.05 (63.49, 74.60)	62.56 (57.02, 68.11)	-0.30 (-0.37, -0.23)	71.03 (65.53, 76.52)	66.52 (61.10, 71.94)	-0.20 (-0.26, -0.14)	70.14 (65.38, 74.91)	59.66 (54.67, 64.66)	-0.55 (-0.61, -0.49)
Eswatini	45.40 (39.04, 51.77)	37.69 (31.05, 44.34)	-0.58 (-0.65, -0.51)	49.38 (43.22, 55.54)	40.23 (33.64, 46.81)	-0.67 (-0.74, -0.60)	50.58 (45.32, 55.84)	51.10 (45.75, 56.45)	0.06 (0.02, 0.10)
Ethiopia	31.62 (24.90, 38.34)	11.83 (4.69, 18.98)	-3.12 (-3.28, -2.97)	35.79 (29.20, 42.38)	13.52 (6.64, 20.39)	-3.08 (-3.23, -2.93)	39.39 (33.98, 44.81)	22.96 (17.27, 28.65)	-1.75 (-1.86, -1.65)
Fiji	53.36 (47.21, 59.50)	57.06 (51.22, 62.91)	0.24 (0.14, 0.35)	55.54 (49.51, 61.57)	61.30 (55.57, 67.04)	0.33 (0.23, 0.42)	59.20 (54.04, 64.36)	59.10 (54.10, 64.10)	-0.00 (-0.06, 0.06)
Finland	43.45 (37.20, 49.70)	38.22 (31.82, 44.62)	-0.39 (-0.52, -0.26)	49.83 (43.81, 55.85)	47.08 (41.07, 53.09)	-0.15 (-0.23, -0.06)	40.39 (34.83, 45.95)	33.57 (27.96, 39.19)	-0.55 (-0.76, -0.33)
France	41.38 (36.04, 46.72)	41.75 (36.28, 47.22)	0.01 (-0.10, 0.13)	43.83 (38.52, 49.15)	47.89 (42.62, 53.16)	0.28 (0.16, 0.39)	50.50 (45.61, 55.39)	41.15 (35.98, 46.33)	-0.63 (-0.70, -0.55)
Gabon	19.75 (13.14, 26.37)	15.75 (8.73, 22.78)	-0.72 (-0.96, -0.49)	29.95 (23.68, 36.22)	25.65 (19.07, 32.23)	-0.54 (-0.76, -0.32)	16.87 (10.93, 22.81)	15.19 (8.99, 21.40)	-0.20 (-0.67, 0.27)
Gambia	37.28 (30.67, 43.89)	22.89 (15.46, 30.32)	-1.62 (-2.04, -1.19)	41.32 (34.86, 47.78)	28.33 (21.20, 35.45)	-1.25 (-1.68, -0.81)	44.22 (38.93, 49.51)	28.75 (22.89, 34.61)	-1.32 (-1.74, -0.90)
Georgia	68.73 (63.26, 74.19)	65.59 (60.14, 71.04)	-0.14 (-0.30, 0.01)	71.05 (65.64, 76.45)	68.83 (63.44, 74.22)	-0.11 (-0.24, 0.03)	68.01 (63.21, 72.81)	61.95 (57.08, 66.81)	-0.29 (-0.46, -0.12)
Germany	58.77 (53.68, 63.87)	59.98 (54.95, 65.00)	0.06 (-0.05, 0.17)	60.62 (55.52, 65.73)	61.44 (56.40, 66.47)	0.03 (-0.06, 0.12)	65.23 (60.58, 69.88)	67.03 (62.53, 71.53)	0.08 (-0.00, 0.17)
Ghana	45.40 (38.98, 51.81)	24.26 (16.78, 31.73)	-1.98 (-2.12, -1.83)	45.69 (39.25, 52.13)	25.93 (18.55, 33.31)	-1.83 (-1.93, -1.73)	53.42 (48.28, 58.56)	39.19 (33.62, 44.76)	-0.96 (-1.06, -0.87)
Greece	59.64 (53.67, 65.62)	57.27 (51.41, 63.14)	-0.13 (-0.21, -0.05)	62.23 (56.37, 68.10)	59.46 (53.77, 65.15)	-0.15 (-0.22, -0.08)	60.34 (55.31, 65.37)	57.12 (52.07, 62.18)	-0.18 (-0.24, -0.12)
Greenland	65.51 (59.75, 71.27)	66.58 (61.21, 71.95)	0.02 (-0.08, 0.12)	68.89 (63.26, 74.51)	69.59 (64.29, 74.89)	-0.02 (-0.13, 0.08)	64.95 (60.00, 69.90)	66.77 (62.07, 71.48)	0.08 (0.03, 0.14)
Grenada	70.60 (65.21, 76.00)	73.49 (68.10, 78.88)	0.14 (0.01, 0.26)	71.91 (66.48, 77.35)	76.89 (71.58, 82.20)	0.10 (-0.05, 0.25)	73.55 (68.96, 78.14)	73.40 (68.63, 78.17)	-0.01 (-0.23, 0.21)
Guam	51.94 (45.82, 58.06)	73.42 (68.49, 78.35)	1.21 (1.00, 1.41)	52.69 (46.63, 58.75)	78.04 (73.20, 82.87)	1.25 (0.86, 1.64)	53.97 (48.90, 59.03)	65.79 (61.19, 70.38)	0.66 (0.27, 1.05)
Guatemala	74.34 (68.98, 79.70)	76.14 (71.02, 81.26)	0.10 (0.05, 0.14)	76.30 (70.92, 81.69)	77.76 (72.59, 82.94)	0.06 (-0.05, 0.18)	73.79 (69.14, 78.43)	75.80 (71.31, 80.29)	0.10 (0.06, 0.14)

Location	Both			Female			Male		
	1990	2021	AAPC	1990	2021	AAPC	1990	2021	AAPC
Guinea	51.69 (45.66, 57.72)	35.22 (28.57, 41.88)	-1.21 (-1.29, -1.13)	53.49 (47.42, 59.56)	38.74 (32.18, 45.30)	-0.98 (-1.09, -0.88)	54.32 (49.25, 59.38)	40.19 (34.75, 45.63)	-0.97 (-1.03, -0.90)
Guinea-Bissau	32.81 (25.94, 39.67)	23.39 (15.84, 30.93)	-1.05 (-1.23, -0.87)	38.73 (32.09, 45.36)	28.36 (21.07, 35.65)	-0.97 (-1.11, -0.82)	35.62 (29.99, 41.24)	29.49 (23.64, 35.34)	-0.57 (-0.71, -0.44)
Guyana	80.07 (74.91, 85.23)	76.94 (71.74, 82.13)	-0.15 (-0.45, 0.16)	83.71 (78.57, 88.85)	80.60 (75.48, 85.72)	-0.16 (-0.45, 0.13)	75.35 (70.77, 79.93)	72.44 (67.84, 77.04)	-0.17 (-0.51, 0.16)
Haiti	73.43 (68.03, 78.82)	69.34 (63.77, 74.92)	-0.20 (-0.22, -0.18)	79.88 (74.64, 85.13)	76.75 (71.39, 82.11)	-0.14 (-0.17, -0.11)	64.82 (59.94, 69.70)	61.98 (56.98, 66.97)	-0.15 (-0.19, -0.10)
Honduras	67.28 (61.86, 72.69)	51.98 (45.97, 57.98)	-0.83 (-0.95, -0.72)	71.47 (66.13, 76.82)	55.50 (49.58, 61.43)	-0.81 (-0.94, -0.68)	64.20 (59.48, 68.91)	52.13 (47.06, 57.20)	-0.65 (-0.74, -0.56)
Hungary	66.46 (60.86, 72.06)	68.43 (63.00, 73.85)	0.08 (0.04, 0.13)	68.96 (63.44, 74.49)	70.72 (65.36, 76.07)	0.07 (0.03, 0.11)	66.69 (61.82, 71.56)	68.40 (63.66, 73.13)	0.08 (0.04, 0.12)
Iceland	68.22 (63.09, 73.34)	60.03 (54.88, 65.18)	-0.41 (-0.50, -0.32)	66.90 (61.73, 72.08)	59.58 (54.38, 64.77)	-0.34 (-0.46, -0.22)	74.37 (69.87, 78.87)	66.00 (61.43, 70.57)	-0.38 (-0.54, -0.23)
India	67.35 (61.64, 73.07)	41.64 (35.21, 48.08)	-1.47 (-1.75, -1.19)	68.06 (62.44, 73.67)	39.71 (33.33, 46.09)	-1.65 (-2.03, -1.26)	68.49 (63.67, 73.31)	52.25 (47.15, 57.34)	-0.87 (-1.11, -0.63)
Indonesia	83.38 (78.43, 88.33)	57.65 (51.77, 63.54)	-1.18 (-1.23, -1.14)	82.21 (77.20, 87.23)	58.81 (52.96, 64.65)	-1.10 (-1.18, -1.02)	85.96 (81.66, 90.25)	62.95 (58.07, 67.83)	-1.00 (-1.06, -0.94)
Iran (Islamic Republic of)	59.43 (53.84, 65.02)	63.72 (58.28, 69.15)	0.22 (0.18, 0.26)	63.30 (57.84, 68.76)	66.50 (61.17, 71.83)	0.16 (0.12, 0.20)	58.47 (53.62, 63.32)	62.38 (57.66, 67.09)	0.20 (0.16, 0.24)
Iraq	63.25 (57.85, 68.65)	60.12 (54.50, 65.75)	-0.19 (-0.25, -0.12)	68.37 (63.08, 73.65)	65.32 (59.84, 70.81)	-0.17 (-0.23, -0.10)	59.21 (54.37, 64.04)	56.78 (51.80, 61.75)	-0.16 (-0.23, -0.09)
Ireland	56.41 (50.28, 62.55)	51.28 (45.29, 57.27)	-0.31 (-0.40, -0.22)	60.01 (54.07, 65.94)	55.92 (50.17, 61.67)	-0.22 (-0.30, -0.13)	58.03 (52.85, 63.22)	51.38 (46.21, 56.55)	-0.39 (-0.49, -0.30)
Israel	57.39 (51.27, 63.51)	51.40 (45.46, 57.35)	-0.34 (-0.45, -0.23)	59.88 (53.91, 65.84)	55.29 (49.54, 61.03)	-0.25 (-0.34, -0.15)	57.34 (52.22, 62.46)	51.91 (46.79, 57.03)	-0.32 (-0.43, -0.20)
Italy	27.75 (21.39, 34.12)	49.00 (43.03, 54.96)	1.86 (1.61, 2.12)	31.25 (25.13, 37.37)	53.19 (47.47, 58.91)	1.73 (1.52, 1.95)	39.71 (34.32, 45.10)	51.71 (46.59, 56.84)	0.91 (0.74, 1.08)
Jamaica	86.48 (81.61, 91.35)	83.77 (78.85, 88.68)	-0.11 (-0.14, -0.09)	90.21 (85.33, 95.08)	87.18 (82.28, 92.08)	-0.14 (-0.18, -0.10)	80.17 (75.77, 84.57)	79.05 (74.61, 83.50)	-0.04 (-0.10, 0.02)
Japan	40.52 (34.26, 46.77)	48.35 (42.81, 53.89)	0.83 (0.57, 1.10)	44.02 (38.02, 50.02)	53.19 (47.84, 58.54)	0.87 (0.56, 1.18)	47.61 (42.37, 52.84)	51.51 (46.52, 56.49)	0.37 (0.24, 0.50)
Jordan	61.64 (56.09, 67.18)	72.94 (67.70, 78.18)	0.53 (0.43, 0.62)	62.85 (57.27, 68.42)	73.20 (67.95, 78.46)	0.47 (0.30, 0.63)	62.79 (58.06, 67.51)	72.50 (67.92, 77.07)	0.45 (0.39, 0.51)
Kazakhstan	67.89 (62.37, 73.41)	71.38 (65.92, 76.84)	0.17 (0.08, 0.26)	69.89 (64.44, 75.35)	73.53 (68.12, 78.95)	0.17 (0.09, 0.26)	68.03 (63.25, 72.81)	70.65 (65.90, 75.40)	0.13 (0.04, 0.22)
Kenya	43.76 (37.64, 49.89)	18.47 (11.70, 25.25)	-2.73 (-2.86, -2.61)	40.73 (34.62, 46.84)	21.14 (14.54, 27.74)	-2.09 (-2.24, -1.94)	57.87 (52.97, 62.78)	39.25 (34.03, 44.46)	-1.22 (-1.38, -1.05)

Location	Both			Female			Male		
	1990	2021	AAPC	1990	2021	AAPC	1990	2021	AAPC
Kiribati	58.68<br>(52.71, 64.65)	44.70<br>(38.38, 51.01)	-0.88<br>(-0.93, -0.84)	63.20<br>(57.43, 68.98)	48.23<br>(42.08, 54.38)	-0.88<br>(-0.92, -0.84)	54.33<br>(48.95, 59.71)	47.73<br>(42.32, 53.13)	-0.41<br>(-0.44, -0.37)
Kuwait	63.30<br>(57.97, 68.63)	67.09<br>(61.82, 72.35)	0.17<br>(-0.12, 0.47)	65.96<br>(60.71, 71.21)	73.85<br>(68.85, 78.85)	0.38<br>(0.09, 0.66)	64.65<br>(59.97, 69.33)	62.92<br>(58.09, 67.76)	0.00<br>(-0.32, 0.33)
Kyrgyzstan	65.30<br>(59.80, 70.80)	70.32<br>(65.09, 75.55)	0.24<br>(0.17, 0.30)	67.74<br>(62.30, 73.19)	72.77<br>(67.57, 77.97)	0.23<br>(0.17, 0.29)	65.61<br>(60.82, 70.41)	68.82<br>(64.12, 73.53)	0.13<br>(0.05, 0.21)
Lao People's Democratic Republic	74.35<br>(69.12, 79.58)	62.79<br>(57.13, 68.45)	-0.55<br>(-0.61, -0.49)	75.06<br>(69.77, 80.34)	63.76<br>(58.11, 69.41)	-0.54<br>(-0.59, -0.48)	76.67<br>(72.04, 81.30)	66.69<br>(61.82, 71.56)	-0.46<br>(-0.49, -0.42)
Latvia	68.95<br>(63.43, 74.46)	69.49<br>(64.08, 74.91)	-0.01<br>(-0.05, 0.04)	71.02<br>(65.57, 76.47)	72.33<br>(66.97, 77.69)	0.04<br>(-0.00, 0.08)	68.94<br>(64.16, 73.72)	67.72<br>(62.95, 72.49)	-0.05<br>(-0.13, 0.03)
Lebanon	72.97<br>(67.73, 78.21)	76.61<br>(71.61, 81.61)	0.15<br>(0.07, 0.22)	71.75<br>(66.47, 77.03)	77.75<br>(72.76, 82.75)	0.25<br>(0.17, 0.33)	78.12<br>(73.61, 82.63)	76.17<br>(71.67, 80.68)	-0.10<br>(-0.19, -0.02)
Lesotho	57.14<br>(51.39, 62.90)	46.86<br>(40.44, 53.28)	-0.63<br>(-0.73, -0.53)	59.96<br>(54.27, 65.64)	48.90<br>(42.58, 55.22)	-0.66<br>(-0.75, -0.57)	61.80<br>(56.88, 66.72)	61.74<br>(56.82, 66.66)	0.02<br>(-0.06, 0.10)
Liberia	38.92<br>(32.15, 45.69)	33.50<br>(26.62, 40.38)	-0.42<br>(-0.65, -0.20)	42.24<br>(35.67, 48.81)	35.88<br>(29.08, 42.69)	-0.47<br>(-0.76, -0.18)	42.64<br>(37.15, 48.13)	38.25<br>(32.72, 43.79)	-0.36<br>(-0.49, -0.22)
Libya	46.34<br>(40.62, 52.07)	51.00<br>(45.07, 56.92)	0.33<br>(0.13, 0.54)	51.26<br>(45.71, 56.81)	55.42<br>(49.68, 61.16)	0.27<br>(0.07, 0.47)	45.84<br>(40.78, 50.91)	50.67<br>(45.47, 55.88)	0.33<br>(0.03, 0.64)
Lithuania	67.65<br>(62.10, 73.19)	66.27<br>(60.74, 71.79)	-0.05<br>(-0.11, 0.00)	69.18<br>(63.73, 74.63)	69.09<br>(63.65, 74.54)	0.01<br>(-0.05, 0.08)	68.30<br>(63.50, 73.10)	65.53<br>(60.72, 70.35)	-0.12<br>(-0.18, -0.06)
Luxembourg	69.77<br>(63.96, 75.58)	54.47<br>(48.49, 60.45)	-0.80<br>(-0.92, -0.68)	72.80<br>(67.13, 78.48)	60.45<br>(54.70, 66.20)	-0.58<br>(-0.72, -0.44)	67.74<br>(62.88, 72.60)	50.97<br>(45.78, 56.17)	-0.91<br>(-1.09, -0.73)
Madagascar	68.74<br>(63.29, 74.18)	58.87<br>(53.16, 64.58)	-0.50<br>(-0.56, -0.45)	67.85<br>(62.37, 73.32)	58.52<br>(52.80, 64.25)	-0.48<br>(-0.53, -0.43)	73.30<br>(68.69, 77.91)	66.36<br>(61.60, 71.11)	-0.34<br>(-0.39, -0.28)
Malawi	43.41<br>(37.09, 49.73)	26.56<br>(19.56, 33.56)	-1.59<br>(-1.83, -1.34)	44.76<br>(38.48, 51.05)	31.31<br>(24.58, 38.04)	-1.22<br>(-1.40, -1.03)	50.34<br>(45.09, 55.59)	37.21<br>(31.63, 42.80)	-0.97<br>(-1.18, -0.76)
Malaysia	57.53<br>(52.03, 63.03)	47.74<br>(41.68, 53.80)	-0.62<br>(-0.89, -0.35)	59.91<br>(54.47, 65.35)	47.98<br>(41.96, 54.00)	-0.69<br>(-0.85, -0.53)	59.43<br>(54.61, 64.24)	52.21<br>(47.15, 57.28)	-0.42<br>(-0.62, -0.22)
Maldives	77.59<br>(72.51, 82.66)	73.41<br>(68.30, 78.53)	-0.14<br>(-0.17, -0.11)	79.07<br>(74.01, 84.13)	73.51<br>(68.33, 78.69)	-0.19<br>(-0.23, -0.14)	77.50<br>(73.00, 81.99)	75.38<br>(70.87, 79.89)	-0.08<br>(-0.13, -0.03)
Mali	17.87<br>(10.54, 25.19)	11.23<br>(3.54, 18.92)	-1.54<br>(-2.14, -0.93)	20.44<br>(13.30, 27.57)	11.82<br>(4.25, 19.38)	-1.79<br>(-2.20, -1.37)	27.48<br>(21.73, 33.23)	21.24<br>(15.27, 27.21)	-0.85<br>(-1.20, -0.50)
Malta	58.94<br>(52.96, 64.92)	53.43<br>(47.54, 59.32)	-0.24<br>(-0.41, -0.08)	62.10<br>(56.27, 67.93)	56.00<br>(50.23, 61.77)	-0.18<br>(-0.29, -0.08)	59.73<br>(54.68, 64.78)	57.10<br>(52.09, 62.11)	-0.06<br>(-0.33, 0.20)
Marshall Islands	35.20<br>(28.30, 42.10)	38.81<br>(32.02, 45.61)	0.32<br>(0.26, 0.37)	41.76<br>(35.20, 48.31)	46.71<br>(40.29, 53.14)	0.36<br>(0.29, 0.43)	35.02<br>(29.19, 40.84)	35.47<br>(29.72, 41.22)	0.04<br>(-0.01, 0.09)
Mauritania	41.63<br>(35.18, 48.08)	29.32<br>(22.13, 36.51)	-1.08<br>(-1.29, -0.87)	44.05<br>(37.60, 50.50)	31.62<br>(24.51, 38.72)	-1.00<br>(-1.28, -0.72)	47.10<br>(41.85, 52.36)	34.12<br>(28.42, 39.82)	-1.03<br>(-1.25, -0.81)

Location	Both			Female			Male		
	1990	2021	AAPC	1990	2021	AAPC	1990	2021	AAPC
Mauritius	62.82 (57.27, 68.38)	68.42 (63.12, 73.73)	0.27 (0.04, 0.50)	65.19 (59.69, 70.68)	68.84 (63.48, 74.19)	0.18 (-0.08, 0.44)	66.01 (61.20, 70.81)	73.67 (69.13, 78.21)	0.37 (0.22, 0.52)
Mexico	84.20 (79.07, 89.32)	78.60 (73.28, 83.93)	-0.20 (-0.23, -0.18)	84.36 (79.21, 89.52)	79.50 (74.14, 84.87)	-0.16 (-0.19, -0.14)	82.01 (77.63, 86.38)	77.41 (72.95, 81.87)	-0.19 (-0.22, -0.16)
Micronesia (Federated States of)	45.75 (39.37, 52.13)	48.50 (42.22, 54.79)	0.19 (0.13, 0.25)	50.78 (44.66, 56.91)	53.62 (47.57, 59.67)	0.17 (0.12, 0.22)	44.85 (39.36, 50.34)	47.13 (41.79, 52.47)	0.16 (0.11, 0.21)
Monaco	55.20 (48.98, 61.42)	40.23 (33.83, 46.63)	-1.01 (-1.07, -0.96)	58.42 (52.41, 64.43)	44.10 (37.97, 50.23)	-0.90 (-0.95, -0.85)	57.05 (51.83, 62.27)	43.99 (38.60, 49.38)	-0.83 (-0.91, -0.74)
Mongolia	60.03 (54.28, 65.77)	65.48 (59.90, 71.06)	0.27 (0.10, 0.44)	63.53 (57.86, 69.21)	69.59 (64.11, 75.08)	0.33 (0.13, 0.52)	58.91 (53.92, 63.89)	61.01 (56.08, 65.94)	0.12 (0.01, 0.23)
Montenegro	69.03 (63.60, 74.45)	67.35 (61.83, 72.87)	-0.10 (-0.16, -0.03)	71.94 (66.58, 77.30)	69.24 (63.73, 74.75)	-0.13 (-0.21, -0.06)	66.79 (62.01, 71.57)	67.76 (63.00, 72.52)	0.03 (-0.04, 0.10)
Morocco	62.86 (57.37, 68.35)	56.11 (50.35, 61.87)	-0.36 (-0.39, -0.34)	65.93 (60.55, 71.30)	61.25 (55.59, 66.91)	-0.23 (-0.25, -0.21)	62.36 (57.52, 67.20)	53.50 (48.46, 58.53)	-0.50 (-0.53, -0.47)
Mozambique	32.48 (25.81, 39.16)	13.56 (5.83, 21.29)	-2.79 (-3.24, -2.33)	39.29 (32.91, 45.66)	23.12 (15.98, 30.26)	-1.78 (-2.05, -1.51)	33.37 (27.65, 39.10)	15.20 (8.82, 21.58)	-2.52 (-2.97, -2.06)
Myanmar	82.02 (77.08, 86.96)	63.85 (58.25, 69.45)	-0.81 (-0.84, -0.79)	78.87 (73.74, 84.00)	64.74 (59.17, 70.31)	-0.64 (-0.68, -0.61)	91.02 (86.81, 95.23)	69.37 (64.63, 74.11)	-0.87 (-0.89, -0.85)
Namibia	55.70 (49.88, 61.53)	37.38 (30.65, 44.10)	-1.30 (-1.37, -1.22)	56.95 (51.14, 62.75)	41.46 (34.96, 47.95)	-1.04 (-1.11, -0.97)	63.56 (58.67, 68.44)	44.88 (39.50, 50.26)	-1.11 (-1.17, -1.06)
Nauru	40.21 (33.50, 46.91)	45.19 (38.63, 51.75)	0.37 (0.31, 0.42)	37.58 (30.89, 44.26)	48.06 (41.63, 54.49)	0.78 (0.73, 0.84)	45.88 (40.49, 51.27)	47.37 (41.98, 52.76)	0.10 (0.07, 0.13)
Nepal	78.83 (73.59, 84.08)	46.16 (39.74, 52.58)	-1.74 (-1.82, -1.66)	77.81 (72.50, 83.12)	46.51 (40.15, 52.86)	-1.68 (-1.75, -1.60)	80.84 (76.35, 85.33)	52.24 (47.01, 57.46)	-1.42 (-1.48, -1.35)
Netherlands	47.78 (42.36, 53.21)	46.29 (40.60, 51.98)	-0.10 (-0.20, 0.00)	53.30 (47.96, 58.64)	53.00 (47.51, 58.50)	-0.02 (-0.07, 0.03)	45.49 (40.46, 50.52)	44.28 (39.08, 49.48)	-0.08 (-0.25, 0.08)
New Zealand	56.57 (50.78, 62.36)	55.53 (49.80, 61.26)	-0.04 (-0.18, 0.11)	60.76 (55.16, 66.36)	59.45 (53.88, 65.02)	-0.01 (-0.13, 0.12)	57.87 (52.87, 62.88)	56.15 (51.14, 61.16)	-0.09 (-0.26, 0.07)
Nicaragua	79.41 (74.41, 84.42)	81.37 (76.49, 86.25)	0.09 (0.00, 0.17)	80.99 (75.96, 86.03)	82.50 (77.60, 87.40)	0.07 (-0.02, 0.16)	77.74 (73.29, 82.20)	80.83 (76.44, 85.22)	0.14 (0.06, 0.23)
Niger	41.51 (35.10, 47.93)	29.77 (22.74, 36.80)	-1.11 (-1.31, -0.91)	46.76 (40.53, 52.99)	34.63 (27.95, 41.31)	-0.99 (-1.13, -0.86)	45.52 (40.23, 50.81)	31.16 (25.43, 36.89)	-1.25 (-1.38, -1.11)
Nigeria	38.43 (31.72, 45.14)	12.41 (4.64, 20.19)	-3.49 (-3.81, -3.17)	46.24 (39.80, 52.68)	14.95 (7.51, 22.39)	-3.60 (-3.96, -3.24)	36.60 (31.08, 42.12)	21.85 (15.57, 28.12)	-1.54 (-1.72, -1.36)
Niue	30.00 (22.99, 37.00)	40.56 (34.05, 47.07)	1.00 (0.89, 1.11)	35.47 (28.81, 42.12)	46.39 (40.15, 52.62)	0.87 (0.82, 0.92)	32.78 (27.03, 38.54)	39.99 (34.45, 45.53)	0.64 (0.56, 0.72)
North Macedonia	72.85 (67.47, 78.24)	70.13 (64.62, 75.64)	-0.11 (-0.13, -0.09)	75.24 (69.88, 80.61)	71.93 (66.44, 77.42)	-0.14 (-0.18, -0.10)	70.81 (66.08, 75.55)	70.72 (65.97, 75.47)	-0.00 (-0.07, 0.06)

Location	Both			Female			Male		
	1990	2021	AAPC	1990	2021	AAPC	1990	2021	AAPC
Northern Mariana Islands	50.79 (44.62, 56.97)	51.75 (45.64, 57.87)	0.22 (-0.03, 0.48)	53.51 (47.47, 59.56)	56.50 (50.61, 62.39)	0.33 (0.05, 0.60)	52.93 (47.72, 58.15)	51.10 (45.86, 56.35)	-0.01 (-0.16, 0.14)
Norway	61.32 (55.60, 67.04)	48.88 (42.72, 55.03)	-0.71 (-0.78, -0.64)	64.52 (58.98, 70.06)	53.94 (48.04, 59.85)	-0.58 (-0.63, -0.52)	61.35 (56.40, 66.30)	49.11 (43.91, 54.31)	-0.72 (-0.79, -0.65)
Oman	53.50 (47.71, 59.30)	53.96 (47.95, 59.97)	-0.01 (-0.24, 0.21)	60.62 (55.10, 66.13)	61.39 (55.71, 67.08)	0.05 (-0.08, 0.18)	46.76 (41.54, 51.99)	47.47 (41.98, 52.96)	0.03 (-0.22, 0.28)
Pakistan	49.53 (43.17, 55.90)	24.17 (16.97, 31.37)	-2.25 (-2.39, -2.10)	41.31 (34.75, 47.87)	15.41 (8.00, 22.81)	-3.09 (-3.45, -2.74)	59.51 (54.51, 64.52)	41.64 (36.19, 47.10)	-1.14 (-1.22, -1.05)
Palau	40.58 (33.87, 47.29)	51.81 (45.35, 58.26)	0.79 (0.72, 0.85)	43.85 (37.41, 50.29)	51.88 (45.45, 58.31)	0.54 (0.49, 0.60)	44.71 (39.27, 50.15)	54.06 (48.88, 59.25)	0.60 (0.49, 0.72)
Palestine	63.21 (57.56, 68.87)	67.16 (61.69, 72.63)	0.20 (0.18, 0.23)	66.91 (61.38, 72.43)	70.65 (65.25, 76.04)	0.18 (0.14, 0.21)	62.62 (57.65, 67.60)	66.46 (61.64, 71.29)	0.19 (0.14, 0.24)
Panama	78.61 (73.53, 83.69)	77.05 (72.06, 82.04)	-0.08 (-0.16, -0.01)	80.61 (75.51, 85.71)	78.48 (73.49, 83.48)	-0.14 (-0.19, -0.10)	76.79 (72.33, 81.26)	76.40 (71.98, 80.82)	-0.03 (-0.12, 0.06)
Papua New Guinea	68.24 (62.76, 73.73)	65.53 (59.78, 71.27)	-0.13 (-0.15, -0.12)	72.17 (66.78, 77.57)	70.60 (65.01, 76.18)	-0.07 (-0.08, -0.05)	65.41 (60.62, 70.20)	61.97 (56.98, 66.97)	-0.18 (-0.22, -0.14)
Paraguay	57.82 (52.33, 63.31)	47.97 (42.30, 53.65)	-0.66 (-0.87, -0.44)	55.21 (49.64, 60.79)	46.59 (40.86, 52.32)	-0.58 (-0.81, -0.36)	67.35 (62.71, 72.00)	58.49 (53.69, 63.29)	-0.47 (-0.57, -0.38)
Peru	63.38 (57.73, 69.03)	65.57 (60.19, 70.95)	0.09 (-0.09, 0.26)	69.01 (63.44, 74.58)	70.81 (65.49, 76.14)	0.05 (-0.13, 0.23)	58.30 (53.44, 63.16)	60.51 (55.77, 65.25)	0.04 (-0.05, 0.13)
Philippines	74.28 (69.06, 79.51)	63.18 (57.72, 68.63)	-0.51 (-0.60, -0.42)	73.86 (68.58, 79.14)	63.36 (57.95, 68.78)	-0.49 (-0.59, -0.38)	75.12 (70.61, 79.63)	66.19 (61.54, 70.85)	-0.42 (-0.48, -0.36)
Poland	68.52 (62.99, 74.05)	64.78 (59.25, 70.32)	-0.18 (-0.21, -0.15)	70.77 (65.32, 76.23)	67.54 (62.13, 72.95)	-0.14 (-0.19, -0.10)	68.30 (63.55, 73.05)	64.55 (59.76, 69.34)	-0.17 (-0.21, -0.13)
Portugal	51.80 (45.42, 58.19)	53.13 (47.22, 59.05)	0.08 (-0.01, 0.17)	55.83 (49.68, 61.98)	56.83 (51.12, 62.54)	0.05 (-0.03, 0.13)	54.03 (48.68, 59.38)	54.38 (49.27, 59.49)	0.02 (-0.05, 0.08)
Puerto Rico	74.34 (69.12, 79.56)	77.08 (72.04, 82.11)	0.12 (0.03, 0.20)	79.07 (73.93, 84.20)	81.55 (76.57, 86.52)	0.10 (0.02, 0.17)	68.56 (63.85, 73.26)	70.86 (66.28, 75.43)	0.11 (0.03, 0.19)
Qatar	44.22 (37.97, 50.47)	55.38 (49.41, 61.35)	0.83 (0.49, 1.16)	52.03 (46.09, 57.97)	60.88 (55.19, 66.57)	0.50 (0.29, 0.70)	44.05 (38.61, 49.48)	56.57 (51.42, 61.71)	0.82 (0.43, 1.20)
Republic of Korea	22.59 (15.92, 29.26)	46.86 (41.52, 52.21)	2.41 (2.20, 2.62)	28.10 (21.63, 34.57)	47.33 (41.96, 52.70)	1.71 (1.47, 1.96)	36.36 (30.89, 41.84)	63.15 (58.47, 67.82)	1.81 (1.73, 1.90)
Republic of Moldova	65.21 (59.56, 70.87)	73.46 (68.25, 78.68)	0.39 (0.34, 0.43)	67.14 (61.51, 72.76)	75.29 (70.08, 80.49)	0.39 (0.32, 0.46)	66.69 (61.83, 71.56)	72.41 (67.79, 77.04)	0.28 (0.22, 0.34)
Romania	68.26 (62.67, 73.85)	68.57 (63.08, 74.05)	0.02 (-0.02, 0.06)	69.84 (64.31, 75.37)	70.42 (65.00, 75.83)	0.03 (-0.01, 0.06)	68.47 (63.70, 73.24)	68.77 (64.05, 73.50)	0.02 (-0.02, 0.05)
Russian Federation	66.81 (61.23, 72.40)	67.50 (62.01, 73.00)	0.03 (-0.01, 0.08)	69.30 (63.82, 74.78)	69.61 (64.19, 75.03)	0.02 (-0.02, 0.06)	66.72 (61.91, 71.54)	68.07 (63.37, 72.77)	0.06 (0.03, 0.09)

Location	Both			Female			Male		
	1990	2021	AAPC	1990	2021	AAPC	1990	2021	AAPC
Rwanda	40.65 (34.15, 47.16)	18.19 (10.96, 25.43)	-2.58 (-2.75, -2.41)	45.96 (39.59, 52.32)	25.34 (18.48, 32.19)	-1.93 (-2.06, -1.79)	42.92 (37.45, 48.39)	22.00 (15.97, 28.03)	-2.14 (-2.35, -1.93)
Saint Kitts and Nevis	73.12 (67.60, 78.64)	74.55 (69.28, 79.82)	0.05 (-0.16, 0.26)	76.92 (71.47, 82.38)	79.03 (73.82, 84.25)	0.11 (-0.12, 0.33)	68.62 (63.82, 73.41)	69.56 (64.85, 74.26)	0.04 (-0.13, 0.21)
Saint Lucia	74.97 (69.58, 80.36)	73.32 (68.06, 78.57)	-0.08 (-0.22, 0.06)	79.40 (74.09, 84.71)	76.53 (71.32, 81.75)	-0.13 (-0.30, 0.03)	68.64 (63.83, 73.46)	71.14 (66.50, 75.78)	0.14 (0.04, 0.24)
Saint Vincent and the Grenadines	71.33 (66.00, 76.67)	76.17 (70.96, 81.39)	0.20 (0.07, 0.32)	76.12 (70.77, 81.46)	79.55 (74.40, 84.70)	0.14 (0.01, 0.26)	66.19 (61.47, 70.91)	73.60 (69.00, 78.20)	0.34 (0.09, 0.60)
Samoa	31.53 (24.77, 38.28)	33.23 (26.31, 40.15)	0.18 (0.10, 0.27)	38.52 (32.18, 44.87)	36.92 (30.17, 43.67)	-0.17 (-0.25, -0.08)	30.70 (24.92, 36.49)	37.65 (32.16, 43.13)	0.65 (0.57, 0.74)
San Marino	65.40 (59.88, 70.92)	62.39 (56.80, 67.98)	-0.08 (-0.31, 0.14)	68.57 (63.16, 73.98)	64.72 (59.23, 70.22)	-0.18 (-0.44, 0.07)	64.41 (59.48, 69.33)	62.47 (57.73, 67.22)	0.02 (-0.18, 0.22)
Sao Tome and Principe	42.60 (36.08, 49.12)	32.61 (25.74, 39.48)	-0.83 (-0.92, -0.75)	47.26 (40.93, 53.58)	34.57 (27.84, 41.30)	-1.01 (-1.08, -0.93)	49.39 (44.11, 54.67)	41.23 (35.76, 46.70)	-0.53 (-0.69, -0.37)
Saudi Arabia	46.66 (40.44, 52.87)	51.22 (45.06, 57.38)	0.30 (0.20, 0.39)	52.54 (46.64, 58.44)	56.48 (50.57, 62.39)	0.23 (0.13, 0.32)	46.55 (41.26, 51.84)	51.61 (46.40, 56.83)	0.32 (0.24, 0.40)
Senegal	38.75 (32.22, 45.27)	15.15 (7.41, 22.88)	-2.94 (-3.18, -2.70)	43.67 (37.36, 49.98)	22.18 (14.84, 29.53)	-2.03 (-2.29, -1.76)	40.90 (35.40, 46.39)	17.52 (11.41, 23.64)	-2.63 (-3.01, -2.25)
Serbia	67.50 (61.85, 73.16)	69.34 (63.83, 74.85)	0.09 (0.03, 0.15)	69.55 (63.99, 75.10)	71.78 (66.32, 77.23)	0.10 (0.06, 0.15)	67.68 (62.79, 72.58)	68.43 (63.63, 73.23)	0.03 (-0.04, 0.10)
Seychelles	62.48 (56.94, 68.03)	64.77 (59.29, 70.25)	0.12 (0.06, 0.18)	65.80 (60.36, 71.23)	66.50 (61.07, 71.92)	0.04 (-0.01, 0.09)	62.54 (57.65, 67.44)	69.68 (64.96, 74.39)	0.34 (0.17, 0.51)
Sierra Leone	54.55 (48.44, 60.66)	46.46 (39.98, 52.94)	-0.52 (-0.60, -0.44)	55.64 (49.58, 61.69)	48.02 (41.69, 54.35)	-0.46 (-0.58, -0.34)	59.68 (54.71, 64.66)	51.43 (46.17, 56.68)	-0.49 (-0.58, -0.40)
Singapore	56.17 (50.90, 61.44)	65.24 (60.56, 69.92)	0.50 (0.28, 0.72)	57.87 (52.53, 63.22)	67.34 (62.59, 72.10)	0.50 (0.31, 0.69)	63.09 (58.44, 67.73)	67.81 (63.42, 72.19)	0.24 (0.06, 0.42)
Slovakia	65.38 (59.76, 71.00)	65.27 (59.82, 70.73)	0.01 (-0.05, 0.06)	67.72 (62.19, 73.26)	67.73 (62.35, 73.11)	-0.01 (-0.08, 0.07)	65.63 (60.76, 70.49)	65.86 (61.10, 70.63)	-0.01 (-0.08, 0.07)
Slovenia	66.71 (61.15, 72.27)	65.02 (59.63, 70.41)	-0.10 (-0.20, -0.00)	68.30 (62.83, 73.78)	68.90 (63.60, 74.21)	0.03 (-0.06, 0.12)	67.43 (62.66, 72.20)	62.81 (57.99, 67.63)	-0.23 (-0.39, -0.07)
Solomon Islands	52.77 (46.48, 59.06)	51.93 (45.70, 58.16)	-0.03 (-0.18, 0.12)	56.05 (49.89, 62.21)	53.83 (47.72, 59.93)	-0.12 (-0.27, 0.02)	53.59 (48.31, 58.88)	54.07 (48.86, 59.28)	0.02 (-0.09, 0.14)
Somalia	51.07 (45.03, 57.11)	39.12 (32.44, 45.81)	-0.85 (-0.94, -0.75)	55.90 (49.94, 61.87)	43.48 (36.98, 49.97)	-0.80 (-0.87, -0.73)	49.86 (44.54, 55.17)	43.02 (37.44, 48.59)	-0.47 (-0.56, -0.39)
South Africa	64.01 (58.48, 69.53)	50.27 (44.19, 56.34)	-0.78 (-0.92, -0.64)	65.44 (59.98, 70.90)	53.13 (47.17, 59.08)	-0.68 (-0.83, -0.53)	67.66 (62.94, 72.37)	56.85 (51.90, 61.80)	-0.56 (-0.63, -0.49)
South Sudan	47.66 (41.54, 53.77)	35.27 (28.75, 41.80)	-0.95 (-1.02, -0.88)	52.90 (46.97, 58.83)	38.54 (32.15, 44.94)	-1.00 (-1.09, -0.91)	50.92 (45.73, 56.12)	39.22 (33.74, 44.70)	-0.83 (-0.92, -0.75)

Location	Both			Female			Male		
	1990	2021	AAPC	1990	2021	AAPC	1990	2021	AAPC
Spain	47.26 (42.06, 52.45)	37.35 (31.34, 43.37)	-1.15 (-1.67, -0.63)	51.11 (45.86, 56.36)	42.46 (36.70, 48.21)	-0.91 (-1.33, -0.48)	50.03 (45.24, 54.82)	40.52 (35.17, 45.87)	-0.96 (-1.30, -0.62)
Sri Lanka	75.04 (69.96, 80.12)	71.21 (66.04, 76.37)	-0.17 (-0.25, -0.09)	73.43 (68.26, 78.60)	72.55 (67.41, 77.68)	-0.03 (-0.12, 0.05)	78.97 (74.53, 83.42)	73.28 (68.65, 77.91)	-0.27 (-0.33, -0.20)
Sudan	65.75 (60.18, 71.33)	65.77 (60.33, 71.21)	-0.00 (-0.03, 0.03)	71.00 (65.62, 76.38)	69.76 (64.40, 75.12)	-0.06 (-0.08, -0.04)	62.02 (57.04, 67.00)	63.82 (59.06, 68.58)	0.09 (0.05, 0.12)
Suriname	80.00 (74.93, 85.07)	77.02 (71.95, 82.10)	-0.14 (-0.24, -0.05)	83.63 (78.55, 88.70)	81.30 (76.24, 86.36)	-0.11 (-0.22, -0.01)	75.50 (71.00, 79.99)	71.37 (66.72, 76.02)	-0.21 (-0.34, -0.09)
Sweden	69.20 (63.88, 74.51)	66.78 (61.29, 72.27)	-0.11 (-0.22, -0.01)	76.08 (71.01, 81.16)	73.45 (68.25, 78.65)	-0.12 (-0.19, -0.04)	57.93 (52.97, 62.90)	58.89 (53.84, 63.94)	0.06 (-0.05, 0.18)
Switzerland	60.07 (54.36, 65.78)	50.04 (44.19, 55.89)	-0.62 (-0.85, -0.40)	63.29 (57.73, 68.84)	53.41 (47.75, 59.07)	-0.52 (-0.74, -0.30)	61.24 (56.22, 66.27)	53.51 (48.41, 58.61)	-0.41 (-0.56, -0.27)
Syrian Arab Republic	69.68 (64.23, 75.13)	66.71 (61.06, 72.35)	-0.14 (-0.20, -0.09)	71.36 (65.99, 76.72)	67.82 (62.26, 73.38)	-0.17 (-0.22, -0.12)	69.41 (64.66, 74.15)	64.96 (60.09, 69.83)	-0.22 (-0.27, -0.16)
Taiwan (Province of China)	45.42 (39.29, 51.54)	50.36 (44.94, 55.77)	0.19 (-0.25, 0.63)	50.83 (44.90, 56.76)	54.79 (49.45, 60.14)	0.12 (-0.27, 0.51)	45.84 (40.66, 51.03)	48.73 (43.78, 53.68)	0.10 (-0.29, 0.50)
Tajikistan	65.02 (59.56, 70.48)	54.43 (48.50, 60.35)	-0.60 (-0.70, -0.49)	67.14 (61.71, 72.58)	58.36 (52.48, 64.23)	-0.49 (-0.62, -0.36)	64.92 (60.18, 69.66)	53.95 (48.95, 58.95)	-0.61 (-0.67, -0.55)
Thailand	49.66 (43.98, 55.34)	54.82 (49.37, 60.27)	0.37 (0.20, 0.54)	53.72 (48.15, 59.28)	57.36 (51.97, 62.75)	0.30 (0.12, 0.48)	50.30 (45.40, 55.20)	57.15 (52.38, 61.91)	0.43 (0.29, 0.57)
Timor-Leste	82.51 (77.58, 87.45)	66.30 (60.79, 71.81)	-0.69 (-0.72, -0.66)	80.48 (75.38, 85.58)	63.38 (57.82, 68.93)	-0.78 (-0.82, -0.73)	86.77 (82.45, 91.09)	74.10 (69.42, 78.77)	-0.50 (-0.53, -0.47)
Togo	39.37 (32.75, 45.99)	24.74 (17.40, 32.07)	-1.41 (-1.60, -1.21)	42.30 (35.81, 48.79)	30.76 (23.78, 37.74)	-0.96 (-1.17, -0.75)	45.01 (39.67, 50.35)	28.66 (22.78, 34.55)	-1.42 (-1.60, -1.24)
Tokelau	26.68 (19.45, 33.91)	38.95 (32.44, 45.46)	1.24 (1.15, 1.32)	31.14 (24.25, 38.03)	42.53 (36.24, 48.82)	1.01 (0.95, 1.07)	30.22 (24.43, 36.01)	40.35 (34.96, 45.75)	0.93 (0.89, 0.98)
Tonga	42.62 (36.30, 48.94)	41.71 (35.54, 47.88)	-0.06 (-0.17, 0.05)	45.19 (39.03, 51.35)	46.08 (40.01, 52.15)	0.05 (-0.10, 0.19)	48.23 (42.96, 53.51)	44.62 (39.38, 49.86)	-0.27 (-0.42, -0.12)
Trinidad and Tobago	76.69 (71.39, 81.99)	78.52 (73.41, 83.64)	0.06 (-0.03, 0.16)	80.97 (75.75, 86.19)	82.52 (77.46, 87.59)	0.05 (-0.05, 0.14)	71.55 (66.88, 76.23)	73.36 (68.80, 77.93)	0.06 (-0.00, 0.13)
Tunisia	61.54 (56.07, 67.00)	62.92 (57.54, 68.31)	0.08 (0.01, 0.15)	66.09 (60.80, 71.39)	67.74 (62.49, 72.98)	0.08 (0.02, 0.15)	59.93 (55.07, 64.78)	59.55 (54.70, 64.40)	-0.00 (-0.12, 0.12)
Turkmenistan	65.33 (59.75, 70.91)	66.25 (60.67, 71.83)	0.04 (-0.04, 0.12)	67.47 (61.93, 73.00)	69.14 (63.63, 74.65)	0.08 (-0.00, 0.16)	65.34 (60.53, 70.14)	64.47 (59.58, 69.36)	-0.03 (-0.14, 0.08)
Tuvalu	39.85 (32.99, 46.70)	42.23 (35.62, 48.84)	0.19 (0.11, 0.26)	43.81 (37.23, 50.39)	45.86 (39.42, 52.30)	0.14 (0.07, 0.20)	42.52 (36.94, 48.09)	44.43 (38.96, 49.90)	0.14 (0.10, 0.18)
Türkiye	59.31 (53.82, 64.80)	66.73 (61.31, 72.14)	0.38 (0.27, 0.48)	62.00 (56.57, 67.43)	69.08 (63.79, 74.37)	0.35 (0.19, 0.50)	61.15 (56.36, 65.93)	66.55 (61.75, 71.35)	0.27 (0.21, 0.32)

Location	Both			Female			Male		
	1990	2021	AAPC	1990	2021	AAPC	1990	2021	AAPC
Uganda	52.57 (46.73, 58.41)	26.31 (19.38, 33.24)	-2.20 (-2.35, -2.04)	59.18 (53.42, 64.95)	33.38 (26.75, 40.01)	-1.85 (-1.95, -1.75)	49.34 (44.06, 54.62)	31.26 (25.52, 37.00)	-1.49 (-1.65, -1.33)
Ukraine	67.90 (62.20, 73.60)	66.17 (60.50, 71.85)	-0.06 (-0.10, -0.02)	70.42 (64.82, 76.01)	69.35 (63.85, 74.86)	-0.04 (-0.09, -0.00)	66.83 (61.98, 71.69)	63.70 (58.64, 68.76)	-0.13 (-0.21, -0.06)
United Arab Emirates	40.91 (34.55, 47.27)	49.82 (43.61, 56.02)	0.55 (0.07, 1.02)	45.10 (38.90, 51.29)	49.55 (43.15, 55.95)	0.17 (-0.48, 0.82)	42.86 (37.53, 48.18)	50.63 (45.52, 55.74)	0.46 (0.12, 0.80)
United Kingdom	53.96 (47.82, 60.10)	51.65 (45.59, 57.72)	-0.11 (-0.21, -0.01)	58.17 (52.27, 64.07)	56.66 (50.86, 62.46)	-0.08 (-0.15, -0.02)	54.57 (49.40, 59.75)	50.80 (45.61, 55.98)	-0.17 (-0.39, 0.06)
United Republic of Tanzania	36.12 (29.75, 42.48)	24.34 (17.88, 30.81)	-1.27 (-1.39, -1.14)	37.46 (31.14, 43.78)	26.23 (19.84, 32.61)	-1.16 (-1.25, -1.06)	48.17 (42.98, 53.35)	39.25 (33.91, 44.60)	-0.68 (-0.78, -0.58)
United States Virgin Islands	68.81 (63.32, 74.29)	78.66 (73.55, 83.78)	0.44 (0.40, 0.48)	74.08 (68.72, 79.44)	86.43 (81.50, 91.36)	0.49 (0.45, 0.53)	61.32 (56.39, 66.24)	65.24 (60.44, 70.04)	0.20 (0.12, 0.28)
United States of America	53.33 (47.71, 58.95)	49.20 (43.60, 54.79)	-0.26 (-0.33, -0.19)	55.67 (50.17, 61.18)	54.63 (49.26, 60.00)	-0.07 (-0.12, -0.01)	56.96 (52.07, 61.85)	48.02 (43.01, 53.03)	-0.55 (-0.60, -0.50)
Uruguay	66.52 (60.90, 72.14)	58.40 (52.63, 64.16)	-0.40 (-0.51, -0.29)	66.90 (61.31, 72.49)	59.30 (53.63, 64.97)	-0.35 (-0.44, -0.26)	71.89 (67.18, 76.61)	64.92 (60.08, 69.76)	-0.33 (-0.41, -0.25)
Uzbekistan	63.51 (57.91, 69.11)	67.23 (61.66, 72.80)	0.19 (0.14, 0.24)	66.04 (60.52, 71.56)	70.34 (64.83, 75.84)	0.22 (0.19, 0.25)	62.93 (58.10, 67.77)	65.23 (60.37, 70.08)	0.13 (0.08, 0.17)
Vanuatu	49.83 (43.44, 56.23)	46.33 (39.79, 52.88)	-0.21 (-0.32, -0.11)	54.84 (48.69, 61.00)	52.08 (45.85, 58.31)	-0.15 (-0.26, -0.05)	48.70 (43.25, 54.15)	44.20 (38.65, 49.76)	-0.31 (-0.38, -0.24)
Venezuela (Bolivarian Republic of)	75.73 (70.65, 80.80)	75.41 (70.39, 80.44)	-0.04 (-0.12, 0.03)	76.76 (71.64, 81.88)	77.59 (72.59, 82.59)	0.03 (-0.05, 0.12)	76.04 (71.59, 80.49)	74.07 (69.56, 78.59)	-0.11 (-0.18, -0.04)
Viet Nam	53.64 (47.94, 59.34)	34.75 (28.29, 41.21)	-1.43 (-1.51, -1.35)	56.89 (51.28, 62.49)	40.26 (34.04, 46.48)	-1.13 (-1.18, -1.07)	56.19 (51.14, 61.25)	38.14 (32.58, 43.71)	-1.25 (-1.37, -1.13)
Yemen	65.51 (60.05, 70.97)	60.92 (55.06, 66.78)	-0.23 (-0.30, -0.16)	71.16 (65.89, 76.44)	66.60 (60.90, 72.31)	-0.22 (-0.26, -0.18)	58.22 (53.17, 63.27)	56.74 (51.63, 61.84)	-0.10 (-0.19, -0.02)
Zambia	48.85 (42.75, 54.95)	35.54 (29.04, 42.03)	-1.01 (-1.14, -0.88)	51.68 (45.64, 57.72)	38.69 (32.33, 45.05)	-0.93 (-1.07, -0.78)	52.46 (47.39, 57.53)	42.72 (37.37, 48.07)	-0.66 (-0.72, -0.60)
Zimbabwe	40.32 (33.97, 46.67)	40.82 (34.29, 47.35)	0.03 (-0.17, 0.23)	44.50 (38.33, 50.68)	42.74 (36.26, 49.21)	-0.14 (-0.33, 0.04)	47.55 (42.34, 52.76)	47.80 (42.63, 52.97)	0.04 (-0.07, 0.14)

Notes: QCI-v, Quality of care index based on variational autoencoders; ADRDs, Alzheimer's disease and related dementia; AAPC, Average annual percentage change.

Table S4. The year trends of slope index of inequality for burden of ADRDs by sex, SDI quantiles and WHO regions, from 1990 to 2021

Sex	Year	Location											
		Global	High SDI	High-middle SDI	Middle SDI	Low-middle SDI	Low SDI	AFR	EMR	EUR	AMR	SEAR	WPR
Both													
	1990	136.93 (36.83, 237.02)	69.63 (-33.58, 172.85)	43.25 (-128.93, 215.42)	56.54 (-129.79, 242.87)	-55.92 (-228.18, 116.34)	327.29 (129.07, 525.50)	158.55 (-76.17, 393.27)	109.65 (7.51, 211.79)	198.03 (118.59, 277.47)	101.20 (-106.83, 309.22)	208.50 (-7.18, 424.17)	51.29 (-63.62, 166.21)
	1991	142.08 (44.23, 239.93)	69.82 (-39.29, 178.93)	62.05 (-87.59, 211.69)	59.90 (-189.22, 309.01)	-295.31 (-646.47, 55.85)	115.79 (-19.88, 251.46)	158.57 (-75.47, 392.60)	107.52 (0.15, 214.90)	194.45 (115.40, 273.50)	104.56 (-103.44, 312.56)	267.75 (77.27, 458.24)	45.89 (-70.79, 162.56)
	1992	135.22 (39.63, 230.82)	67.02 (-61.57, 195.62)	-20.44 (-217.56, 176.69)	-58.73 (-265.27, 147.82)	-192.39 (-412.69, 27.92)	109.97 (-26.80, 246.75)	163.53 (-73.77, 400.83)	94.18 (-6.22, 194.57)	189.51 (106.32, 272.70)	107.15 (-115.47, 329.76)	268.40 (72.20, 464.60)	15.96 (-93.90, 125.81)
	1993	125.66 (30.67, 220.65)	56.53 (-238.17, 351.23)	76.98 (-152.58, 306.55)	-80.18 (-268.98, 108.62)	-149.35 (-330.39, 31.68)	115.79 (-26.85, 258.44)	191.19 (-44.67, 427.05)	86.99 (-8.87, 182.86)	191.30 (112.38, 270.21)	112.63 (-109.55, 334.82)	261.29 (49.30, 473.28)	27.18 (-80.22, 134.58)
	1994	123.01 (28.61, 217.41)	22.40 (-194.43, 239.22)	96.18 (-139.37, 331.74)	-30.60 (-218.49, 157.28)	-124.75 (-299.36, 49.85)	139.98 (-3.53, 283.48)	188.07 (-51.27, 427.41)	83.17 (-11.19, 177.53)	193.39 (119.89, 266.89)	137.26 (-93.27, 367.79)	262.50 (51.80, 473.20)	23.72 (-86.14, 133.59)
	1995	125.82 (30.44, 221.21)	55.69 (-235.95, 347.33)	162.97 (-84.44, 410.38)	107.06 (-74.92, 289.04)	-110.83 (-269.38, 47.71)	115.53 (-39.86, 270.92)	183.71 (-61.43, 428.85)	66.73 (-35.93, 169.38)	204.04 (130.79, 277.29)	128.45 (-92.41, 349.31)	267.86 (49.28, 486.44)	20.20 (-88.54, 128.95)
	1996	123.89 (29.96, 217.82)	-11.17 (-289.49, 267.15)	203.40 (-59.32, 466.12)	81.31 (-73.27, 235.89)	-44.89 (-193.11, 103.33)	144.26 (-14.07, 302.59)	201.53 (-41.48, 444.55)	68.35 (-27.85, 164.56)	196.17 (116.64, 275.71)	121.97 (-97.33, 341.27)	267.33 (46.05, 488.61)	-4.70 (-108.66, 99.25)
	1997	122.23 (27.86, 216.60)	-10.47 (-290.57, 269.63)	162.77 (-87.81, 413.36)	70.58 (-103.15, 244.31)	-10.90 (-156.50, 134.69)	170.42 (11.05, 329.78)	192.64 (-51.54, 436.82)	73.66 (-19.61, 166.93)	197.57 (120.65, 274.49)	114.23 (-103.41, 331.87)	267.24 (51.01, 483.46)	-6.45 (-110.29, 97.40)
	1998	116.85 (22.62, 211.08)	261.27 (-6.13, 528.67)	259.61 (14.72, 504.51)	-25.82 (-198.39, 146.75)	-64.43 (-213.68, 84.82)	144.91 (-13.61, 303.42)	193.71 (-54.93, 442.36)	74.16 (-17.92, 166.25)	191.62 (112.94, 270.30)	113.00 (-99.76, 325.76)	261.48 (36.97, 485.98)	-12.47 (-117.82, 92.89)
	1999	120.02 (26.80, 213.24)	114.74 (-123.54, 353.02)	278.76 (17.46, 540.05)	-21.77 (-218.72, 175.19)	-48.51 (-185.15, 88.13)	185.31 (12.36, 358.26)	198.25 (-54.48, 450.98)	74.51 (-5.92, 154.93)	185.33 (106.32, 264.35)	102.84 (-107.37, 313.04)	254.02 (22.73, 485.30)	-11.29 (-113.78, 91.21)
	2000	109.81 (17.22, 202.40)	123.32 (-118.04, 364.68)	222.18 (-39.38, 483.75)	-173.89 (-397.19, 49.41)	-69.60 (-205.31, 66.10)	158.15 (-22.43, 338.74)	200.13 (-41.74, 442.00)	79.71 (-0.67, 160.08)	186.61 (108.38, 264.84)	89.68 (-117.74, 297.09)	250.18 (9.25, 491.12)	-3.21 (-110.13, 103.72)
	2001	111.35 (19.43, 203.27)	11.25 (-215.63, 238.12)	316.17 (32.53, 599.80)	-87.28 (-330.73, 156.16)	-65.44 (-202.66, 71.77)	166.79 (-13.43, 347.01)	211.01 (-43.72, 465.74)	81.94 (0.60, 163.27)	184.77 (107.35, 262.19)	87.40 (-125.94, 300.73)	244.34 (30.74, 457.95)	-5.16 (-109.58, 99.25)
	2002	104.80 (14.58, 195.03)	7.08 (-184.72, 198.87)	463.31 (131.49, 795.13)	-86.94 (-301.25, 127.36)	-98.27 (-255.50, 58.96)	86.49 (-99.92, 272.91)	209.37 (-42.04, 460.77)	74.96 (-8.29, 158.20)	180.78 (107.05, 254.52)	61.73 (-143.36, 266.83)	244.59 (29.45, 459.74)	-6.18 (-108.86, 96.51)
	2003	100.59 (10.61, 190.56)	7.51 (-195.08, 210.09)	451.28 (107.76, 794.81)	-55.64 (-264.72, 153.43)	-115.90 (-271.58, 39.77)	63.79 (-137.09, 264.67)	216.39 (-40.80, 473.57)	73.95 (-11.51, 159.41)	174.71 (104.35, 245.06)	17.02 (-175.81, 209.86)	237.74 (13.55, 461.92)	-7.59 (-114.20, 99.02)
	2004	91.43 (3.89, 178.96)	-55.60 (-274.67, 163.47)	199.96 (-66.86, 466.78)	-113.39 (-306.30, 79.52)	-110.18 (-264.21, 43.85)	25.30 (-188.38, 238.99)	206.30 (-50.89, 463.49)	63.49 (-19.25, 146.23)	164.57 (93.41, 235.74)	11.09 (-179.34, 201.53)	241.10 (14.80, 467.40)	-12.53 (-122.80, 97.74)
	2005	94.05 (7.21, 180.89)	5.54 (-156.05, 167.13)	191.72 (-99.45, 482.88)	-131.37 (-317.33, 54.59)	-102.66 (-273.87, 68.56)	-21.10 (-250.23, 208.04)	223.69 (-41.63, 489.00)	59.11 (-25.37, 143.59)	164.55 (96.85, 232.25)	-4.15 (-189.24, 180.94)	240.88 (12.87, 468.88)	-16.53 (-122.31, 89.24)
	2006	88.86 (2.59, 175.13)	8.14 (-105.15, 121.43)	182.26 (-145.77, 510.29)	-106.52 (-345.78, 132.74)	-126.88 (-307.92, 54.17)	-29.51 (-258.34, 199.33)	223.48 (-33.17, 480.14)	51.09 (-31.19, 133.37)	165.15 (98.55, 231.76)	37.24 (-160.23, 234.72)	237.24 (6.85, 467.63)	-23.23 (-125.95, 79.48)

Sex	Year	Location											
		**Global**	**High SDI**	**High-middle SDI**	**Middle SDI**	**Low-middle SDI**	**Low SDI**	**AFR**	**EMR**	**EUR**	**AMR**	**SEAR**	**WPR**
	2007	88.35 (4.49, 172.21)	22.69 (-127.55, 172.94)	231.63 (-79.28, 542.53)	-10.07 (-276.43, 256.29)	-129.04 (-354.58, 96.51)	-65.41 (-312.15, 181.34)	225.42 (-39.25, 490.09)	49.15 (-31.67, 129.97)	161.01 (93.74, 228.28)	51.06 (-140.27, 242.39)	230.12 (-7.42, 467.66)	-27.10 (-128.47, 74.27)
	2008	80.68 (-4.41, 165.78)	68.76 (-176.89, 314.41)	90.31 (-88.17, 268.79)	80.10 (-208.61, 368.80)	-159.51 (-392.24, 73.22)	-17.52 (-287.21, 252.17)	221.93 (-34.05, 477.91)	44.68 (-39.43, 128.79)	155.29 (86.28, 224.31)	47.85 (-140.52, 236.22)	224.23 (-16.11, 464.58)	-19.73 (-124.04, 84.57)
	2009	82.53 (-0.82, 165.88)	1.87 (-224.56, 228.29)	104.13 (-52.12, 260.39)	-11.35 (-176.22, 153.52)	-263.28 (-484.71, -41.85)	-134.84 (-486.35, 216.66)	208.32 (-48.72, 465.36)	39.87 (-37.59, 117.33)	154.55 (86.32, 222.77)	36.67 (-146.39, 219.73)	215.88 (-27.65, 459.41)	-25.82 (-129.93, 78.28)
	2010	81.99 (-1.49, 165.46)	-1.92 (-199.64, 195.79)	138.27 (-8.27, 284.81)	43.10 (-102.51, 188.71)	-306.53 (-742.79, 129.72)	2.74 (-220.60, 226.09)	196.04 (-63.06, 455.15)	26.61 (-50.49, 103.71)	154.53 (88.25, 220.81)	34.42 (-142.59, 211.43)	208.39 (-8.13, 424.91)	-26.91 (-131.19, 77.37)
	2011	76.15 (-8.38, 160.69)	31.95 (-139.34, 203.23)	141.59 (-7.35, 290.52)	83.81 (-52.19, 219.81)	19.78 (-386.72, 426.28)	-21.96 (-247.92, 204.00)	193.69 (-66.43, 453.80)	14.53 (-59.94, 88.99)	158.21 (90.99, 225.44)	86.79 (-106.38, 279.95)	204.03 (-14.24, 422.31)	-27.11 (-129.52, 75.30)
	2012	74.47 (-8.93, 157.86)	36.10 (-95.44, 167.65)	20.99 (-147.31, 189.29)	105.08 (-34.24, 244.40)	214.81 (-106.75, 536.38)	34.55 (-192.78, 261.88)	192.50 (-64.06, 449.05)	8.53 (-61.57, 78.63)	165.79 (101.18, 230.41)	84.03 (-106.97, 275.04)	206.15 (-22.35, 434.65)	-26.85 (-130.11, 76.41)
	2013	66.52 (-16.06, 149.09)	66.18 (-69.61, 201.98)	29.70 (-155.88, 215.29)	101.92 (-20.49, 224.33)	49.26 (-215.63, 314.15)	-34.39 (-298.01, 229.23)	192.27 (-61.31, 445.85)	7.03 (-68.89, 82.95)	162.57 (97.74, 227.41)	65.00 (-114.70, 244.69)	203.82 (-25.28, 432.91)	-25.03 (-129.25, 79.20)
	2014	59.55 (-22.84, 141.94)	47.67 (-86.39, 181.74)	-46.36 (-241.63, 148.91)	99.67 (-55.13, 254.47)	11.88 (-212.72, 236.48)	-36.23 (-309.35, 236.89)	201.83 (-52.76, 456.41)	0.45 (-77.05, 77.96)	158.70 (91.73, 225.68)	62.82 (-115.93, 241.58)	204.40 (-23.61, 432.40)	-26.57 (-132.14, 78.99)
	2015	54.47 (-27.38, 136.32)	28.11 (-103.29, 159.51)	-106.48 (-269.38, 56.42)	72.46 (-85.70, 230.63)	11.51 (-169.59, 192.60)	-90.72 (-368.79, 187.36)	200.66 (-54.98, 456.30)	-9.44 (-82.15, 63.27)	150.98 (84.92, 217.04)	3.20 (-156.16, 162.56)	199.07 (-3.19, 401.32)	-29.00 (-132.74, 74.74)
	2016	54.63 (-27.82, 137.08)	73.16 (-56.14, 202.46)	-54.74 (-245.27, 135.79)	75.48 (-89.38, 240.34)	85.33 (-108.05, 278.70)	-54.58 (-312.07, 202.91)	201.02 (-49.29, 451.32)	-8.11 (-82.86, 66.64)	145.48 (75.92, 215.04)	-0.30 (-162.06, 161.47)	183.74 (-16.84, 384.32)	-30.65 (-132.45, 71.14)
	2017	52.06 (-30.63, 134.75)	96.22 (-45.51, 237.96)	16.31 (-197.73, 230.35)	72.51 (-95.71, 240.73)	160.77 (-12.90, 334.44)	62.06 (-189.46, 313.58)	195.67 (-54.91, 446.25)	-15.13 (-90.62, 60.36)	137.58 (72.42, 202.74)	-1.09 (-165.94, 163.75)	178.24 (-18.27, 374.74)	-32.02 (-134.76, 70.72)
	2018	48.07 (-35.23, 131.38)	121.73 (-27.56, 271.01)	51.82 (-94.00, 197.64)	70.79 (-192.23, 333.82)	175.03 (5.38, 344.68)	41.65 (-235.85, 319.14)	201.63 (-48.70, 451.96)	-24.92 (-96.18, 46.33)	135.63 (72.29, 198.98)	-8.01 (-171.83, 155.82)	174.41 (-15.95, 364.76)	-37.79 (-140.07, 64.49)
	2019	40.09 (-42.59, 122.77)	122.96 (-25.03, 270.96)	26.74 (-114.61, 168.09)	23.84 (-358.61, 406.30)	128.55 (-52.65, 309.75)	29.05 (-257.95, 316.05)	179.44 (-69.67, 428.55)	-35.54 (-107.89, 36.81)	127.55 (63.98, 191.12)	-7.10 (-176.07, 161.86)	171.34 (-18.01, 360.68)	-41.44 (-143.38, 60.51)
	2020	37.30 (-45.98, 120.58)	98.95 (-47.58, 245.48)	-372.26 (-803.62, 59.09)	-135.16 (-357.15, 86.82)	73.88 (-78.64, 226.41)	118.11 (-190.64, 426.86)	180.24 (-68.24, 428.72)	-33.38 (-118.00, 51.24)	116.34 (53.53, 179.16)	0.54 (-169.46, 170.55)	95.94 (-113.45, 305.32)	-52.46 (-154.84, 49.93)
	2021	36.48 (-47.49, 120.45)	101.20 (-37.02, 239.42)	-103.67 (-320.63, 113.29)	-121.72 (-311.97, 68.53)	79.26 (-97.87, 256.39)	132.93 (-173.78, 439.63)	166.06 (-81.35, 413.47)	-26.68 (-105.58, 52.22)	110.61 (48.51, 172.70)	-1.13 (-171.23, 168.96)	95.46 (-112.04, 302.96)	-48.99 (-150.73, 52.75)
Male													
	1990	188.74 (114.99, 262.48)	-88.01 (-242.31, 66.30)	82.14 (-77.94, 242.23)	43.88 (-92.66, 180.43)	-67.56 (-192.83, 57.72)	372.16 (207.64, 536.69)	96.48 (-60.51, 253.47)	80.66 (-20.45, 181.77)	135.34 (74.33, 196.35)	-20.93 (-138.20, 96.33)	139.95 (-46.68, 326.59)	-12.85 (-105.64, 79.94)
	1991	190.92 (115.94, 265.89)	-85.58 (-236.19, 65.03)	79.51 (-61.28, 220.31)	30.57 (-134.56, 195.70)	-257.31 (-549.46, 34.84)	153.86 (47.11, 260.61)	96.59 (-58.26, 251.44)	82.64 (-32.69, 197.96)	134.04 (72.05, 196.04)	-15.61 (-143.91, 112.70)	181.76 (53.80, 309.73)	-16.17 (-109.05, 76.70)
	1992	185.27 (110.25, 260.29)	-84.91 (-235.47, 65.64)	34.07 (-112.71, 180.86)	-45.41 (-175.81, 84.99)	-145.56 (-317.49, 26.37)	155.56 (46.86, 264.26)	99.69 (-55.90, 255.29)	77.22 (-38.88, 193.32)	128.90 (64.37, 193.43)	0.09 (-137.63, 137.81)	183.01 (56.92, 309.10)	-39.87 (-127.90, 48.15)

Sex	Year	Location											
		Global	High SDI	High-middle SDI	Middle SDI	Low-middle SDI	Low SDI	AFR	EMR	EUR	AMR	SEAR	WPR
	1993	177.71 (102.70, 252.73)	-103.40 (-207.77, 0.98)	51.69 (-149.33, 252.72)	-54.81 (-153.62, 44.01)	-117.37 (-266.87, 32.13)	165.72 (52.07, 279.37)	120.84 (-39.26, 280.95)	75.64 (-29.52, 180.81)	133.08 (73.35, 192.81)	9.27 (-128.02, 146.55)	179.88 (59.85, 299.91)	-40.70 (-127.28, 45.89)
	1994	173.25 (97.33, 249.17)	-118.53 (-209.17, -27.89)	46.40 (-141.02, 233.82)	-39.32 (-160.09, 81.44)	-96.69 (-238.97, 45.59)	182.15 (67.40, 296.89)	118.40 (-40.34, 277.14)	74.17 (-9.95, 158.28)	129.94 (69.14, 190.74)	13.33 (-118.46, 145.12)	179.11 (41.54, 316.67)	-42.37 (-127.61, 42.86)
	1995	175.07 (99.68, 250.47)	21.46 (-192.33, 235.26)	135.03 (-65.63, 335.70)	57.66 (-42.70, 158.03)	-79.95 (-211.94, 52.04)	172.34 (54.36, 290.32)	115.93 (-44.82, 276.68)	66.13 (-18.34, 150.60)	134.75 (74.69, 194.82)	13.91 (-127.58, 155.39)	191.66 (94.93, 288.40)	-42.39 (-128.01, 43.22)
	1996	174.85 (100.35, 249.35)	21.37 (-174.65, 217.40)	178.96 (-42.56, 400.47)	73.93 (-45.40, 193.27)	-32.09 (-160.35, 96.16)	194.21 (72.85, 315.56)	128.25 (-38.67, 295.17)	76.69 (-6.44, 159.83)	132.44 (69.04, 195.85)	9.97 (-130.77, 150.70)	190.98 (106.09, 275.87)	-63.31 (-135.72, 9.10)
	1997	173.53 (99.16, 247.89)	21.77 (-174.65, 218.19)	129.68 (-69.10, 328.46)	56.41 (-77.52, 190.33)	-6.52 (-129.90, 116.86)	214.59 (102.66, 326.53)	116.51 (-46.07, 279.09)	86.22 (-3.31, 175.75)	133.98 (72.30, 195.66)	-0.69 (-135.70, 134.32)	187.72 (112.30, 263.14)	-64.34 (-139.33, 10.65)
	1998	169.25 (95.10, 243.39)	177.83 (-50.67, 406.32)	210.92 (-2.33, 424.17)	-33.85 (-142.04, 74.33)	-52.10 (-177.65, 73.45)	199.82 (83.94, 315.71)	114.87 (-47.09, 276.83)	80.66 (9.31, 152.00)	129.68 (69.42, 189.93)	1.67 (-136.43, 139.77)	187.03 (108.66, 265.40)	-74.96 (-142.41, -7.51)
	1999	170.90 (98.56, 243.23)	81.46 (-96.46, 259.37)	218.38 (-5.27, 442.03)	1.97 (-127.70, 131.63)	-24.55 (-147.41, 98.31)	222.92 (101.51, 344.33)	112.81 (-47.30, 272.92)	99.07 (28.19, 169.96)	127.03 (67.30, 186.75)	-26.21 (-141.01, 88.58)	181.32 (117.32, 245.32)	-74.04 (-145.20, -2.89)
	2000	163.91 (90.61, 237.21)	86.20 (-91.99, 264.40)	176.20 (-40.06, 392.45)	-72.28 (-208.23, 63.68)	-42.90 (-159.97, 74.16)	191.92 (67.82, 316.01)	108.84 (-51.68, 269.37)	95.64 (37.13, 154.15)	128.49 (69.67, 187.30)	-36.59 (-146.24, 73.06)	181.54 (117.42, 245.66)	-74.31 (-144.21, -4.42)
	2001	166.25 (93.39, 239.12)	29.92 (-140.37, 200.20)	219.50 (-1.98, 440.98)	-28.64 (-197.02, 139.75)	-38.08 (-159.68, 83.53)	193.77 (70.27, 317.26)	119.14 (-42.07, 280.35)	99.73 (29.81, 169.64)	129.16 (73.08, 185.23)	-39.64 (-152.53, 73.24)	176.31 (106.92, 245.69)	-75.21 (-146.59, -3.82)
	2002	161.81 (89.31, 234.32)	4.13 (-125.16, 133.42)	265.67 (25.31, 506.04)	-40.65 (-189.50, 108.20)	-53.73 (-168.09, 60.64)	122.95 (-8.18, 254.07)	115.96 (-42.49, 274.41)	100.64 (15.92, 185.37)	126.11 (69.02, 183.21)	-52.09 (-157.44, 53.26)	177.47 (97.97, 256.96)	-75.52 (-146.63, -4.42)
	2003	157.96 (85.62, 230.31)	3.35 (-122.56, 129.26)	257.63 (17.29, 497.97)	-24.34 (-152.78, 104.11)	-62.89 (-174.81, 49.03)	110.73 (-34.20, 255.66)	118.83 (-48.97, 286.64)	97.55 (6.23, 188.87)	121.39 (62.77, 180.01)	-70.00 (-168.64, 28.64)	171.33 (72.03, 270.63)	-73.99 (-145.97, -2.01)
	2004	153.74 (82.34, 225.14)	-24.88 (-144.58, 94.83)	190.82 (-9.11, 390.75)	-25.32 (-161.97, 111.34)	-60.80 (-169.61, 48.01)	97.09 (-54.95, 249.13)	112.92 (-50.03, 275.87)	92.37 (6.06, 178.67)	116.51 (61.59, 171.43)	-72.49 (-171.26, 26.28)	171.68 (63.38, 279.97)	-74.45 (-143.91, -4.98)
	2005	155.63 (85.12, 226.14)	-1.93 (-90.40, 86.55)	174.82 (-34.08, 383.73)	-55.11 (-195.68, 85.45)	-58.71 (-184.67, 67.25)	61.85 (-99.78, 223.48)	124.18 (-51.73, 300.09)	89.82 (5.37, 174.27)	114.40 (57.78, 171.02)	-77.36 (-180.39, 25.66)	170.30 (64.33, 276.27)	-74.78 (-142.66, -6.90)
	2006	150.72 (80.15, 221.29)	-13.98 (-104.42, 76.47)	123.01 (-107.13, 353.15)	-54.74 (-263.15, 153.67)	-87.69 (-224.48, 49.10)	51.86 (-111.37, 215.08)	120.41 (-48.98, 289.80)	92.48 (11.78, 173.17)	113.86 (58.07, 169.65)	-54.23 (-160.74, 52.27)	164.88 (51.66, 278.09)	-78.24 (-145.98, -10.49)
	2007	150.73 (79.73, 221.72)	5.73 (-101.01, 112.48)	125.65 (-101.62, 352.93)	11.69 (-193.19, 216.58)	-81.85 (-249.40, 85.69)	45.92 (-129.35, 221.20)	120.92 (-54.30, 296.13)	93.18 (13.07, 173.28)	110.39 (54.52, 166.26)	-49.41 (-141.52, 42.69)	154.95 (27.47, 282.42)	-79.33 (-152.01, -6.64)
	2008	144.12 (73.78, 214.47)	53.24 (-137.87, 244.35)	47.33 (-85.03, 179.70)	8.18 (-226.05, 242.41)	-67.95 (-237.66, 101.76)	126.77 (-60.26, 313.79)	116.41 (-54.55, 287.38)	95.60 (14.54, 176.65)	108.84 (50.48, 167.20)	-48.62 (-144.96, 47.71)	152.13 (37.56, 266.69)	-69.72 (-149.68, 10.24)
	2009	148.07 (77.30, 218.84)	20.46 (-141.68, 182.60)	49.07 (-58.16, 156.29)	-9.58 (-145.25, 126.09)	-163.79 (-332.59, 5.01)	27.03 (-214.31, 268.36)	109.27 (-58.96, 277.50)	97.56 (16.70, 178.41)	111.31 (57.00, 165.62)	-44.83 (-148.02, 58.37)	147.74 (24.00, 271.48)	-72.79 (-148.70, 3.12)
	2010	151.00 (80.39, 221.60)	1.21 (-146.59, 149.02)	73.74 (-23.25, 170.73)	40.66 (-73.66, 154.98)	-163.69 (-526.54, 199.15)	66.03 (-85.45, 217.51)	101.16 (-64.22, 266.53)	117.26 (31.07, 203.46)	111.98 (56.92, 167.04)	-45.87 (-141.98, 50.23)	143.54 (21.93, 265.14)	-73.56 (-145.07, -2.06)
	2011	145.38 (74.39, 216.36)	20.98 (-103.57, 145.53)	74.06 (-31.62, 179.74)	57.46 (-43.29, 158.20)	124.30 (-194.73, 443.34)	68.35 (-79.86, 216.56)	98.41 (-71.11, 267.92)	114.54 (23.97, 205.11)	115.80 (63.30, 168.30)	-11.59 (-119.96, 96.79)	144.27 (22.10, 266.43)	-73.95 (-145.91, -2.00)

Sex	Year	Location											
		Global	High SDI	High-middle SDI	Middle SDI	Low-middle SDI	Low SDI	AFR	EMR	EUR	AMR	SEAR	WPR
Female	2012	143.25 (73.44, 213.07)	42.28 (-74.63, 159.18)	30.06 (-97.20, 157.32)	72.53 (-24.52, 169.57)	246.42 (-10.80, 503.64)	103.18 (-50.41, 256.77)	97.04 (-70.13, 264.21)	104.99 (19.38, 190.59)	122.08 (67.01, 177.15)	-17.21 (-119.90, 85.49)	151.09 (25.08, 277.11)	-74.11 (-143.21, -5.01)
	2013	138.59 (69.63, 207.54)	58.86 (-54.24, 171.96)	23.87 (-94.72, 142.47)	71.19 (-17.37, 159.75)	96.99 (-119.41, 313.39)	33.31 (-138.53, 205.15)	98.18 (-67.39, 263.74)	101.06 (10.79, 191.33)	120.72 (66.91, 174.52)	-29.56 (-129.53, 70.40)	147.92 (14.26, 281.59)	-74.58 (-141.24, -7.92)
	2014	134.05 (65.17, 202.93)	37.62 (-81.36, 156.59)	-16.51 (-134.75, 101.73)	71.15 (-38.14, 180.44)	31.63 (-145.78, 209.05)	34.55 (-141.22, 210.33)	102.17 (-61.51, 265.85)	104.84 (5.77, 203.92)	119.12 (65.84, 172.40)	-27.54 (-130.28, 75.21)	147.70 (18.48, 276.91)	-73.97 (-142.15, -5.80)
	2015	129.37 (61.22, 197.51)	6.76 (-114.28, 127.79)	-65.05 (-178.74, 48.64)	50.65 (-64.59, 165.88)	39.74 (-109.82, 189.30)	23.41 (-161.26, 208.08)	99.98 (-63.22, 263.17)	90.33 (-14.81, 195.47)	115.48 (62.16, 168.80)	-63.20 (-166.74, 40.35)	141.50 (19.74, 263.26)	-75.74 (-142.36, -9.13)
	2016	130.57 (62.69, 198.46)	45.03 (-69.75, 159.82)	-33.03 (-160.94, 94.88)	63.95 (-54.43, 182.32)	120.34 (-31.83, 272.51)	23.71 (-144.43, 191.85)	101.67 (-61.77, 265.11)	88.30 (-15.01, 191.61)	109.51 (55.16, 163.85)	-66.32 (-163.42, 30.77)	129.73 (6.84, 252.61)	-76.62 (-145.59, -7.64)
	2017	128.72 (61.07, 196.38)	64.06 (-56.68, 184.80)	6.10 (-128.69, 140.89)	55.74 (-74.45, 185.92)	189.28 (54.88, 323.68)	117.60 (-39.34, 274.54)	101.15 (-61.27, 263.57)	80.27 (-16.44, 176.97)	102.86 (51.63, 154.09)	-64.55 (-165.42, 36.33)	125.75 (8.87, 242.64)	-77.25 (-145.77, -8.73)
	2018	125.94 (58.24, 193.64)	83.92 (-42.09, 209.93)	41.96 (-74.60, 158.52)	46.07 (-192.71, 284.85)	189.14 (59.81, 318.47)	111.96 (-64.32, 288.24)	106.06 (-64.73, 276.85)	68.22 (-34.23, 170.67)	98.92 (48.02, 149.82)	-66.86 (-171.44, 37.72)	122.68 (8.79, 236.57)	-79.95 (-149.68, -10.23)
	2019	121.79 (54.10, 189.47)	88.01 (-37.85, 213.88)	42.30 (-80.43, 165.03)	-2.87 (-366.82, 361.08)	160.73 (28.27, 293.19)	102.54 (-75.34, 280.43)	92.04 (-73.22, 257.29)	56.51 (-47.56, 160.58)	92.86 (40.74, 144.98)	-64.85 (-172.51, 42.81)	121.31 (8.29, 234.33)	-80.27 (-149.38, -11.15)
	2020	118.52 (52.17, 184.87)	65.14 (-77.15, 207.43)	-170.45 (-508.20, 167.31)	-72.35 (-258.34, 113.63)	125.99 (-6.94, 258.92)	140.92 (-46.20, 328.04)	96.91 (-64.07, 257.90)	48.63 (-45.64, 142.91)	85.38 (34.94, 135.81)	-55.56 (-158.71, 47.58)	97.44 (-33.29, 228.16)	-85.41 (-156.89, -13.93)
	2021	122.50 (56.69, 188.31)	69.25 (-59.77, 198.28)	-25.31 (-199.11, 148.48)	-85.29 (-248.02, 77.43)	117.86 (-26.32, 262.04)	147.22 (-34.91, 329.34)	82.10 (-70.10, 234.31)	53.16 (-42.72, 149.05)	77.55 (27.83, 127.27)	-59.40 (-165.87, 47.07)	98.86 (-30.86, 228.58)	-81.87 (-151.74, -11.99)
	1990	47.12 (-69.33, 163.56)	134.66 (17.10, 252.22)	36.35 (-126.09, 198.79)	87.99 (-188.72, 364.70)	-52.38 (-267.45, 162.69)	261.08 (30.73, 491.44)	115.53 (-172.17, 403.23)	120.61 (44.91, 196.30)	240.97 (147.07, 334.88)	161.32 (-75.02, 397.67)	202.60 (-85.80, 491.00)	93.23 (-40.85, 227.32)
	1991	54.42 (-63.34, 172.18)	136.89 (44.73, 229.06)	44.36 (-73.99, 162.71)	76.37 (-241.77, 394.51)	-279.48 (-653.51, 94.55)	66.88 (-91.61, 225.36)	114.76 (-169.43, 398.96)	116.34 (49.87, 182.82)	239.47 (145.59, 333.35)	172.91 (-68.34, 414.15)	306.27 (87.96, 524.58)	87.17 (-43.87, 218.20)
	1992	47.33 (-68.32, 162.97)	138.01 (80.94, 195.07)	-25.76 (-209.51, 157.99)	-90.24 (-354.94, 174.46)	-231.53 (-472.95, 9.89)	56.18 (-101.79, 214.16)	118.86 (-166.96, 404.68)	98.50 (31.36, 165.64)	228.15 (130.02, 326.27)	171.43 (-72.66, 415.52)	305.47 (76.75, 534.20)	52.40 (-83.41, 188.20)
	1993	38.11 (-77.31, 153.53)	135.41 (-144.13, 414.96)	56.88 (-128.75, 242.51)	-95.31 (-333.86, 143.25)	-163.56 (-366.80, 39.67)	65.32 (-99.43, 230.07)	146.56 (-146.47, 439.60)	92.61 (24.84, 160.39)	230.72 (135.81, 325.62)	176.42 (-76.80, 429.65)	332.17 (160.46, 503.88)	76.42 (-51.95, 204.80)
	1994	35.61 (-78.78, 150.00)	96.33 (-166.18, 358.84)	103.13 (-160.46, 366.71)	-1.58 (-232.95, 229.79)	-120.20 (-320.36, 79.95)	91.41 (-72.95, 255.77)	140.48 (-154.36, 435.32)	89.49 (21.60, 157.37)	236.28 (144.25, 328.30)	192.57 (-58.78, 443.92)	334.68 (170.71, 498.64)	69.26 (-46.01, 184.54)
	1995	36.40 (-74.89, 147.68)	61.03 (-353.70, 475.77)	159.90 (-142.35, 462.15)	180.89 (-68.42, 430.21)	-118.36 (-291.04, 54.32)	46.26 (-133.79, 226.30)	134.15 (-159.80, 428.10)	78.36 (2.79, 153.92)	250.34 (162.32, 338.35)	186.17 (-74.83, 447.17)	303.69 (49.52, 557.86)	66.62 (-54.21, 187.44)
	1996	35.72 (-73.41, 144.84)	-12.69 (-404.09, 378.71)	186.85 (-116.63, 490.32)	97.13 (-107.46, 301.72)	-52.87 (-220.82, 115.08)	78.64 (-104.18, 261.46)	150.68 (-144.39, 445.74)	78.22 (9.07, 147.37)	242.38 (146.75, 338.01)	174.54 (-81.78, 430.85)	314.07 (74.96, 553.18)	39.42 (-77.27, 156.11)
	1997	33.42 (-74.86, 141.71)	-6.68 (-399.29, 385.94)	142.68 (-117.14, 402.49)	83.87 (-166.40, 334.13)	-8.24 (-174.17, 157.70)	110.93 (-70.22, 292.09)	140.46 (-152.22, 433.14)	78.41 (13.66, 143.15)	239.42 (141.00, 337.84)	161.94 (-89.47, 413.34)	318.28 (89.50, 547.06)	35.58 (-77.11, 148.27)

Sex	Year	Location											
		**Global**	**High SDI**	**High-middle SDI**	**Middle SDI**	**Low-middle SDI**	**Low SDI**	**AFR**	**EMR**	**EUR**	**AMR**	**SEAR**	**WPR**
	1998	27.07 (-80.89, 135.02)	293.47 (-29.43, 616.36)	264.75 (-22.01, 551.51)	-45.22 (-264.65, 174.21)	-78.56 (-243.52, 86.39)	83.06 (-98.09, 264.21)	137.69 (-157.39, 432.77)	84.74 (21.43, 148.05)	223.21 (127.64, 318.79)	164.52 (-85.32, 414.36)	311.99 (79.85, 544.13)	9.60 (-111.41, 130.61)
	1999	33.07 (-72.96, 139.10)	158.48 (-156.70, 473.65)	280.03 (-28.47, 588.52)	-96.39 (-378.65, 185.88)	-67.71 (-227.59, 92.18)	108.63 (-95.15, 312.41)	140.46 (-160.28, 441.21)	74.65 (12.56, 136.74)	221.17 (127.02, 315.31)	155.96 (-93.74, 405.66)	305.26 (72.28, 538.24)	9.13 (-113.65, 131.91)
	2000	23.85 (-82.55, 130.25)	161.64 (-151.98, 475.26)	222.65 (-77.67, 522.98)	-251.25 (-548.23, 45.73)	-95.89 (-270.23, 78.45)	80.33 (-129.02, 289.68)	140.34 (-162.50, 443.18)	87.75 (19.41, 156.08)	226.79 (134.01, 319.57)	144.92 (-106.90, 396.75)	302.14 (61.36, 542.92)	19.19 (-103.28, 141.65)
	2001	26.90 (-79.31, 133.10)	14.07 (-344.48, 372.62)	345.05 (24.72, 665.38)	-146.02 (-505.80, 213.76)	-84.75 (-257.55, 88.05)	88.36 (-119.79, 296.52)	151.42 (-144.32, 447.17)	93.17 (17.26, 169.07)	230.92 (136.55, 325.29)	134.97 (-119.39, 389.32)	294.33 (46.28, 542.37)	15.62 (-104.16, 135.40)
	2002	21.87 (-83.50, 127.23)	3.91 (-246.09, 253.91)	529.07 (169.68, 888.46)	-104.01 (-387.75, 179.74)	-125.73 (-309.48, 58.03)	5.45 (-213.78, 224.67)	151.31 (-151.39, 454.00)	86.15 (6.18, 166.12)	220.10 (124.59, 315.60)	102.69 (-144.26, 349.63)	293.89 (48.23, 539.54)	14.32 (-112.89, 141.52)
	2003	19.18 (-84.51, 122.88)	7.17 (-256.00, 270.34)	522.23 (166.96, 877.50)	-76.12 (-345.12, 192.88)	-145.05 (-329.35, 39.26)	-19.93 (-252.20, 212.34)	157.17 (-150.31, 464.66)	75.96 (-4.39, 156.31)	215.05 (122.07, 308.04)	33.96 (-211.08, 279.00)	285.52 (34.95, 536.09)	13.85 (-116.29, 143.98)
	2004	11.30 (-93.69, 116.29)	-81.36 (-391.51, 228.80)	198.38 (-142.90, 539.65)	-176.66 (-397.96, 44.65)	-131.23 (-310.95, 48.48)	-60.03 (-312.43, 192.37)	148.89 (-156.60, 454.38)	78.86 (-3.59, 161.30)	206.84 (113.70, 299.98)	31.08 (-202.70, 264.86)	289.18 (37.20, 541.15)	9.44 (-122.03, 140.90)
	2005	17.12 (-88.30, 122.53)	-27.65 (-255.03, 199.74)	180.22 (-175.76, 536.20)	-199.69 (-450.22, 50.83)	-126.07 (-327.44, 75.30)	-114.75 (-387.18, 157.67)	169.47 (-139.63, 478.58)	81.05 (-5.09, 167.18)	213.67 (123.60, 303.75)	5.43 (-229.47, 240.33)	283.35 (19.68, 547.03)	5.26 (-118.90, 129.42)
	2006	12.78 (-89.68, 115.23)	47.87 (-46.26, 142.01)	202.68 (-159.61, 564.98)	-165.42 (-485.16, 154.32)	-145.11 (-353.94, 63.73)	-121.91 (-393.45, 149.62)	170.97 (-139.52, 481.45)	75.96 (-13.00, 164.93)	206.05 (115.77, 296.34)	54.90 (-176.51, 286.30)	271.02 (-9.27, 551.31)	-2.19 (-127.91, 123.52)
	2007	15.69 (-86.66, 118.04)	31.13 (-181.36, 243.62)	270.71 (-78.77, 620.20)	-65.07 (-394.22, 264.07)	-148.39 (-412.23, 115.45)	-171.97 (-462.37, 118.44)	176.52 (-132.50, 485.53)	72.43 (-14.72, 159.58)	199.69 (103.22, 296.17)	68.94 (-162.84, 300.71)	255.48 (-44.58, 555.55)	-6.95 (-124.21, 110.31)
	2008	7.91 (-93.98, 109.79)	70.66 (-253.67, 394.98)	104.02 (-128.47, 336.50)	44.91 (-359.26, 449.09)	-215.32 (-505.44, 74.80)	-131.88 (-449.36, 185.60)	178.41 (-135.60, 492.43)	62.78 (-24.67, 150.23)	192.23 (99.38, 285.08)	69.48 (-165.64, 304.59)	238.96 (-80.67, 558.59)	12.53 (-109.98, 135.03)
	2009	11.37 (-91.77, 114.51)	-5.60 (-268.51, 257.30)	133.44 (-76.62, 343.49)	-23.46 (-255.00, 208.08)	-328.72 (-584.70, -72.75)	-272.70 (-688.15, 142.74)	166.49 (-144.70, 477.68)	48.48 (-40.81, 137.77)	194.28 (103.86, 284.70)	48.33 (-186.82, 283.47)	220.59 (-118.47, 559.64)	5.68 (-113.81, 125.18)
	2010	11.15 (-92.25, 114.56)	2.20 (-231.22, 235.63)	163.56 (-19.26, 346.39)	41.47 (-140.01, 222.96)	-409.55 (-912.64, 93.54)	-61.60 (-328.86, 205.67)	152.34 (-158.41, 463.08)	26.76 (-53.83, 107.36)	193.55 (103.41, 283.69)	47.06 (-178.34, 272.47)	205.55 (-97.61, 508.71)	5.55 (-112.67, 123.76)
	2011	5.24 (-96.59, 107.08)	50.33 (-149.34, 250.00)	150.83 (-35.82, 337.48)	107.68 (-62.63, 277.99)	-5.14 (-452.13, 441.86)	-88.07 (-352.50, 176.36)	148.19 (-164.67, 461.05)	16.84 (-56.51, 90.18)	197.07 (110.70, 283.45)	100.80 (-132.17, 333.76)	199.93 (-76.88, 476.74)	7.53 (-109.58, 124.65)
	2012	5.05 (-95.37, 105.47)	32.26 (-138.42, 202.94)	-7.82 (-249.50, 233.87)	128.33 (-53.87, 310.53)	212.96 (-159.16, 585.08)	-28.98 (-302.02, 244.05)	149.09 (-162.41, 460.59)	15.69 (-51.96, 83.34)	205.53 (118.48, 292.59)	101.89 (-131.41, 335.19)	201.22 (-83.94, 486.39)	9.61 (-108.04, 127.26)
	2013	-2.91 (-102.43, 96.60)	67.20 (-110.90, 245.30)	1.23 (-258.39, 260.84)	132.35 (-37.20, 301.90)	39.11 (-269.42, 347.63)	-74.63 (-385.95, 236.69)	150.71 (-158.21, 459.64)	19.45 (-49.44, 88.34)	203.67 (109.97, 297.38)	82.27 (-141.00, 305.54)	196.62 (-54.11, 447.35)	14.17 (-103.93, 132.27)
	2014	-9.11 (-108.31, 90.08)	38.95 (-134.84, 212.73)	-88.72 (-302.50, 125.06)	125.25 (-81.41, 331.91)	17.14 (-234.69, 268.97)	-78.14 (-401.23, 244.96)	158.11 (-148.99, 465.21)	14.24 (-51.76, 80.24)	198.05 (104.27, 291.83)	77.21 (-147.80, 302.21)	198.76 (-49.94, 447.46)	13.06 (-106.25, 132.37)
	2015	-12.52 (-111.13, 86.09)	19.78 (-145.63, 185.18)	-165.50 (-365.87, 34.87)	81.08 (-129.74, 291.89)	5.06 (-225.90, 236.02)	-149.09 (-477.64, 179.45)	159.75 (-149.18, 468.67)	3.98 (-64.14, 72.09)	186.62 (94.14, 279.10)	10.45 (-211.27, 232.17)	192.35 (-53.27, 437.97)	11.16 (-106.03, 128.36)
	2016	-11.74 (-110.10, 86.63)	87.02 (-76.05, 250.08)	-92.41 (-324.97, 140.16)	80.28 (-141.81, 302.37)	69.40 (-161.18, 299.98)	-94.93 (-400.41, 210.55)	160.01 (-148.83, 468.85)	6.52 (-62.70, 75.74)	181.15 (91.24, 271.07)	5.59 (-216.24, 227.43)	175.15 (-67.34, 417.64)	10.54 (-107.54, 128.62)

**Sex**	**Year**	**Location**											
		**Global**	**High SDI**	**High-middle SDI**	**Middle SDI**	**Low-middle SDI**	**Low SDI**	**AFR**	**EMR**	**EUR**	**AMR**	**SEAR**	**WPR**
	2017	-12.74	114.76	-17.01	82.04	130.48	13.21	159.67	-0.37	172.96	5.47	169.36	8.09
		(-111.50, 86.02)	(-60.75, 290.27)	(-309.10, 275.07)	(-151.70, 315.78)	(-61.15, 322.10)	(-301.09, 327.51)	(-147.58, 466.92)	(-74.25, 73.50)	(83.56, 262.36)	(-222.58, 233.53)	(-68.06, 406.78)	(-109.62, 125.80)
	2018	-16.01	143.00	42.55	101.71	143.99	-26.75	165.45	-9.92	171.90	-3.68	166.69	1.86
		(-114.13, 82.10)	(-30.79, 316.80)	(-160.78, 245.88)	(-191.51, 394.94)	(-41.74, 329.72)	(-369.50, 315.99)	(-139.53, 470.42)	(-81.84, 62.00)	(83.41, 260.39)	(-233.25, 225.88)	(-63.14, 396.53)	(-117.06, 120.79)
	2019	-23.68	142.07	7.35	34.03	75.82	-51.80	143.71	-21.25	161.58	-6.56	163.07	-3.28
		(-122.44, 75.09)	(-30.74, 314.88)	(-192.28, 206.98)	(-417.36, 485.43)	(-121.63, 273.28)	(-393.51, 289.92)	(-164.16, 451.59)	(-90.82, 48.31)	(72.98, 250.17)	(-241.71, 228.60)	(-65.31, 391.45)	(-122.87, 116.32)
	2020	-26.47	123.63	-485.77	-167.37	29.05	64.76	145.20	-18.49	146.46	5.33	97.82	-15.48
		(-125.64, 72.71)	(-47.39, 294.66)	(-1008.47, 36.94)	(-429.10, 94.36)	(-157.96, 216.07)	(-315.05, 444.58)	(-159.96, 450.37)	(-75.94, 38.97)	(57.67, 235.25)	(-211.73, 222.39)	(-145.95, 341.59)	(-136.99, 106.02)
	2021	-25.04	135.43	-153.39	-157.69	27.42	80.53	126.87	-8.33	146.56	6.58	96.51	-12.36
		(-123.56, 73.49)	(-32.31, 303.18)	(-437.70, 130.92)	(-413.87, 98.49)	(-170.22, 225.06)	(-294.78, 455.85)	(-174.00, 427.75)	(-66.37, 49.71)	(58.20, 234.92)	(-214.76, 227.92)	(-145.35, 338.36)	(-132.64, 107.92)

Notes: ADRDs, Alzheimer's disease and related dementia; SDI, Socio-demographic Index; WHO, World Health Organization; AFR, African Region; EMR, Eastern Mediterranean Region; AMR, Region of the Americas; EUR, European Region; SEAR, South-East Asia Region; WPR, Western Pacific Region.

Table S5. The year trends of concentration index for burden of ADRDs by sex, SDI quantiles and WHO regions, from 1990 to 2021

Sex	Year	Location											
		Global	High SDI	High-middle SDI	Middle SDI	Low-middle SDI	Low SDI	AFR	EMR	EUR	AMR	SEAR	WPR
Both													
	1990	0.0604 (0.0427, 0.0781)	-0.0450 (-0.0565, -0.0335)	0.0198 (-0.0142, 0.0538)	0.0491 (0.0256, 0.0726)	-0.0352 (-0.0623, -0.0082)	0.1565 (0.1278, 0.1852)	-0.0142 (-0.0507, 0.0223)	0.0610 (0.0073, 0.1147)	0.0234 (0.0041, 0.0427)	0.0671 (0.0260, 0.1083)	0.0925 (0.0094, 0.1756)	-0.0378 (-0.0655, -0.0102)
	1991	0.0544 (0.0362, 0.0726)	-0.0445 (-0.0559, -0.0330)	0.0204 (-0.0119, 0.0526)	0.0484 (0.0248, 0.0721)	-0.0962 (-0.1164, -0.0759)	-0.0185 (-0.0622, 0.0252)	-0.0123 (-0.0489, 0.0242)	0.0604 (0.0080, 0.1129)	0.0228 (0.0037, 0.0418)	0.0686 (0.0275, 0.1098)	0.0962 (0.0124, 0.1800)	-0.0410 (-0.0689, -0.0132)
	1992	0.0537 (0.0355, 0.0718)	-0.0447 (-0.0563, -0.0332)	-0.0022 (-0.0330, 0.0286)	-0.0031 (-0.0169, 0.0108)	-0.0952 (-0.1153, -0.0752)	-0.0181 (-0.0618, 0.0257)	-0.0123 (-0.0487, 0.0242)	0.0595 (0.0083, 0.1108)	0.0226 (0.0037, 0.0415)	0.0694 (0.0284, 0.1105)	0.0980 (0.0144, 0.1816)	-0.0422 (-0.0697, -0.0148)
	1993	0.0534 (0.0352, 0.0716)	0.0109 (-0.0554, 0.0772)	0.0029 (-0.0290, 0.0348)	-0.0063 (-0.0196, 0.0071)	-0.0951 (-0.1152, -0.0749)	-0.0183 (-0.0627, 0.0261)	-0.0113 (-0.0477, 0.0251)	0.0587 (0.0086, 0.1088)	0.0230 (0.0042, 0.0417)	0.0700 (0.0286, 0.1113)	0.1001 (0.0157, 0.1846)	-0.0435 (-0.0709, -0.0160)
	1994	0.0524 (0.0341, 0.0706)	0.0131 (-0.0276, 0.0538)	0.0079 (-0.0242, 0.0400)	-0.0039 (-0.0178, 0.0101)	-0.0971 (-0.1165, -0.0777)	-0.0180 (-0.0624, 0.0265)	-0.0104 (-0.0469, 0.0261)	0.0580 (0.0089, 0.1071)	0.0227 (0.0040, 0.0414)	0.0706 (0.0293, 0.1120)	0.1012 (0.0166, 0.1859)	-0.0453 (-0.0726, -0.0180)
	1995	0.0531 (0.0351, 0.0711)	0.0296 (0.0085, 0.0507)	0.0337 (0.0017, 0.0657)	-0.0003 (-0.0139, 0.0133)	-0.0751 (-0.0987, -0.0516)	-0.0523 (-0.0950, -0.0096)	-0.0115 (-0.0482, 0.0252)	0.0569 (0.0088, 0.1051)	0.0234 (0.0047, 0.0421)	0.0706 (0.0294, 0.1119)	0.0994 (0.0160, 0.1827)	-0.0454 (-0.0721, -0.0187)
	1996	0.0579 (0.0401, 0.0756)	0.0308 (0.0101, 0.0515)	0.0418 (0.0099, 0.0737)	0.0114 (0.0006, 0.0223)	-0.0294 (-0.0596, 0.0009)	-0.0524 (-0.0956, -0.0091)	-0.0105 (-0.0476, 0.0266)	0.0568 (0.0094, 0.1042)	0.0232 (0.0045, 0.0419)	0.0695 (0.0281, 0.1109)	0.1002 (0.0160, 0.1844)	-0.0459 (-0.0725, -0.0192)
	1997	0.0578 (0.0401, 0.0756)	0.0307 (0.0100, 0.0514)	0.0432 (0.0133, 0.0730)	0.0085 (-0.0027, 0.0197)	-0.0277 (-0.0579, 0.0025)	-0.0533 (-0.0970, -0.0095)	-0.0123 (-0.0494, 0.0248)	0.0568 (0.0103, 0.1033)	0.0227 (0.0040, 0.0415)	0.0684 (0.0268, 0.1100)	0.1014 (0.0160, 0.1868)	-0.0455 (-0.0719, -0.0192)
	1998	0.0579 (0.0402, 0.0755)	0.0302 (0.0094, 0.0510)	0.0475 (0.0179, 0.0770)	0.0010 (-0.0126, 0.0145)	-0.0253 (-0.0554, 0.0047)	-0.0878 (-0.1277, -0.0479)	-0.0131 (-0.0506, 0.0244)	0.0569 (0.0114, 0.1025)	0.0227 (0.0040, 0.0414)	0.0673 (0.0255, 0.1092)	0.1014 (0.0162, 0.1866)	-0.0444 (-0.0707, -0.0182)
	1999	0.0603 (0.0428, 0.0778)	0.0269 (0.0078, 0.0460)	0.0573 (0.0298, 0.0848)	-0.0013 (-0.0153, 0.0128)	-0.0130 (-0.0433, 0.0174)	-0.0891 (-0.1301, -0.0481)	-0.0124 (-0.0501, 0.0254)	0.0567 (0.0118, 0.1016)	0.0228 (0.0043, 0.0414)	0.0669 (0.0247, 0.1092)	0.1018 (0.0142, 0.1895)	-0.0433 (-0.0693, -0.0172)
	2000	0.0616 (0.0442, 0.0791)	0.0274 (0.0085, 0.0464)	0.0561 (0.0295, 0.0828)	-0.0007 (-0.0154, 0.0141)	-0.0011 (-0.0311, 0.0290)	-0.1008 (-0.1401, -0.0615)	-0.0119 (-0.0497, 0.0260)	0.0559 (0.0121, 0.0997)	0.0230 (0.0047, 0.0413)	0.0664 (0.0240, 0.1089)	0.1018 (0.0130, 0.1906)	-0.0160 (-0.0471, 0.0151)
	2001	0.0610 (0.0437, 0.0784)	0.0251 (0.0057, 0.0444)	0.0604 (0.0357, 0.0850)	0.0027 (-0.0128, 0.0181)	-0.0007 (-0.0313, 0.0298)	-0.0982 (-0.1369, -0.0594)	-0.0115 (-0.0495, 0.0265)	0.0556 (0.0129, 0.0983)	0.0221 (0.0039, 0.0403)	0.0656 (0.0230, 0.1081)	0.0999 (0.0126, 0.1871)	-0.0159 (-0.0468, 0.0151)
	2002	0.0618 (0.0446, 0.0790)	0.0247 (0.0066, 0.0428)	0.0665 (0.0452, 0.0878)	0.0104 (-0.0083, 0.0291)	0.0077 (-0.0221, 0.0375)	-0.1001 (-0.1382, -0.0620)	-0.0108 (-0.0486, 0.0270)	0.0551 (0.0132, 0.0970)	0.0222 (0.0041, 0.0402)	0.0636 (0.0207, 0.1064)	0.0995 (0.0129, 0.1861)	-0.0154 (-0.0465, 0.0156)
	2003	0.0619 (0.0448, 0.0790)	0.0240 (0.0060, 0.0419)	0.0662 (0.0450, 0.0875)	0.0105 (-0.0080, 0.0289)	0.0131 (-0.0164, 0.0427)	-0.0974 (-0.1357, -0.0591)	-0.0117 (-0.0497, 0.0263)	0.0544 (0.0131, 0.0956)	0.0225 (0.0046, 0.0404)	0.0637 (0.0211, 0.1063)	0.0986 (0.0138, 0.1835)	-0.0151 (-0.0464, 0.0163)
	2004	0.0611 (0.0439, 0.0782)	0.0005 (-0.0260, 0.0270)	0.0568 (0.0343, 0.0792)	-0.0053 (-0.0249, 0.0143)	0.0142 (-0.0151, 0.0435)	-0.0960 (-0.1344, -0.0576)	-0.0117 (-0.0496, 0.0261)	0.0532 (0.0125, 0.0939)	0.0223 (0.0047, 0.0399)	0.0623 (0.0197, 0.1049)	0.0990 (0.0157, 0.1822)	-0.0146 (-0.0462, 0.0170)
	2005	0.0650 (0.0481, 0.0818)	0.0007 (-0.0236, 0.0250)	0.0551 (0.0314, 0.0788)	-0.0059 (-0.0248, 0.0131)	0.0420 (0.0138, 0.0702)	-0.0965 (-0.1348, -0.0582)	-0.0126 (-0.0504, 0.0252)	0.0524 (0.0120, 0.0927)	0.0224 (0.0050, 0.0398)	0.0629 (0.0205, 0.1053)	0.0988 (0.0162, 0.1814)	-0.0140 (-0.0455, 0.0174)

Sex	Year	Location											
		Global	High SDI	High-middle SDI	Middle SDI	Low-middle SDI	Low SDI	AFR	EMR	EUR	AMR	SEAR	WPR
	2006	0.0650 (0.0484, 0.0815)	0.0038 (-0.0189, 0.0265)	0.0565 (0.0325, 0.0805)	-0.0246 (-0.0454, -0.0037)	0.0486 (0.0206, 0.0767)	-0.0952 (-0.1332, -0.0573)	-0.0133 (-0.0509, 0.0244)	0.0515 (0.0115, 0.0916)	0.0218 (0.0042, 0.0393)	0.0646 (0.0224, 0.1068)	0.0972 (0.0160, 0.1784)	-0.0129 (-0.0433, 0.0174)
	2007	0.0650 (0.0487, 0.0812)	0.0080 (-0.0149, 0.0310)	0.0586 (0.0353, 0.0820)	-0.0118 (-0.0326, 0.0090)	0.0601 (0.0332, 0.0870)	-0.0960 (-0.1330, -0.0590)	-0.0137 (-0.0517, 0.0243)	0.0509 (0.0110, 0.0909)	0.0219 (0.0044, 0.0395)	0.0642 (0.0219, 0.1066)	0.0953 (0.0161, 0.1744)	-0.0122 (-0.0419, 0.0176)
	2008	0.0647 (0.0485, 0.0809)	0.0050 (-0.0249, 0.0348)	0.0064 (-0.0175, 0.0303)	-0.1041 (-0.1169, -0.0913)	-0.0108 (-0.0419, 0.0204)	-0.0977 (-0.1350, -0.0603)	-0.0141 (-0.0519, 0.0238)	0.0507 (0.0110, 0.0904)	0.0215 (0.0038, 0.0392)	0.0631 (0.0205, 0.1057)	0.0944 (0.0160, 0.1729)	-0.0119 (-0.0415, 0.0177)
	2009	0.0671 (0.0510, 0.0831)	-0.0040 (-0.0329, 0.0250)	0.0079 (-0.0135, 0.0293)	-0.0346 (-0.0693, 0.0001)	-0.0131 (-0.0437, 0.0176)	-0.1197 (-0.1528, -0.0866)	-0.0145 (-0.0524, 0.0233)	0.0499 (0.0104, 0.0895)	0.0209 (0.0032, 0.0387)	0.0617 (0.0191, 0.1044)	0.0946 (0.0151, 0.1740)	-0.0120 (-0.0418, 0.0178)
	2010	0.0680 (0.0520, 0.0839)	-0.0022 (-0.0311, 0.0267)	0.0085 (-0.0122, 0.0291)	-0.0090 (-0.0461, 0.0281)	0.1242 (0.0880, 0.1604)	-0.0385 (-0.0726, -0.0045)	-0.0154 (-0.0532, 0.0224)	0.0560 (0.0187, 0.0932)	0.0207 (0.0031, 0.0384)	0.0614 (0.0187, 0.1042)	0.0936 (0.0145, 0.1726)	-0.0111 (-0.0406, 0.0183)
	2011	0.0690 (0.0532, 0.0849)	0.0053 (-0.0226, 0.0333)	0.0094 (-0.0113, 0.0301)	0.0219 (-0.0132, 0.0569)	0.1217 (0.0837, 0.1597)	-0.0526 (-0.0877, -0.0174)	-0.0171 (-0.0549, 0.0207)	0.0551 (0.0177, 0.0925)	0.0213 (0.0036, 0.0390)	0.0844 (0.0463, 0.1225)	0.0943 (0.0166, 0.1720)	-0.0084 (-0.0374, 0.0206)
	2012	0.0693 (0.0535, 0.0850)	0.0111 (-0.0142, 0.0364)	0.0106 (-0.0105, 0.0318)	0.0449 (0.0073, 0.0826)	0.1278 (0.0922, 0.1634)	-0.0491 (-0.0857, -0.0124)	-0.0164 (-0.0543, 0.0216)	0.0545 (0.0168, 0.0923)	0.0213 (0.0037, 0.0389)	0.0842 (0.0460, 0.1224)	0.0957 (0.0201, 0.1713)	-0.0070 (-0.0356, 0.0215)
	2013	0.0676 (0.0520, 0.0832)	0.0272 (0.0017, 0.0527)	0.0301 (0.0114, 0.0488)	0.0460 (0.0072, 0.0849)	0.1131 (0.0779, 0.1482)	-0.0609 (-0.0985, -0.0233)	-0.0172 (-0.0551, 0.0208)	0.0540 (0.0161, 0.0919)	0.0208 (0.0033, 0.0384)	0.0821 (0.0434, 0.1208)	0.0917 (0.0223, 0.1611)	-0.0064 (-0.0348, 0.0220)
	2014	0.0661 (0.0504, 0.0819)	0.0264 (0.0015, 0.0513)	0.0281 (0.0091, 0.0471)	0.0595 (0.0229, 0.0961)	0.0913 (0.0523, 0.1304)	-0.0613 (-0.0992, -0.0234)	-0.0180 (-0.0564, 0.0203)	0.0529 (0.0150, 0.0907)	0.0195 (0.0019, 0.0371)	0.0813 (0.0424, 0.1202)	0.0903 (0.0232, 0.1574)	-0.0070 (-0.0360, 0.0221)
	2015	0.0656 (0.0498, 0.0814)	0.0255 (0.0010, 0.0500)	0.0219 (0.0023, 0.0416)	0.0686 (0.0353, 0.1019)	0.0736 (0.0228, 0.1244)	-0.0759 (-0.1129, -0.0389)	-0.0180 (-0.0566, 0.0205)	0.0517 (0.0136, 0.0899)	0.0186 (0.0009, 0.0363)	0.0539 (0.0094, 0.0984)	0.0889 (0.0232, 0.1546)	-0.0072 (-0.0365, 0.0220)
	2016	0.0650 (0.0491, 0.0809)	0.0287 (0.0052, 0.0521)	0.0269 (0.0072, 0.0467)	0.0668 (0.0350, 0.0987)	0.0600 (0.0112, 0.1088)	-0.0784 (-0.1174, -0.0395)	-0.0187 (-0.0576, 0.0201)	0.0512 (0.0130, 0.0894)	0.0193 (0.0011, 0.0375)	0.0527 (0.0080, 0.0974)	0.0879 (0.0222, 0.1535)	-0.0077 (-0.0371, 0.0218)
	2017	0.0649 (0.0491, 0.0806)	0.0297 (0.0064, 0.0529)	-0.0003 (-0.0233, 0.0227)	0.0735 (0.0453, 0.1018)	0.0405 (-0.0063, 0.0873)	-0.0633 (-0.1065, -0.0202)	-0.0196 (-0.0586, 0.0193)	0.0490 (0.0114, 0.0866)	0.0107 (-0.0088, 0.0302)	0.0531 (0.0084, 0.0978)	0.0851 (0.0209, 0.1494)	-0.0080 (-0.0376, 0.0216)
	2018	0.0644 (0.0486, 0.0802)	0.0323 (0.0091, 0.0555)	0.0104 (-0.0107, 0.0315)	0.0826 (0.0550, 0.1101)	0.0382 (-0.0085, 0.0850)	-0.0677 (-0.1109, -0.0246)	-0.0200 (-0.0591, 0.0192)	0.0480 (0.0102, 0.0857)	0.0117 (-0.0084, 0.0319)	0.0525 (0.0077, 0.0973)	0.0842 (0.0210, 0.1475)	-0.0087 (-0.0387, 0.0213)
	2019	0.0633 (0.0475, 0.0791)	0.0299 (0.0063, 0.0536)	0.0083 (-0.0124, 0.0289)	0.0831 (0.0553, 0.1108)	0.0358 (-0.0054, 0.0771)	-0.0088 (-0.0526, 0.0349)	-0.0205 (-0.0598, 0.0188)	0.0463 (0.0087, 0.0840)	0.0114 (-0.0091, 0.0319)	0.0439 (-0.0019, 0.0896)	0.0835 (0.0209, 0.1460)	-0.0095 (-0.0397, 0.0208)
	2020	0.0614 (0.0458, 0.0770)	0.0298 (0.0077, 0.0518)	-0.0993 (-0.1142, -0.0844)	-0.0289 (-0.0604, 0.0026)	-0.0068 (-0.0452, 0.0316)	0.0026 (-0.0436, 0.0488)	-0.0190 (-0.0588, 0.0209)	0.0455 (0.0080, 0.0831)	0.0117 (-0.0084, 0.0318)	0.0431 (-0.0027, 0.0889)	0.0662 (-0.0022, 0.1346)	-0.0109 (-0.0417, 0.0199)
	2021	0.0639 (0.0470, 0.0809)	0.0329 (0.0112, 0.0546)	-0.0971 (-0.1191, -0.0750)	-0.0433 (-0.0728, -0.0137)	-0.0126 (-0.0517, 0.0265)	0.0031 (-0.0428, 0.0491)	-0.0204 (-0.0597, 0.0189)	0.0443 (0.0074, 0.0811)	0.0105 (-0.0093, 0.0304)	0.0423 (-0.0036, 0.0882)	0.0663 (-0.0025, 0.1351)	-0.0172 (-0.0551, 0.0208)
Male	1990	0.0628 (0.0456, 0.0800)	-0.0484 (-0.0558, -0.0409)	0.0116 (-0.0331, 0.0563)	0.0387 (0.0209, 0.0565)	-0.0449 (-0.0733, -0.0165)	0.1516 (0.1251, 0.1781)	-0.0076 (-0.0445, 0.0294)	0.0747 (0.0209, 0.1285)	0.0087 (-0.0092, 0.0267)	0.0616 (0.0194, 0.1038)	0.0769 (0.0059, 0.1478)	-0.0542 (-0.0785, -0.0298)
	1991	0.0579 (0.0404, 0.0755)	-0.0473 (-0.0546, -0.0400)	0.0118 (-0.0306, 0.0543)	0.0375 (0.0192, 0.0558)	-0.0768 (-0.0994, -0.0542)	0.0009 (-0.0402, 0.0421)	-0.0054 (-0.0421, 0.0313)	0.0744 (0.0217, 0.1270)	0.0076 (-0.0104, 0.0255)	0.0633 (0.0210, 0.1055)	0.0798 (0.0081, 0.1515)	-0.0567 (-0.0813, -0.0321)

Sex	Year	Location											
		Global	High SDI	High-middle SDI	Middle SDI	Low-middle SDI	Low SDI	AFR	EMR	EUR	AMR	SEAR	WPR
	1992	0.0574<br>(0.0398, 0.0751)	-0.0473<br>(-0.0546, -0.0399)	0.0003<br>(-0.0365, 0.0370)	-0.0035<br>(-0.0165, 0.0094)	-0.0783<br>(-0.1002, -0.0564)	0.0011<br>(-0.0404, 0.0425)	-0.0056<br>(-0.0421, 0.0309)	0.0738<br>(0.0221, 0.1255)	0.0074<br>(-0.0106, 0.0253)	0.0645<br>(0.0222, 0.1067)	0.0816<br>(0.0092, 0.1541)	-0.0580<br>(-0.0824, -0.0336)
	1993	0.0570<br>(0.0393, 0.0747)	-0.0222<br>(-0.0558, 0.0114)	0.0026<br>(-0.0360, 0.0411)	-0.0081<br>(-0.0205, 0.0043)	-0.0784<br>(-0.1002, -0.0566)	0.0010<br>(-0.0409, 0.0430)	-0.0043<br>(-0.0406, 0.0321)	0.0734<br>(0.0226, 0.1241)	0.0076<br>(-0.0103, 0.0255)	0.0651<br>(0.0227, 0.1076)	0.0830<br>(0.0097, 0.1562)	-0.0595<br>(-0.0843, -0.0347)
	1994	0.0556<br>(0.0379, 0.0734)	-0.0146<br>(-0.0371, 0.0080)	0.0065<br>(-0.0318, 0.0449)	-0.0054<br>(-0.0187, 0.0078)	-0.0847<br>(-0.1047, -0.0647)	0.0022<br>(-0.0398, 0.0442)	-0.0036<br>(-0.0400, 0.0328)	0.0729<br>(0.0231, 0.1228)	0.0077<br>(-0.0103, 0.0256)	0.0660<br>(0.0236, 0.1083)	0.0832<br>(0.0100, 0.1564)	-0.0603<br>(-0.0850, -0.0356)
	1995	0.0568<br>(0.0392, 0.0743)	0.0337<br>(0.0144, 0.0531)	0.0543<br>(0.0227, 0.0860)	-0.0060<br>(-0.0184, 0.0064)	-0.0689<br>(-0.0914, -0.0464)	-0.0366<br>(-0.0750, 0.0018)	-0.0037<br>(-0.0402, 0.0329)	0.0721<br>(0.0231, 0.1211)	0.0080<br>(-0.0098, 0.0258)	0.0662<br>(0.0239, 0.1084)	0.0830<br>(0.0099, 0.1560)	-0.0608<br>(-0.0851, -0.0364)
	1996	0.0611<br>(0.0439, 0.0783)	0.0341<br>(0.0147, 0.0535)	0.0621<br>(0.0309, 0.0934)	0.0154<br>(0.0052, 0.0257)	-0.0242<br>(-0.0525, 0.0040)	-0.0358<br>(-0.0744, 0.0028)	-0.0033<br>(-0.0402, 0.0335)	0.0720<br>(0.0237, 0.1202)	0.0080<br>(-0.0098, 0.0258)	0.0645<br>(0.0222, 0.1068)	0.0825<br>(0.0097, 0.1552)	-0.0609<br>(-0.0852, -0.0366)
	1997	0.0604<br>(0.0433, 0.0775)	0.0342<br>(0.0146, 0.0538)	0.0603<br>(0.0311, 0.0895)	0.0138<br>(0.0031, 0.0244)	-0.0227<br>(-0.0511, 0.0056)	-0.0338<br>(-0.0725, 0.0049)	-0.0053<br>(-0.0424, 0.0318)	0.0720<br>(0.0245, 0.1194)	0.0081<br>(-0.0096, 0.0258)	0.0617<br>(0.0193, 0.1041)	0.0814<br>(0.0096, 0.1533)	-0.0608<br>(-0.0850, -0.0366)
	1998	0.0601<br>(0.0430, 0.0771)	0.0339<br>(0.0145, 0.0534)	0.0614<br>(0.0325, 0.0902)	0.0000<br>(-0.0166, 0.0166)	-0.0212<br>(-0.0495, 0.0071)	-0.0584<br>(-0.0946, -0.0223)	-0.0061<br>(-0.0435, 0.0313)	0.0720<br>(0.0253, 0.1187)	0.0083<br>(-0.0094, 0.0259)	0.0586<br>(0.0160, 0.1012)	0.0818<br>(0.0098, 0.1538)	-0.0598<br>(-0.0839, -0.0357)
	1999	0.0611<br>(0.0441, 0.0780)	0.0209<br>(-0.0036, 0.0453)	0.0690<br>(0.0424, 0.0956)	0.0008<br>(-0.0157, 0.0173)	-0.0174<br>(-0.0455, 0.0108)	-0.0597<br>(-0.0969, -0.0225)	-0.0057<br>(-0.0434, 0.0319)	0.0721<br>(0.0258, 0.1184)	0.0086<br>(-0.0089, 0.0261)	0.0566<br>(0.0137, 0.0994)	0.0820<br>(0.0072, 0.1568)	-0.0585<br>(-0.0824, -0.0346)
	2000	0.0622<br>(0.0453, 0.0790)	0.0214<br>(-0.0031, 0.0459)	0.0654<br>(0.0393, 0.0914)	0.0006<br>(-0.0167, 0.0179)	-0.0075<br>(-0.0354, 0.0204)	-0.0710<br>(-0.1056, -0.0363)	-0.0058<br>(-0.0434, 0.0318)	0.0713<br>(0.0258, 0.1168)	0.0090<br>(-0.0083, 0.0263)	0.0556<br>(0.0126, 0.0985)	0.0817<br>(0.0062, 0.1572)	-0.0332<br>(-0.0644, -0.0020)
	2001	0.0618<br>(0.0450, 0.0786)	0.0203<br>(-0.0037, 0.0443)	0.0657<br>(0.0415, 0.0898)	0.0020<br>(-0.0168, 0.0209)	-0.0071<br>(-0.0355, 0.0213)	-0.0684<br>(-0.1026, -0.0342)	-0.0054<br>(-0.0432, 0.0324)	0.0716<br>(0.0268, 0.1164)	0.0085<br>(-0.0086, 0.0256)	0.0549<br>(0.0118, 0.0979)	0.0799<br>(0.0058, 0.1541)	-0.0330<br>(-0.0641, -0.0018)
	2002	0.0624<br>(0.0458, 0.0791)	0.0198<br>(-0.0029, 0.0425)	0.0612<br>(0.0380, 0.0844)	0.0035<br>(-0.0195, 0.0266)	0.0009<br>(-0.0270, 0.0288)	-0.0701<br>(-0.1038, -0.0365)	-0.0048<br>(-0.0422, 0.0327)	0.0718<br>(0.0271, 0.1164)	0.0089<br>(-0.0080, 0.0258)	0.0533<br>(0.0100, 0.0965)	0.0812<br>(0.0070, 0.1553)	-0.0328<br>(-0.0645, -0.0011)
	2003	0.0623<br>(0.0457, 0.0789)	0.0195<br>(-0.0026, 0.0417)	0.0605<br>(0.0374, 0.0837)	0.0041<br>(-0.0185, 0.0267)	0.0052<br>(-0.0225, 0.0329)	-0.0672<br>(-0.1009, -0.0335)	-0.0048<br>(-0.0424, 0.0328)	0.0714<br>(0.0272, 0.1155)	0.0094<br>(-0.0073, 0.0261)	0.0535<br>(0.0104, 0.0965)	0.0807<br>(0.0086, 0.1528)	-0.0327<br>(-0.0652, -0.0002)
	2004	0.0617<br>(0.0451, 0.0782)	0.0068<br>(-0.0156, 0.0293)	0.0628<br>(0.0404, 0.0853)	0.0072<br>(-0.0185, 0.0329)	0.0060<br>(-0.0213, 0.0334)	-0.0651<br>(-0.0987, -0.0315)	-0.0047<br>(-0.0422, 0.0328)	0.0707<br>(0.0268, 0.1145)	0.0093<br>(-0.0073, 0.0258)	0.0524<br>(0.0094, 0.0955)	0.0812<br>(0.0103, 0.1521)	-0.0322<br>(-0.0650, 0.0006)
	2005	0.0658<br>(0.0495, 0.0821)	0.0070<br>(-0.0133, 0.0272)	0.0610<br>(0.0372, 0.0848)	0.0051<br>(-0.0193, 0.0295)	0.0305<br>(0.0036, 0.0575)	-0.0674<br>(-0.1012, -0.0335)	-0.0053<br>(-0.0427, 0.0320)	0.0701<br>(0.0265, 0.1137)	0.0095<br>(-0.0068, 0.0259)	0.0534<br>(0.0105, 0.0964)	0.0819<br>(0.0095, 0.1544)	-0.0315<br>(-0.0645, 0.0014)
	2006	0.0661<br>(0.0500, 0.0823)	0.0064<br>(-0.0124, 0.0251)	0.0628<br>(0.0387, 0.0868)	-0.0269<br>(-0.0556, 0.0018)	0.0340<br>(0.0065, 0.0615)	-0.0671<br>(-0.1008, -0.0334)	-0.0060<br>(-0.0432, 0.0312)	0.0696<br>(0.0261, 0.1131)	0.0094<br>(-0.0069, 0.0258)	0.0554<br>(0.0126, 0.0981)	0.0812<br>(0.0089, 0.1535)	-0.0305<br>(-0.0628, 0.0019)
	2007	0.0661<br>(0.0502, 0.0820)	0.0086<br>(-0.0111, 0.0283)	0.0637<br>(0.0401, 0.0873)	-0.0018<br>(-0.0323, 0.0287)	0.0459<br>(0.0194, 0.0725)	-0.0664<br>(-0.0994, -0.0335)	-0.0061<br>(-0.0434, 0.0312)	0.0692<br>(0.0259, 0.1125)	0.0101<br>(-0.0062, 0.0264)	0.0552<br>(0.0121, 0.0982)	0.0787<br>(0.0088, 0.1486)	-0.0293<br>(-0.0613, 0.0027)
	2008	0.0660<br>(0.0501, 0.0819)	-0.0097<br>(-0.0438, 0.0244)	0.0016<br>(-0.0210, 0.0243)	-0.0948<br>(-0.1127, -0.0769)	-0.0013<br>(-0.0345, 0.0320)	-0.0664<br>(-0.0998, -0.0329)	-0.0063<br>(-0.0434, 0.0309)	0.0682<br>(0.0247, 0.1118)	0.0102<br>(-0.0061, 0.0266)	0.0541<br>(0.0108, 0.0975)	0.0780<br>(0.0087, 0.1474)	-0.0282<br>(-0.0599, 0.0036)
	2009	0.0683<br>(0.0526, 0.0840)	-0.0192<br>(-0.0513, 0.0130)	0.0035<br>(-0.0170, 0.0240)	-0.0304<br>(-0.0659, 0.0052)	-0.0055<br>(-0.0383, 0.0273)	-0.0876<br>(-0.1148, -0.0603)	-0.0062<br>(-0.0432, 0.0308)	0.0675<br>(0.0240, 0.1110)	0.0102<br>(-0.0063, 0.0267)	0.0530<br>(0.0095, 0.0964)	0.0779<br>(0.0081, 0.1477)	-0.0271<br>(-0.0591, 0.0049)

Sex	Year	Location											
		**Global**	**High SDI**	**High-middle SDI**	**Middle SDI**	**Low-middle SDI**	**Low SDI**	**AFR**	**EMR**	**EUR**	**AMR**	**SEAR**	**WPR**
Female	2010	0.0695 (0.0540, 0.0850)	-0.0156 (-0.0480, 0.0169)	0.0048 (-0.0149, 0.0245)	-0.0004 (-0.0379, 0.0371)	0.1309 (0.0937, 0.1680)	-0.0212 (-0.0503, 0.0079)	-0.0068 (-0.0439, 0.0303)	0.0755 (0.0359, 0.1151)	0.0099 (-0.0067, 0.0264)	0.0530 (0.0093, 0.0967)	0.0774 (0.0083, 0.1465)	-0.0260 (-0.0576, 0.0056)
	2011	0.0704 (0.0549, 0.0859)	-0.0107 (-0.0419, 0.0206)	0.0053 (-0.0145, 0.0252)	0.0123 (-0.0225, 0.0472)	0.1295 (0.0908, 0.1682)	-0.0293 (-0.0596, 0.0009)	-0.0081 (-0.0452, 0.0289)	0.0741 (0.0343, 0.1139)	0.0111 (-0.0054, 0.0275)	0.0769 (0.0372, 0.1166)	0.0783 (0.0109, 0.1458)	-0.0247 (-0.0565, 0.0071)
	2012	0.0711 (0.0556, 0.0866)	-0.0037 (-0.0331, 0.0257)	0.0128 (-0.0072, 0.0329)	0.0353 (-0.0025, 0.0731)	0.1357 (0.0991, 0.1724)	-0.0275 (-0.0586, 0.0037)	-0.0069 (-0.0439, 0.0300)	0.0732 (0.0333, 0.1130)	0.0121 (-0.0041, 0.0282)	0.0773 (0.0373, 0.1172)	0.0808 (0.0156, 0.1461)	-0.0241 (-0.0561, 0.0079)
	2013	0.0700 (0.0546, 0.0854)	0.0106 (-0.0184, 0.0396)	0.0298 (0.0115, 0.0481)	0.0373 (-0.0019, 0.0766)	0.1193 (0.0821, 0.1564)	-0.0452 (-0.0749, -0.0154)	-0.0071 (-0.0440, 0.0297)	0.0728 (0.0326, 0.1131)	0.0130 (-0.0029, 0.0289)	0.0755 (0.0350, 0.1161)	0.0784 (0.0187, 0.1381)	-0.0241 (-0.0566, 0.0085)
	2014	0.0682 (0.0528, 0.0836)	0.0089 (-0.0194, 0.0373)	0.0268 (0.0084, 0.0453)	0.0437 (0.0061, 0.0813)	0.0860 (0.0448, 0.1272)	-0.0448 (-0.0747, -0.0149)	-0.0076 (-0.0447, 0.0295)	0.0710 (0.0312, 0.1108)	0.0129 (-0.0029, 0.0287)	0.0754 (0.0345, 0.1164)	0.0762 (0.0195, 0.1328)	-0.0240 (-0.0571, 0.0090)
	2015	0.0682 (0.0527, 0.0838)	0.0075 (-0.0202, 0.0352)	0.0221 (0.0036, 0.0406)	0.0593 (0.0251, 0.0935)	0.0784 (0.0266, 0.1303)	-0.0515 (-0.0817, -0.0212)	-0.0075 (-0.0445, 0.0295)	0.0694 (0.0295, 0.1093)	0.0124 (-0.0032, 0.0281)	0.0474 (0.0014, 0.0934)	0.0760 (0.0200, 0.1319)	-0.0242 (-0.0580, 0.0096)
	2016	0.0685 (0.0529, 0.0840)	0.0114 (-0.0154, 0.0382)	0.0272 (0.0091, 0.0452)	0.0572 (0.0246, 0.0897)	0.0676 (0.0178, 0.1173)	-0.0599 (-0.0917, -0.0282)	-0.0076 (-0.0448, 0.0297)	0.0692 (0.0294, 0.1090)	0.0135 (-0.0025, 0.0294)	0.0462 (-0.0000, 0.0925)	0.0756 (0.0184, 0.1327)	-0.0231 (-0.0566, 0.0104)
	2017	0.0693 (0.0539, 0.0848)	0.0138 (-0.0127, 0.0403)	0.0071 (-0.0141, 0.0283)	0.0643 (0.0349, 0.0937)	0.0557 (0.0073, 0.1040)	-0.0333 (-0.0678, 0.0011)	-0.0082 (-0.0454, 0.0290)	0.0680 (0.0287, 0.1073)	0.0071 (-0.0100, 0.0241)	0.0464 (0.0001, 0.0927)	0.0734 (0.0164, 0.1305)	-0.0214 (-0.0549, 0.0121)
	2018	0.0690 (0.0536, 0.0844)	0.0178 (-0.0085, 0.0442)	0.0209 (0.0007, 0.0411)	0.0709 (0.0416, 0.1001)	0.0538 (0.0057, 0.1020)	-0.0375 (-0.0721, -0.0028)	-0.0089 (-0.0465, 0.0287)	0.0671 (0.0280, 0.1063)	0.0078 (-0.0102, 0.0259)	0.0455 (-0.0009, 0.0919)	0.0731 (0.0170, 0.1291)	-0.0197 (-0.0531, 0.0136)
	2019	0.0684 (0.0530, 0.0839)	0.0170 (-0.0097, 0.0436)	0.0183 (-0.0019, 0.0385)	0.0716 (0.0421, 0.1011)	0.0446 (0.0021, 0.0871)	0.0015 (-0.0359, 0.0388)	-0.0088 (-0.0464, 0.0287)	0.0652 (0.0265, 0.1039)	0.0081 (-0.0105, 0.0268)	0.0369 (-0.0103, 0.0842)	0.0728 (0.0174, 0.1282)	-0.0192 (-0.0528, 0.0145)
	2020	0.0668 (0.0516, 0.0820)	0.0154 (-0.0097, 0.0406)	-0.0955 (-0.1098, -0.0811)	-0.0233 (-0.0579, 0.0113)	0.0155 (-0.0268, 0.0578)	0.0090 (-0.0297, 0.0477)	-0.0071 (-0.0450, 0.0308)	0.0650 (0.0260, 0.1039)	0.0091 (-0.0092, 0.0274)	0.0358 (-0.0114, 0.0831)	0.0609 (0.0039, 0.1179)	-0.0198 (-0.0536, 0.0140)
	2021	0.0696 (0.0530, 0.0861)	0.0176 (-0.0072, 0.0425)	-0.0954 (-0.1160, -0.0748)	-0.0387 (-0.0716, -0.0058)	0.0103 (-0.0326, 0.0533)	0.0092 (-0.0294, 0.0478)	-0.0079 (-0.0455, 0.0296)	0.0644 (0.0260, 0.1028)	0.0073 (-0.0108, 0.0254)	0.0354 (-0.0118, 0.0827)	0.0616 (0.0045, 0.1188)	-0.0268 (-0.0685, 0.0150)
	1990	0.0450 (0.0273, 0.0627)	-0.0346 (-0.0455, -0.0236)	0.0257 (-0.0063, 0.0576)	0.0490 (0.0220, 0.0760)	-0.0322 (-0.0584, -0.0060)	0.1416 (0.1117, 0.1716)	-0.0199 (-0.0588, 0.0189)	0.0437 (-0.0089, 0.0963)	0.0263 (0.0050, 0.0476)	0.0617 (0.0225, 0.1009)	0.0893 (0.0056, 0.1730)	-0.0322 (-0.0589, -0.0056)
	1991	0.0390 (0.0209, 0.0571)	-0.0342 (-0.0451, -0.0233)	0.0257 (-0.0045, 0.0560)	0.0473 (0.0200, 0.0746)	-0.0979 (-0.1173, -0.0786)	-0.0332 (-0.0785, 0.0120)	-0.0189 (-0.0577, 0.0200)	0.0432 (-0.0080, 0.0945)	0.0255 (0.0046, 0.0465)	0.0633 (0.0241, 0.1025)	0.0933 (0.0091, 0.1776)	-0.0360 (-0.0631, -0.0089)
	1992	0.0380 (0.0199, 0.0560)	-0.0345 (-0.0455, -0.0236)	0.0003 (-0.0298, 0.0305)	-0.0117 (-0.0272, 0.0037)	-0.0972 (-0.1164, -0.0781)	-0.0327 (-0.0778, 0.0125)	-0.0195 (-0.0581, 0.0191)	0.0424 (-0.0076, 0.0924)	0.0254 (0.0047, 0.0462)	0.0641 (0.0249, 0.1032)	0.0947 (0.0113, 0.1782)	-0.0376 (-0.0643, -0.0108)
	1993	0.0377 (0.0196, 0.0558)	0.0222 (-0.0468, 0.0912)	0.0057 (-0.0251, 0.0365)	-0.0103 (-0.0255, 0.0049)	-0.0969 (-0.1162, -0.0776)	-0.0329 (-0.0787, 0.0128)	-0.0194 (-0.0578, 0.0190)	0.0415 (-0.0073, 0.0903)	0.0261 (0.0057, 0.0466)	0.0648 (0.0253, 0.1043)	0.0970 (0.0127, 0.1814)	-0.0388 (-0.0656, -0.0119)
	1994	0.0368 (0.0187, 0.0550)	0.0203 (-0.0219, 0.0626)	0.0113 (-0.0199, 0.0425)	-0.0077 (-0.0234, 0.0081)	-0.0975 (-0.1167, -0.0784)	-0.0329 (-0.0787, 0.0128)	-0.0191 (-0.0573, 0.0192)	0.0409 (-0.0069, 0.0887)	0.0259 (0.0055, 0.0463)	0.0655 (0.0259, 0.1050)	0.0984 (0.0137, 0.1832)	-0.0411 (-0.0680, -0.0141)
	1995	0.0374 (0.0195, 0.0553)	0.0206 (-0.0015, 0.0427)	0.0294 (-0.0034, 0.0622)	-0.0006 (-0.0161, 0.0150)	-0.0738 (-0.0974, -0.0502)	-0.0659 (-0.1099, -0.0220)	-0.0215 (-0.0598, 0.0168)	0.0400 (-0.0068, 0.0869)	0.0270 (0.0067, 0.0474)	0.0655 (0.0259, 0.1050)	0.0962 (0.0146, 0.1777)	-0.0413 (-0.0678, -0.0149)

Sex	Year	Location											
		Global	High SDI	High-middle SDI	Middle SDI	Low-middle SDI	Low SDI	AFR	EMR	EUR	AMR	SEAR	WPR
	1996	0.0419<br>(0.0241, 0.0596)	0.0223<br>(0.0005, 0.0440)	0.0354<br>(0.0032, 0.0676)	0.0032<br>(-0.0105, 0.0169)	-0.0308<br>(-0.0610, -0.0006)	-0.0664<br>(-0.1109, -0.0218)	-0.0210<br>(-0.0595, 0.0175)	0.0404<br>(-0.0056, 0.0865)	0.0270<br>(0.0067, 0.0474)	0.0648<br>(0.0250, 0.1047)	0.0977<br>(0.0149, 0.1805)	-0.0423<br>(-0.0689, -0.0157)
	1997	0.0425<br>(0.0247, 0.0603)	0.0224<br>(0.0007, 0.0441)	0.0373<br>(0.0071, 0.0674)	-0.0028<br>(-0.0164, 0.0107)	-0.0292<br>(-0.0594, 0.0010)	-0.0690<br>(-0.1143, -0.0237)	-0.0239<br>(-0.0619, 0.0141)	0.0408<br>(-0.0044, 0.0860)	0.0264<br>(0.0061, 0.0468)	0.0649<br>(0.0248, 0.1049)	0.1007<br>(0.0150, 0.1863)	-0.0420<br>(-0.0684, -0.0157)
	1998	0.0430<br>(0.0252, 0.0607)	0.0221<br>(0.0002, 0.0440)	0.0426<br>(0.0125, 0.0726)	-0.0083<br>(-0.0233, 0.0068)	-0.0260<br>(-0.0561, 0.0040)	-0.1060<br>(-0.1479, -0.0641)	-0.0255<br>(-0.0636, 0.0126)	0.0413<br>(-0.0028, 0.0854)	0.0264<br>(0.0061, 0.0468)	0.0651<br>(0.0247, 0.1055)	0.1009<br>(0.0153, 0.1866)	-0.0410<br>(-0.0673, -0.0148)
	1999	0.0462<br>(0.0285, 0.0639)	0.0223<br>(0.0032, 0.0414)	0.0530<br>(0.0249, 0.0812)	-0.0124<br>(-0.0288, 0.0039)	-0.0115<br>(-0.0421, 0.0191)	-0.1076<br>(-0.1506, -0.0646)	-0.0252<br>(-0.0634, 0.0130)	0.0413<br>(-0.0018, 0.0845)	0.0267<br>(0.0067, 0.0468)	0.0658<br>(0.0250, 0.1067)	0.1017<br>(0.0133, 0.1900)	-0.0400<br>(-0.0662, -0.0138)
	2000	0.0478<br>(0.0301, 0.0655)	0.0230<br>(0.0040, 0.0420)	0.0537<br>(0.0266, 0.0808)	-0.0118<br>(-0.0289, 0.0053)	-0.0004<br>(-0.0307, 0.0299)	-0.1195<br>(-0.1613, -0.0778)	-0.0248<br>(-0.0628, 0.0132)	0.0410<br>(-0.0009, 0.0829)	0.0269<br>(0.0071, 0.0467)	0.0659<br>(0.0247, 0.1071)	0.1023<br>(0.0120, 0.1927)	-0.0154<br>(-0.0459, 0.0151)
	2001	0.0475<br>(0.0299, 0.0650)	0.0207<br>(0.0012, 0.0402)	0.0601<br>(0.0350, 0.0852)	-0.0087<br>(-0.0271, 0.0097)	-0.0001<br>(-0.0308, 0.0307)	-0.1171<br>(-0.1582, -0.0761)	-0.0248<br>(-0.0627, 0.0132)	0.0407<br>(0.0003, 0.0812)	0.0264<br>(0.0067, 0.0461)	0.0653<br>(0.0239, 0.1067)	0.1006<br>(0.0114, 0.1897)	-0.0151<br>(-0.0455, 0.0153)
	2002	0.0486<br>(0.0311, 0.0660)	0.0206<br>(0.0022, 0.0390)	0.0743<br>(0.0531, 0.0955)	0.0110<br>(-0.0108, 0.0328)	0.0083<br>(-0.0218, 0.0383)	-0.1191<br>(-0.1594, -0.0789)	-0.0241<br>(-0.0617, 0.0135)	0.0400<br>(0.0008, 0.0792)	0.0267<br>(0.0070, 0.0463)	0.0634<br>(0.0217, 0.1051)	0.0994<br>(0.0109, 0.1880)	-0.0144<br>(-0.0447, 0.0160)
	2003	0.0491<br>(0.0318, 0.0665)	0.0200<br>(0.0014, 0.0387)	0.0743<br>(0.0532, 0.0953)	0.0106<br>(-0.0110, 0.0321)	0.0143<br>(-0.0155, 0.0441)	-0.1173<br>(-0.1578, -0.0768)	-0.0255<br>(-0.0631, 0.0122)	0.0391<br>(0.0006, 0.0777)	0.0271<br>(0.0077, 0.0465)	0.0639<br>(0.0225, 0.1053)	0.0985<br>(0.0112, 0.1858)	-0.0136<br>(-0.0441, 0.0169)
	2004	0.0487<br>(0.0313, 0.0661)	-0.0029<br>(-0.0294, 0.0236)	0.0613<br>(0.0381, 0.0845)	-0.0150<br>(-0.0361, 0.0061)	0.0157<br>(-0.0140, 0.0453)	-0.1160<br>(-0.1567, -0.0754)	-0.0255<br>(-0.0629, 0.0120)	0.0376<br>(-0.0004, 0.0755)	0.0270<br>(0.0079, 0.0461)	0.0626<br>(0.0212, 0.1041)	0.0990<br>(0.0132, 0.1847)	-0.0129<br>(-0.0435, 0.0178)
	2005	0.0527<br>(0.0356, 0.0697)	-0.0027<br>(-0.0273, 0.0219)	0.0600<br>(0.0356, 0.0844)	-0.0164<br>(-0.0373, 0.0045)	0.0435<br>(0.0151, 0.0719)	-0.1150<br>(-0.1553, -0.0746)	-0.0261<br>(-0.0634, 0.0112)	0.0367<br>(-0.0010, 0.0743)	0.0271<br>(0.0082, 0.0461)	0.0634<br>(0.0221, 0.1048)	0.0983<br>(0.0143, 0.1822)	-0.0120<br>(-0.0424, 0.0184)
	2006	0.0529<br>(0.0361, 0.0696)	0.0022<br>(-0.0215, 0.0259)	0.0615<br>(0.0369, 0.0861)	-0.0335<br>(-0.0558, -0.0111)	0.0504<br>(0.0223, 0.0786)	-0.1127<br>(-0.1525, -0.0730)	-0.0265<br>(-0.0635, 0.0105)	0.0359<br>(-0.0015, 0.0733)	0.0265<br>(0.0074, 0.0455)	0.0653<br>(0.0242, 0.1065)	0.0961<br>(0.0143, 0.1778)	-0.0109<br>(-0.0400, 0.0182)
	2007	0.0532<br>(0.0368, 0.0697)	0.0066<br>(-0.0174, 0.0306)	0.0634<br>(0.0394, 0.0874)	-0.0221<br>(-0.0446, 0.0003)	0.0624<br>(0.0356, 0.0892)	-0.1137<br>(-0.1522, -0.0752)	-0.0267<br>(-0.0640, 0.0107)	0.0354<br>(-0.0019, 0.0727)	0.0263<br>(0.0071, 0.0454)	0.0653<br>(0.0240, 0.1065)	0.0945<br>(0.0147, 0.1742)	-0.0101<br>(-0.0386, 0.0184)
	2008	0.0532<br>(0.0368, 0.0696)	0.0041<br>(-0.0244, 0.0325)	0.0103<br>(-0.0167, 0.0374)	-0.1087<br>(-0.1219, -0.0955)	-0.0144<br>(-0.0445, 0.0158)	-0.1157<br>(-0.1544, -0.0770)	-0.0266<br>(-0.0637, 0.0105)	0.0352<br>(-0.0018, 0.0722)	0.0257<br>(0.0064, 0.0449)	0.0645<br>(0.0230, 0.1059)	0.0935<br>(0.0146, 0.1723)	-0.0100<br>(-0.0385, 0.0184)
	2009	0.0560<br>(0.0396, 0.0724)	-0.0031<br>(-0.0307, 0.0246)	0.0126<br>(-0.0116, 0.0368)	-0.0362<br>(-0.0712, -0.0012)	-0.0160<br>(-0.0458, 0.0137)	-0.1380<br>(-0.1730, -0.1030)	-0.0264<br>(-0.0635, 0.0107)	0.0345<br>(-0.0024, 0.0714)	0.0252<br>(0.0058, 0.0446)	0.0635<br>(0.0220, 0.1049)	0.0938<br>(0.0138, 0.1737)	-0.0104<br>(-0.0391, 0.0183)
	2010	0.0574<br>(0.0411, 0.0737)	-0.0011<br>(-0.0287, 0.0264)	0.0134<br>(-0.0099, 0.0367)	-0.0099<br>(-0.0474, 0.0277)	0.1150<br>(0.0798, 0.1501)	-0.0448<br>(-0.0797, -0.0098)	-0.0269<br>(-0.0638, 0.0100)	0.0391<br>(0.0033, 0.0750)	0.0252<br>(0.0060, 0.0444)	0.0633<br>(0.0218, 0.1049)	0.0926<br>(0.0127, 0.1725)	-0.0095<br>(-0.0379, 0.0190)
	2011	0.0587<br>(0.0425, 0.0749)	0.0071<br>(-0.0198, 0.0340)	0.0153<br>(-0.0082, 0.0387)	0.0241<br>(-0.0119, 0.0601)	0.1133<br>(0.0764, 0.1501)	-0.0580<br>(-0.0940, -0.0220)	-0.0283<br>(-0.0650, 0.0084)	0.0390<br>(0.0030, 0.0750)	0.0259<br>(0.0066, 0.0453)	0.0854<br>(0.0486, 0.1222)	0.0934<br>(0.0146, 0.1721)	-0.0062<br>(-0.0340, 0.0217)
	2012	0.0591<br>(0.0430, 0.0753)	0.0118<br>(-0.0123, 0.0359)	0.0109<br>(-0.0130, 0.0349)	0.0473<br>(0.0087, 0.0859)	0.1190<br>(0.0845, 0.1535)	-0.0547<br>(-0.0922, -0.0173)	-0.0274<br>(-0.0642, 0.0095)	0.0393<br>(0.0026, 0.0760)	0.0259<br>(0.0066, 0.0453)	0.0853<br>(0.0485, 0.1221)	0.0940<br>(0.0172, 0.1707)	-0.0042<br>(-0.0314, 0.0230)
	2013	0.0576<br>(0.0417, 0.0736)	0.0304<br>(0.0053, 0.0556)	0.0307<br>(0.0087, 0.0528)	0.0482<br>(0.0084, 0.0879)	0.1051<br>(0.0713, 0.1390)	-0.0631<br>(-0.1020, -0.0242)	-0.0280<br>(-0.0649, 0.0089)	0.0389<br>(0.0023, 0.0755)	0.0251<br>(0.0057, 0.0445)	0.0834<br>(0.0463, 0.1206)	0.0891<br>(0.0186, 0.1595)	-0.0028<br>(-0.0297, 0.0240)

Sex	Year	Location											
		Global	High SDI	High-middle SDI	Middle SDI	Low-middle SDI	Low SDI	AFR	EMR	EUR	AMR	SEAR	WPR
	2014	0.0569	0.0301	0.0280	0.0644	0.0886	-0.0639	-0.0288	0.0390	0.0235	0.0826	0.0886	-0.0032
		(0.0406, 0.0731)	(0.0055, 0.0547)	(0.0054, 0.0506)	(0.0278, 0.1010)	(0.0507, 0.1266)	(-0.1032, -0.0246)	(-0.0661, 0.0085)	(0.0021, 0.0759)	(0.0039, 0.0431)	(0.0453, 0.1199)	(0.0196, 0.1576)	(-0.0308, 0.0245)
	2015	0.0565	0.0296	0.0174	0.0699	0.0671	-0.0790	-0.0287	0.0386	0.0223	0.0567	0.0867	-0.0029
		(0.0402, 0.0727)	(0.0054, 0.0538)	(-0.0068, 0.0416)	(0.0363, 0.1035)	(0.0171, 0.1171)	(-0.1173, -0.0407)	(-0.0664, 0.0090)	(0.0012, 0.0760)	(0.0024, 0.0423)	(0.0137, 0.0996)	(0.0190, 0.1544)	(-0.0304, 0.0246)
	2016	0.0558	0.0325	0.0233	0.0687	0.0482	-0.0832	-0.0295	0.0382	0.0229	0.0558	0.0856	-0.0035
		(0.0395, 0.0722)	(0.0093, 0.0557)	(-0.0013, 0.0480)	(0.0366, 0.1008)	(-0.0003, 0.0966)	(-0.1236, -0.0427)	(-0.0675, 0.0085)	(0.0007, 0.0757)	(0.0025, 0.0433)	(0.0127, 0.0990)	(0.0183, 0.1529)	(-0.0316, 0.0246)
	2017	0.0554	0.0329	-0.0102	0.0753	0.0226	-0.0758	-0.0304	0.0359	0.0118	0.0566	0.0827	-0.0042
		(0.0393, 0.0716)	(0.0100, 0.0558)	(-0.0373, 0.0168)	(0.0470, 0.1035)	(-0.0232, 0.0684)	(-0.1206, -0.0309)	(-0.0686, 0.0077)	(-0.0009, 0.0727)	(-0.0100, 0.0337)	(0.0135, 0.0998)	(0.0174, 0.1481)	(-0.0324, 0.0241)
	2018	0.0551	0.0350	0.0010	0.0862	0.0205	-0.0796	-0.0303	0.0354	0.0131	0.0565	0.0816	-0.0054
		(0.0389, 0.0713)	(0.0121, 0.0578)	(-0.0236, 0.0256)	(0.0591, 0.1133)	(-0.0253, 0.0663)	(-0.1245, -0.0347)	(-0.0686, 0.0080)	(-0.0017, 0.0724)	(-0.0093, 0.0355)	(0.0133, 0.0997)	(0.0174, 0.1459)	(-0.0342, 0.0235)
	2019	0.0541	0.0324	-0.0005	0.0864	0.0213	-0.0199	-0.0309	0.0346	0.0131	0.0481	0.0807	-0.0062
		(0.0379, 0.0703)	(0.0090, 0.0557)	(-0.0245, 0.0234)	(0.0591, 0.1137)	(-0.0192, 0.0618)	(-0.0656, 0.0258)	(-0.0695, 0.0076)	(-0.0025, 0.0716)	(-0.0096, 0.0357)	(0.0038, 0.0924)	(0.0171, 0.1442)	(-0.0352, 0.0228)
	2020	0.0525	0.0319	-0.1083	-0.0274	-0.0202	-0.0061	-0.0296	0.0341	0.0133	0.0478	0.0650	-0.0075
		(0.0365, 0.0686)	(0.0100, 0.0538)	(-0.1245, -0.0920)	(-0.0582, 0.0033)	(-0.0570, 0.0167)	(-0.0546, 0.0423)	(-0.0687, 0.0096)	(-0.0024, 0.0706)	(-0.0089, 0.0355)	(0.0034, 0.0923)	(-0.0038, 0.1338)	(-0.0372, 0.0222)
	2021	0.0557	0.0369	-0.1052	-0.0417	-0.0267	-0.0053	-0.0312	0.0333	0.0127	0.0471	0.0650	-0.0129
		(0.0383, 0.0731)	(0.0153, 0.0585)	(-0.1289, -0.0815)	(-0.0707, -0.0126)	(-0.0641, 0.0107)	(-0.0534, 0.0428)	(-0.0698, 0.0074)	(-0.0024, 0.0689)	(-0.0093, 0.0347)	(0.0023, 0.0918)	(-0.0047, 0.1348)	(-0.0499, 0.0241)

Notes: ADRDs, Alzheimer's disease and related dementia; SDI, Socio-demographic Index; WHO, World Health Organization; AFR, African Region; EMR, Eastern Mediterranean Region; AMR, Region of the Americas; EUR, European Region; SEAR, South-East Asia Region; WPR, Western Pacific Region.

Table S6. The year trends of slope index of inequality for care quality of ADRDs by sex, SDI quantiles and WHO regions, from 1990 to 2021

Sex	Year	Location											
		Global	High SDI	High-middle SDI	Middle SDI	Low-middle SDI	Low SDI	AFR	EMR	EUR	AMR	SEAR	WPR
Both													
	1990	14.26 (7.49, 21.03)	-7.51 (-15.24, 0.23)	-2.87 (-17.23, 11.50)	-8.69 (-24.52, 7.13)	1.67 (-9.60, 12.94)	13.29 (-1.21, 27.78)	13.72 (3.27, 24.17)	-0.19 (-14.35, 13.98)	-10.04 (-16.19, -3.90)	5.06 (-6.70, 16.82)	-9.74 (-27.56, 8.08)	-9.83 (-24.37, 4.72)
	1991	14.68 (7.85, 21.51)	-7.57 (-15.40, 0.26)	-0.19 (-12.92, 12.55)	-9.78 (-27.17, 7.62)	2.69 (-18.99, 24.37)	11.09 (1.64, 20.54)	12.05 (1.54, 22.57)	1.15 (-14.04, 16.35)	-10.47 (-16.66, -4.28)	5.45 (-5.97, 16.86)	-7.58 (-25.80, 10.63)	-9.32 (-22.96, 4.32)
	1992	15.32 (8.54, 22.09)	-7.36 (-15.67, 0.95)	7.60 (-9.20, 24.40)	-0.93 (-15.15, 13.30)	7.48 (-6.03, 20.99)	12.21 (2.74, 21.67)	11.39 (0.96, 21.82)	1.78 (-12.99, 16.55)	-10.76 (-16.90, -4.63)	8.43 (-3.53, 20.39)	-7.80 (-26.04, 10.44)	-7.63 (-19.87, 4.61)
	1993	15.74 (8.97, 22.51)	-0.82 (-14.21, 12.58)	7.40 (-9.32, 24.12)	2.17 (-12.15, 16.48)	8.16 (-2.58, 18.90)	11.79 (1.71, 21.88)	11.85 (1.51, 22.19)	2.88 (-12.35, 18.10)	-11.04 (-17.17, -4.91)	7.24 (-4.68, 19.17)	-7.56 (-25.98, 10.85)	-8.16 (-19.62, 3.31)
	1994	15.92 (9.25, 22.60)	6.99 (-16.56, 30.55)	6.20 (-10.89, 23.28)	-3.69 (-17.48, 10.10)	4.03 (-5.82, 13.88)	10.69 (0.77, 20.61)	11.68 (1.75, 21.62)	3.60 (-11.61, 18.80)	-11.59 (-17.81, -5.36)	7.56 (-4.84, 19.97)	-7.31 (-25.81, 11.19)	-7.78 (-19.46, 3.90)
	1995	15.87 (9.15, 22.58)	14.33 (-24.48, 53.14)	3.03 (-14.22, 20.29)	-16.32 (-29.71, -2.93)	5.09 (-3.80, 13.97)	12.69 (1.48, 23.89)	12.58 (2.31, 22.84)	3.34 (-10.98, 17.66)	-12.89 (-19.07, -6.71)	7.41 (-4.41, 19.23)	-6.58 (-26.17, 13.01)	-7.22 (-18.88, 4.45)
	1996	16.16 (9.42, 22.89)	17.91 (-10.30, 46.11)	3.76 (-13.01, 20.54)	-5.26 (-18.35, 7.83)	2.63 (-5.62, 10.88)	11.22 (0.01, 22.42)	12.42 (2.20, 22.65)	2.93 (-11.21, 17.06)	-12.98 (-19.10, -6.85)	9.94 (-3.00, 22.87)	-6.40 (-26.03, 13.23)	-2.65 (-14.41, 9.11)
	1997	16.52 (9.88, 23.17)	16.29 (-11.44, 44.03)	2.31 (-13.60, 18.23)	-5.41 (-21.65, 10.82)	2.77 (-5.40, 10.95)	11.60 (0.38, 22.83)	11.78 (1.06, 22.50)	2.54 (-11.93, 17.01)	-13.75 (-19.66, -7.84)	9.39 (-3.55, 22.33)	-5.78 (-25.31, 13.75)	-2.22 (-13.18, 8.74)
	1998	16.96 (10.31, 23.62)	2.05 (-19.63, 23.73)	-0.29 (-19.46, 18.88)	0.50 (-12.98, 13.98)	8.28 (-0.63, 17.20)	14.21 (3.14, 25.28)	10.80 (0.06, 21.54)	3.21 (-12.38, 18.79)	-14.43 (-20.59, -8.28)	8.35 (-4.10, 20.80)	-5.06 (-24.77, 14.65)	-0.63 (-11.73, 10.47)
	1999	16.87 (10.21, 23.53)	-9.43 (-33.62, 14.76)	-9.05 (-24.52, 6.42)	5.10 (-8.15, 18.35)	9.71 (0.80, 18.62)	16.17 (3.88, 28.45)	9.95 (-0.59, 20.49)	6.52 (-8.72, 21.77)	-15.13 (-21.33, -8.94)	8.18 (-4.10, 20.46)	-4.00 (-23.97, 15.98)	-0.72 (-11.67, 10.23)
	2000	17.61 (10.90, 24.33)	-9.65 (-33.63, 14.32)	-8.51 (-24.03, 7.01)	13.85 (-1.26, 28.95)	10.28 (1.82, 18.74)	15.39 (2.62, 28.17)	9.12 (-1.40, 19.64)	6.50 (-9.06, 22.06)	-16.00 (-21.97, -10.02)	9.59 (-2.41, 21.59)	-3.46 (-23.72, 16.80)	-1.39 (-12.33, 9.55)
	2001	17.71 (11.11, 24.31)	-10.87 (-35.40, 13.66)	-15.08 (-33.29, 3.13)	10.22 (-7.54, 27.98)	11.12 (2.37, 19.88)	14.66 (2.00, 27.32)	7.58 (-2.76, 17.92)	6.18 (-9.29, 21.64)	-16.33 (-22.47, -10.18)	9.20 (-2.85, 21.25)	-1.93 (-21.85, 17.99)	-0.93 (-11.25, 9.40)
	2002	18.39 (11.81, 24.98)	-11.67 (-29.35, 6.00)	-30.44 (-48.53, -12.34)	5.20 (-8.39, 18.79)	12.03 (3.45, 20.61)	16.94 (3.46, 30.41)	7.08 (-3.41, 17.58)	7.49 (-7.77, 22.75)	-17.09 (-23.52, -10.67)	7.96 (-4.07, 19.99)	-0.65 (-20.37, 19.07)	-0.52 (-10.95, 9.91)
	2003	18.67 (12.07, 25.27)	-12.97 (-30.75, 4.81)	-29.72 (-47.02, -12.42)	4.93 (-7.63, 17.49)	12.06 (2.95, 21.17)	17.36 (3.06, 31.67)	6.49 (-3.81, 16.79)	8.17 (-6.84, 23.17)	-17.52 (-23.98, -11.06)	5.27 (-6.02, 16.57)	1.01 (-18.00, 20.02)	-0.23 (-10.73, 10.28)
	2004	19.47 (12.84, 26.10)	-3.18 (-23.86, 17.49)	-15.17 (-31.58, 1.25)	10.71 (-0.74, 22.15)	11.84 (2.98, 20.71)	22.15 (7.62, 36.68)	7.00 (-3.60, 17.61)	9.36 (-6.82, 25.53)	-17.11 (-23.63, -10.59)	5.68 (-6.01, 17.38)	1.66 (-19.18, 22.49)	0.51 (-9.23, 10.25)
	2005	19.30 (12.54, 26.06)	-2.37 (-15.88, 11.14)	-15.07 (-31.29, 1.14)	7.28 (-2.43, 16.99)	9.39 (-0.79, 19.58)	24.46 (8.92, 40.01)	7.67 (-3.20, 18.54)	9.87 (-6.41, 26.15)	-17.47 (-23.66, -11.28)	5.98 (-5.63, 17.59)	2.52 (-18.42, 23.45)	1.42 (-8.07, 10.90)

Sex	Year	Location											
		**Global**	**High SDI**	**High-middle SDI**	**Middle SDI**	**Low-middle SDI**	**Low SDI**	**AFR**	**EMR**	**EUR**	**AMR**	**SEAR**	**WPR**
	2006	20.08 (13.31, 26.85)	0.53 (-13.27, 14.33)	-13.95 (-32.48, 4.58)	3.67 (-7.58, 14.91)	10.36 (-0.67, 21.39)	26.36 (10.39, 42.33)	9.07 (-2.27, 20.41)	11.51 (-4.95, 27.96)	-17.77 (-24.16, -11.39)	6.02 (-5.78, 17.81)	3.52 (-15.41, 22.46)	1.95 (-7.09, 10.98)
	2007	19.66 (13.02, 26.30)	-0.19 (-12.21, 11.82)	-16.06 (-34.72, 2.59)	-3.74 (-17.39, 9.90)	11.29 (-2.74, 25.32)	30.89 (14.86, 46.92)	9.04 (-1.84, 19.93)	12.24 (-3.87, 28.35)	-17.46 (-24.12, -10.79)	4.25 (-7.64, 16.15)	4.71 (-16.95, 26.37)	2.12 (-6.43, 10.66)
	2008	20.72 (14.05, 27.40)	-1.50 (-18.61, 15.61)	-5.92 (-17.94, 6.10)	-4.20 (-23.22, 14.81)	21.59 (8.54, 34.64)	36.56 (19.11, 54.02)	9.84 (-1.38, 21.07)	13.17 (-2.50, 28.85)	-17.36 (-23.95, -10.77)	3.83 (-7.60, 15.26)	5.06 (-15.73, 25.84)	0.89 (-8.74, 10.52)
	2009	20.91 (14.02, 27.80)	0.43 (-13.69, 14.55)	-6.51 (-18.29, 5.27)	1.37 (-8.15, 10.89)	23.23 (9.40, 37.06)	38.63 (17.08, 60.17)	9.96 (-1.36, 21.28)	14.47 (-0.54, 29.48)	-17.65 (-24.71, -10.60)	2.53 (-8.20, 13.26)	5.66 (-14.11, 25.43)	1.23 (-8.15, 10.62)
	2010	21.33 (14.51, 28.16)	2.44 (-10.61, 15.50)	-7.33 (-17.83, 3.18)	-0.14 (-8.82, 8.54)	42.44 (15.19, 69.68)	17.25 (3.31, 31.19)	10.37 (-1.16, 21.91)	19.03 (4.09, 33.96)	-17.93 (-25.19, -10.66)	2.07 (-8.21, 12.36)	7.04 (-12.90, 26.98)	1.48 (-7.53, 10.49)
	2011	21.68 (14.72, 28.64)	-1.21 (-12.92, 10.50)	-5.38 (-16.23, 5.48)	-4.76 (-12.66, 3.14)	8.57 (-18.63, 35.77)	16.35 (3.09, 29.61)	11.40 (-0.45, 23.25)	19.99 (5.21, 34.76)	-18.68 (-26.03, -11.33)	0.79 (-8.96, 10.53)	7.86 (-11.63, 27.36)	2.00 (-6.93, 10.93)
	2012	21.50 (14.57, 28.43)	-4.38 (-13.98, 5.23)	-1.82 (-13.07, 9.44)	-3.64 (-10.66, 3.37)	2.58 (-20.52, 25.68)	13.60 (-0.15, 27.34)	11.43 (-0.32, 23.18)	20.53 (5.97, 35.08)	-19.94 (-26.56, -13.32)	0.92 (-9.35, 11.18)	8.06 (-11.95, 28.07)	2.23 (-6.24, 10.70)
	2013	22.01 (15.03, 28.99)	-3.27 (-13.72, 7.18)	0.70 (-8.75, 10.16)	-3.26 (-10.53, 4.01)	4.37 (-14.38, 23.11)	7.60 (-6.98, 22.18)	12.07 (0.89, 23.26)	20.92 (6.30, 35.53)	-19.96 (-26.93, -12.99)	0.31 (-9.91, 10.53)	9.39 (-12.60, 31.39)	1.94 (-7.44, 11.32)
	2014	22.21 (15.29, 29.13)	-4.40 (-14.26, 5.46)	-0.41 (-10.21, 9.38)	-2.89 (-10.90, 5.12)	0.97 (-16.54, 18.47)	6.60 (-9.38, 22.58)	12.00 (-0.02, 24.02)	21.64 (6.85, 36.43)	-20.28 (-27.35, -13.21)	-0.15 (-10.21, 9.92)	10.18 (-10.83, 31.19)	1.94 (-8.17, 12.05)
	2015	22.48 (15.60, 29.35)	-4.58 (-14.01, 4.85)	1.42 (-8.72, 11.57)	-2.65 (-11.32, 6.01)	1.00 (-14.09, 16.10)	11.10 (-5.30, 27.50)	12.06 (0.14, 23.99)	22.98 (8.10, 37.87)	-20.27 (-27.55, -12.99)	1.09 (-8.77, 10.96)	11.04 (-10.25, 32.34)	2.05 (-7.79, 11.89)
	2016	22.95 (16.03, 29.88)	-8.00 (-18.03, 2.03)	-2.75 (-13.65, 8.15)	-2.90 (-12.05, 6.26)	7.12 (-8.92, 23.16)	7.24 (-8.50, 22.98)	11.74 (-0.42, 23.90)	23.37 (7.94, 38.80)	-20.16 (-27.36, -12.96)	0.74 (-8.52, 10.00)	11.01 (-10.37, 32.38)	2.33 (-7.66, 12.32)
	2017	23.71 (16.73, 30.69)	-9.65 (-19.48, 0.18)	-7.77 (-20.86, 5.31)	-3.53 (-12.15, 5.08)	9.88 (-5.08, 24.84)	8.02 (-6.32, 22.36)	12.29 (-0.01, 24.59)	24.78 (8.85, 40.71)	-19.48 (-26.68, -12.28)	0.60 (-8.97, 10.16)	12.26 (-9.47, 33.99)	2.32 (-7.96, 12.59)
	2018	24.53 (17.51, 31.55)	-11.55 (-21.49, -1.61)	-8.25 (-19.93, 3.43)	1.87 (-11.00, 14.74)	6.84 (-8.65, 22.33)	12.34 (-4.44, 29.13)	11.80 (-0.86, 24.46)	26.10 (10.03, 42.16)	-19.47 (-26.92, -12.03)	1.00 (-8.46, 10.46)	13.49 (-7.90, 34.88)	2.74 (-7.64, 13.12)
	2019	25.69 (18.66, 32.72)	-11.09 (-21.80, -0.39)	-6.78 (-18.26, 4.70)	7.34 (-11.75, 26.43)	13.83 (-1.17, 28.82)	14.45 (-2.83, 31.73)	12.00 (-0.67, 24.66)	27.62 (11.45, 43.79)	-18.53 (-26.00, -11.06)	2.79 (-7.41, 12.99)	14.15 (-7.58, 35.88)	3.14 (-7.42, 13.71)
	2020	26.40 (19.33, 33.47)	-10.36 (-20.46, -0.25)	-1.57 (-30.42, 27.29)	15.16 (1.71, 28.60)	17.98 (4.13, 31.83)	15.83 (-3.36, 35.02)	11.52 (-1.11, 24.16)	27.19 (11.31, 43.06)	-17.01 (-24.41, -9.62)	4.01 (-7.15, 15.17)	17.65 (-12.31, 47.60)	5.03 (-5.31, 15.37)
	2021	25.96 (18.78, 33.14)	-12.12 (-21.96, -2.28)	-3.14 (-17.18, 10.90)	9.06 (-2.06, 20.17)	16.89 (1.49, 32.30)	14.71 (-4.27, 33.68)	14.37 (1.90, 26.85)	26.65 (9.97, 43.33)	-16.62 (-24.12, -9.12)	2.25 (-7.77, 12.26)	17.21 (-5.72, 40.14)	2.64 (-7.35, 12.64)
Female	1990	13.64 (6.96, 20.32)	-7.17 (-16.13, 1.79)	-1.68 (-15.33, 11.96)	-8.27 (-23.85, 7.32)	1.50 (-9.55, 12.55)	12.84 (-1.12, 26.81)	11.67 (0.76, 22.58)	0.94 (-13.17, 15.05)	-8.91 (-14.08, -3.73)	4.81 (-6.92, 16.53)	-8.78 (-24.84, 7.28)	-10.74 (-23.91, 2.43)

Sex	Year	Location											
		Global	High SDI	High-middle SDI	Middle SDI	Low-middle SDI	Low SDI	AFR	EMR	EUR	AMR	SEAR	WPR
	1991	13.94 (7.24, 20.65)	-7.34 (-16.36, 1.68)	0.61 (-11.15, 12.37)	-10.22 (-29.26, 8.82)	2.41 (-18.18, 23.00)	10.82 (1.50, 20.14)	10.16 (-0.32, 20.64)	1.51 (-12.80, 15.81)	-9.44 (-14.63, -4.24)	3.86 (-6.99, 14.71)	-6.11 (-22.64, 10.43)	-10.14 (-22.68, 2.40)
	1992	14.61 (7.98, 21.23)	-7.23 (-16.59, 2.14)	7.23 (-8.55, 23.01)	-0.68 (-17.15, 15.79)	7.09 (-5.41, 19.60)	11.77 (2.47, 21.06)	9.57 (-0.70, 19.84)	3.69 (-11.15, 18.53)	-9.64 (-14.91, -4.37)	6.04 (-6.07, 18.15)	-6.24 (-22.66, 10.19)	-8.67 (-20.18, 2.84)
	1993	15.10 (8.62, 21.58)	-1.10 (-19.51, 17.31)	8.60 (-8.36, 25.55)	2.11 (-13.14, 17.36)	7.53 (-2.28, 17.34)	11.40 (1.27, 21.52)	10.26 (-0.08, 20.60)	5.00 (-10.56, 20.56)	-9.90 (-15.32, -4.48)	5.21 (-6.66, 17.07)	-6.19 (-21.24, 8.85)	-9.19 (-19.75, 1.37)
	1994	15.41 (8.85, 21.98)	5.07 (-14.47, 24.60)	7.47 (-8.50, 23.44)	-2.83 (-15.94, 10.29)	4.72 (-4.77, 14.20)	10.37 (0.10, 20.64)	10.11 (0.13, 20.10)	5.61 (-9.81, 21.03)	-10.37 (-15.72, -5.03)	5.09 (-7.73, 17.90)	-5.50 (-22.20, 11.21)	-8.63 (-19.16, 1.91)
	1995	15.52 (8.97, 22.06)	12.36 (-24.50, 49.22)	1.48 (-13.44, 16.41)	-15.73 (-29.13, -2.32)	5.53 (-3.09, 14.14)	12.88 (1.78, 23.99)	11.38 (1.28, 21.47)	2.97 (-9.80, 15.74)	-11.91 (-17.24, -6.59)	6.64 (-6.99, 20.26)	-5.27 (-21.61, 11.08)	-8.14 (-18.43, 2.15)
	1996	16.01 (9.55, 22.47)	16.12 (-11.27, 43.51)	2.41 (-12.57, 17.38)	-3.95 (-16.60, 8.71)	2.52 (-5.75, 10.79)	11.56 (0.79, 22.33)	11.64 (1.41, 21.87)	2.84 (-9.63, 15.30)	-12.06 (-17.39, -6.74)	7.17 (-6.29, 20.64)	-5.34 (-21.59, 10.91)	-4.06 (-14.52, 6.40)
	1997	16.27 (9.73, 22.81)	14.33 (-12.40, 41.06)	1.79 (-11.71, 15.30)	-3.91 (-19.60, 11.77)	2.00 (-5.99, 10.00)	11.86 (0.79, 22.93)	11.55 (1.03, 22.07)	3.21 (-10.01, 16.42)	-12.65 (-18.05, -7.26)	6.12 (-7.44, 19.68)	-4.94 (-21.06, 11.18)	-3.57 (-14.06, 6.91)
	1998	16.90 (10.44, 23.36)	0.66 (-20.36, 21.68)	-0.63 (-17.45, 16.18)	0.54 (-12.21, 13.29)	7.52 (-1.73, 16.77)	14.05 (2.94, 25.16)	10.43 (-0.16, 21.02)	3.31 (-10.73, 17.35)	-12.94 (-18.55, -7.33)	5.18 (-8.90, 19.25)	-4.04 (-20.31, 12.24)	-1.25 (-11.71, 9.21)
	1999	16.94 (10.44, 23.45)	-12.30 (-38.10, 13.50)	-7.82 (-23.32, 7.68)	4.85 (-8.71, 18.42)	9.12 (-0.13, 18.37)	15.69 (3.41, 27.98)	9.76 (-0.68, 20.19)	6.44 (-8.33, 21.20)	-13.50 (-19.04, -7.96)	5.29 (-7.97, 18.55)	-2.91 (-19.75, 13.93)	-0.93 (-12.06, 10.19)
	2000	17.87 (11.39, 24.35)	-12.75 (-38.19, 12.70)	-7.70 (-22.14, 6.74)	16.13 (0.88, 31.37)	9.66 (1.11, 18.20)	15.12 (1.70, 28.54)	8.88 (-1.69, 19.45)	5.69 (-9.29, 20.67)	-14.27 (-19.77, -8.78)	8.26 (-4.58, 21.10)	-2.21 (-19.28, 14.85)	-1.99 (-12.24, 8.26)
	2001	17.99 (11.44, 24.55)	-19.59 (-49.08, 9.91)	-13.61 (-29.68, 2.45)	10.80 (-6.19, 27.80)	10.28 (1.45, 19.12)	14.58 (1.91, 27.26)	7.39 (-3.81, 18.58)	5.39 (-9.61, 20.40)	-14.58 (-20.27, -8.90)	6.67 (-6.00, 19.35)	-0.74 (-17.39, 15.91)	-1.98 (-10.81, 6.85)
	2002	18.70 (12.18, 25.22)	-10.05 (-27.65, 7.55)	-28.22 (-46.44, -10.00)	6.07 (-6.69, 18.82)	10.99 (2.10, 19.88)	16.31 (2.80, 29.83)	6.68 (-3.95, 17.31)	6.55 (-8.13, 21.23)	-15.20 (-20.94, -9.47)	6.45 (-6.28, 19.19)	0.64 (-15.86, 17.14)	-1.79 (-10.51, 6.92)
	2003	18.73 (12.30, 25.16)	-10.17 (-26.80, 6.45)	-27.76 (-45.08, -10.43)	5.99 (-6.80, 18.78)	11.06 (2.08, 20.04)	16.55 (2.43, 30.66)	6.38 (-4.59, 17.34)	6.39 (-8.03, 20.80)	-15.74 (-21.56, -9.91)	5.70 (-7.62, 19.03)	2.25 (-13.47, 17.97)	-1.53 (-10.10, 7.04)
	2004	19.43 (12.98, 25.89)	-1.68 (-22.98, 19.62)	-11.56 (-28.23, 5.10)	11.80 (-0.87, 24.47)	10.73 (2.15, 19.31)	21.50 (6.68, 36.33)	6.41 (-4.48, 17.30)	7.43 (-7.63, 22.49)	-15.57 (-21.42, -9.72)	5.60 (-6.91, 18.12)	2.89 (-12.40, 18.19)	-1.02 (-10.45, 8.41)
	2005	19.31 (12.78, 25.84)	-1.89 (-14.70, 10.93)	-11.72 (-29.82, 6.38)	9.12 (-2.12, 20.35)	8.70 (-1.30, 18.70)	23.68 (7.16, 40.19)	7.18 (-3.95, 18.30)	8.03 (-8.52, 24.57)	-15.97 (-21.84, -10.10)	5.86 (-6.13, 17.85)	3.27 (-13.41, 19.96)	-0.19 (-9.19, 8.81)
	2006	20.03 (13.37, 26.70)	0.60 (-12.86, 14.06)	-12.82 (-32.22, 6.59)	5.24 (-8.46, 18.94)	9.73 (-0.79, 20.26)	25.00 (8.73, 41.27)	7.89 (-2.88, 18.67)	9.23 (-7.23, 25.69)	-16.22 (-22.16, -10.29)	5.36 (-6.05, 16.77)	4.36 (-12.74, 21.46)	0.19 (-8.42, 8.79)
	2007	19.53 (12.96, 26.10)	0.09 (-13.52, 13.69)	-13.73 (-34.18, 6.71)	1.39 (-15.06, 17.84)	10.78 (-2.72, 24.28)	29.20 (12.90, 45.50)	7.59 (-3.03, 18.20)	10.47 (-6.96, 27.90)	-16.04 (-22.10, -9.98)	2.58 (-7.36, 12.52)	5.89 (-12.18, 23.97)	-0.06 (-8.95, 8.83)
	2008	20.56 (14.04, 27.07)	-2.64 (-20.16, 14.89)	-4.88 (-17.01, 7.25)	-3.33 (-21.41, 14.75)	20.31 (6.94, 33.69)	34.66 (17.36, 51.96)	8.18 (-2.44, 18.81)	11.43 (-6.43, 29.29)	-15.76 (-22.35, -9.16)	2.60 (-7.79, 12.99)	7.38 (-9.33, 24.08)	-1.46 (-10.07, 7.14)

Sex	Year	Location											
		Global	High SDI	High-middle SDI	Middle SDI	Low-middle SDI	Low SDI	AFR	EMR	EUR	AMR	SEAR	WPR
	2009	20.83 (14.19, 27.47)	0.62 (-14.81, 16.05)	-5.65 (-17.40, 6.10)	0.60 (-8.34, 9.53)	21.61 (7.72, 35.51)	36.26 (13.84, 58.68)	7.83 (-2.54, 18.20)	12.93 (-6.04, 31.89)	-15.53 (-21.90, -9.15)	0.82 (-8.04, 9.69)	8.11 (-9.73, 25.94)	-0.98 (-9.60, 7.64)
	2010	21.42 (14.79, 28.05)	2.62 (-10.06, 15.30)	-6.51 (-17.75, 4.72)	-0.85 (-9.69, 8.00)	39.97 (14.03, 65.90)	17.00 (2.28, 31.71)	8.14 (-2.39, 18.66)	21.47 (3.86, 39.09)	-15.63 (-22.03, -9.23)	1.31 (-8.00, 10.62)	8.87 (-8.30, 26.04)	-1.16 (-9.82, 7.50)
	2011	21.78 (15.10, 28.46)	-0.50 (-11.67, 10.67)	-5.14 (-15.75, 5.47)	-5.93 (-13.32, 1.46)	9.29 (-17.01, 35.60)	15.72 (2.14, 29.31)	9.21 (-1.49, 19.91)	22.44 (4.41, 40.46)	-16.21 (-22.89, -9.52)	0.26 (-8.59, 9.11)	9.73 (-7.19, 26.65)	-0.83 (-8.98, 7.32)
	2012	21.63 (14.85, 28.41)	-3.67 (-13.04, 5.70)	0.07 (-11.33, 11.47)	-4.85 (-11.76, 2.07)	2.44 (-20.29, 25.18)	12.47 (-1.72, 26.66)	9.18 (-1.86, 20.21)	23.04 (5.68, 40.40)	-17.34 (-23.79, -10.88)	0.51 (-9.32, 10.34)	10.10 (-7.57, 27.77)	-0.68 (-9.01, 7.64)
	2013	22.26 (15.39, 29.12)	-2.45 (-12.13, 7.23)	2.46 (-6.79, 11.71)	-5.02 (-12.01, 1.97)	3.90 (-14.75, 22.55)	4.32 (-10.41, 19.05)	9.85 (-0.83, 20.52)	22.99 (5.26, 40.71)	-17.57 (-24.39, -10.76)	-0.86 (-10.33, 8.60)	11.58 (-8.10, 31.26)	-0.54 (-8.65, 7.56)
	2014	22.34 (15.55, 29.12)	-3.91 (-13.22, 5.41)	1.58 (-7.80, 10.95)	-4.90 (-12.70, 2.90)	1.15 (-15.81, 18.11)	3.96 (-11.99, 19.90)	10.00 (-1.28, 21.29)	23.75 (5.59, 41.91)	-17.66 (-24.49, -10.83)	-1.40 (-10.40, 7.61)	11.59 (-8.70, 31.89)	-0.03 (-8.20, 8.13)
	2015	22.62 (15.72, 29.51)	-4.43 (-13.32, 4.46)	2.86 (-7.33, 13.05)	-4.07 (-12.56, 4.41)	1.06 (-13.54, 15.67)	8.72 (-7.68, 25.12)	10.29 (-1.30, 21.89)	25.42 (6.35, 44.49)	-17.28 (-23.60, -10.96)	-0.61 (-8.62, 7.40)	12.62 (-8.11, 33.36)	0.27 (-7.96, 8.50)
	2016	23.17 (16.31, 30.03)	-7.57 (-16.71, 1.56)	-1.30 (-12.47, 9.87)	-4.36 (-13.21, 4.49)	8.77 (-7.74, 25.29)	6.75 (-7.83, 21.33)	10.43 (-1.46, 22.33)	25.83 (7.12, 44.53)	-17.24 (-23.84, -10.64)	-0.46 (-8.34, 7.41)	12.64 (-8.29, 33.57)	0.52 (-7.89, 8.93)
	2017	23.96 (17.05, 30.87)	-9.62 (-19.38, 0.15)	-7.04 (-21.05, 6.97)	-4.80 (-13.49, 3.90)	11.54 (-4.15, 27.24)	8.25 (-6.29, 22.78)	10.86 (-1.37, 23.08)	27.40 (7.19, 47.61)	-16.76 (-23.41, -10.10)	-0.47 (-8.70, 7.76)	13.73 (-7.61, 35.08)	0.66 (-7.73, 9.05)
	2018	24.66 (17.82, 31.49)	-11.74 (-21.68, -1.81)	-6.62 (-19.09, 5.86)	0.01 (-12.94, 12.97)	9.53 (-6.28, 25.35)	12.84 (-3.45, 29.12)	10.80 (-1.31, 22.91)	28.20 (7.62, 48.78)	-17.05 (-23.89, -10.22)	-0.01 (-7.84, 7.83)	15.04 (-9.02, 39.10)	1.09 (-7.46, 9.63)
	2019	25.77 (18.88, 32.65)	-11.36 (-21.94, -0.78)	-4.81 (-15.97, 6.34)	5.53 (-14.07, 25.13)	15.72 (0.72, 30.73)	15.46 (-1.71, 32.63)	10.93 (-1.58, 23.43)	30.26 (8.74, 51.78)	-16.19 (-22.93, -9.45)	1.64 (-6.41, 9.69)	15.81 (-8.58, 40.21)	1.51 (-7.31, 10.33)
	2020	26.57 (19.72, 33.42)	-10.34 (-19.66, -1.01)	-0.47 (-30.47, 29.52)	15.98 (3.07, 28.90)	19.14 (4.46, 33.83)	17.68 (0.12, 35.23)	10.75 (-2.12, 23.61)	29.46 (7.96, 50.97)	-14.75 (-21.33, -8.18)	2.64 (-6.57, 11.84)	18.14 (-8.77, 45.04)	3.45 (-5.18, 12.08)
	2021	26.22 (19.29, 33.15)	-12.05 (-21.23, -2.88)	-1.27 (-15.66, 13.13)	10.02 (-2.67, 22.70)	17.59 (1.09, 34.10)	16.36 (-1.24, 33.97)	13.44 (0.42, 26.45)	26.47 (6.77, 46.17)	-14.59 (-21.17, -8.01)	1.49 (-6.28, 9.26)	17.31 (-7.41, 42.03)	1.28 (-7.75, 10.32)
Male													
	1990	11.80 (6.18, 17.42)	-6.14 (-13.70, 1.42)	-6.86 (-19.63, 5.91)	-8.07 (-17.36, 1.23)	2.53 (-7.92, 12.97)	9.71 (-1.52, 20.93)	14.30 (5.17, 23.42)	0.10 (-11.12, 11.33)	-9.70 (-15.35, -4.06)	4.31 (-6.11, 14.74)	-9.60 (-31.31, 12.11)	-3.80 (-18.24, 10.63)
	1991	12.46 (6.84, 18.07)	-5.99 (-14.63, 2.66)	-3.65 (-14.91, 7.61)	-5.34 (-14.71, 4.03)	8.69 (-12.52, 29.89)	8.82 (1.37, 16.26)	12.73 (3.78, 21.68)	-0.13 (-11.57, 11.32)	-9.81 (-15.51, -4.10)	4.40 (-5.80, 14.61)	-7.64 (-29.45, 14.17)	-3.27 (-17.33, 10.80)
	1992	12.90 (7.20, 18.61)	-5.64 (-15.37, 4.09)	-0.17 (-15.88, 15.54)	0.24 (-8.29, 8.77)	10.15 (-2.67, 22.97)	9.65 (2.26, 17.04)	12.34 (3.36, 21.31)	0.24 (-11.11, 11.59)	-10.06 (-16.20, -3.93)	3.42 (-6.41, 13.25)	-7.72 (-29.40, 13.96)	-0.72 (-12.74, 11.29)
	1993	13.24 (7.59, 18.89)	3.10 (-14.49, 20.69)	1.50 (-13.79, 16.78)	1.76 (-7.26, 10.78)	9.97 (-0.63, 20.58)	9.25 (1.70, 16.80)	12.06 (3.27, 20.85)	0.29 (-10.76, 11.33)	-10.13 (-16.14, -4.12)	2.50 (-8.31, 13.31)	-7.50 (-29.26, 14.25)	-1.38 (-12.99, 10.22)

Sex	Year	Location											
		Global	High SDI	High-middle SDI	Middle SDI	Low-middle SDI	Low SDI	AFR	EMR	EUR	AMR	SEAR	WPR
	1994	12.96 (7.36, 18.56)	11.53 (-11.77, 34.82)	1.56 (-13.83, 16.94)	-3.01 (-13.29, 7.26)	4.15 (-5.54, 13.83)	8.29 (0.62, 15.96)	11.85 (3.35, 20.35)	0.27 (-10.42, 10.97)	-10.57 (-16.60, -4.55)	3.80 (-5.75, 13.34)	-7.20 (-28.81, 14.41)	-0.60 (-11.27, 10.06)
	1995	12.78 (7.29, 18.26)	16.13 (-22.61, 54.87)	6.50 (-7.35, 20.35)	-13.06 (-22.54, -3.57)	4.94 (-3.60, 13.47)	8.64 (0.56, 16.72)	12.37 (3.65, 21.09)	0.44 (-10.06, 10.94)	-11.62 (-17.68, -5.56)	5.17 (-4.96, 15.30)	-6.22 (-28.74, 16.29)	0.13 (-9.83, 10.08)
	1996	13.01 (7.59, 18.43)	18.48 (-4.89, 41.86)	7.26 (-6.82, 21.34)	-5.74 (-12.88, 1.41)	2.19 (-5.66, 10.03)	7.27 (-1.13, 15.67)	11.52 (2.40, 20.65)	-0.58 (-10.97, 9.80)	-11.57 (-17.66, -5.48)	6.60 (-3.20, 16.40)	-5.75 (-28.22, 16.71)	3.64 (-6.55, 13.83)
	1997	13.46 (8.02, 18.91)	17.85 (-6.12, 41.82)	4.98 (-8.18, 18.13)	-5.02 (-14.29, 4.25)	3.42 (-4.52, 11.36)	8.11 (-0.32, 16.54)	10.69 (1.73, 19.65)	-1.26 (-11.75, 9.22)	-12.61 (-18.68, -6.55)	7.49 (-2.40, 17.39)	-4.91 (-27.21, 17.39)	4.14 (-5.76, 14.04)
	1998	13.85 (8.37, 19.33)	4.93 (-15.31, 25.17)	4.08 (-10.81, 18.97)	2.11 (-8.16, 12.38)	9.00 (1.37, 16.64)	10.97 (2.26, 19.68)	10.06 (0.94, 19.17)	-1.24 (-12.75, 10.26)	-13.35 (-19.47, -7.23)	8.16 (-2.07, 18.39)	-4.60 (-27.13, 17.94)	5.40 (-4.27, 15.07)
	1999	13.50 (7.95, 19.05)	-6.83 (-31.54, 17.87)	-9.77 (-22.35, 2.81)	0.88 (-10.07, 11.84)	9.23 (1.63, 16.84)	12.62 (3.49, 21.76)	9.69 (0.66, 18.73)	0.37 (-10.82, 11.56)	-13.98 (-20.11, -7.84)	9.97 (-0.35, 20.30)	-3.66 (-26.04, 18.72)	5.25 (-4.08, 14.59)
	2000	14.10 (8.49, 19.71)	-1.66 (-26.20, 22.89)	-8.81 (-22.11, 4.49)	6.53 (-5.27, 18.33)	9.79 (2.38, 17.21)	13.10 (3.51, 22.69)	9.75 (0.80, 18.70)	0.66 (-10.63, 11.95)	-15.11 (-21.04, -9.19)	11.42 (1.42, 21.43)	-3.50 (-26.22, 19.23)	4.88 (-3.84, 13.59)
	2001	14.26 (8.75, 19.77)	-20.79 (-55.14, 13.56)	-12.65 (-25.47, 0.16)	6.40 (-10.14, 22.95)	10.99 (3.66, 18.33)	12.32 (2.67, 21.98)	8.45 (-0.67, 17.58)	0.48 (-10.78, 11.74)	-15.36 (-21.57, -9.14)	10.73 (0.70, 20.76)	-2.19 (-24.72, 20.33)	5.11 (-3.15, 13.37)
	2002	15.05 (9.58, 20.52)	-16.82 (-39.67, 6.04)	-19.81 (-33.88, -5.74)	2.73 (-10.69, 16.15)	11.61 (4.45, 18.78)	14.85 (4.66, 25.04)	8.48 (-1.06, 18.02)	1.19 (-9.90, 12.27)	-15.45 (-21.72, -9.18)	9.46 (-0.74, 19.65)	-1.33 (-26.11, 23.46)	5.02 (-3.30, 13.34)
	2003	15.20 (9.78, 20.63)	-17.33 (-39.63, 4.96)	-19.47 (-33.40, -5.53)	1.45 (-11.11, 14.02)	11.09 (3.41, 18.77)	14.73 (4.38, 25.08)	8.20 (-0.56, 16.95)	1.98 (-9.58, 13.53)	-15.41 (-21.67, -9.16)	6.76 (-3.26, 16.79)	0.22 (-23.92, 24.37)	5.22 (-2.97, 13.41)
	2004	15.76 (10.37, 21.14)	-12.83 (-32.62, 6.96)	-15.36 (-28.33, -2.40)	6.23 (-4.83, 17.29)	11.11 (3.63, 18.59)	18.84 (7.42, 30.26)	8.90 (-0.06, 17.86)	3.09 (-8.20, 14.39)	-15.08 (-21.52, -8.64)	7.07 (-3.38, 17.53)	0.12 (-22.65, 22.89)	5.49 (-2.55, 13.53)
	2005	15.85 (10.41, 21.29)	-7.12 (-18.53, 4.29)	-15.35 (-28.37, -2.34)	2.44 (-5.97, 10.84)	9.00 (0.38, 17.62)	22.71 (10.66, 34.77)	10.14 (1.03, 19.26)	3.46 (-9.73, 16.66)	-15.29 (-21.29, -9.28)	6.60 (-4.25, 17.44)	-0.36 (-24.55, 23.84)	6.37 (-1.58, 14.32)
	2006	16.62 (11.18, 22.07)	-5.79 (-15.10, 3.52)	-11.98 (-25.49, 1.53)	-1.45 (-12.57, 9.67)	9.85 (0.62, 19.08)	24.03 (11.48, 36.58)	11.57 (2.27, 20.87)	4.05 (-9.08, 17.18)	-15.68 (-21.71, -9.64)	6.82 (-3.92, 17.55)	0.22 (-27.26, 27.69)	7.34 (-0.07, 14.75)
	2007	16.33 (10.84, 21.83)	-5.77 (-17.29, 5.75)	-15.88 (-30.94, -0.83)	-7.85 (-20.15, 4.44)	10.53 (-1.30, 22.37)	27.41 (14.50, 40.33)	11.83 (2.57, 21.09)	4.96 (-7.73, 17.65)	-15.68 (-21.95, -9.41)	5.70 (-4.58, 15.99)	1.89 (-26.39, 30.16)	7.44 (0.25, 14.62)
	2008	17.29 (11.79, 22.78)	-3.00 (-20.50, 14.51)	-4.24 (-13.98, 5.51)	-7.70 (-24.57, 9.18)	17.69 (6.73, 28.66)	32.11 (18.50, 45.73)	12.77 (3.06, 22.47)	5.89 (-6.30, 18.09)	-15.92 (-22.25, -9.59)	5.48 (-4.99, 15.96)	3.13 (-21.41, 27.68)	6.39 (-1.61, 14.40)
	2009	17.26 (11.65, 22.87)	-1.86 (-15.31, 11.59)	-4.83 (-13.94, 4.29)	1.58 (-6.89, 10.04)	19.28 (7.94, 30.62)	35.15 (16.88, 53.42)	13.42 (3.85, 22.99)	6.53 (-6.06, 19.13)	-16.51 (-22.94, -10.09)	4.08 (-5.63, 13.79)	4.79 (-22.45, 32.04)	6.95 (-0.90, 14.80)
	2010	17.34 (11.58, 23.10)	-0.53 (-12.15, 11.09)	-5.23 (-13.35, 2.90)	0.31 (-7.25, 7.86)	34.67 (11.89, 57.44)	16.41 (4.06, 28.76)	13.84 (3.57, 24.11)	10.04 (-1.92, 22.00)	-16.83 (-23.18, -10.48)	4.05 (-6.01, 14.10)	5.55 (-18.91, 30.00)	6.83 (-1.35, 15.01)
	2011	17.66 (11.99, 23.33)	-2.87 (-13.26, 7.52)	-3.35 (-11.42, 4.72)	-1.59 (-7.46, 4.29)	3.30 (-19.96, 26.56)	15.93 (4.77, 27.09)	14.61 (4.96, 24.26)	11.27 (-0.25, 22.78)	-17.58 (-23.81, -11.34)	3.11 (-6.92, 13.14)	5.92 (-15.27, 27.11)	7.59 (0.10, 15.08)

Sex	Year	Location											
		Global	High SDI	High-middle SDI	Middle SDI	Low-middle SDI	Low SDI	AFR	EMR	EUR	AMR	SEAR	WPR
	2012	17.54	-4.58	-2.84	-0.40	1.40	15.01	14.73	11.37	-19.47	2.83	5.20	7.68
		(11.82, 23.26)	(-13.58, 4.42)	(-11.80, 6.13)	(-6.09, 5.28)	(-18.59, 21.40)	(3.28, 26.73)	(4.57, 24.90)	(-0.53, 23.26)	(-25.78, -13.17)	(-7.28, 12.93)	(-15.09, 25.49)	(0.24, 15.11)
	2013	17.78	-3.61	-0.36	0.63	3.23	10.13	15.41	11.79	-19.91	3.06	5.58	7.64
		(11.96, 23.59)	(-13.41, 6.19)	(-7.91, 7.20)	(-5.06, 6.31)	(-12.86, 19.31)	(-3.68, 23.94)	(5.28, 25.55)	(0.06, 23.52)	(-26.56, -13.27)	(-6.67, 12.79)	(-15.56, 26.72)	(-0.36, 15.64)
	2014	17.95	-3.70	-1.63	0.67	0.16	10.33	14.99	12.62	-20.30	2.54	6.98	7.78
		(12.00, 23.90)	(-13.21, 5.80)	(-9.61, 6.35)	(-5.70, 7.03)	(-14.48, 14.81)	(-4.21, 24.87)	(4.56, 25.43)	(1.23, 24.02)	(-27.30, -13.30)	(-6.80, 11.88)	(-16.10, 30.07)	(-0.73, 16.30)
	2015	18.47	-4.41	-0.15	0.35	-1.25	11.65	15.53	14.07	-20.70	3.81	7.86	7.98
		(12.52, 24.41)	(-13.90, 5.09)	(-8.17, 7.88)	(-7.05, 7.75)	(-13.34, 10.84)	(-3.51, 26.80)	(5.04, 26.01)	(2.54, 25.60)	(-27.93, -13.46)	(-6.01, 13.63)	(-15.66, 31.38)	(-0.37, 16.34)
	2016	18.75	-6.85	-4.07	-0.62	1.90	5.79	15.14	14.18	-20.24	4.06	7.87	8.36
		(12.82, 24.69)	(-16.29, 2.58)	(-12.87, 4.73)	(-7.70, 6.46)	(-10.55, 14.34)	(-8.06, 19.63)	(4.40, 25.88)	(2.14, 26.22)	(-27.53, -12.95)	(-5.98, 14.10)	(-15.66, 31.41)	(-0.19, 16.91)
	2017	19.23	-6.55	-5.36	-0.77	4.18	5.93	15.26	14.91	-19.38	3.70	9.00	8.74
		(13.26, 25.21)	(-15.48, 2.38)	(-15.78, 5.06)	(-7.42, 5.88)	(-7.96, 16.33)	(-7.28, 19.15)	(4.33, 26.20)	(2.53, 27.29)	(-26.49, -12.26)	(-6.38, 13.79)	(-12.40, 30.41)	(0.05, 17.43)
	2018	19.87	-7.82	-6.14	3.38	1.61	8.46	14.16	15.87	-18.73	3.90	10.08	9.15
		(13.92, 25.81)	(-16.32, 0.69)	(-16.12, 3.84)	(-6.21, 12.96)	(-11.42, 14.65)	(-6.64, 23.56)	(3.04, 25.27)	(3.43, 28.30)	(-25.71, -11.75)	(-6.26, 14.05)	(-10.99, 31.15)	(0.10, 18.20)
	2019	20.64	-7.42	-6.00	5.38	7.63	10.55	14.33	17.18	-17.59	5.76	10.47	9.38
		(14.61, 26.67)	(-15.79, 0.95)	(-16.06, 4.06)	(-9.17, 19.93)	(-4.38, 19.63)	(-4.36, 25.45)	(2.94, 25.72)	(4.69, 29.66)	(-24.48, -10.71)	(-4.69, 16.21)	(-10.81, 31.75)	(-0.77, 19.54)
	2020	20.82	-6.65	-0.57	9.85	13.01	12.51	13.67	15.87	-16.06	6.47	15.05	10.47
		(14.92, 26.73)	(-15.03, 1.72)	(-26.28, 25.15)	(-0.37, 20.07)	(1.49, 24.52)	(-4.32, 29.34)	(2.45, 24.90)	(3.20, 28.53)	(-22.85, -9.28)	(-3.62, 16.57)	(-6.71, 36.81)	(0.48, 20.46)
	2021	19.74	-8.20	-5.56	6.66	12.56	10.56	16.81	14.24	-15.50	6.25	15.15	8.19
		(13.70, 25.78)	(-16.49, 0.09)	(-17.51, 6.40)	(-2.54, 15.86)	(0.46, 24.66)	(-5.67, 26.78)	(4.30, 29.31)	(2.04, 26.44)	(-22.42, -8.59)	(-3.85, 16.36)	(-7.32, 37.62)	(-2.43, 18.81)

Notes: ADRDs, Alzheimer's disease and related dementia; SDI, Socio-demographic Index; WHO, World Health Organization; AFR, African Region; EMR, Eastern Mediterranean Region; AMR, Region of the Americas; EUR, European Region; SEAR, South-East Asia Region; WPR, Western Pacific Region.

Table S7. The year trends of concentration index for care quality of ADRDs by sex, SDI quantiles and WHO regions, from 1990 to 2021

Sex	Year	Location											
		Global	High SDI	High-middle SDI	Middle SDI	Low-middle SDI	Low SDI	AFR	EMR	EUR	AMR	SEAR	WPR
Both													
	1990	-0.0027 (-0.0319, 0.0265)	0.0079 (0.0009, 0.0149)	-0.0200 (-0.0888, 0.0489)	-0.1083 (-0.1582, -0.0584)	0.0271 (-0.0050, 0.0592)	-0.1699 (-0.2232, -0.1166)	0.0829 (0.0406, 0.1252)	0.0354 (-0.0113, 0.0820)	-0.0477 (-0.0810, -0.0144)	-0.0140 (-0.0680, 0.0400)	0.0126 (-0.0586, 0.0839)	0.1109 (-0.0151, 0.2369)
	1991	0.0098 (-0.0169, 0.0366)	0.0059 (-0.0009, 0.0128)	-0.0188 (-0.0862, 0.0485)	-0.1046 (-0.1565, -0.0527)	0.2518 (0.2023, 0.3013)	0.0679 (0.0359, 0.0999)	0.0766 (0.0341, 0.1192)	0.0370 (-0.0106, 0.0845)	-0.0468 (-0.0810, -0.0126)	-0.0131 (-0.0681, 0.0419)	0.0070 (-0.0635, 0.0776)	0.0734 (-0.0305, 0.1773)
	1992	0.0077 (-0.0166, 0.0319)	0.0059 (-0.0010, 0.0129)	0.0533 (-0.0224, 0.1290)	0.0145 (-0.0159, 0.0449)	0.1996 (0.1551, 0.2442)	0.0669 (0.0348, 0.0990)	0.0727 (0.0307, 0.1147)	0.0395 (-0.0088, 0.0879)	-0.0470 (-0.0818, -0.0121)	-0.0097 (-0.0656, 0.0462)	0.0051 (-0.0660, 0.0762)	0.0391 (-0.0445, 0.1227)
	1993	0.0054 (-0.0173, 0.0281)	0.0444 (-0.0229, 0.1117)	0.0515 (-0.0266, 0.1295)	0.0085 (-0.0247, 0.0417)	0.1704 (0.1302, 0.2105)	0.0672 (0.0343, 0.1000)	0.0722 (0.0303, 0.1141)	0.0419 (-0.0079, 0.0917)	-0.0482 (-0.0838, -0.0127)	-0.0095 (-0.0669, 0.0479)	0.0023 (-0.0686, 0.0731)	0.0184 (-0.0533, 0.0900)
	1994	0.0041 (-0.0175, 0.0258)	0.0730 (0.0135, 0.1325)	0.0454 (-0.0319, 0.1226)	0.0076 (-0.0292, 0.0445)	0.1344 (0.0935, 0.1753)	0.0646 (0.0312, 0.0979)	0.0711 (0.0295, 0.1127)	0.0441 (-0.0071, 0.0952)	-0.0483 (-0.0845, -0.0120)	-0.0099 (-0.0673, 0.0475)	0.0014 (-0.0693, 0.0722)	0.0095 (-0.0539, 0.0729)
	1995	0.0050 (-0.0157, 0.0257)	0.1223 (0.0671, 0.1774)	0.1252 (0.0610, 0.1895)	-0.0061 (-0.0439, 0.0316)	0.0911 (0.0492, 0.1330)	0.0757 (0.0422, 0.1091)	0.0763 (0.0349, 0.1177)	0.0469 (-0.0052, 0.0990)	-0.0528 (-0.0893, -0.0163)	-0.0104 (-0.0673, 0.0465)	0.0075 (-0.0656, 0.0805)	0.0024 (-0.0547, 0.0596)
	1996	-0.0013 (-0.0216, 0.0189)	0.1119 (0.0557, 0.1682)	0.1177 (0.0552, 0.1802)	0.0050 (-0.0269, 0.0368)	0.0063 (-0.0420, 0.0546)	0.0752 (0.0414, 0.1090)	0.0756 (0.0352, 0.1160)	0.0484 (-0.0042, 0.1009)	-0.0543 (-0.0913, -0.0173)	-0.0104 (-0.0665, 0.0458)	0.0069 (-0.0659, 0.0797)	0.0054 (-0.0477, 0.0585)
	1997	0.0007 (-0.0191, 0.0204)	0.1035 (0.0463, 0.1607)	0.1001 (0.0391, 0.1610)	0.0170 (-0.0165, 0.0504)	0.0052 (-0.0401, 0.0505)	0.0796 (0.0453, 0.1139)	0.0730 (0.0327, 0.1134)	0.0503 (-0.0030, 0.1036)	-0.0538 (-0.0913, -0.0162)	-0.0128 (-0.0675, 0.0420)	0.0062 (-0.0641, 0.0766)	0.0074 (-0.0425, 0.0572)
	1998	0.0036 (-0.0158, 0.0230)	0.0990 (0.0461, 0.1520)	0.1007 (0.0392, 0.1623)	0.0252 (-0.0080, 0.0583)	0.0102 (-0.0332, 0.0537)	0.0967 (0.0634, 0.1300)	0.0719 (0.0318, 0.1120)	0.0529 (-0.0020, 0.1078)	-0.0553 (-0.0935, -0.0172)	-0.0160 (-0.0697, 0.0377)	0.0079 (-0.0631, 0.0789)	0.0088 (-0.0388, 0.0564)
	1999	0.0015 (-0.0178, 0.0207)	-0.0297 (-0.1682, 0.1089)	0.0506 (0.0028, 0.0985)	0.0263 (-0.0079, 0.0604)	-0.0055 (-0.0477, 0.0367)	0.1037 (0.0694, 0.1379)	0.0705 (0.0297, 0.1114)	0.0555 (-0.0003, 0.1113)	-0.0578 (-0.0959, -0.0197)	-0.0208 (-0.0739, 0.0324)	0.0092 (-0.0615, 0.0798)	0.0105 (-0.0355, 0.0565)
	2000	0.0024 (-0.0168, 0.0217)	-0.0342 (-0.1650, 0.0967)	0.0420 (-0.0048, 0.0888)	0.0263 (-0.0097, 0.0623)	-0.0258 (-0.0665, 0.0149)	0.1102 (0.0746, 0.1458)	0.0697 (0.0274, 0.1120)	0.0608 (0.0029, 0.1187)	-0.0592 (-0.0973, -0.0212)	-0.0230 (-0.0756, 0.0295)	0.0110 (-0.0578, 0.0798)	-0.0374 (-0.0800, 0.0052)
	2001	0.0075 (-0.0114, 0.0264)	-0.0381 (-0.1634, 0.0872)	0.0059 (-0.0418, 0.0537)	0.0210 (-0.0184, 0.0605)	-0.0228 (-0.0635, 0.0180)	0.1052 (0.0696, 0.1409)	0.0653 (0.0234, 0.1072)	0.0652 (0.0050, 0.1254)	-0.0597 (-0.0975, -0.0219)	-0.0239 (-0.0765, 0.0286)	0.0183 (-0.0478, 0.0844)	-0.0295 (-0.0716, 0.0126)
	2002	0.0117 (-0.0071, 0.0306)	-0.0456 (-0.1582, 0.0670)	-0.0712 (-0.1150, -0.0274)	-0.0525 (-0.0959, -0.0091)	-0.0249 (-0.0642, 0.0143)	0.1065 (0.0696, 0.1434)	0.0641 (0.0197, 0.1085)	0.0697 (0.0087, 0.1307)	-0.0611 (-0.0987, -0.0235)	-0.0248 (-0.0777, 0.0281)	0.0250 (-0.0395, 0.0895)	-0.0223 (-0.0644, 0.0199)
	2003	0.0166 (-0.0022, 0.0354)	-0.0514 (-0.1601, 0.0573)	-0.0700 (-0.1141, -0.0259)	-0.0508 (-0.0940, -0.0076)	-0.0312 (-0.0699, 0.0075)	0.1024 (0.0651, 0.1396)	0.0615 (0.0165, 0.1065)	0.0744 (0.0122, 0.1366)	-0.0622 (-0.1000, -0.0244)	-0.0291 (-0.0821, 0.0238)	0.0341 (-0.0272, 0.0955)	-0.0149 (-0.0581, 0.0283)
	2004	0.0232 (0.0045, 0.0419)	-0.0078 (-0.1114, 0.0959)	-0.0394 (-0.0867, 0.0078)	0.0278 (0.0045, 0.0511)	-0.0266 (-0.0652, 0.0119)	0.1010 (0.0625, 0.1394)	0.0624 (0.0147, 0.1101)	0.0799 (0.0163, 0.1435)	-0.0602 (-0.0970, -0.0234)	-0.0281 (-0.0808, 0.0246)	0.0390 (-0.0206, 0.0985)	-0.0100 (-0.0537, 0.0337)
	2005	0.0214 (0.0025, 0.0403)	-0.0066 (-0.0995, 0.0863)	-0.0393 (-0.0882, 0.0096)	0.0207 (-0.0019, 0.0434)	-0.0597 (-0.0965, -0.0229)	0.1006 (0.0608, 0.1404)	0.0619 (0.0112, 0.1126)	0.0829 (0.0186, 0.1472)	-0.0606 (-0.0976, -0.0237)	-0.0313 (-0.0837, 0.0211)	0.0422 (-0.0163, 0.1008)	-0.0073 (-0.0501, 0.0356)

Sex	Year	Location											
		Global	High SDI	High-middle SDI	Middle SDI	Low-middle SDI	Low SDI	AFR	EMR	EUR	AMR	SEAR	WPR
	2006	0.0261 (0.0073, 0.0448)	-0.0002 (-0.0855, 0.0850)	-0.0388 (-0.0882, 0.0106)	0.0133 (-0.0084, 0.0351)	-0.0647 (-0.0999, -0.0295)	0.1011 (0.0600, 0.1422)	0.0639 (0.0098, 0.1179)	0.0866 (0.0215, 0.1517)	-0.0587 (-0.0960, -0.0214)	-0.0316 (-0.0843, 0.0211)	0.0482 (-0.0119, 0.1083)	-0.0086 (-0.0474, 0.0303)
	2007	0.0298 (0.0111, 0.0486)	0.0042 (-0.0729, 0.0812)	-0.0419 (-0.0923, 0.0085)	0.0193 (-0.0057, 0.0444)	-0.0697 (-0.1046, -0.0347)	0.0973 (0.0562, 0.1385)	0.0646 (0.0102, 0.1190)	0.0887 (0.0228, 0.1546)	-0.0558 (-0.0940, -0.0176)	-0.0315 (-0.0842, 0.0212)	0.0556 (-0.0080, 0.1191)	-0.0103 (-0.0471, 0.0264)
	2008	0.0325 (0.0135, 0.0516)	0.0192 (-0.0347, 0.0731)	-0.0680 (-0.1308, -0.0051)	0.1191 (0.0977, 0.1405)	0.0480 (0.0158, 0.0803)	0.1053 (0.0631, 0.1475)	0.0683 (0.0111, 0.1255)	0.0923 (0.0253, 0.1592)	-0.0523 (-0.0915, -0.0131)	-0.0321 (-0.0847, 0.0205)	0.0593 (-0.0042, 0.1228)	-0.0102 (-0.0458, 0.0254)
	2009	0.0319 (0.0126, 0.0512)	0.0279 (-0.0245, 0.0803)	-0.0807 (-0.1366, -0.0248)	0.0485 (0.0074, 0.0897)	0.0445 (0.0118, 0.0772)	0.1133 (0.0692, 0.1573)	0.0695 (0.0095, 0.1296)	0.0953 (0.0272, 0.1633)	-0.0490 (-0.0890, -0.0091)	-0.0297 (-0.0821, 0.0226)	0.0597 (0.0004, 0.1189)	-0.0078 (-0.0435, 0.0279)
	2010	0.0347 (0.0151, 0.0543)	0.0456 (-0.0053, 0.0966)	-0.0775 (-0.1327, -0.0223)	0.0355 (-0.0084, 0.0794)	0.0813 (0.0503, 0.1124)	0.0599 (-0.0062, 0.1261)	0.0738 (0.0095, 0.1380)	0.1188 (0.0559, 0.1817)	-0.0474 (-0.0878, -0.0069)	-0.0301 (-0.0827, 0.0224)	0.0630 (0.0037, 0.1222)	-0.0085 (-0.0439, 0.0270)
	2011	0.0357 (0.0160, 0.0554)	0.0384 (-0.0113, 0.0882)	-0.0884 (-0.1435, -0.0333)	-0.0434 (-0.0847, -0.0020)	0.0608 (0.0227, 0.0989)	0.0073 (-0.0605, 0.0752)	0.0750 (0.0046, 0.1454)	0.1211 (0.0582, 0.1840)	-0.0496 (-0.0904, -0.0088)	-0.0545 (-0.1058, -0.0031)	0.0608 (0.0015, 0.1202)	-0.0103 (-0.0444, 0.0237)
	2012	0.0391 (0.0195, 0.0588)	0.0361 (-0.0094, 0.0817)	-0.0919 (-0.1479, -0.0360)	-0.0458 (-0.0804, -0.0111)	0.0676 (0.0271, 0.1080)	0.0056 (-0.0661, 0.0773)	0.0774 (0.0032, 0.1516)	0.1212 (0.0589, 0.1836)	-0.0540 (-0.0961, -0.0119)	-0.0574 (-0.1088, -0.0060)	0.0577 (-0.0088, 0.1242)	-0.0133 (-0.0465, 0.0200)
	2013	0.0524 (0.0320, 0.0728)	0.0536 (0.0103, 0.0970)	-0.0704 (-0.1290, -0.0118)	-0.0313 (-0.0608, -0.0019)	0.0950 (0.0498, 0.1401)	-0.0247 (-0.0996, 0.0503)	0.0839 (0.0054, 0.1625)	0.1216 (0.0586, 0.1845)	-0.0566 (-0.0994, -0.0137)	-0.0599 (-0.1113, -0.0085)	0.0749 (-0.0200, 0.1698)	-0.0141 (-0.0484, 0.0202)
	2014	0.0606 (0.0395, 0.0817)	0.0557 (0.0129, 0.0986)	-0.0731 (-0.1325, -0.0137)	-0.0240 (-0.0548, 0.0068)	0.1159 (0.0588, 0.1730)	-0.0247 (-0.1002, 0.0508)	0.0883 (0.0087, 0.1678)	0.1247 (0.0601, 0.1892)	-0.0586 (-0.1024, -0.0148)	-0.0624 (-0.1136, -0.0112)	0.0842 (-0.0281, 0.1966)	-0.0115 (-0.0478, 0.0249)
	2015	0.0643 (0.0427, 0.0860)	0.0600 (0.0173, 0.1026)	-0.0640 (-0.1249, -0.0030)	-0.0214 (-0.0498, 0.0069)	0.0716 (-0.0004, 0.1437)	-0.0135 (-0.0920, 0.0651)	0.0945 (0.0126, 0.1764)	0.1267 (0.0621, 0.1912)	-0.0588 (-0.1042, -0.0134)	-0.0377 (-0.0923, 0.0170)	0.0884 (-0.0304, 0.2072)	-0.0100 (-0.0476, 0.0277)
	2016	0.0683 (0.0469, 0.0897)	0.0534 (0.0123, 0.0946)	-0.0602 (-0.1172, -0.0032)	-0.0177 (-0.0447, 0.0093)	0.0975 (0.0263, 0.1687)	-0.0004 (-0.0863, 0.0855)	0.0992 (0.0153, 0.1831)	0.1275 (0.0611, 0.1938)	-0.0585 (-0.1007, -0.0163)	-0.0358 (-0.0905, 0.0189)	0.0910 (-0.0283, 0.2103)	-0.0085 (-0.0467, 0.0297)
	2017	0.0770 (0.0555, 0.0986)	0.0484 (0.0083, 0.0885)	0.0228 (-0.0297, 0.0753)	-0.0213 (-0.0461, 0.0035)	0.1017 (0.0189, 0.1845)	0.1371 (0.0718, 0.2023)	0.1064 (0.0194, 0.1933)	0.1354 (0.0648, 0.2060)	-0.0339 (-0.0734, 0.0055)	-0.0359 (-0.0903, 0.0186)	0.1032 (-0.0239, 0.2302)	-0.0077 (-0.0468, 0.0314)
	2018	0.0829 (0.0615, 0.1043)	0.0388 (-0.0009, 0.0785)	0.0085 (-0.0312, 0.0482)	-0.0196 (-0.0474, 0.0081)	0.1030 (0.0162, 0.1898)	0.1418 (0.0740, 0.2096)	0.1110 (0.0218, 0.2001)	0.1372 (0.0663, 0.2082)	-0.0368 (-0.0721, -0.0015)	-0.0336 (-0.0875, 0.0204)	0.1065 (-0.0251, 0.2381)	-0.0060 (-0.0452, 0.0333)
	2019	0.0897 (0.0683, 0.1111)	0.0388 (0.0004, 0.0772)	0.0097 (-0.0262, 0.0457)	-0.0167 (-0.0453, 0.0119)	0.0666 (-0.0151, 0.1483)	0.1214 (0.0289, 0.2138)	0.1173 (0.0248, 0.2099)	0.1426 (0.0698, 0.2154)	-0.0365 (-0.0696, -0.0033)	-0.0195 (-0.0735, 0.0345)	0.1100 (-0.0256, 0.2455)	-0.0026 (-0.0417, 0.0365)
	2020	0.0824 (0.0619, 0.1029)	0.0342 (-0.0011, 0.0696)	0.0211 (-0.0111, 0.0532)	0.0664 (0.0329, 0.0998)	0.0947 (0.0213, 0.1681)	0.1206 (0.0157, 0.2255)	0.1248 (0.0323, 0.2174)	0.1433 (0.0707, 0.2159)	-0.0330 (-0.0681, 0.0021)	-0.0186 (-0.0719, 0.0346)	0.0860 (-0.0163, 0.1882)	0.0046 (-0.0346, 0.0439)
	2021	0.0760 (0.0557, 0.0963)	0.0112 (-0.0252, 0.0476)	-0.0076 (-0.0335, 0.0183)	0.0562 (0.0224, 0.0901)	0.1049 (0.0347, 0.1751)	0.1179 (0.0085, 0.2274)	0.1341 (0.0370, 0.2313)	0.1470 (0.0721, 0.2219)	-0.0357 (-0.0659, -0.0056)	-0.0285 (-0.0795, 0.0225)	0.0739 (-0.0053, 0.1531)	-0.0241 (-0.0713, 0.0230)
Female	1990	0.0037 (-0.0206, 0.0279)	0.0160 (0.0079, 0.0241)	-0.0167 (-0.0793, 0.0459)	-0.0963 (-0.1410, -0.0516)	0.0274 (-0.0018, 0.0567)	-0.1230 (-0.1677, -0.0783)	0.0759 (0.0391, 0.1128)	0.0808 (0.0135, 0.1482)	-0.0434 (-0.0739, -0.0128)	-0.0113 (-0.0627, 0.0400)	0.0133 (-0.0474, 0.0740)	0.0607 (-0.0316, 0.1531)

Sex	Year	Location											
		**Global**	**High SDI**	**High-middle SDI**	**Middle SDI**	**Low-middle SDI**	**Low SDI**	**AFR**	**EMR**	**EUR**	**AMR**	**SEAR**	**WPR**
	1991	0.0123 (-0.0100, 0.0346)	0.0147 (0.0066, 0.0227)	-0.0159 (-0.0770, 0.0451)	-0.0926 (-0.1391, -0.0461)	0.1890 (0.1501, 0.2279)	0.0664 (0.0354, 0.0975)	0.0711 (0.0342, 0.1080)	0.0833 (0.0140, 0.1526)	-0.0429 (-0.0742, -0.0116)	-0.0114 (-0.0635, 0.0407)	0.0072 (-0.0525, 0.0670)	0.0342 (-0.0426, 0.1110)
	1992	0.0107 (-0.0096, 0.0310)	0.0150 (0.0068, 0.0232)	0.0484 (-0.0192, 0.1160)	0.0147 (-0.0114, 0.0409)	0.1468 (0.1117, 0.1819)	0.0646 (0.0333, 0.0958)	0.0679 (0.0317, 0.1040)	0.0869 (0.0156, 0.1582)	-0.0432 (-0.0749, -0.0114)	-0.0086 (-0.0614, 0.0442)	0.0060 (-0.0545, 0.0665)	0.0086 (-0.0530, 0.0701)
	1993	0.0083 (-0.0109, 0.0274)	0.0325 (-0.0122, 0.0771)	0.0466 (-0.0231, 0.1162)	0.0103 (-0.0184, 0.0390)	0.1224 (0.0908, 0.1541)	0.0648 (0.0327, 0.0970)	0.0687 (0.0328, 0.1046)	0.0904 (0.0166, 0.1643)	-0.0444 (-0.0767, -0.0121)	-0.0083 (-0.0625, 0.0458)	0.0029 (-0.0568, 0.0626)	-0.0075 (-0.0598, 0.0448)
	1994	0.0073 (-0.0111, 0.0258)	0.0516 (0.0098, 0.0933)	0.0405 (-0.0288, 0.1097)	0.0100 (-0.0219, 0.0418)	0.0940 (0.0619, 0.1261)	0.0622 (0.0294, 0.0951)	0.0682 (0.0327, 0.1037)	0.0935 (0.0179, 0.1691)	-0.0446 (-0.0775, -0.0117)	-0.0083 (-0.0625, 0.0459)	0.0013 (-0.0581, 0.0607)	-0.0130 (-0.0592, 0.0332)
	1995	0.0099 (-0.0079, 0.0276)	0.1041 (0.0525, 0.1556)	0.1088 (0.0499, 0.1677)	-0.0028 (-0.0357, 0.0300)	0.0605 (0.0281, 0.0929)	0.0691 (0.0356, 0.1026)	0.0748 (0.0394, 0.1103)	0.0961 (0.0190, 0.1732)	-0.0487 (-0.0817, -0.0156)	-0.0080 (-0.0616, 0.0457)	0.0088 (-0.0543, 0.0719)	-0.0182 (-0.0599, 0.0236)
	1996	0.0062 (-0.0113, 0.0237)	0.0937 (0.0426, 0.1449)	0.1030 (0.0460, 0.1601)	0.0090 (-0.0190, 0.0370)	0.0027 (-0.0341, 0.0395)	0.0689 (0.0350, 0.1027)	0.0744 (0.0396, 0.1093)	0.0967 (0.0190, 0.1745)	-0.0500 (-0.0834, -0.0166)	-0.0061 (-0.0588, 0.0466)	0.0069 (-0.0556, 0.0693)	-0.0138 (-0.0529, 0.0252)
	1997	0.0075 (-0.0097, 0.0247)	0.0848 (0.0339, 0.1357)	0.0911 (0.0365, 0.1457)	0.0204 (-0.0086, 0.0494)	0.0017 (-0.0329, 0.0362)	0.0749 (0.0404, 0.1094)	0.0727 (0.0379, 0.1074)	0.0981 (0.0196, 0.1766)	-0.0496 (-0.0835, -0.0157)	-0.0049 (-0.0557, 0.0460)	0.0030 (-0.0561, 0.0622)	-0.0109 (-0.0478, 0.0259)
	1998	0.0112 (-0.0058, 0.0282)	0.0795 (0.0336, 0.1254)	0.0952 (0.0407, 0.1497)	0.0252 (-0.0033, 0.0538)	0.0066 (-0.0267, 0.0398)	0.0895 (0.0546, 0.1243)	0.0723 (0.0378, 0.1069)	0.1006 (0.0202, 0.1811)	-0.0510 (-0.0854, -0.0165)	-0.0040 (-0.0533, 0.0452)	0.0049 (-0.0540, 0.0638)	-0.0081 (-0.0439, 0.0278)
	1999	0.0118 (-0.0052, 0.0287)	-0.0300 (-0.1475, 0.0874)	0.0573 (0.0130, 0.1017)	0.0270 (-0.0025, 0.0565)	0.0042 (-0.0285, 0.0370)	0.0957 (0.0598, 0.1315)	0.0718 (0.0371, 0.1064)	0.1032 (0.0214, 0.1851)	-0.0532 (-0.0876, -0.0189)	-0.0049 (-0.0530, 0.0433)	0.0059 (-0.0538, 0.0656)	-0.0048 (-0.0399, 0.0303)
	2000	0.0134 (-0.0035, 0.0304)	-0.0352 (-0.1448, 0.0744)	0.0507 (0.0073, 0.0941)	0.0272 (-0.0038, 0.0582)	-0.0092 (-0.0413, 0.0228)	0.1017 (0.0643, 0.1390)	0.0707 (0.0350, 0.1064)	0.1081 (0.0236, 0.1925)	-0.0545 (-0.0887, -0.0203)	-0.0051 (-0.0524, 0.0423)	0.0066 (-0.0511, 0.0643)	-0.0328 (-0.0639, -0.0018)
	2001	0.0184 (0.0017, 0.0351)	-0.0391 (-0.1427, 0.0644)	0.0173 (-0.0272, 0.0618)	0.0228 (-0.0112, 0.0568)	-0.0061 (-0.0381, 0.0260)	0.0970 (0.0591, 0.1349)	0.0664 (0.0313, 0.1015)	0.1139 (0.0257, 0.2021)	-0.0551 (-0.0891, -0.0212)	-0.0054 (-0.0528, 0.0419)	0.0133 (-0.0413, 0.0679)	-0.0247 (-0.0556, 0.0063)
	2002	0.0223 (0.0055, 0.0390)	-0.0467 (-0.1385, 0.0451)	-0.0588 (-0.0998, -0.0178)	-0.0431 (-0.0810, -0.0052)	-0.0085 (-0.0396, 0.0226)	0.0976 (0.0582, 0.1369)	0.0647 (0.0285, 0.1009)	0.1203 (0.0297, 0.2109)	-0.0569 (-0.0909, -0.0230)	-0.0057 (-0.0533, 0.0420)	0.0214 (-0.0324, 0.0752)	-0.0171 (-0.0484, 0.0141)
	2003	0.0263 (0.0095, 0.0431)	-0.0525 (-0.1403, 0.0352)	-0.0576 (-0.0991, -0.0161)	-0.0418 (-0.0797, -0.0040)	-0.0139 (-0.0447, 0.0170)	0.0947 (0.0550, 0.1344)	0.0626 (0.0262, 0.0989)	0.1251 (0.0337, 0.2166)	-0.0585 (-0.0928, -0.0243)	-0.0102 (-0.0580, 0.0376)	0.0299 (-0.0203, 0.0801)	-0.0096 (-0.0417, 0.0225)
	2004	0.0319 (0.0151, 0.0486)	-0.0072 (-0.0962, 0.0818)	-0.0285 (-0.0717, 0.0148)	0.0292 (0.0101, 0.0483)	-0.0104 (-0.0414, 0.0205)	0.0930 (0.0520, 0.1340)	0.0628 (0.0246, 0.1010)	0.1316 (0.0383, 0.2249)	-0.0571 (-0.0905, -0.0237)	-0.0096 (-0.0573, 0.0381)	0.0338 (-0.0142, 0.0819)	-0.0044 (-0.0372, 0.0283)
	2005	0.0318 (0.0149, 0.0487)	-0.0061 (-0.0858, 0.0736)	-0.0281 (-0.0728, 0.0166)	0.0238 (0.0044, 0.0431)	-0.0361 (-0.0663, -0.0059)	0.0883 (0.0460, 0.1306)	0.0622 (0.0221, 0.1023)	0.1349 (0.0408, 0.2291)	-0.0576 (-0.0911, -0.0241)	-0.0125 (-0.0598, 0.0348)	0.0382 (-0.0109, 0.0874)	-0.0019 (-0.0339, 0.0300)
	2006	0.0363 (0.0194, 0.0532)	-0.0016 (-0.0745, 0.0713)	-0.0281 (-0.0733, 0.0170)	0.0159 (-0.0029, 0.0347)	-0.0389 (-0.0683, -0.0095)	0.0867 (0.0432, 0.1303)	0.0627 (0.0203, 0.1051)	0.1388 (0.0436, 0.2340)	-0.0560 (-0.0897, -0.0223)	-0.0133 (-0.0610, 0.0344)	0.0450 (-0.0075, 0.0975)	-0.0036 (-0.0328, 0.0255)
	2007	0.0392 (0.0222, 0.0562)	0.0013 (-0.0649, 0.0674)	-0.0304 (-0.0763, 0.0155)	0.0238 (0.0015, 0.0460)	-0.0410 (-0.0706, -0.0113)	0.0838 (0.0401, 0.1276)	0.0628 (0.0205, 0.1051)	0.1414 (0.0455, 0.2372)	-0.0534 (-0.0878, -0.0190)	-0.0132 (-0.0609, 0.0346)	0.0511 (-0.0048, 0.1070)	-0.0066 (-0.0343, 0.0210)
	2008	0.0412 (0.0240, 0.0584)	0.0070 (-0.0381, 0.0521)	-0.0715 (-0.1281, -0.0149)	0.0826 (0.0639, 0.1014)	0.0442 (0.0131, 0.0753)	0.0919 (0.0467, 0.1370)	0.0649 (0.0206, 0.1092)	0.1464 (0.0486, 0.2441)	-0.0505 (-0.0857, -0.0153)	-0.0133 (-0.0610, 0.0343)	0.0546 (-0.0015, 0.1107)	-0.0082 (-0.0345, 0.0181)

Sex	Year	Location											
		Global	High SDI	High-middle SDI	Middle SDI	Low-middle SDI	Low SDI	AFR	EMR	EUR	AMR	SEAR	WPR
	2009	0.0401 (0.0227, 0.0575)	0.0129 (-0.0314, 0.0572)	-0.0812 (-0.1311, -0.0313)	0.0323 (0.0023, 0.0623)	0.0390 (0.0073, 0.0708)	0.1000 (0.0531, 0.1469)	0.0650 (0.0185, 0.1114)	0.1499 (0.0508, 0.2491)	-0.0477 (-0.0834, -0.0121)	-0.0112 (-0.0589, 0.0365)	0.0541 (0.0030, 0.1053)	-0.0074 (-0.0332, 0.0184)
	2010	0.0421 (0.0244, 0.0598)	0.0273 (-0.0165, 0.0710)	-0.0781 (-0.1273, -0.0289)	0.0253 (-0.0067, 0.0572)	0.0767 (0.0467, 0.1067)	0.0262 (-0.0429, 0.0952)	0.0672 (0.0164, 0.1181)	0.1795 (0.0890, 0.2700)	-0.0466 (-0.0825, -0.0106)	-0.0113 (-0.0592, 0.0366)	0.0567 (0.0060, 0.1075)	-0.0093 (-0.0349, 0.0164)
	2011	0.0435 (0.0255, 0.0615)	0.0205 (-0.0222, 0.0632)	-0.0876 (-0.1362, -0.0390)	-0.0355 (-0.0668, -0.0041)	0.0542 (0.0177, 0.0907)	-0.0328 (-0.1028, 0.0371)	0.0674 (0.0108, 0.1241)	0.1803 (0.0904, 0.2702)	-0.0478 (-0.0838, -0.0118)	-0.0355 (-0.0833, 0.0123)	0.0555 (0.0037, 0.1073)	-0.0125 (-0.0384, 0.0133)
	2012	0.0470 (0.0289, 0.0652)	0.0189 (-0.0200, 0.0577)	-0.0872 (-0.1363, -0.0381)	-0.0356 (-0.0626, -0.0085)	0.0588 (0.0197, 0.0978)	-0.0308 (-0.1036, 0.0421)	0.0682 (0.0077, 0.1288)	0.1790 (0.0905, 0.2674)	-0.0504 (-0.0870, -0.0137)	-0.0383 (-0.0866, 0.0100)	0.0549 (-0.0046, 0.1144)	-0.0154 (-0.0432, 0.0124)
	2013	0.0594 (0.0405, 0.0784)	0.0392 (0.0014, 0.0771)	-0.0620 (-0.1128, -0.0113)	-0.0233 (-0.0468, 0.0002)	0.0827 (0.0387, 0.1267)	-0.0693 (-0.1439, 0.0052)	0.0725 (0.0078, 0.1372)	0.1811 (0.0909, 0.2712)	-0.0508 (-0.0875, -0.0142)	-0.0408 (-0.0898, 0.0082)	0.0731 (-0.0131, 0.1593)	-0.0165 (-0.0471, 0.0142)
	2014	0.0657 (0.0461, 0.0852)	0.0397 (0.0030, 0.0765)	-0.0634 (-0.1146, -0.0122)	-0.0046 (-0.0351, 0.0259)	0.1037 (0.0513, 0.1560)	-0.0684 (-0.1439, 0.0072)	0.0752 (0.0086, 0.1418)	0.1820 (0.0920, 0.2721)	-0.0509 (-0.0879, -0.0139)	-0.0431 (-0.0925, 0.0064)	0.0784 (-0.0208, 0.1777)	-0.0134 (-0.0463, 0.0195)
	2015	0.0699 (0.0498, 0.0899)	0.0428 (0.0065, 0.0791)	-0.0530 (-0.1061, 0.0002)	0.0014 (-0.0266, 0.0295)	0.0678 (0.0012, 0.1345)	-0.0605 (-0.1384, 0.0174)	0.0793 (0.0106, 0.1481)	0.1819 (0.0930, 0.2707)	-0.0502 (-0.0883, -0.0121)	-0.0163 (-0.0686, 0.0360)	0.0847 (-0.0213, 0.1907)	-0.0126 (-0.0476, 0.0224)
	2016	0.0748 (0.0548, 0.0948)	0.0372 (0.0023, 0.0721)	-0.0495 (-0.0991, -0.0000)	0.0038 (-0.0232, 0.0307)	0.1205 (0.0486, 0.1924)	0.0007 (-0.0759, 0.0773)	0.0835 (0.0119, 0.1552)	0.1853 (0.0942, 0.2764)	-0.0495 (-0.0846, -0.0143)	-0.0144 (-0.0671, 0.0383)	0.0888 (-0.0206, 0.1982)	-0.0109 (-0.0462, 0.0243)
	2017	0.0849 (0.0645, 0.1052)	0.0326 (-0.0013, 0.0665)	0.0240 (-0.0208, 0.0689)	0.0026 (-0.0225, 0.0277)	0.1249 (0.0408, 0.2089)	0.1224 (0.0624, 0.1824)	0.0887 (0.0142, 0.1632)	0.1972 (0.1001, 0.2943)	-0.0273 (-0.0599, 0.0054)	-0.0139 (-0.0663, 0.0385)	0.1023 (-0.0185, 0.2230)	-0.0099 (-0.0465, 0.0266)
	2018	0.0911 (0.0708, 0.1114)	0.0240 (-0.0097, 0.0578)	0.0138 (-0.0198, 0.0474)	0.0073 (-0.0207, 0.0354)	0.1287 (0.0403, 0.2172)	0.1235 (0.0611, 0.1860)	0.0897 (0.0114, 0.1681)	0.1983 (0.1019, 0.2946)	-0.0306 (-0.0595, -0.0017)	-0.0115 (-0.0634, 0.0405)	0.1071 (-0.0190, 0.2332)	-0.0079 (-0.0446, 0.0288)
	2019	0.0977 (0.0774, 0.1181)	0.0245 (-0.0082, 0.0571)	0.0150 (-0.0151, 0.0452)	0.0117 (-0.0171, 0.0404)	0.0887 (0.0048, 0.1725)	0.1240 (0.0417, 0.2064)	0.0950 (0.0136, 0.1765)	0.2021 (0.1048, 0.2995)	-0.0305 (-0.0575, -0.0035)	0.0039 (-0.0478, 0.0555)	0.1118 (-0.0185, 0.2422)	-0.0049 (-0.0417, 0.0319)
	2020	0.0928 (0.0730, 0.1126)	0.0214 (-0.0084, 0.0513)	0.0055 (-0.0217, 0.0327)	0.0713 (0.0393, 0.1034)	0.0986 (0.0180, 0.1792)	0.1209 (0.0264, 0.2153)	0.1020 (0.0199, 0.1841)	0.2033 (0.1057, 0.3010)	-0.0271 (-0.0565, 0.0022)	0.0033 (-0.0477, 0.0542)	0.0879 (-0.0193, 0.1951)	0.0010 (-0.0355, 0.0376)
	2021	0.0910 (0.0708, 0.1112)	0.0019 (-0.0284, 0.0321)	-0.0191 (-0.0410, 0.0028)	0.0608 (0.0284, 0.0932)	0.0987 (0.0170, 0.1804)	0.1198 (0.0214, 0.2182)	0.1101 (0.0244, 0.1958)	0.2066 (0.1065, 0.3067)	-0.0301 (-0.0550, -0.0052)	-0.0073 (-0.0555, 0.0410)	0.0827 (-0.0151, 0.1805)	-0.0254 (-0.0720, 0.0212)
Male													
	1990	-0.0003 (-0.0271, 0.0266)	-0.0231 (-0.0278, -0.0183)	-0.0163 (-0.0688, 0.0362)	-0.0724 (-0.1077, -0.0371)	0.0224 (-0.0052, 0.0500)	-0.1699 (-0.2187, -0.1212)	0.0528 (0.0116, 0.0940)	-0.0061 (-0.0351, 0.0228)	-0.0302 (-0.0550, -0.0055)	-0.0087 (-0.0513, 0.0340)	0.0135 (-0.0638, 0.0908)	0.1586 (0.0491, 0.2682)
	1991	0.0140 (-0.0106, 0.0386)	-0.0258 (-0.0314, -0.0203)	-0.0136 (-0.0658, 0.0386)	-0.0691 (-0.1064, -0.0318)	0.2499 (0.2030, 0.2969)	0.0540 (0.0251, 0.0828)	0.0474 (0.0060, 0.0888)	-0.0060 (-0.0349, 0.0230)	-0.0281 (-0.0540, -0.0023)	-0.0060 (-0.0499, 0.0378)	0.0104 (-0.0660, 0.0867)	0.1247 (0.0329, 0.2165)
	1992	0.0120 (-0.0106, 0.0345)	-0.0267 (-0.0329, -0.0204)	0.0300 (-0.0243, 0.0843)	0.0117 (-0.0157, 0.0392)	0.2075 (0.1644, 0.2507)	0.0545 (0.0254, 0.0836)	0.0453 (0.0041, 0.0865)	-0.0050 (-0.0337, 0.0237)	-0.0276 (-0.0544, -0.0008)	-0.0026 (-0.0476, 0.0425)	0.0087 (-0.0673, 0.0847)	0.0947 (0.0190, 0.1705)
	1993	0.0110 (-0.0101, 0.0321)	0.0560 (-0.0498, 0.1618)	0.0310 (-0.0250, 0.0870)	0.0037 (-0.0261, 0.0334)	0.1833 (0.1436, 0.2229)	0.0545 (0.0249, 0.0841)	0.0433 (0.0026, 0.0840)	-0.0044 (-0.0334, 0.0246)	-0.0282 (-0.0558, -0.0006)	-0.0023 (-0.0487, 0.0440)	0.0078 (-0.0686, 0.0841)	0.0764 (0.0103, 0.1425)

Sex	Year	Location											
		Global	High SDI	High-middle SDI	Middle SDI	Low-middle SDI	Low SDI	AFR	EMR	EUR	AMR	SEAR	WPR
	1994	0.0096 (-0.0104, 0.0295)	0.1001 (0.0146, 0.1856)	0.0291 (-0.0263, 0.0845)	0.0016 (-0.0314, 0.0346)	0.1485 (0.1081, 0.1889)	0.0514 (0.0215, 0.0813)	0.0428 (0.0027, 0.0829)	-0.0036 (-0.0333, 0.0262)	-0.0278 (-0.0562, 0.0006)	-0.0033 (-0.0497, 0.0431)	0.0088 (-0.0675, 0.0851)	0.0631 (0.0049, 0.1212)
	1995	0.0082 (-0.0110, 0.0274)	0.1030 (0.0420, 0.1640)	0.0869 (0.0385, 0.1353)	-0.0092 (-0.0426, 0.0243)	0.1082 (0.0658, 0.1505)	0.0648 (0.0350, 0.0946)	0.0442 (0.0053, 0.0831)	-0.0012 (-0.0311, 0.0286)	-0.0313 (-0.0601, -0.0026)	-0.0052 (-0.0514, 0.0410)	0.0133 (-0.0631, 0.0897)	0.0562 (0.0035, 0.1088)
	1996	-0.0003 (-0.0189, 0.0183)	0.0987 (0.0349, 0.1626)	0.0825 (0.0350, 0.1299)	-0.0048 (-0.0331, 0.0234)	0.0082 (-0.0418, 0.0582)	0.0630 (0.0332, 0.0929)	0.0448 (0.0073, 0.0823)	0.0002 (-0.0291, 0.0295)	-0.0326 (-0.0619, -0.0032)	-0.0088 (-0.0552, 0.0376)	0.0153 (-0.0611, 0.0917)	0.0547 (0.0064, 0.1030)
	1997	0.0016 (-0.0164, 0.0196)	0.0958 (0.0295, 0.1621)	0.0642 (0.0167, 0.1117)	0.0041 (-0.0260, 0.0341)	0.0081 (-0.0392, 0.0554)	0.0635 (0.0338, 0.0931)	0.0451 (0.0084, 0.0818)	0.0022 (-0.0271, 0.0314)	-0.0321 (-0.0620, -0.0023)	-0.0173 (-0.0641, 0.0296)	0.0188 (-0.0567, 0.0943)	0.0543 (0.0087, 0.0999)
	1998	0.0020 (-0.0157, 0.0197)	0.0952 (0.0313, 0.1590)	0.0589 (0.0091, 0.1087)	0.0155 (-0.0149, 0.0460)	0.0119 (-0.0334, 0.0573)	0.0776 (0.0490, 0.1061)	0.0450 (0.0085, 0.0814)	0.0045 (-0.0253, 0.0344)	-0.0337 (-0.0639, -0.0034)	-0.0277 (-0.0757, 0.0202)	0.0195 (-0.0577, 0.0967)	0.0523 (0.0090, 0.0955)
	1999	-0.0040 (-0.0214, 0.0134)	-0.0145 (-0.1419, 0.1130)	0.0025 (-0.0280, 0.0330)	0.0156 (-0.0157, 0.0469)	-0.0185 (-0.0615, 0.0244)	0.0840 (0.0547, 0.1134)	0.0448 (0.0076, 0.0819)	0.0066 (-0.0235, 0.0367)	-0.0353 (-0.0656, -0.0051)	-0.0389 (-0.0882, 0.0105)	0.0208 (-0.0539, 0.0955)	0.0497 (0.0080, 0.0913)
	2000	-0.0049 (-0.0223, 0.0125)	-0.0159 (-0.1403, 0.1084)	-0.0070 (-0.0378, 0.0238)	0.0155 (-0.0177, 0.0486)	-0.0396 (-0.0801, 0.0010)	0.0893 (0.0593, 0.1193)	0.0464 (0.0084, 0.0844)	0.0113 (-0.0197, 0.0424)	-0.0367 (-0.0671, -0.0062)	-0.0450 (-0.0947, 0.0047)	0.0232 (-0.0503, 0.0968)	-0.0083 (-0.0547, 0.0382)
	2001	-0.0016 (-0.0187, 0.0154)	-0.0187 (-0.1417, 0.1044)	-0.0289 (-0.0599, 0.0021)	0.0110 (-0.0252, 0.0472)	-0.0370 (-0.0774, 0.0034)	0.0847 (0.0551, 0.1143)	0.0450 (0.0079, 0.0821)	0.0128 (-0.0188, 0.0445)	-0.0366 (-0.0670, -0.0062)	-0.0467 (-0.0966, 0.0032)	0.0298 (-0.0427, 0.1023)	-0.0059 (-0.0512, 0.0395)
	2002	0.0020 (-0.0149, 0.0190)	-0.0224 (-0.1365, 0.0917)	-0.0643 (-0.0948, -0.0339)	-0.0504 (-0.0890, -0.0117)	-0.0373 (-0.0761, 0.0015)	0.0862 (0.0557, 0.1167)	0.0462 (0.0071, 0.0853)	0.0142 (-0.0166, 0.0450)	-0.0368 (-0.0667, -0.0069)	-0.0486 (-0.0987, 0.0015)	0.0330 (-0.0372, 0.1033)	-0.0035 (-0.0487, 0.0416)
	2003	0.0064 (-0.0105, 0.0232)	-0.0262 (-0.1390, 0.0866)	-0.0643 (-0.0949, -0.0337)	-0.0480 (-0.0861, -0.0099)	-0.0434 (-0.0815, -0.0053)	0.0815 (0.0509, 0.1121)	0.0450 (0.0061, 0.0840)	0.0173 (-0.0141, 0.0487)	-0.0369 (-0.0665, -0.0072)	-0.0514 (-0.1013, -0.0014)	0.0409 (-0.0280, 0.1098)	-0.0002 (-0.0464, 0.0459)
	2004	0.0120 (-0.0046, 0.0286)	-0.0179 (-0.1129, 0.0771)	-0.0540 (-0.0879, -0.0201)	0.0140 (-0.0116, 0.0396)	-0.0382 (-0.0752, -0.0013)	0.0802 (0.0490, 0.1115)	0.0475 (0.0069, 0.0880)	0.0206 (-0.0109, 0.0522)	-0.0347 (-0.0637, -0.0057)	-0.0500 (-0.0996, -0.0004)	0.0455 (-0.0223, 0.1134)	0.0015 (-0.0439, 0.0469)
	2005	0.0081 (-0.0086, 0.0249)	-0.0166 (-0.1019, 0.0687)	-0.0550 (-0.0904, -0.0196)	0.0077 (-0.0157, 0.0310)	-0.0690 (-0.1037, -0.0343)	0.0856 (0.0535, 0.1177)	0.0485 (0.0057, 0.0914)	0.0227 (-0.0089, 0.0542)	-0.0349 (-0.0639, -0.0058)	-0.0531 (-0.1025, -0.0037)	0.0465 (-0.0181, 0.1110)	0.0037 (-0.0411, 0.0484)
	2006	0.0115 (-0.0052, 0.0281)	-0.0061 (-0.0855, 0.0734)	-0.0544 (-0.0902, -0.0187)	0.0065 (-0.0158, 0.0288)	-0.0760 (-0.1095, -0.0426)	0.0877 (0.0550, 0.1204)	0.0526 (0.0078, 0.0974)	0.0248 (-0.0070, 0.0566)	-0.0334 (-0.0632, -0.0037)	-0.0525 (-0.1021, -0.0028)	0.0500 (-0.0140, 0.1141)	0.0060 (-0.0359, 0.0479)
	2007	0.0159 (-0.0007, 0.0326)	0.0015 (-0.0697, 0.0726)	-0.0570 (-0.0937, -0.0202)	0.0059 (-0.0172, 0.0291)	-0.0848 (-0.1177, -0.0519)	0.0826 (0.0496, 0.1155)	0.0537 (0.0080, 0.0995)	0.0256 (-0.0067, 0.0580)	-0.0304 (-0.0615, 0.0006)	-0.0526 (-0.1021, -0.0032)	0.0575 (-0.0091, 0.1241)	0.0097 (-0.0307, 0.0502)
	2008	0.0194 (0.0025, 0.0362)	0.0541 (-0.0000, 0.1082)	-0.0301 (-0.0764, 0.0162)	0.1307 (0.1117, 0.1497)	0.0387 (0.0120, 0.0654)	0.0867 (0.0531, 0.1204)	0.0575 (0.0097, 0.1052)	0.0270 (-0.0056, 0.0596)	-0.0271 (-0.0596, 0.0054)	-0.0541 (-0.1032, -0.0049)	0.0605 (-0.0055, 0.1266)	0.0147 (-0.0251, 0.0545)
	2009	0.0200 (0.0030, 0.0371)	0.0641 (0.0127, 0.1155)	-0.0428 (-0.0851, -0.0004)	0.0538 (0.0096, 0.0980)	0.0388 (0.0122, 0.0654)	0.0908 (0.0555, 0.1261)	0.0592 (0.0094, 0.1091)	0.0285 (-0.0046, 0.0615)	-0.0242 (-0.0579, 0.0096)	-0.0524 (-0.1010, -0.0038)	0.0621 (-0.0014, 0.1256)	0.0206 (-0.0199, 0.0612)
	2010	0.0232 (0.0061, 0.0404)	0.0804 (0.0322, 0.1285)	-0.0415 (-0.0837, 0.0007)	0.0349 (-0.0119, 0.0818)	0.0711 (0.0465, 0.0957)	0.0776 (0.0296, 0.1256)	0.0638 (0.0133, 0.1142)	0.0414 (0.0094, 0.0734)	-0.0221 (-0.0568, 0.0127)	-0.0533 (-0.1019, -0.0046)	0.0654 (0.0016, 0.1293)	0.0237 (-0.0163, 0.0636)
	2011	0.0234 (0.0063, 0.0405)	0.0776 (0.0308, 0.1243)	-0.0505 (-0.0930, -0.0079)	-0.0355 (-0.0779, 0.0070)	0.0587 (0.0273, 0.0901)	0.0412 (-0.0058, 0.0882)	0.0657 (0.0127, 0.1188)	0.0443 (0.0119, 0.0766)	-0.0258 (-0.0612, 0.0097)	-0.0687 (-0.1153, -0.0221)	0.0634 (0.0003, 0.1264)	0.0269 (-0.0107, 0.0644)

Sex	Year	Location											
		Global	High SDI	High-middle SDI	Middle SDI	Low-middle SDI	Low SDI	AFR	EMR	EUR	AMR	SEAR	WPR
	2012	0.0252 (0.0084, 0.0421)	0.0742 (0.0306, 0.1179)	-0.0606 (-0.1044, -0.0168)	-0.0424 (-0.0783, -0.0064)	0.0669 (0.0345, 0.0994)	0.0391 (-0.0105, 0.0888)	0.0684 (0.0135, 0.1234)	0.0454 (0.0131, 0.0777)	-0.0320 (-0.0693, 0.0053)	-0.0706 (-0.1166, -0.0246)	0.0582 (-0.0084, 0.1247)	0.0254 (-0.0081, 0.0589)
	2013	0.0351 (0.0179, 0.0524)	0.0786 (0.0374, 0.1198)	-0.0535 (-0.1009, -0.0061)	-0.0313 (-0.0627, 0.0002)	0.0900 (0.0551, 0.1250)	0.0272 (-0.0261, 0.0805)	0.0737 (0.0162, 0.1311)	0.0444 (0.0128, 0.0761)	-0.0379 (-0.0771, 0.0012)	-0.0721 (-0.1173, -0.0270)	0.0686 (-0.0174, 0.1546)	0.0247 (-0.0066, 0.0561)
	2014	0.0436 (0.0257, 0.0615)	0.0831 (0.0414, 0.1248)	-0.0568 (-0.1052, -0.0084)	-0.0445 (-0.0697, -0.0193)	0.1029 (0.0554, 0.1505)	0.0258 (-0.0269, 0.0785)	0.0772 (0.0198, 0.1346)	0.0489 (0.0153, 0.0825)	-0.0430 (-0.0840, -0.0020)	-0.0738 (-0.1182, -0.0294)	0.0798 (-0.0225, 0.1821)	0.0239 (-0.0071, 0.0549)
	2015	0.0451 (0.0269, 0.0634)	0.0880 (0.0462, 0.1299)	-0.0506 (-0.0997, -0.0015)	-0.0525 (-0.0755, -0.0295)	0.0448 (-0.0116, 0.1012)	0.0262 (-0.0303, 0.0827)	0.0824 (0.0229, 0.1418)	0.0522 (0.0179, 0.0864)	-0.0444 (-0.0873, -0.0016)	-0.0615 (-0.1080, -0.0150)	0.0798 (-0.0250, 0.1845)	0.0260 (-0.0051, 0.0572)
	2016	0.0461 (0.0282, 0.0640)	0.0823 (0.0420, 0.1226)	-0.0500 (-0.0971, -0.0029)	-0.0473 (-0.0691, -0.0256)	0.0392 (-0.0128, 0.0911)	-0.0015 (-0.0652, 0.0622)	0.0839 (0.0246, 0.1433)	0.0509 (0.0158, 0.0860)	-0.0451 (-0.0858, -0.0045)	-0.0599 (-0.1061, -0.0137)	0.0794 (-0.0207, 0.1795)	0.0257 (-0.0056, 0.0570)
	2017	0.0495 (0.0317, 0.0672)	0.0781 (0.0392, 0.1170)	0.0148 (-0.0305, 0.0602)	-0.0528 (-0.0721, -0.0334)	0.0391 (-0.0178, 0.0961)	0.0933 (0.0411, 0.1455)	0.0884 (0.0273, 0.1494)	0.0538 (0.0170, 0.0906)	-0.0259 (-0.0646, 0.0128)	-0.0605 (-0.1066, -0.0144)	0.0852 (-0.0139, 0.1843)	0.0248 (-0.0073, 0.0569)
	2018	0.0531 (0.0356, 0.0706)	0.0696 (0.0315, 0.1077)	-0.0022 (-0.0381, 0.0338)	-0.0564 (-0.0759, -0.0370)	0.0362 (-0.0221, 0.0944)	0.0981 (0.0443, 0.1518)	0.0937 (0.0338, 0.1537)	0.0557 (0.0183, 0.0932)	-0.0260 (-0.0613, 0.0094)	-0.0588 (-0.1045, -0.0130)	0.0856 (-0.0154, 0.1867)	0.0240 (-0.0084, 0.0565)
	2019	0.0574 (0.0399, 0.0749)	0.0666 (0.0293, 0.1039)	-0.0012 (-0.0345, 0.0320)	-0.0575 (-0.0775, -0.0375)	0.0225 (-0.0308, 0.0758)	0.0732 (0.0030, 0.1434)	0.0963 (0.0346, 0.1580)	0.0610 (0.0218, 0.1002)	-0.0259 (-0.0595, 0.0076)	-0.0510 (-0.0971, -0.0050)	0.0861 (-0.0166, 0.1888)	0.0259 (-0.0071, 0.0588)
	2020	0.0478 (0.0313, 0.0643)	0.0591 (0.0229, 0.0952)	0.0427 (0.0140, 0.0715)	0.0368 (0.0090, 0.0646)	0.0608 (0.0163, 0.1054)	0.0779 (0.0030, 0.1529)	0.0990 (0.0384, 0.1597)	0.0590 (0.0209, 0.0970)	-0.0248 (-0.0583, 0.0087)	-0.0485 (-0.0938, -0.0031)	0.0679 (-0.0025, 0.1384)	0.0308 (-0.0029, 0.0645)
	2021	0.0358 (0.0199, 0.0518)	0.0386 (0.0015, 0.0756)	0.0187 (-0.0080, 0.0455)	0.0296 (0.0018, 0.0575)	0.0809 (0.0398, 0.1221)	0.0712 (-0.0049, 0.1473)	0.1030 (0.0398, 0.1662)	0.0598 (0.0213, 0.0984)	-0.0256 (-0.0560, 0.0047)	-0.0554 (-0.0998, -0.0110)	0.0522 (0.0064, 0.0979)	0.0082 (-0.0271, 0.0434)

Notes: ADRDs, Alzheimer's disease and related dementia; SDI, Socio-demographic Index; WHO, World Health Organization; AFR, African Region; EMR, Eastern Mediterranean Region; AMR, Region of the Americas; EUR, European Region; SEAR, South-East Asia Region; WPR, Western Pacific Region.

Supplementary figures

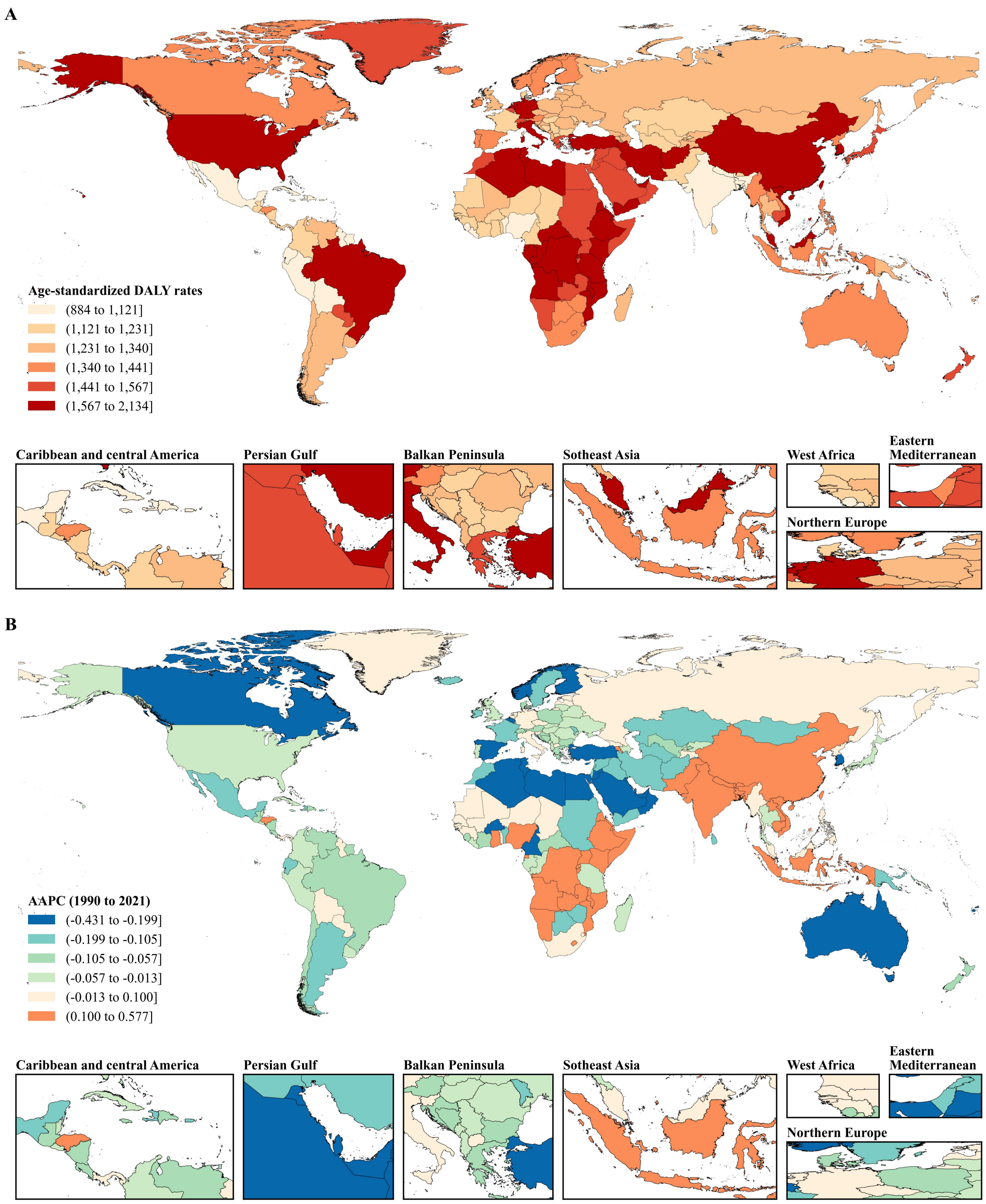

Figure S1. Age-standardized DALY rates of ADRDs in females across all countries in 2021 (A) and AAPC from 1990 to 2021 (B)

Notes: DALY, Disability-adjusted life year; ADRDs, Alzheimer's disease and related dementia; AAPC, Average annual percentage change.

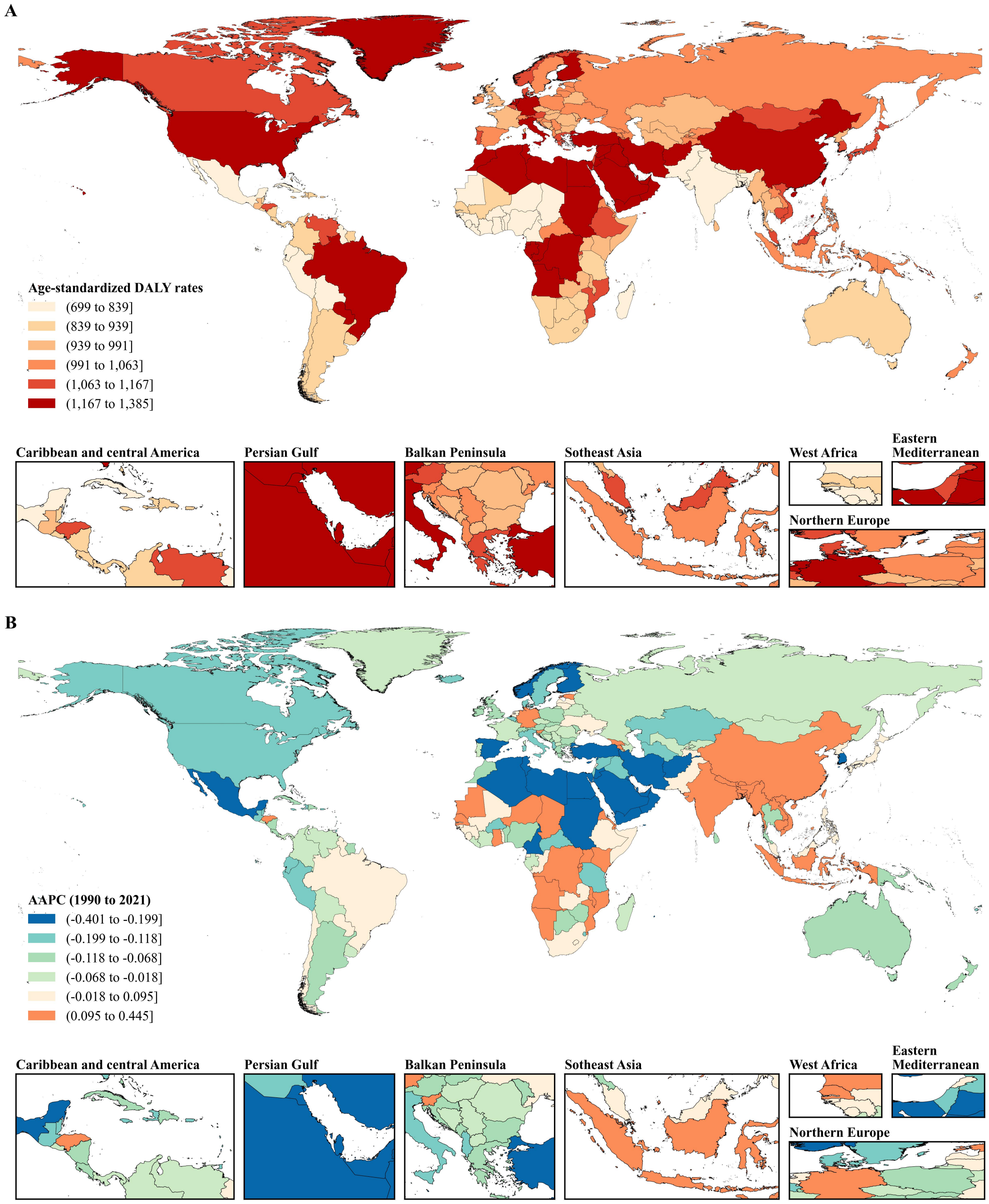

Figure S2. Age-standardized DALY rates of ADRDs in males across all countries in 2021 (A) and AAPC from 1990 to 2021 (B)

Notes: DALY, Disability-adjusted life year; ADRDs, Alzheimer's disease and related dementia; AAPC, Average annual percentage change.

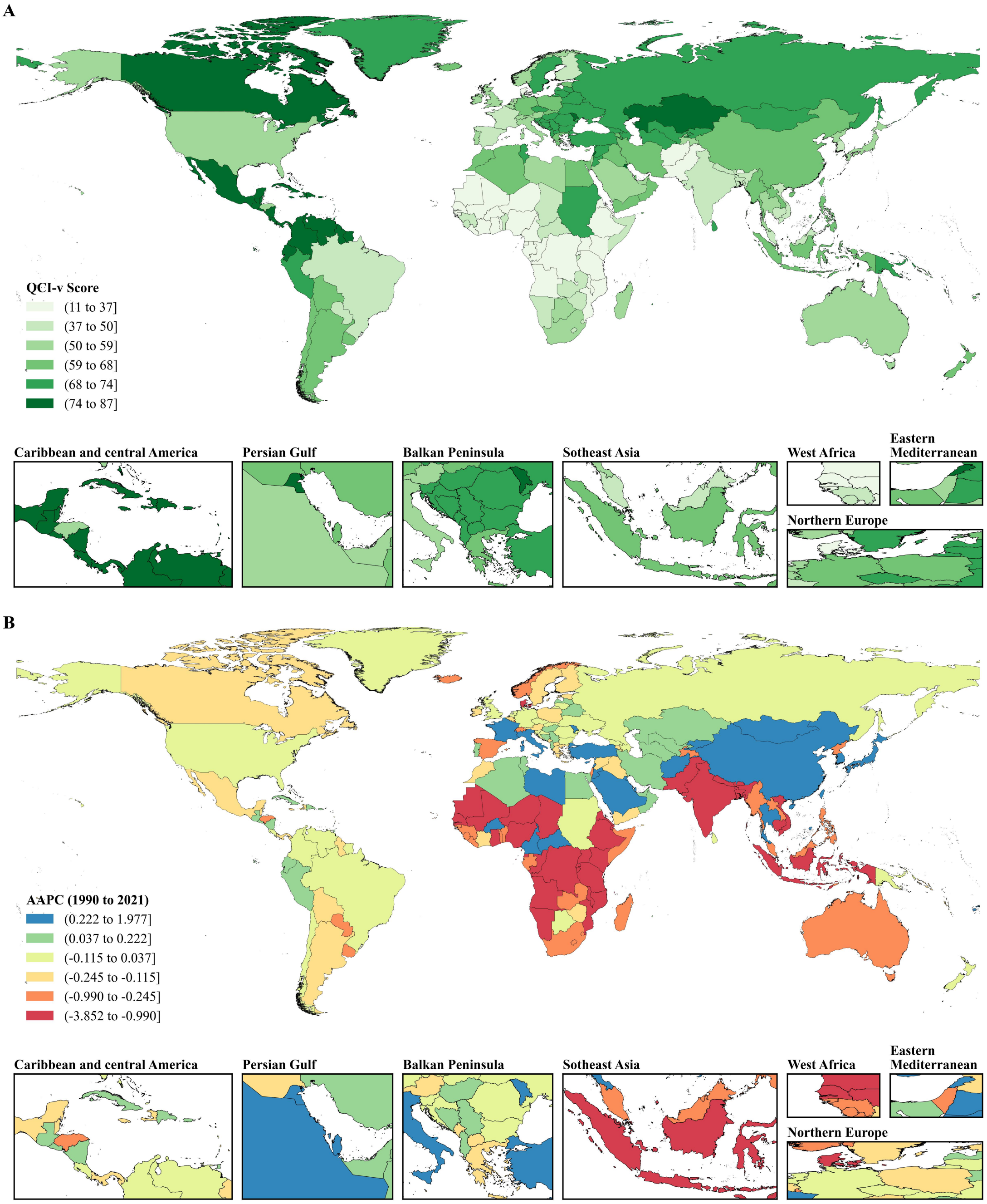

Figure S3. Age-standardized QCI-v of ADRDs in females across all countries in 2021 (A) and AAPC from 1990 to 2021 (B)

Notes: QCI-v, Quality of care index based on variational autoencoders; ADRDs, Alzheimer's disease and related dementia; AAPC, Average annual percentage change.

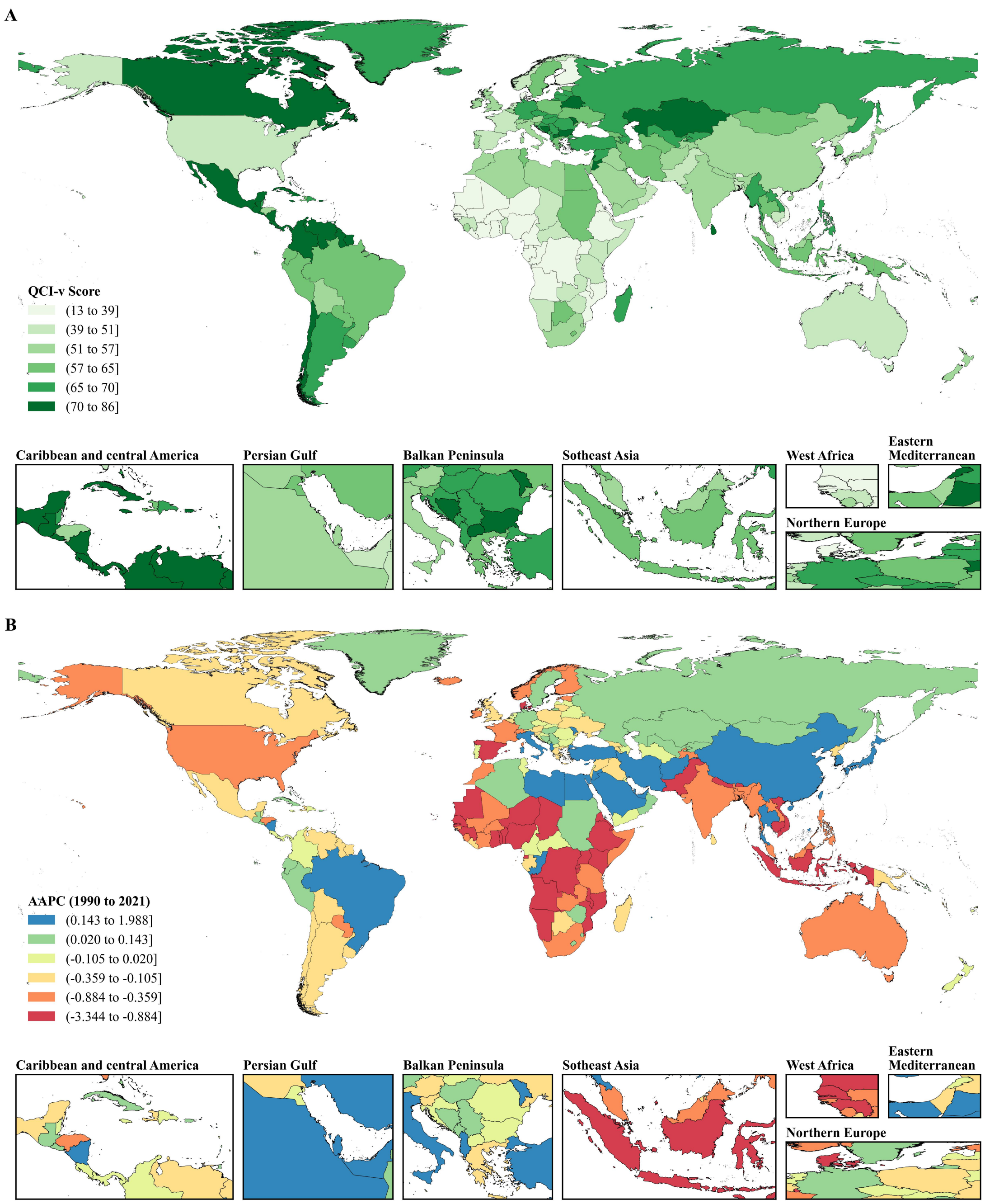

Figure S4. Age-standardized QCI-v of ADRDs in males across all countries in 2021 (A) and AAPC from 1990 to 2021 (B)

Notes: QCI-v, Quality of care index based on variational autoencoders; ADRDs, Alzheimer's disease and related dementia; AAPC, Average annual percentage change.

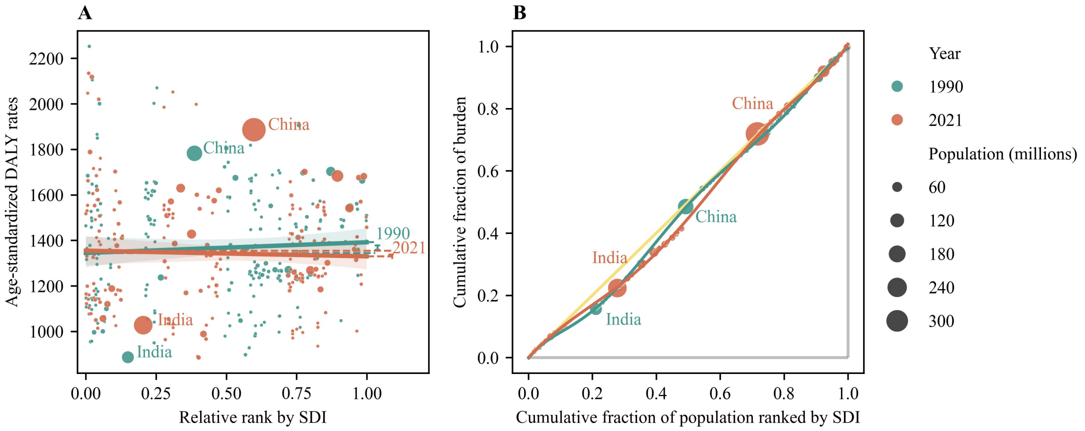

Figure S5. Absolute and relative inequalities in the age-standardized DALY rates of ADRDs in females worldwide, 1990 and 2021

Notes: A) the slope index of inequality, shown as the slope of the regression line, represents the absolute difference in ADRDs burden between countries or territories with the highest and lowest SDI. B) concentration curves (Lorenz curves) graphed by plotting the cumulative fraction of population ranked by SDI (x-axis) against the cumulative fraction of ADRDs burden ranked by age-standardised DALY (y-axis). The concentration index, calculated as twice the area between the 45° diagonal line and the Lorenz curve, representing the relative extent to which the ADRDs burden is concentrated among the poor (negative value) or the rich (positive value).

Dots representing countries or territories, with different sizes representing the population sizes. DALY, disability-adjusted life-year; ADRDs, Alzheimer's disease and related dementia; SDI, sociodemographic index.

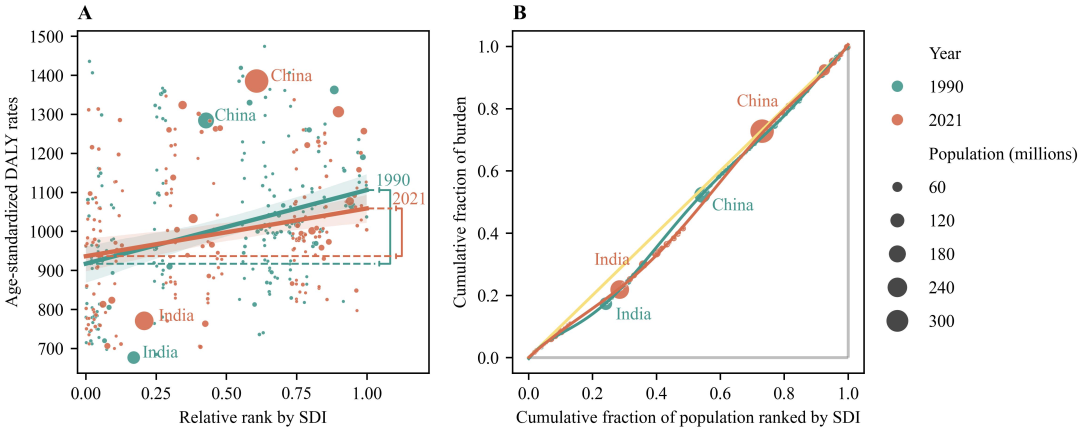

Figure S6. Absolute and relative inequalities in the age-standardized DALY rates of ADRDs in males worldwide, 1990 and 2021

Notes: A) the slope index of inequality, shown as the slope of the regression line, represents the absolute difference in ADRDs burden between countries or territories with the highest and lowest SDI. B) concentration curves (Lorenz curves) graphed by plotting the cumulative fraction of population ranked by SDI (x-axis) against the cumulative fraction of ADRDs burden ranked by age-standardised DALY (y-axis). The concentration index, calculated as twice the area between the 45° diagonal line and the Lorenz curve, representing the relative extent to which the ADRDs burden is concentrated among the poor (negative value) or the rich (positive value).

Dots representing countries or territories, with different sizes representing the population sizes. DALY, disability-adjusted life-year; ADRDs, Alzheimer's disease and related dementia; SDI, sociodemographic index.

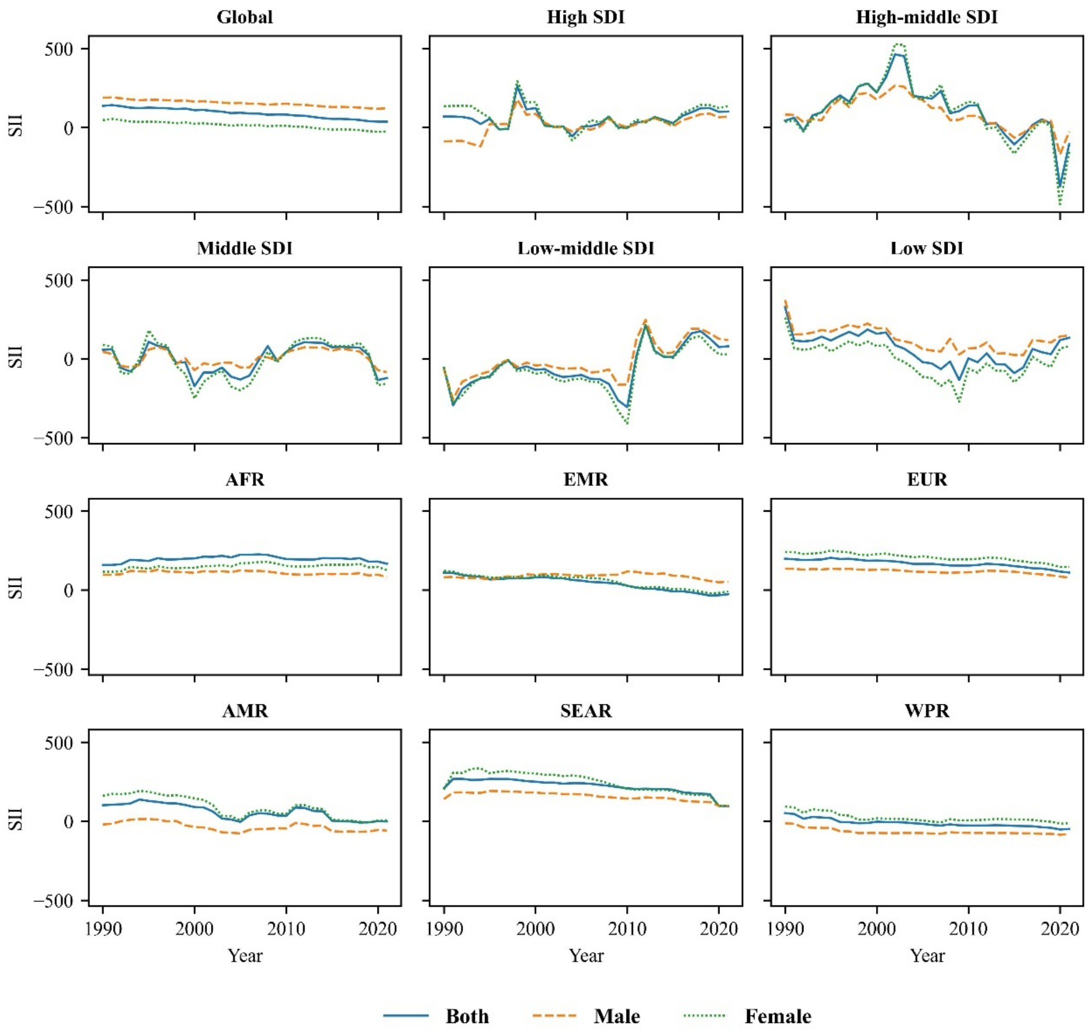

Figure S7. The year trends of slope index of inequality for burden of ADRDs by sex, SDI quantiles and WHO regions, from 1990 to 2021

Notes: ADRDs, Alzheimer's disease and related dementia; SDI, Socio-demographic Index; WHO, World Health Organization; AFR, African Region; EMR, Eastern Mediterranean Region; AMR, Region of the Americas; EUR, European Region; SEAR, South-East Asia Region; WPR, Western Pacific Region.

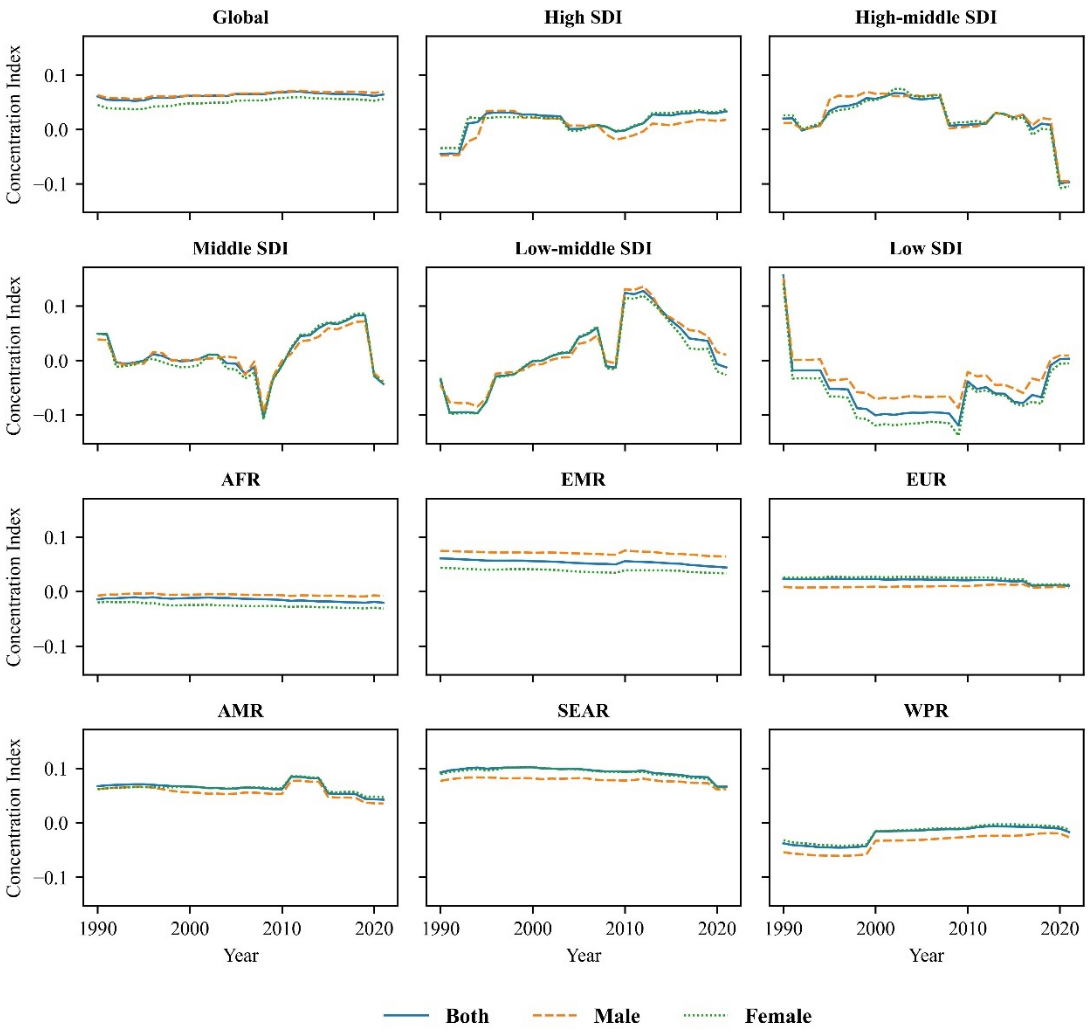

Figure S8. The year trends of concentration index for burden of ADRDs by sex, SDI quantiles and WHO regions, from 1990 to 2021

Notes: ADRDs, Alzheimer's disease and related dementia; SDI, Socio-demographic Index; WHO, World Health Organization; AFR, African Region; EMR, Eastern Mediterranean Region; AMR, Region of the Americas; EUR, European Region; SEAR, South-East Asia Region; WPR, Western Pacific Region.

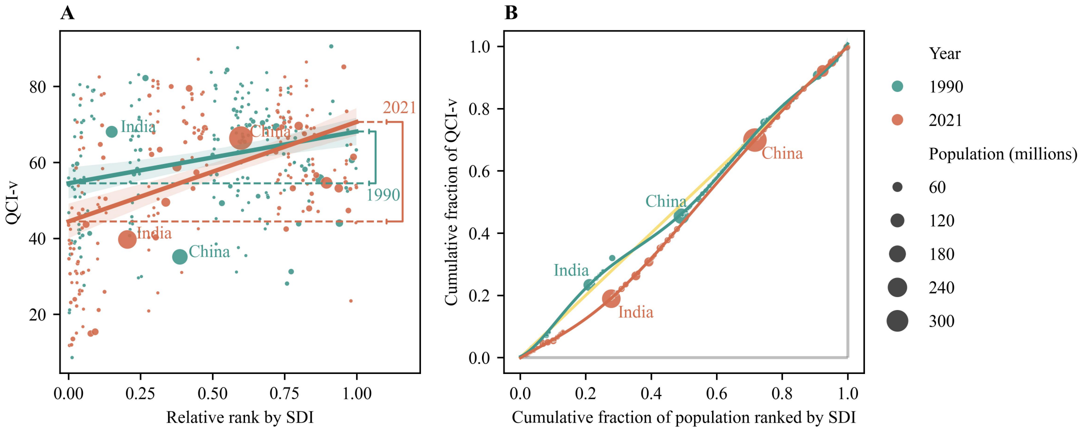

Figure S9. Absolute and relative inequalities in the age-standardized QCI-v of ADRDs in females worldwide, 1990 and 2021

Notes: A) the slope index of inequality, shown as the slope of the regression line, represents the absolute difference in ADRDs care quality between countries or territories with the highest and lowest SDI. B) concentration curves (Lorenz curves) graphed by plotting the cumulative fraction of population ranked by SDI (x-axis) against the cumulative fraction of ADRDs burden ranked by age-standardised QCI (y-axis). The concentration index, calculated as twice the area between the 45° diagonal line and the Lorenz curve, representing the relative extent to which the ADRDs care quality is concentrated among the poor (negative value) or the rich (positive value).

Dots representing countries or territories, with different sizes representing the population sizes. QCI-v, quality of care index based on variational autoencoders; DALY, disability-adjusted life-year; ADRDs, Alzheimer's disease and related dementia; SDI, socio-demographic index.

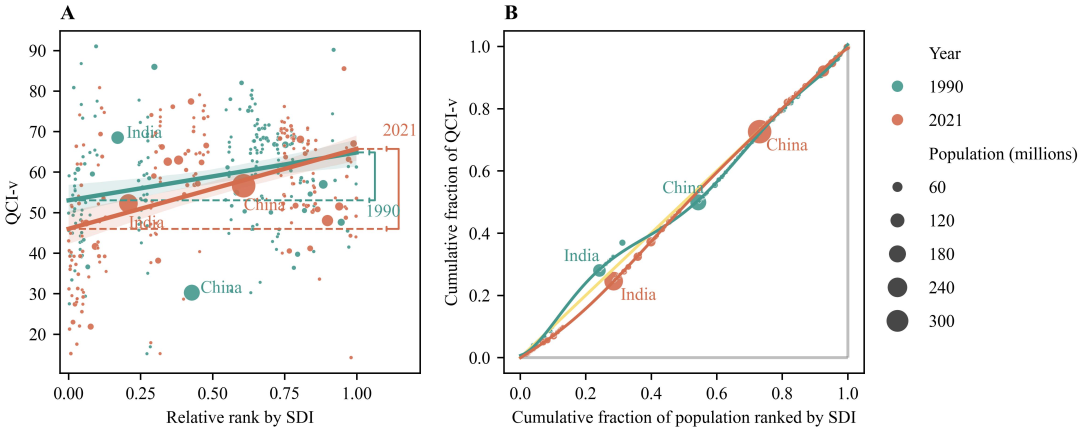

Figure S10. Absolute and relative inequalities in the age-standardized QCI-v of ADRDs in males worldwide, 1990 and 2021

Notes: A) the slope index of inequality, shown as the slope of the regression line, represents the absolute difference in ADRDs care quality between countries or territories with the highest and lowest SDI. B) concentration curves (Lorenz curves) graphed by plotting the cumulative fraction of population ranked by SDI (x-axis) against the cumulative fraction of ADRDs burden ranked by age-standardised QCI (y-axis). The concentration index, calculated as twice the area between the 45° diagonal line and the Lorenz curve, representing the relative extent to which the ADRDs care quality is concentrated among the poor (negative value) or the rich (positive value).

Dots representing countries or territories, with different sizes representing the population sizes. QCI-v, quality of care index based on variational autoencoders; DALY, disability-adjusted life-year; ADRDs, Alzheimer's disease and related dementia; SDI, socio-demographic index.

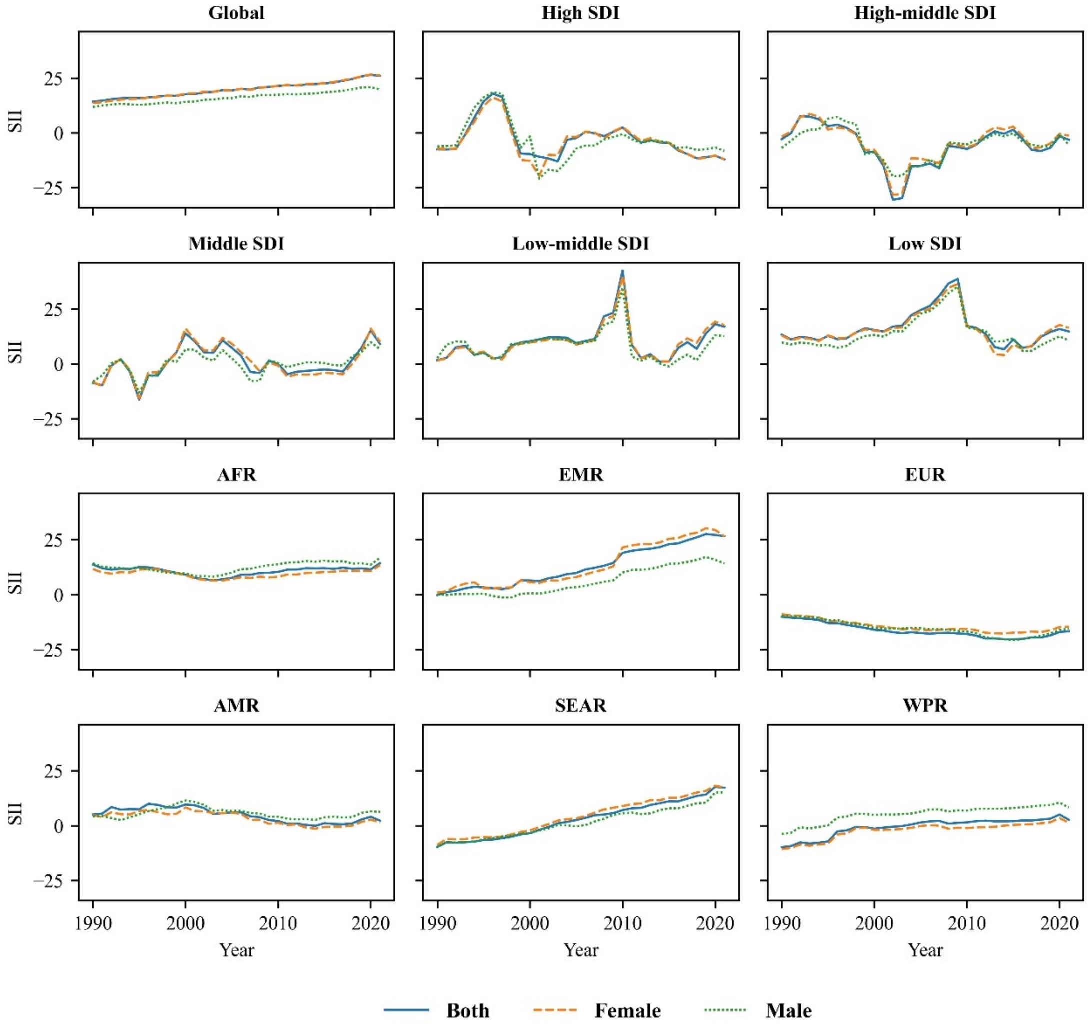

Figure S11. The year trends of slope index of inequality for care quality of ADRDs by sex, SDI quantiles and WHO regions, from 1990 to 2021

Notes: ADRDs, Alzheimer's disease and related dementia; SDI, Socio-demographic Index; AFR, African Region; EMR, Eastern Mediterranean Region; AMR, Region of the Americas; EUR, European Region; SEAR, South-East Asia Region; WPR, Western Pacific Region.

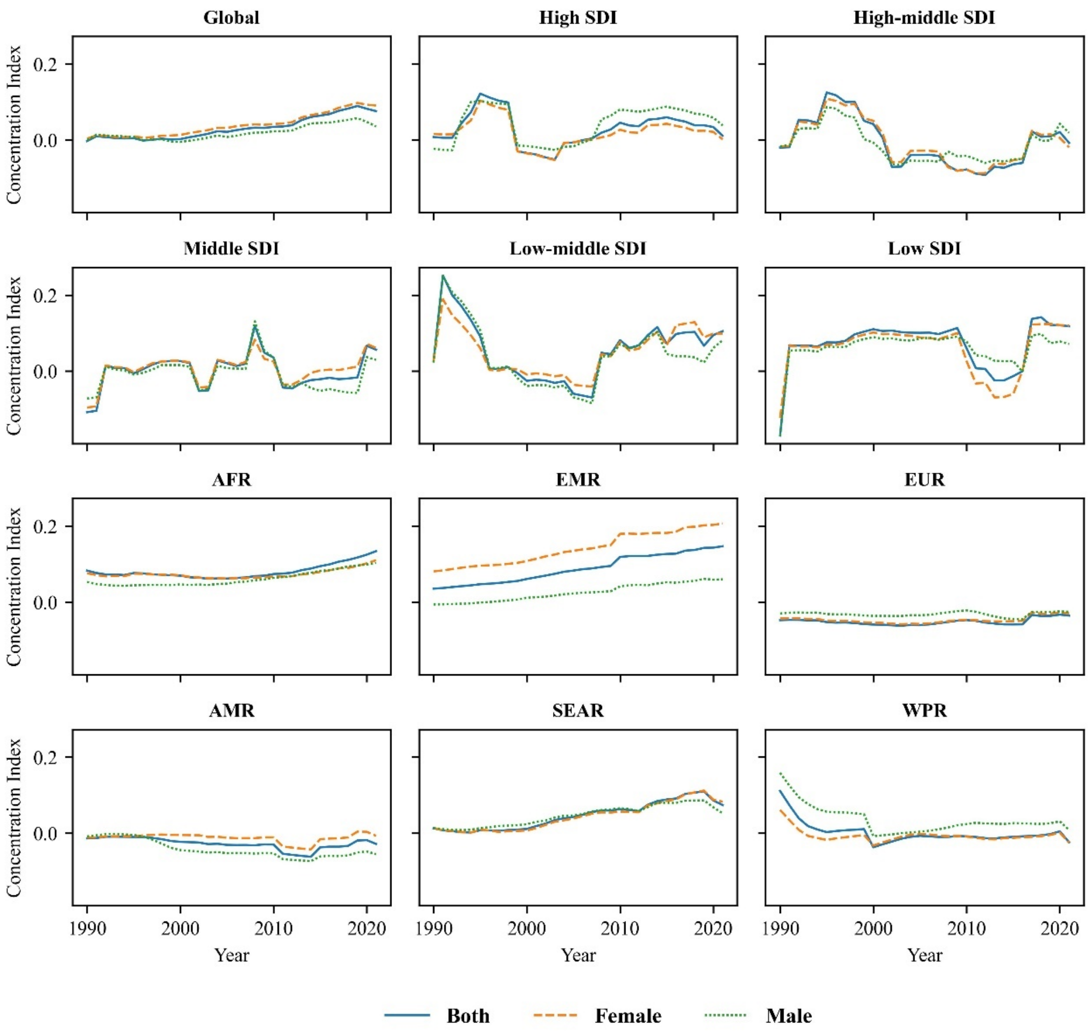

Figure S12. The year trends of concentration index for care quality of ADRDs by sex, SDI quantiles and WHO regions, from 1990 to 2021

Notes: ADRDs, Alzheimer's disease and related dementia; SDI, Socio-demographic Index; AFR, African Region; EMR, Eastern Mediterranean Region; AMR, Region of the Americas; EUR, European Region; SEAR, South-East Asia Region; WPR, Western Pacific Region.
